# Phenomic Impact of Genetically-Determined Euthyroid Function and Molecular Differences between Thyroid Disorders

**DOI:** 10.3390/jcm7100296

**Published:** 2018-09-21

**Authors:** Silvia Ravera, Nancy Carrasco, Joel Gelernter, Renato Polimanti

**Affiliations:** 1 Department of Cellular and Molecular Physiology, Yale School of Medicine, New Haven, CT 06510, USA; silvia.ravera@yale.edu (S.R.); nancy.carrasco@yale.edu (N.C.); 2 Department of Psychiatry, Yale School of Medicine and VA CT Healthcare Center, West Haven, CT 06516, USA; joel.gelernter@yale.edu; 3 Departments of Genetics and Neuroscience, Yale University School of Medicine, New Haven, CT 06510, USA

**Keywords:** thyroid gland, thyrotropin, free thyroxine, Mendelian randomization, causality

## Abstract

Background: The thyroid plays a key role in development and homeostasis, but it has been difficult to establish causality with diseases and phenotypic traits because of several potential confounders. Methods: To determine the causal effect of euthyroid function, we conducted a two-sample Mendelian randomization study of euthyroid thyrotropin (TSH) and free thyroxine (FT4) levels with respect to 2419 traits assessed in 337,199 individuals from UK Biobank. Additionally, we investigated the molecular differences between hypothyroidism and hyperthyroidism using genome-wide data. Results: After multiple testing correction, sixteen traits appear to be affected by genetically-determined euthyroid TSH, including multiple thyroid-related traits, e.g., hypothyroidism (*p* = 2.39 × 10^−17^), height (*p* = 2.76 × 10^−10^), body fat distribution (impedance of whole body, *p* = 4.43 × 10^−8^), pulse rate (*p* = 2.84 × 10^−8^), female infertility (*p* = 4.91 × 10^−6^), and hearing aid use (*p* = 7.10 × 10^−5^). Moreover, we found a consistent genetic correlation between hypothyroidism and hyperthyroidism (*rg* = 0.45, *p* = 5.45 × 10^−6^) with several immune pathways shared between these diseases. Two molecular pathways survived multiple testing correction for specificity to hyperthyroidism, JAK/STAT signaling (*p* = 1.02 × 10^−6^) and Rac guanyl-nucleotide exchange factor activity (*p* = 4.39 × 10^−6^). Conclusion: Our data shed new light on the inter-individual variability of euthyroid function and the molecular mechanisms of the two thyroid disorders investigated.

## 1. Introduction

Thyroid hormones play a key role in the regulation of a wide range of biological processes and functions with a particular relevance in the early stages of life [1]. The function of the thyroid gland is finely regulated by the negative feedback loop of the hypothalamus-pituitary-thyroid axis: low thyroid hormone levels induce the hypothalamus to release thyrotropin-releasing hormone, which causes the pituitary gland to release thyroid stimulating hormone (TSH), which stimulates the thyroid to synthesize and release the pro-hormone thyroxine (FT4) in the blood stream [1]. Altered thyroid function (and consequent hyper- or hypothyroidism) can be associated with severe symptoms affecting multiple organs, including heart, brain, and bone, and general metabolism [2]. Subjects with subclinical hypo- and hyperthyroidism (no or mild symptoms, TSH concentration above or below the reference range, and normal FT4 concentration) also have increased risk for adverse outcomes [3,4,5]. In euthyroid individuals, the variability of TSH and FT4 levels has been associated with atrial fibrillation, bone fracture, dementia, and mortality [6,7]. There is thus a continuum of health outcomes across the spectrum of thyroid function, even in euthyroid individuals. The inter-individual variability of TSH and FT4 levels within the euthyroid range is a multifactorial trait, and 40–65% of the variance in these traits is explained by genetic factors [8]. Genome-wide association studies (GWAS) and a whole-genome sequence-based analysis have identified several common variants associated with TSH and FT4 levels in euthyroid individuals [9,10,11]. This genetic information can be used to investigate the medical consequences of the spectrum of thyroid function: Mendelian randomization (MR) is a strategy in which evidence on the associations of genetic variants (i.e., instrumental variable) with the risk factor (i.e., exposure) and with the outcome can be used to test the hypothesis that the exposure causes the outcome [12]. In contrast to observational analyses, studies based on genetically-determined traits cannot be biased by environmental confounders [13,14,15,16]. This is highly relevant when TSH and FT4 levels are investigated because thyroid function is affected by numerous external factors, such as age, diet, smoking, pollutants, and infections [17,18,19]. Previous MR analyses focused on specific hypotheses testing the causal relationship of thyroid function with respect to bone mineral density, ischemic heart disease, insulin resistance, and type 2 diabetes [20,21,22].

Due to its pivotal regulatory role in numerous biological processes, we hypothesize that thyroid function has a broad impact on the human phenome; MR provides an unprecedented opportunity to test this hypothesis. Phenome-wide analysis can permit to discover novel phenotypic relationships of loci previously identified [23,24,25]. Therefore, we conducted a phenome-wide Mendelian randomization analysis of thyroid function that investigated 2419 traits in 337,199 individuals from the UK Biobank [26]. Furthermore, we analyzed the polygenic architecture of hyper- and hypothyroidism, probing the mechanisms shared between these two thyroid disorders and those that differentiate them.

## 2. Materials and Methods

### 2.1. TSH and FT4 Genetic Instruments

TSH and FT4 genetic instruments were derived combining alleles that reached genome-wide significance (*p* < 5 × 10^−8^) in two previous GWAS of thyroid function in euthyroid subjects [9,10]. Each study included multiple cohorts and some of the cohorts investigated are present in both studies. In TSH GWAS, the sample size ranged between 11,544 and 16,335 individuals in one study [10], and between 23,482 and 26,054 individuals in the second study [9]. In the FT4 GWAS, the first study [10] included 13,650 individuals, while the sample size of the second study [9] was between 10,997 and 14,529 subjects. Combining the data of the two studies, we excluded alleles with linkage disequilibrium (LD) *r*^2^ ≥ 0.01, keeping the alleles with the strongest association. The final genetic instruments included 24 and five variants for TSH and FT4, respectively (Table S1).

### 2.2. UK Biobank Summary Association Data

The UK Biobank cohort is an open access resource available to investigate a wide range of serious and life-threatening illnesses [26]. This project has recruited more than 500,000 people assessed through detailed web-based questionnaires on their diet, cognitive function, work history, health status, and other relevant phenotypes. To date, complete genome-wide data are available for the entire cohort. All genetic and phenotypic data are available for bona fide scientists through an access application. In our MR analysis, the outcome information was derived from GWAS summary association data regarding 2419 traits (Table S2) assessed in up to 337,199 unrelated individuals of British descent. GWAS summary association data related to hyperthyroidism (2547 cases and 334,612 controls) and hypothyroidism (16,376 cases and 320,783 controls) were used to investigate the polygenic architecture of these two disorders. Details regarding QC criteria, GWAS methods, and the original data are available at https://github.com/Nealelab/UK_Biobank_GWAS/tree/master/imputed-v2-gwas.

### 2.3. Two-Sample Mendelian Randomization

To dissect causality between thyroid function and the human phenome, we conducted a two-sample MR analysis using TSH and FT4 genetic instruments with respect to outcome data from the UK Biobank. Since different MR methods have sensitivities to different potential issues, accommodate different scenarios, and vary in their statistical efficiency, we considered 28 MR approaches (Table S3). These included methods based on median [27], mean [28], and mode [29]; and various adjustments within these MR methods, including fixed vs. random effects [27], Rucker framework [30], and Steiger filtering [31]. We verified the stability of the results, comparing the results across the different MR methods. MR-Egger regression intercept was considered to verify the presence of pleiotropic effects of the single-nucleotide polymorphisms (SNPs) on the outcome (i.e., to verify whether the instrumental variable is associated with the outcome independently from its association with the exposure) [32]. We applied a false discovery rate (FDR) correction (*q* < 0.05) to correct for the multiple testing considering the number of outcomes tested (*n* = 2419) and the number of MR methods considered (*n* = 28). The MR analyses were conducted using the TwoSampleMR R package [33].

### 2.4. Genetic Correlation and Enrichment Analysis

Based on GWAS summary association data related to hyper- and hypothyroidism, we investigated the polygenic architecture of these disorders with respect to other complex traits and molecular pathways and gene ontology (GO) annotations. Heritability estimates and genetic correlations based on UK Biobank GWAS summary association data were calculated using the LD score regression (LDSC) method [34]. Enrichment analysis for molecular pathways and GO annotations was conducted using MAGMA tool [35] implemented in FUMA platform [36]. Bonferroni correction was applied to adjust the results of genetic-correlation and enrichment analyses for the number of phenotypes (*n* = 1578) and annotations tested (*n* = 10,891), respectively.

## 3. Results

### 3.1. Two-Sample Mendelian Randomization of Thyroid Function

We conducted a two-sample Mendelian randomization analysis [12] using genetic instruments (Table S1) derived from two previous genome-wide studies of TSH and FT4 levels in euthyroid individuals [9,10] to ascertain the causal effects of thyroid function on the human phenome. After FDR multiple testing correction accounting for the number of traits tested and MR methods used, 29 phenotypes resulted significantly affected by genetically-determined TSH levels (*q* < 0.05; Table S4). To ascertain the reliability of these results, we determined their concordance across the different MR methods used and tested for the presence or absence of pleiotropy using the MR-Egger regression intercept. Sixteen of the 29 phenotypes showed consistency across MR methods and did not show pleiotropy with respect to the TSH genetic instrument (*p* > 0.05; Table S5). Table 1 reports the details of the significant findings that passed the sensitivity analyses.

As expected, the strongest results were observed with respect to thyroid-related traits, where increased TSH levels were associated with increased risk of hypothyroidism (*β* = 0.02, *p* = 2.39 × 10^−17^), levothyroxine sodium use (*β* = 0.017, *p* = 2.41 × 10^−13^), thyroxine product use (*β* = 0.005, *p* = 3.34 × 10^−8^), and reduced risk of non-toxic goiter (*β* = −0.002, *p* = 9.45 × 10^−14^), hyperthyroidism (*β* = −0.004, *p* = 7.49 × 10^−8^), and non-cancer thyroid problems (*β* = −0.001, *p* = 3.71 × 10^−6^). Genetically determined TSH levels also appear to affect anthropometric traits related to body fat distribution negatively (i.e., impedance of left arm: *β* = −0.029, *p* = 2.22 × 10^−13^; impedance of right arm: *β* = −0.024, *p* = 9.6 × 10^−12^; impedance of whole body: *β* = −0.022, *p* = 4.43 × 10^−8^; hip circumference: *β* = −0.022, *p* = 1.51 × 10^−5^; trunk fat percentage: *β* = −0.048, *p* = 2.23 × 10^−5^) and to adulthood and childhood standing height (*β* = −0.023, *p* = 2.76 × 10^−10^; comparative height size at age 10: *β* = −0.028, *p* = 1.74 × 10^−9^). Additional causal associations with TSH levels were observed for pulse rate (*β* = −0.037, *p* = 2.84 × 10^−8^), female infertility (*β* = 0.001, *p* = 4.91 × 10^−6^), and hearing aid use (*β* = −0.005, *p* = 7.1 × 10^−5^). No result survived FDR multiple testing correction in the MR analysis based on FT4 genetic instrument.

### 3.2. Genetic Differences between Hyperthyroidism and Hypothyroidism

**Figure 1:**
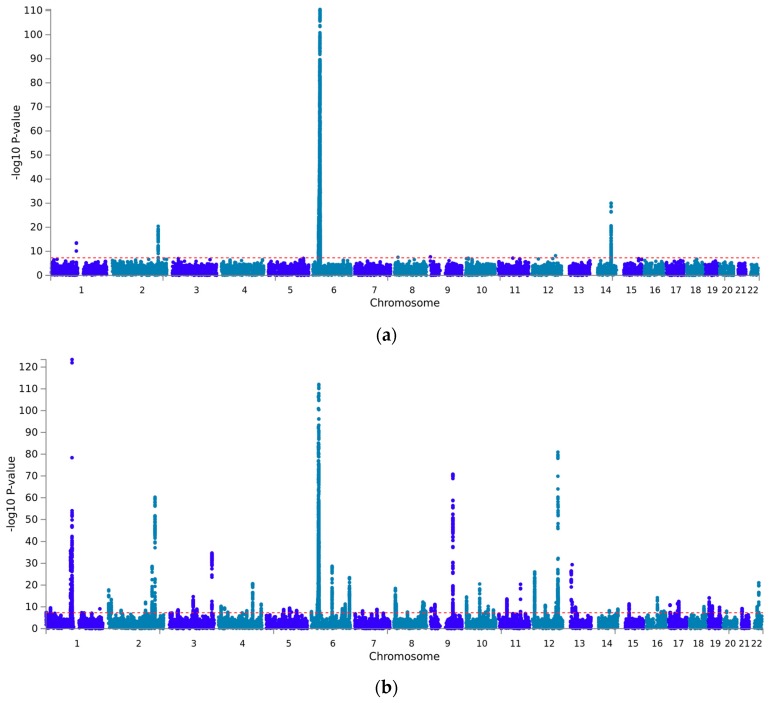
Manhattan plots of hyperthyroidism and hypothyroidism genome-wide association studies (GWAS) (**a** and **b**, respectively). Red dotted line indicates genome-wide significance (*p* < 5 × 10^−8^). Genomic risk loci identified are reported in Tables S6 and S7.

Although hyper- and hypothyroidism have opposite phenotypic presentations, it is not uncommon to observe a conversion from hyperthyroidism to hypothyroidism but conversion from hypothyroidism to hyperthyroidism is very rare [37]. Leveraging the GWAS summary association data from UK Biobank, we analyzed the genetic architecture of hyper- and hypothyroidism, investigating the differences and commonalities in their pathogenesis. In hyperthyroidism GWAS (2547 cases and 334,612 controls; Figure 1A), five genomic risk loci were identified, including a total of 12 independent variants (LD *r*^2^ < 0.1; Table S6). In hypothyroidism analysis (16,376 cases and 320,783 controls; Figure 1B), 86 genomic risk loci were identified, including a total of 154 independent variants (LD *r*^2^ < 0.1; Table S7). Three genome-wide significant loci were associated with both disorders. On chromosome 1, rs2476601, a previously known missense variant located on *PTPN22* [38], was genome-wide significant in both hyper- and hypothyroidism analyses (*p*_hyperthyroidism_ = 3.15 × 10^−14^; *p*_hypothyroidism_ = 3.49 × 10^−124^). On chromosome 2, two variants, rs3087243 (*p*_hyperthyroidism_ = 3.80 × 10^−21^) and rs11571297 (*p*_hypothyroidism_ = 5.34 × 10^−61^) located 6 Kb from each other and previously investigated with respect to *CTL4* gene [39], present a strong LD (*r*^2^ = 0.891). In MHC region (chromosome 6), a strong LD (*r*^2^ = 0.612) was observed between rs13217620 (*p*_hyperthyroidism_ = 7.75 × 10^−27^) and rs200949 (*p*_hypothyroidism_ = 2.95 × 10^−12^).

**Figure 2:**
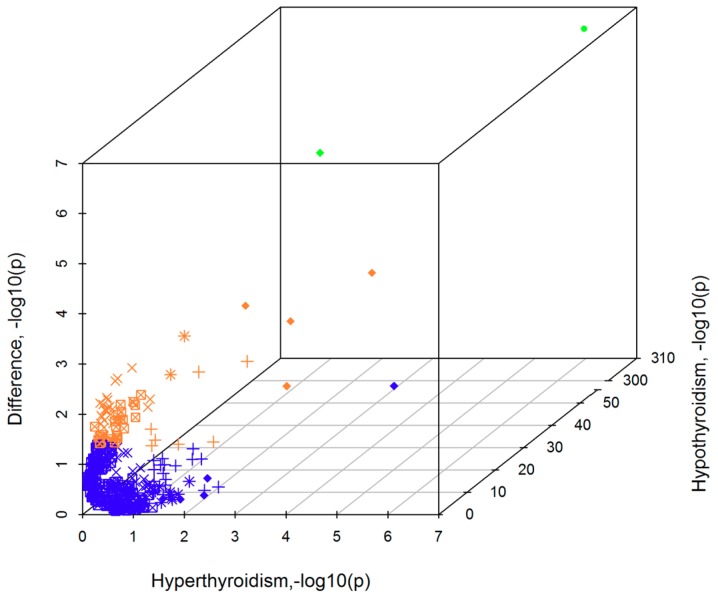
Three-dimensional scatter plot showing linkage disequilibrium score regression (LDSC) results, −log10(*p* value), for hyperthyroidism (*x*-axis), hypothyroidism (*z*-axis), and difference between the two disorders (*y*-axis). The plot includes the traits that showed at least nominally significant correlation with one of the two disorders. The color scheme reflects the significance of the comparison analysis between the two disorders: blue, non-significant difference; orange, nominally significant difference; and green, Bonferroni significant difference. The marker scheme reflects the significance of the analysis conducted with respect to each disorder: “circle” for Bonferroni significance in both disorders; “diamond” for Bonferroni significance in hypothyroidism and nominal significance in hyperthyroidism; “cross” for Bonferroni significance in hypothyroidism and non-significance in hyperthyroidism; “star” for nominal significance in both disorders; “square-cross” for nominal significance in hypothyroidism and non-significance in hyperthyroidism; “plus” for nominal significance in hyperthyroidism and non-significance in hypothyroidism. Genetic-correlation results are reported in Table S8.

Common genetic variation accounted for 15% (*p* = 5.11 × 10^−4^) and 24% (*p* = 3.87 × 10^−21^) of the heritability of hyper- and hypothyroidism, respectively. We calculated genetic correlations between these disorders, and genetic correlations of each with respect to the other available phenotypic traits, testing the presence of significant differences between them. Forty-five percent of the genetic liability is shared between these two thyroid disorders (*rg* = 0.45, *p* = 5.45 × 10^−6^). Genetic correlation of these diseases was calculated with respect to 1578 phenotypic traits (Table S8). Figure 2 graphically shows differences and commonalities in the genetic-correlation analyses of hyper- and hypothyroidism.

After Bonferroni multiple testing correction, hyperthyroidism showed a significant genetic correlation with levothyroxine sodium use (*rg* = 0.48, *p* = 5.72 × 10^−8^). The same trait showed a much stronger genetic correlation with hypothyroidism (*rg* = 1, *p* < 4.5 × 10^−308^), suggesting that the correlation between levothyroxine sodium use and hyperthyroidism is driven by the fact that levothyroxine sodium use is a proxy of hypothyroidism. Hypothyroidism also showed significant correlations with fifty-seven other traits (Table 2), such as taking other prescription medications (*rg* = 49, *p* = 5.09 × 10^−52^), number of self-reported non-cancer illnesses (*rg* = 0.36, *p* = 2.53 × 10^−36^), anthropometric traits (e.g., waist circumference: *rg* = 0.17, *p* = 6.94 × 10^−11^), and behavioral traits (e.g., “seen a doctor for nerves, anxiety, tension or depression”: *rg* = 0.15, *p* = 1.24 × 10^−7^).

Comparing the results of the two thyroid disorders, we observed significant differences for levothyroxine sodium use (*z*_difference_ = −5.24, *p*_difference_ = 1.64 × 10^−7^) and taking other prescription medications (*z*_difference_ = −4.38, *p*_difference_ = 1.18 × 10^−5^): hypothyroidism showed much stronger genetic correlation than hyperthyroidism. Although it did not survive Bonferroni multiple testing correction, the strongest difference showing genetic correlations with opposite effect direction between the thyroid disorders was “walking for pleasure in the last 4 weeks,” where a positive correlation was observed with respect to hyperthyroidisms and a negative correlation was observed for hypothyroidism (*rg*_hyperthyroidism_ = 0.18, *p*_hyperthyroidism_ = 0.014, *rg*_hyperthyroidism_ = −0.12, *p*_hypothyroidism_ = 0.003; *z*_difference_ = 3.57, *p*_difference_ = 3.61 × 10^−4^).

**Figure 3:**
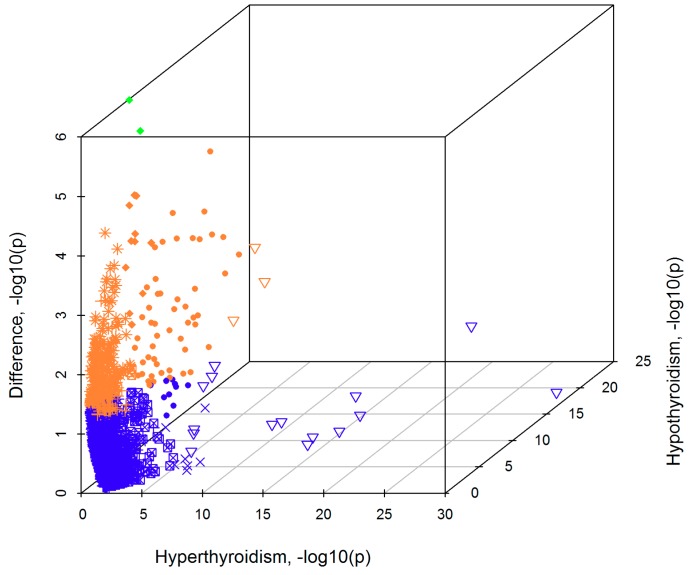
Three-dimensional scatter plot showing enrichment-analysis results, −log10(*p* value), for hyperthyroidism (*x*-axis), hypothyroidism (*z*-axis), and the comparison analysis between the two disorders (*y*-axis). The plot includes the molecular mechanisms that showed at least nominally significant enrichment with one of the two disorders. The color scheme reflects the significance of the comparison analysis between the two disorders: blue, non-significant difference; orange, nominally significant difference; and green, Bonferroni significant difference. The marker scheme reflects the significance of the analysis conducted with respect to each disorder: “point-down triangle” for Bonferroni significance in both disorders; “circle” for Bonferroni significance in hypothyroidism and nominal significance in hyperthyroidism; “diamond” for Bonferroni significance in hypothyroidism and non-significance in hyperthyroidism; “cross” for Bonferroni significance in hyperthyroidism and nominal significance in hypothyroidism; “square-cross” for nominal significance in both disorders; “star” for nominal significance in hypothyroidism and non-significance in hyperthyroidism; plus for nominal significance in hyperthyroidism and non-significance in hypothyroidism. Enrichments surviving Bonferroni multiple testing correction are reported in Table S9.

Additionally, we estimated the enrichment for molecular pathways from KEGG (Kyoto Encyclopedia of Genes and Genomes) database [40] and biological processes from GO annotations [41], with attention to the differences between the two thyroid disorders. Figure 3 graphically shows differences and commonalities in the two enrichment analyses.

Twenty-five and 92 enrichments survived Bonferroni multiple testing correction for hyper- and hypothyroidism, respectively (Table S9). Comparing the two disorders, 18 enrichments were significant after multiple testing correction in both analyses (Table 3) and these shared mechanisms include many gene sets related to immune function, such as GO~Major Histocompatibility Complex (*GO~MHC) class II protein complex* (*p*_hyperthyroidism_ = 4.58 × 10^−30^, *p*_hypothyroidism_ = 2.08 × 10^−18^) and *KEGG~autoimmune thyroid disease* (*p*_hyperthyroidism_ = 4.41 × 10^−21^, *p*_hypothyroidism_ = 6 × 10^−22^).

Two gene sets showed a specific enrichment for hypothyroidism: GO*~Rac guanyl-nucleotide exchange factor (GEF) activity* (*p*_hyperthyroidism_ = 0.90, *p*_hypothyroidism_ = 8.01 × 10^−8^; *z*_difference_ = −4.89, *p*_difference_ = 1.02 × 10^−6^) and *KEGG~JAK/STAT signaling pathway* (*p*_hyperthyroidism_ = 0.567; *p*_hypothyroidism_ = 4.41 × 10^−9^; *z*_difference_ = −4.59, *p*_difference_ = 4.38 × 10^−6^).

## 4. Discussion

The present study provides novel data regarding the consequences of inter-individual variability of thyroid function and the genetic similarities and differences between hypo- and hyperthyroidism. Genetically-determined TSH levels within the euthyroid range appear to be involved in causal mechanisms of several phenotypic traits. The strongest TSH findings were related to thyroid traits: increased genetically determined TSH levels were associated with increased risk of hypothyroidism, thyroid medication use, and reduced risk of hyperthyroidism, non-toxic goiter, and non-cancer thyroid problems. These results are in agreement with the findings of the 11-Year Follow-Up of the HUNT Study (>15,000 individuals): (i) high TSH within the reference range strongly positively associated with the risk of future hypothyroidism; (ii) TSH at the lower limit of the reference range may be associated with an increased risk of hyperthyroidism [42]. The relationship between TSH level and the risk of future thyroid disorders is likely due to a combination of genetic and environmental factors. Our findings clearly indicate that genetic predisposition to high or low TSH level within the reference range partially explains the risk of future hypo- or hyperthyroidism independent of non-genetic contributions. We also observed that increased genetically determined TSH levels within the reference range are associated with a reduced risk of non-toxic goiter. To our knowledge, no previous study has reported this relationship. However, a previous GWAS of hypothyroidism observed that the Forkhead box protein E1 (*FOXE1)* risk allele identified is associated with several thyroid phenotypes, including thyroiditis, nodular and multinodular goiters, and thyrotoxicosis [43].

In our MR analysis, we observed significant causal effects of thyroid function on several anthropometric traits. Increased genetically-determined TSH was associated with reduced adulthood standing height and comparative height size at age 10. Thyroid hormones are important for skeletal development [44]. Expression of thyroid hormone receptors has been shown in the resting and proliferative zones of the growth plate [45], which is the area of growing tissue near the ends of the long bones in children and adolescents. Both hypo- and hyperthyroidism during childhood and adolescence cause decreased final height via different mechanisms: the first induces a delay in skeletal maturation and growth arrest; the second advances skeletal maturation and growth acceleration, but due to the early fusion of the growth plates, the final outcome is reduced height [46]. In agreement with this, our data support the conclusion that increased TSH is also associated with reduced height development in euthyroid subjects.

Beyond height, further causal associations were observed between TSH and traits related to body fat distribution. There have been several studies that investigated the role of thyroid function with respect to adipose tissue accumulation, observing inadequate thyroid function in obese individuals [47], altered fat distribution in subjects with hypothyroidism [48], and associations between thyroid hormone levels and body fat accumulation [49]. Although there is a clear relationship between thyroid function and body fat, it is not easy to understand the causal direction, especially considering the molecular mechanisms involved in the cross-talk between thyroid gland and adipose tissue [50]. The results of our MR analysis support a causal role for TSH levels in determining body fat distribution in euthyroid individuals. Specifically, increased genetically determined TSH levels in euthyroid individuals cause high impedance values of the arms and of the whole body (which indicates increased levels of fat mass) and low values for hip circumference and trunk fat percentage (reduced fat percentage in the torso). Three recent studies investigated the association between TSH levels and visceral adipose tissue, with conflicting results. In a study by Chen et al. including 8727 subjects, TSH levels were positively associated with visceral adiposity index [51]. A study by Witte et al. conducted on 1977 individuals and a study conducted on the Framingham Third Generation cohorts (*n* = 3483) did not replicate this association [52,53]. The non-concordance between these analyses may be explained by different statistical power (i.e., Chen et al.’s sample size is larger than the other samples), or by adjustment of possible confounders (population-based differences for such basic functions seem highly unlikely). For example, Witte et al.’s analysis was adjusted by physical activity; this could affect TSH-visceral adiposity association, as also indicated by our hyper-/hypothyroidism genetic-correlation result for “walking for pleasure”. Other confounders that were not accounted for in these observational analyses, such as dietary habits (which, unlike basic metabolic factors, do differ greatly between the populations studied), may have biased the results. Our MR results, which are not biased by any such environmental confounders, indicated that TSH may affect body fat distribution, increasing whole body fat mass while reducing visceral adiposity.

Further causal effects of increased genetically determined TSH levels were observed for reduced pulse rate, increased risk of female infertility, and reduced likelihood of hearing aid use. Thyroid function modulates every major component of the cardiovascular system necessary for normal cardiovascular development and function [53]. Thyroid disorders and subclinical thyroid conditions are associated with heart diseases [54], but variability within the euthyroid range seems not to be associated with any pathological heart traits [55]. In line with these results, we observed a causal association between genetically-determined TSH and pulse rate, but no significant results for any traits related to heart disease.

In contrast to this latter finding, the observed TSH causal associations with the strikingly different traits of female infertility and hearing aid use indicate that the inter-individual variability of thyroid function within the reference range could be causal with respect to pathological conditions known to be associated with thyroid disorders. Hypothyroidism is associated with infertility and miscarriage, and subclinical hypothyroidism is a primary cause of subfertility, with a prevalence of 20% in infertile women [56,57,58,59]. Our data indicate that increased TSH within the euthyroid reference could also be associated with increased risk of infertility in euthyroid women. Clinical guidelines recommend the screening of serum TSH and FT4 levels in infertile women and women planning a pregnancy, including those planning to use assisted reproduction in the immediate future [60,61]. According to our analysis, a stricter TSH cutoff in women considering pregnancy may be beneficial in the clinical setting. Regarding the “hearing aid use” finding, hypo-, hyperthyroidism, and subclinical thyroid conditions have all been reported to be associated with hearing impairment [62,63,64]. To our knowledge, no observational data are currently available regarding the effect of thyroid function on hearing loss in euthyroid individuals. Our MR results indicate that low TSH may play a causal role in hearing impairment in euthyroid subjects, as reflected by the increased risk of hearing aid use. 

Our MR analysis based on FT4 genetic instruments did not reveal any significant results. This is likely due to the fact that few variants reached genome-wide significance in previous FT4 GWAS and they explain a smaller variance than those identified in TSH analysis (variance explained, FT4 = 2.3% vs. TSH = 5.6%) [10].

Using genome-wide data, we also investigated the polygenic architecture of hypo- and hyperthyroidism, analyzing commonalities and differences in the molecular pathways involved in their predisposition. These thyroid disorders show opposite phenotypic presentations: hyperthyroidism is characterized by high FT4 and low TSH, whereas primary hypothyroidism presents low FT4 and high TSH. On the trait level, it is not uncommon to observe a conversion from hyperthyroidism to hypothyroidism but the conversion from hypothyroidism to hyperthyroidism is very rare [37], although there may be a a substantial risk of developing a suppressed thyrotropin level following levothyroxine treatment [65]. Investigating the shared genetic liability between these two thyroid disorders, we observed a 45% genetic correlation. Most of the shared pathways are related to immune function, with the strongest common enrichment being MHC (major histocompatibility complex) class II proteins. Hashimoto’s thyroiditis, which has autoimmune etiology, is an example of a trait that, in some cases, is characterized by a sequential hyper- and hypothyroid states, consistent with this observation [66]. These findings confirm the key role of altered immune pathways in the pathogenesis of thyroid disorders [67]. Two pathways displayed a specific enrichment for hypothyroidism that is statistically different from that observed in hyperthyroidism: Janus kinase/signal transducers and activators of transcription (JAK/STAT) signaling and Rac GEF activity. Previous molecular studies found that the JAK/STAT signaling is involved in the transduction of a multitude of signals for development and homeostasis [68], also including certain TSH-related pathways [69]. On the basis of our results, we hypothesize that altered JAK/STAT signaling may lead to the onset of hypothyroidism. Altered Rac GEF activity was observed in multiple cancer types, including thyroid cancer [70], but no study previously investigated this pathway with respect to thyroid function. Given the role of Rac GEF in a number of physiological processes (e.g., embryonic development, immune responses, wound healing) [71], we hypothesize that germline variation associated with altered Rac GEF activity is also associated with hypothyroidism.

Testing the differences between the two thyroid disorders with respect to phenome-wide genetic correlations, we observed that hypothyroidism has a much stronger genetic correlation with taking prescription medications (*rg*, 49% vs. 16%). This suggests that hypothyroidism may be associated with more pathological conditions requiring taking prescription medications (beyond thyroid hormone replacement) than hyperthyroidism. There is also suggestive evidence of opposite effects of these thyroid disorders on physical activity: it is increased in hyperthyroidisms (*rg* = 0.18) and reduced in hypothyroidism (*rg* = −0.12). Although these results cannot be biased by environmental factors and genetic correlations cannot be used to infer causality, the consistency of these results with the known biological effects of thyroid hormone, suggests a causal relationship. Additional analyses will be needed to investigate the causal directions of the genetic correlations observed. A further limitation of our comparison analysis is due to the hyperthyroidism data, which were less informative than the hypothyroidism data. This is likely the reason why we were not able to detect any molecular pathway significantly altered in hyperthyroidism.

## 5. Conclusions

We provide novel data regarding the effects of the inter-individual variability in thyroid function within the reference range and of the molecular mechanisms involved in hypothyroidism and hyperthyroidism. To our knowledge, no previous study has reported similar information regarding thyroid biology. Future investigations of more informative datasets on the genetics of FT4 levels and hyperthyroidism will be able to provide additional findings regarding the biology of physiological and pathological conditions associated with thyroid function.

## Figures and Tables

**Table 1:** Significant causal associations based on thyroid stimulating hormone (TSH) genetic instrument that passed the sensitivity analyses.

Trait	Cases/Controls, *n*	Method	Estimate	SE	*p*
Hypothyroidism	16,376/320,783	Penalised median	0.020	0.0023	2.39 × 10^−17^
Non-toxic goiter	322/336,837	IVW fixed effects	−0.002	0.0001	9.45 × 10^−14^
Impedance of arm (left)	331,279	IVW fixed effects	−0.029	0.0017	2.22 × 10^−13^
Levothyroxine sodium use	13,717/323,442	IVW fixed effects	0.017	0.0008	2.41 × 10^−13^
Impedance of arm (right)	331,279	IVW fixed effects	−0.024	0.0018	9.60 × 10^−12^
Standing height	336,474	IVW fixed effects	−0.023	0.0019	2.76 × 10^−10^
Comparative height size at age 10	332,021	IVW fixed effects	−0.028	0.0026	1.74 × 10^−9^
Pulse rate, automated reading	317,756	IVW fixed effects	−0.037	0.0041	2.84 × 10^−8^
Thyroxine product use	3917/333,242	IVW fixed effects	0.005	0.0005	3.34 × 10^−8^
Impedance of whole body	331,284	IVW fixed effects	−0.022	0.0027	4.43 × 10^−8^
Hyperthyroidism	2,547/334,612	IVW fixed effects	−0.004	0.0004	7.49 × 10^−8^
Thyroid problem (not cancer)	887/336,272	IVW fixed effects	−0.001	0.0002	3.71 × 10^−6^
Female infertility	393/336,766	IVW random effects	0.001	0.0002	4.91 × 10^−6^
Hip circumference	336,601	IVW fixed effects	−0.022	0.0039	1.51 × 10^−5^
Trunk fat percentage	331,113	Egger fixed effects	−0.048	0.0087	2.23 × 10^−5^
Hearing aid use	10,322/193,918	Rucker median (JK)	−0.005	0.0010	7.10 × 10^−5^

SE: Standard Error; IVW: Inverse Variance Weighted; JK: Jackknife.

**Table 2:** Genetics correlations surviving Bonferroni multiple-testing correction (*p* < 3.17 × 10^−5^) in hypothyroidism analysis.

Trait	Hyperthyroidism		Hypothyroidism		Difference	
	*rg*	*p*	*rg*	*p*	*z*	*p*
Levothyroxine sodium use	0.49	5.72 × 10^−7^	1.00	<4.50 × 10^−308^	−5.24	1.64 × 10^−7^
Thyroxine product use	0.51	0.002	1.03	2.15 × 10^−52^	−2.96	0.003
Taking other prescription medications	0.16	0.015	0.49	5.09 × 10^−52^	−4.38	1.18 × 10^−5^
Other serious medical condition/disability diagnosed by doctor	0.32	8.65 × 10^−5^	0.46	7.43 × 10^−38^	−1.54	0.122
Number of self-reported non-cancer illnesses	0.17	0.008	0.36	2.53 × 10^−36^	−2.78	0.005
Long-standing illness, disability or infirmity	0.14	0.026	0.36	8.63 × 10^−30^	−3.21	0.001
Number of treatments/medications taken	0.18	0.002	0.32	1.43×10^−22^	−2.23	0.026
Frequency of tiredness/lethargy in last 2 weeks	0.07	0.278	0.24	1.25 × 10^−14^	−2.31	0.021
Chest pain or discomfort	0.19	0.014	0.24	2.58 × 10^−11^	−0.58	0.561
Waist circumference	0.04	0.381	0.17	6.94 × 10^−11^	−2.42	0.015
Overall health rating	0.07	0.166	0.21	8.86 × 10^−10^	−2.36	0.018
Trunk fat percentage	0.01	0.771	0.14	1.94 × 10^−8^	−2.42	0.016
Illness, injury, bereavement, stress in last 2 years: Serious illness, injury or assault to yourself	0.11	0.345	0.30	1.99 × 10^−8^	−1.52	0.129
Trunk fat mass	0.03	0.516	0.14	3.00 × 10^−8^	−2.20	0.028
Body fat percentage	0.01	0.841	0.14	3.28 × 10^−8^	−2.54	0.011
Whole body fat mass	0.02	0.653	0.15	9.32 × 10^−8^	−2.39	0.017
Seen doctor for nerves, anxiety, tension or depression	0.01	0.896	0.15	1.24 × 10^−7^	−2.21	0.027
Medication for pain relief, constipation, heartburn	−0.08	0.173	−0.16	1.41 × 10^−7^	1.19	0.235
Illness, injury, bereavement, stress in last 2 years	0.06	0.488	−0.21	1.42 × 10^−7^	2.89	0.004
Arm fat percentage (right)	0.01	0.737	0.14	1.81 × 10^−7^	−2.44	0.015
Leg fat percentage (left)	0.01	0.904	0.14	2.68 × 10^−7^	−2.49	0.013
Arm fat percentage (left)	0.01	0.770	0.14	4.23 × 10^−7^	−2.40	0.016
Leg fat mass (left)	0.02	0.744	0.15	4.90 × 10^−7^	−2.41	0.016
Arm fat mass (right)	0.02	0.619	0.15	5.56 × 10^−7^	−2.35	0.019
Pain type(s) experienced in last month: Neck or shoulder pain	0.11	0.116	0.17	6.22 × 10^−7^	−0.68	0.495
Insulin product use	0.15	0.337	0.42	6.26 × 10^−7^	−1.59	0.112
Arm fat mass (left)	0.03	0.548	0.15	7.97 × 10^−7^	−2.19	0.029
Leg fat percentage (right)	0.00	0.970	0.13	8.15 × 10^−7^	−2.53	0.011
Leg fat mass (right)	0.01	0.794	0.14	8.55 × 10^−7^	−2.43	0.015
Illness, injury, bereavement, stress in last 2 years: Financial difficulties	0.06	0.447	0.19	8.82 × 10^−7^	−1.50	0.134
Pain in throat and chest	−0.13	0.228	0.23	1.10 × 10^−6^	−3.06	0.002
Paracetamol use	0.12	0.108	0.18	1.21 × 10^−6^	−0.71	0.477
Falls in the last year	0.02	0.824	0.18	1.34 × 10^−6^	−1.97	0.049
Loneliness, isolation	0.11	0.128	0.19	1.82 × 10^−6^	−0.93	0.352
Weight	0.04	0.413	0.13	2.18 × 10^−6^	−1.74	0.082
Frequency of depressed mood in last 2 weeks	0.05	0.508	0.19	2.76 × 10^−6^	−1.68	0.094
Physical activity in last 4 weeks	0.03	0.768	0.22	3.58 × 10^−6^	−1.97	0.049
Pain type(s) experienced in last month	−0.13	0.024	−0.14	5.19 × 10^−6^	0.19	0.851
Pain type(s) experienced in last month: Stomach or abdominal pain	0.08	0.426	0.20	5.36 × 10^−6^	−1.17	0.243
Hip circumference	0.03	0.480	0.13	6.31 × 10^−6^	−1.73	0.084
Medication for pain relief, constipation, heartburn: Paracetamol	0.14	0.050	0.16	7.60 × 10^−6^	−0.24	0.812
Illnesses of siblings: Heart disease	0.27	0.008	0.22	8.44 × 10^−6^	0.40	0.693
Alcohol intake frequency	−0.02	0.719	0.12	8.46 × 10^−6^	−2.16	0.031
Body mass index	0.01	0.816	0.14	9.77 × 10^−6^	−2.37	0.018
Shortness of breath walking on level ground	−0.02	0.841	0.23	1.05 × 10^−5^	−2.23	0.026
Angina pectoris	0.08	0.571	0.31	1.33 × 10^−5^	−1.52	0.129
Illnesses of mother: Heart disease	0.14	0.172	0.24	1.33 × 10^−5^	−0.89	0.374
Types of physical activity in last 4 weeks: Other exercises (e.g., swimming, cycling, keep fit, bowling)	0.02	0.760	−0.15	1.42 × 10^−5^	2.35	0.019
Frequency of unenthusiasm/disinterest in last 2 weeks	0.01	0.865	0.18	1.57 × 10^−5^	−1.92	0.055
Diabetes diagnosed by doctor	0.12	0.104	0.18	1.61 × 10^−5^	−0.76	0.450
Fed-up feelings	−0.06	0.417	0.18	1.66 × 10^−5^	−2.92	0.004
Cholesterol lowering medication use	0.11	0.198	0.19	2.05 × 10^−5^	−0.81	0.418
Diabetes	0.12	0.116	0.18	2.35 × 10^−5^	−0.74	0.462
Health satisfaction	0.10	0.186	0.21	2.46 × 10^−5^	−1.23	0.219
Sensitivity/hurt feelings	0.01	0.834	0.16	2.67 × 10^−5^	−2.00	0.045
Mood swings	−0.03	0.585	0.16	2.80 × 10^−5^	−2.68	0.007
Insulin use	0.28	0.089	0.41	2.99 × 10^−5^	−0.67	0.506
Wheeze or whistling in the chest in last year	0.11	0.085	0.17	3.00 × 10^−5^	−0.89	0.372

**Table 3:** Enrichments surviving Bonferroni multiple-testing correction (*p* < 4.59 × 10^−6^) in both hyperthyroidism and hypothyroidism analyses.

Enrichment	Genes, *n*	Hyperthyroidism		Hypothyroidism	
		*β*	*p*	*β*	*p*
GO~MHC Class II Protein Complex	14	3.33	4.58 × 10^−30^	3.18	2.08 × 10^−18^
KEGG~Autoimmune Thyroid Disease	49	1.5	4.41 × 10^−21^	1.9	6.00 × 10^−22^
GO~MHC Protein Complex	24	1.66	3.42 × 10^−19^	1.32	7.16 × 10^−9^
GO~MHC Class II Receptor Activity	10	2.34	3.60 × 10^−17^	2.06	1.91 × 10^−9^
KEGG~Allograft Rejection	34	1.36	9.42 × 10^−16^	1.6	2.06 × 10^−14^
KEGG~Type I Diabetes Mellitus	41	1.09	2.29 × 10^−14^	1.08	9.20 × 10^−10^
KEGG~Asthma	27	1.27	2.51 × 10^−14^	1.32	1.32 × 10^−10^
KEGG~Graft Versus Host Disease	37	1.03	8.75 × 10^−12^	1.21	1.44 × 10^−10^
KEGG~Intestinal Immune Network for IgA Production	43	0.989	3.31 × 10^−11^	1.16	3.34 × 10^−10^
GO~Regulation of Immune Response	780	0.171	7.93 × 10^−8^	0.316	3.53 × 10^−15^
GO~Positive Regulation of Cell Activation	275	0.262	8.44 × 10^−7^	0.539	1.32 × 10^−15^
KEGG~Leishmania Infection	66	0.559	9.05 × 10^−7^	0.656	3.34 × 10^−6^
GO~Positive Regulation of Immune Response	505	0.187	1.02 × 10^−6^	0.342	1.44 × 10^−12^
KEGG~Antigen Processing and Presentation	82	0.512	1.76 × 10^−6^	0.675	4.44 × 10^−7^
GO~Interferon Gamma Mediated Signaling Pathway	66	0.53	1.96 × 10^−6^	0.863	7.38 × 10^−10^
GO~Peptide Antigen Binding	26	0.861	2.01 × 10^−6^	1.16	2.89 × 10^−7^
GO~Positive Regulation of Cell Cell Adhesion	229	0.283	2.02 × 10^−6^	0.475	2.71 × 10^−10^
GO~Lumenal Side of Membrane	28	0.8	4.23 × 10^−6^	1.29	3.47 × 10^−9^

## Supplementary Materials

The following are available online at http://www.mdpi.com/2077-0383/7/10/296/s1, Table S1: Variants included in the genetic instruments used to investigate TSH and FT4. Table S2: List of GWAS summary association data regarding 2419 traits used in the study. Table S3: List of the MR methods used in the study. Table S4: Significant causal associations identified using TSH genetic instrument (FDR *q* < 0.05). Table S5: Significant causal associations identified using TSH genetic instrument with the corresponding sensitivity analyses. Table S6: Genomic risk loci and independent variants (LD *r*^2^ < 0.1) identified in hyperthyroidism GWAS. Table S7: Genomic risk loci and independent variants (LD *r*^2^ < 0.1) identified in hypothyroidism GWAS. Table S8: Genetic correlation of hyperthyroidism and hypothyroidism calculated with respect to 1578 phenotypic traits. Table S9: Results from enrichment analysis conducted with respect to hyperthyroidism and hypothyroidism.

## References

[1] Ravera S., Reyna-Neyra A., Ferrandino G., Amzel L.M., Carrasco N. (2017). The sodium/iodide symporter (NIS): Molecular physiology and preclinical and clinical applications. Annu. Rev. Physiol..

[2] Taylor P.N., Albrecht D., Scholz A., Gutierrez-Buey G., Lazarus J.H., Dayan C.M., Okosieme O.E. (2018). Global epidemiology of hyperthyroidism and hypothyroidism. Nat. Rev. Endocrinol..

[3] Gencer B., Collet T.H., Virgini V., Bauer D.C., Gussekloo J., Cappola A.R., Nanchen D., den Elzen W.P., Balmer P., Luben R.N. (2012). Subclinical thyroid dysfunction and the risk of heart failure events: An individual participant data analysis from 6 prospective cohorts. Circulation.

[4] Rodondi N., den Elzen W.P., Bauer D.C., Cappola A.R., Razvi S., Walsh J.P., Asvold B.O., Iervasi G., Imaizumi M., Collet T.H. (2010). Subclinical hypothyroidism and the risk of coronary heart disease and mortality. JAMA.

[5] Vadiveloo T., Donnan P.T., Cochrane L., Leese G.P. (2011). The thyroid epidemiology, audit, and research study (TEARS): Morbidity in patients with endogenous subclinical hyperthyroidism. J. Clin. Endocrinol. Metab..

[6] Cappola A.R., Arnold A.M., Wulczyn K., Carlson M., Robbins J., Psaty B.M. (2015). Thyroid function in the euthyroid range and adverse outcomes in older adults. J. Clin. Endocrinol. Metab..

[7] Taylor P.N., Razvi S., Pearce S.H., Dayan C.M. (2013). Clinical review: A review of the clinical consequences of variation in thyroid function within the reference range. J. Clin. Endocrinol. Metab..

[8] Medici M., Visser T.J., Peeters R.P. (2017). Genetics of thyroid function. Best Pract. Res. Clin. Endocrinol. Metab..

[9] Porcu E., Medici M., Pistis G., Volpato C.B., Wilson S.G., Cappola A.R., Bos S.D., Deelen J., den Heijer M., Freathy R.M. (2013). A meta-analysis of thyroid-related traits reveals novel loci and gender-specific differences in the regulation of thyroid function. PLoS Genet..

[10] Taylor P.N., Porcu E., Chew S., Campbell P.J., Traglia M., Brown S.J., Mullin B.H., Shihab H.A., Min J., Walter K. (2015). Whole-genome sequence-based analysis of thyroid function. Nat. Commun..

[11] Nielsen T.R., Appel E.V., Svendstrup M., Ohrt J.D., Dahl M., Fonvig C.E., Hollensted M., Have C.T., Kadarmideen H.N., Pedersen O. (2017). A genome-wide association study of thyroid stimulating hormone and free thyroxine in danish children and adolescents. PLoS ONE.

[12] Burgess S., Scott R.A., Timpson N.J., Davey Smith G., Thompson S.G., Consortium E.-I. (2015). Using published data in mendelian randomization: A blueprint for efficient identification of causal risk factors. Eur. J. Epidemiol..

[13] Emdin C.A., Khera A.V., Natarajan P., Klarin D., Zekavat S.M., Hsiao A.J., Kathiresan S. (2017). Genetic association of waist-to-hip ratio with cardiometabolic traits, type 2 diabetes, and coronary heart disease. JAMA.

[14] Polimanti R., Amstadter A.B., Stein M.B., Almli L.M., Baker D.G., Bierut L.J., Bradley B., Farrer L.A., Johnson E.O., King A. (2017). A putative causal relationship between genetically determined female body shape and posttraumatic stress disorder. Genome Med..

[15] Polimanti R., Gelernter J., Stein D.J. (2017). Genetically determined schizophrenia is not associated with impaired glucose homeostasis. Schizophr. Res..

[16] Polimanti R., Peterson R.E., Ong J.S., Macgregor S., Edwards A., Clarke T.-K., Frank J., Gerring Z., Gillespie N.A., Lind P.A. (2018). Evidence of causal effect of major depression on alcohol dependence: Findings from the psychiatric genomics consortium. bioRxiv.

[17] Maffini M.V., Trasande L., Neltner T.G. (2016). Perchlorate and diet: Human exposures, risks, and mitigation strategies. Curr. Environ. Health Rep..

[18] Palkowska-Gozdzik E., Lachowicz K., Rosolowska-Huszcz D. (2017). Effects of dietary protein on thyroid axis activity. Nutrients.

[19] Shukla S.K., Singh G., Ahmad S., Pant P. (2018). Infections, genetic and environmental factors in pathogenesis of autoimmune thyroid diseases. Microb. Pathog..

[20] Bos M.M., Smit R.A.J., Trompet S., van Heemst D., Noordam R. (2017). Thyroid signaling, insulin resistance, and 2 diabetes mellitus: A mendelian randomization study. J. Clin. Endocrinol. Metab..

[21] Van Vliet N.A., Noordam R., Van Klinken J.B., Westendorp R.G.J., Bassett J.H.D., Williams G.R., Van Heemst D. (2018). Thyroid stimulating hormone and bone mineral density: Evidence from a two-sample mendelian randomization study and a candidate gene association study. J. Bone Miner. Res..

[22] Zhao J.V., Schooling C.M. (2017). Thyroid function and ischemic heart disease: A mendelian randomization study. Sci. Rep..

[23] Polimanti R., Jensen K.P., Gelernter J. (2017). Phenome-wide association study for CYP2A6 alleles: Rs113288603 is associated with hearing loss symptoms in elderly smokers. Sci. Rep..

[24] Karaca S., Civelek E., Karaca M., Sahiner U.M., Ozgul R.K., Kocabas C.N., Polimanti R., Sekerel B.E. (2016). Allergy-specific phenome-wide association study for immunogenes in turkish children. Sci. Rep..

[25] Polimanti R., Kranzler H.R., Gelernter J. (2016). Phenome-wide association study for alcohol and nicotine risk alleles in 26394 women. Neuropsychopharmacology.

[26] Allen N.E., Sudlow C., Peakman T., Collins R., Biobank U.K. (2014). Uk biobank data: Come and get it. Sci. Transl. Med..

[27] Bowden J., Del Greco M.F., Minelli C., Davey Smith G., Sheehan N., Thompson J. (2017). A framework for the investigation of pleiotropy in two-sample summary data mendelian randomization. Stat. Med..

[28] Bowden J., Davey Smith G., Haycock P.C., Burgess S. (2016). Consistent estimation in mendelian randomization with some invalid instruments using a weighted median estimator. Genet. Epidemiol..

[29] Hartwig F.P., Davey Smith G., Bowden J. (2017). Robust inference in summary data mendelian randomization via the zero modal pleiotropy assumption. Int. J. Epidemiol..

[30] Rucker G., Schwarzer G., Carpenter J.R., Binder H., Schumacher M. (2011). Treatment-effect estimates adjusted for small-study effects via a limit meta-analysis. Biostatistics.

[31] Hemani G., Tilling K., Davey Smith G. (2017). Orienting the causal relationship between imprecisely measured traits using gwas summary data. PLoS Genet..

[32] Bowden J., Davey Smith G., Burgess S. (2015). Mendelian randomization with invalid instruments: Effect estimation and bias detection through egger regression. Int. J. Epidemiol..

[33] Hemani G., Zheng J., Wade K.H., Laurin C., Elsworth B., Burgess S., Bowden J., Langdon R., Tan V., Yarmolinsky J. (2016). Mr-base: A platform for systematic causal inference across the phenome using billions of genetic associations. bioRxiv.

[34] Bulik-Sullivan B., Finucane H.K., Anttila V., Gusev A., Day F.R., Loh P.R., ReproGen C., Duncan L., Perry J.R.B., Patterson N. (2015). An atlas of genetic correlations across human diseases and traits. Nat. Genet..

[35] De Leeuw C.A., Mooij J.M., Heskes T., Posthuma D. (2015). Magma: Generalized gene-set analysis of gwas data. PLoS Comput. Biol..

[36] Watanabe K., Taskesen E., van Bochoven A., Posthuma D. (2017). Functional mapping and annotation of genetic associations with fuma. Nat. Commun..

[37] Furqan S., Haque N.U., Islam N. (2014). Conversion of autoimmune hypothyroidism to hyperthyroidism. BMC Res. Notes.

[38] Stanford S.M., Bottini N. (2014). Ptpn22: The archetypal non-hla autoimmunity gene. Nat. Rev. Rheumatol..

[39] Hradsky O., Dusatkova P., Lenicek M., Bronsky J., Nevoral J., Vitek L., Lukas M., Zeniskova I., Cinek O. (2010). The CTLA4 variants may interact with the IL23R- and NOD2-conferred risk in development of Crohn’s disease. BMC Med. Genet..

[40] Kanehisa M., Furumichi M., Tanabe M., Sato Y., Morishima K. (2017). KEGG: New perspectives on genomes, pathways, diseases and drugs. Nucleic Acids Res..

[41] The Gene Ontology Consortium (2017). Expansion of the gene ontology knowledgebase and resources. Nucleic Acids Res..

[42] Asvold B.O., Vatten L.J., Midthjell K., Bjoro T. (2012). Serum TSH within the reference range as a predictor of future hypothyroidism and hyperthyroidism: 11-Year Follow-Up of the hunt study in norway. J. Clin. Endocrinol. Metab..

[43] Denny J.C., Crawford D.C., Ritchie M.D., Bielinski S.J., Basford M.A., Bradford Y., Chai H.S., Bastarache L., Zuvich R., Peissig P. (2011). Variants near foxe1 are associated with hypothyroidism and other thyroid conditions: Using electronic medical records for genome- and phenome-wide studies. Am. J. Hum. Genet..

[44] Benyi E., Savendahl L. (2017). The physiology of childhood growth: Hormonal regulation. Horm. Res. Paediatr..

[45] Robson H., Siebler T., Stevens D.A., Shalet S.M., Williams G.R. (2000). Thyroid hormone acts directly on growth plate chondrocytes to promote hypertrophic differentiation and inhibit clonal expansion and cell proliferation. Endocrinology.

[46] Williams G.R. (2013). Thyroid hormone actions in cartilage and bone. Eur. Thyroid J..

[47] Brufani C., Manco M., Nobili V., Fintini D., Barbetti F., Cappa M. (2012). Thyroid function tests in obese prepubertal children: Correlations with insulin sensitivity and body fat distribution. Horm. Res. Paediatr..

[48] Pearce E.N. (2012). Thyroid hormone and obesity. Curr. Opin. Endocrinol. Diabetes Obes..

[49] Ren R., Jiang X., Zhang X., Guan Q., Yu C., Li Y., Gao L., Zhang H., Zhao J. (2014). Association between thyroid hormones and body fat in euthyroid subjects. Clin. Endocrinol..

[50] Santini F., Marzullo P., Rotondi M., Ceccarini G., Pagano L., Ippolito S., Chiovato L., Biondi B. (2014). Mechanisms in endocrinology: The crosstalk between thyroid gland and adipose tissue: Signal integration in health and disease. Eur. J. Endocrinol..

[51] Chen Y., Chen Y., Wang N., Chen C., Nie X., Li Q., Han B., Lu Y. (2018). Thyroid stimulating hormone within the reference range is associated with visceral adiposity index and lipid accumulation product: A population-based study of spect-China. Horm. Metab. Res..

[52] Witte T., Volzke H., Lerch M.M., Hegenscheid K., Friedrich N., Ittermann T., Batsis J.A. (2017). Association between serum thyroid-stimulating hormone levels and visceral adipose tissue: A population-based study in northeast Germany. Eur. Thyroid J..

[53] Lee J.J., Pedley A., Marqusee E., Sutherland P., Hoffmann U., Massaro J.M., Fox C.S. (2016). Thyroid function and cardiovascular disease risk factors in euthyroid adults: A cross-sectional and longitudinal study. Clin. Endocrinol..

[54] Grais I.M., Sowers J.R. (2014). Thyroid and the heart. Am. J. Med..

[55] Baumgartner C., da Costa B.R., Collet T.H., Feller M., Floriani C., Bauer D.C., Cappola A.R., Heckbert S.R., Ceresini G., Gussekloo J. (2017). Thyroid function within the normal range, subclinical hypothyroidism, and the risk of atrial fibrillation. Circulation.

[56] De Groot L., Abalovich M., Alexander E.K., Amino N., Barbour L., Cobin R.H., Eastman C.J., Lazarus J.H., Luton D., Mandel S.J. (2012). Management of thyroid dysfunction during pregnancy and postpartum: An endocrine society clinical practice guideline. J. Clin. Endocrinol. Metab..

[57] Kuroda K., Uchida T., Nagai S., Ozaki R., Yamaguchi T., Sato Y., Brosens J.J., Takeda S. (2015). Elevated serum thyroid-stimulating hormone is associated with decreased anti-mullerian hormone in infertile women of reproductive age. J. Assist. Reprod. Genet..

[58] Poppe K., Velkeniers B., Glinoer D. (2007). Thyroid disease and female reproduction. Clin. Endocrinol..

[59] Karmon A.E., Batsis M., Chavarro J.E., Souter I. (2015). Preconceptional thyroid-stimulating hormone levels and outcomes of intrauterine insemination among euthyroid infertile women. Fertil. Steril..

[60] Glendenning P. (2008). Management of thyroid dysfunction during pregnancy and postpartum: An endocrine society clinical practice guideline. Clin. Biochem. Rev..

[61] Garber J.R., Cobin R.H., Gharib H., Hennessey J.V., Klein I., Mechanick J.I., Pessah-Pollack R., Singer P.A., Woeber K.A., American Association of Clinical Endocrinologists (2012). Clinical practice guidelines for hypothyroidism in adults: Cosponsored by the american association of clinical endocrinologists and the american thyroid association. Endocr. Pract..

[62] Berker D., Karabulut H., Isik S., Tutuncu Y., Ozuguz U., Erden G., Aydin Y., Dagli M., Guler S. (2012). Evaluation of hearing loss in patients with graves’ disease. Endocrine.

[63] Arduc A., Isik S., Allusoglu S., Iriz A., Dogan B.A., Gocer C., Tuna M.M., Berker D., Guler S. (2015). Evaluation of hearing functions in patients with euthyroid hashimoto’s thyroiditis. Endocrine.

[64] Sharma K., Behera J.K., Kumar N., Sood S., Madan H.S., Das S. (2015). Brainstem evoked potential in newly diagnosed patients of subclinical hypothyroidism. N. Am. J. Med. Sci..

[65] Taylor P.N., Iqbal A., Minassian C., Sayers A., Draman M.S., Greenwood R., Hamilton W., Okosieme O., Panicker V., Thomas S.L. (2014). Falling threshold for treatment of borderline elevated thyrotropin levels-balancing benefits and risks: Evidence from a large community-based study. JAMA Intern. Med..

[66] Antonelli A., Ferrari S.M., Corrado A., Di Domenicantonio A., Fallahi P. (2015). Autoimmune thyroid disorders. Autoimmun. Rev..

[67] Lee H.J., Li C.W., Hammerstad S.S., Stefan M., Tomer Y. (2015). Immunogenetics of autoimmune thyroid diseases: A comprehensive review. J. Autoimmun..

[68] Rawlings J.S., Rosler K.M., Harrison D.A. (2004). The JAK/STAT signaling pathway. J. Cell Sci..

[69] Park E.S., Kim H., Suh J.M., Park S.J., You S.H., Chung H.K., Lee K.W., Kwon O.Y., Cho B.Y., Kim Y.K. (2000). Involvement of JAK/STAT (Janus kinase/signal transducer and activator of transcription) in the thyrotropin signaling pathway. Mol. Endocrinol..

[70] Welch H.C. (2015). Regulation and function of P-rex family Rac-GEFs. Small GTPases.

[71] Marei H., Malliri A. (2017). Gefs: Dual regulation of Rac1 signaling. Small GTPases.

