# Supplementary material for: Phenomic Impact of Genetically-Determined Euthyroid Function and Molecular Differences between Thyroid Disorders
Source: J Clin Med. 2018 Sep 21;7(10):296. doi: 10.3390/jcm7100296 (PMC6210201; doi:10.3390/jcm7100296)
Supplement: Supplementary file 1 [file jcm-07-00296-s001.pdf]

Table S1. Variants included in the genetic instruments used to investigate TSH and FT4.

Phenotype	SNP	effect_allele	other_allele	eaf	*β*	SE	*p*	*n*	Reference
TSH	rs6885099	A	G	0.594	-0.141	0.009	1.95E-56	26,042	9
	rs28435578	C	T	0.227	-0.166	0.014	4.59E-32	16,333	10
	rs3008034	C	T	0.312	-0.131	0.012	4.68E-26	16,335	10
	rs10799824	A	G	0.161	-0.113	0.012	3.60E-21	26,031	9
	rs17767742	G	C	0.354	-0.113	0.012	5.64E-20	16,335	10
	rs112817873	T	A	0.323	-0.14	0.015	6.15E-20	11,544	10
	rs7568039	A	C	0.25	-0.122	0.014	2.11E-19	16,335	10
	rs9472138	T	C	0.285	-0.079	0.01	6.72E-16	25,767	9
	rs6923866	C	T	0.28	-0.102	0.013	7.55E-15	16,333	10
	rs2928167	G	A	0.104	-0.145	0.019	5.94E-14	16,334	10
	rs9915657	T	C	0.541	-0.064	0.009	7.53E-13	25,692	9
	rs334699	A	G	0.052	-0.141	0.021	5.40E-12	25,757	9
	rs10519227	A	T	0.245	-0.072	0.011	1.02E-11	25,988	9
	rs116909374	T	C	0.043	-0.208	0.032	4.69E-11	15,037	10
	rs17723470	T	C	0.279	-0.065	0.01	8.83E-11	26,054	9
	rs17776563	A	G	0.322	-0.06	0.01	2.89E-10	25,758	9
	rs4804416	T	G	0.569	-0.057	0.009	3.16E-10	25,632	9
	rs657152	A	C	0.343	0.058	0.009	4.11E-10	25,765	9
	rs11624776	A	C	0.66	-0.064	0.011	1.79E-09	23,482	9
	rs7825175	A	G	0.21	-0.066	0.011	2.94E-09	25,996	9
	rs310763	T	C	0.235	0.083	0.014	6.15E-09	16,334	10
	rs1537424	T	C	0.608	-0.052	0.009	1.17E-08	25,478	9
	rs9497965	T	C	0.415	0.051	0.009	2.25E-08	25,980	9
	rs1571583	A	G	0.249	0.057	0.01	2.55E-08	25,766	9
FT4	rs2235544	A	C	0.499	0.154	0.013	5.23E-34	13,650	10
	rs7860634	A	G	0.53	0.102	0.013	2.30E-14	14,529	9
	rs7045138	T	C	0.553	0.098	0.015	1.50E-11	10,997	9
	rs7694879	T	C	0.095	0.137	0.022	4.15E-10	13,650	10
	rs113107469	T	C	0.032	0.223	0.037	1.27E-09	13,649	10

Table S2: List of GWAS summary association data regarding 2419 traits used in the study.

UKB code	UKB Description	Analyses Conducted
46	Hand grip strength (left)	MR, LDSC
47	Hand grip strength (right)	MR, LDSC
48	Waist circumference	MR, LDSC
49	Hip circumference	MR, LDSC
50	Standing height	MR, LDSC
78	Heel bone mineral density (BMD) T-score, automated	MR, LDSC
102	Pulse rate, automated reading	MR, LDSC
134	Number of self-reported cancers	MR, LDSC
135	Number of self-reported non-cancer illnesses	MR, LDSC
136	Number of operations, self-reported	MR, LDSC
137	Number of treatments/medications taken	MR, LDSC
189	Townsend deprivation index at recruitment	MR, LDSC
399	Number of incorrect matches in round	MR, LDSC
757	Time employed in main current job	MR, LDSC
767	Length of working week for main job	MR, LDSC
777	Frequency of travelling from home to job workplace	MR, LDSC
796	Distance between home and job workplace	MR, LDSC
806	Job involves mainly walking or standing	MR, LDSC
816	Job involves heavy manual or physical work	MR, LDSC
826	Job involves shift work	MR, LDSC
845	Age completed full time education	MR, LDSC
864	Number of days/week walked 10+ minutes	MR, LDSC
874	Duration of walks	MR, LDSC
884	Number of days/week of moderate physical activity 10+ minutes	MR, LDSC
894	Duration of moderate activity	MR, LDSC
904	Number of days/week of vigorous physical activity 10+ minutes	MR, LDSC
914	Duration of vigorous activity	MR, LDSC

924	Usual walking pace	MR, LDSC
943	Frequency of stair climbing in last 4 weeks	MR, LDSC
971	Frequency of walking for pleasure in last 4 weeks	MR, LDSC
981	Duration walking for pleasure	MR, LDSC
991	Frequency of strenuous sports in last 4 weeks	MR, LDSC
1001	Duration of strenuous sports	MR, LDSC
1011	Frequency of light DIY in last 4 weeks	MR, LDSC
1021	Duration of light DIY	MR, LDSC
1070	Time spent watching television (TV)	MR, LDSC
1080	Time spent using computer	MR, LDSC
1090	Time spent driving	MR, LDSC
1100	Drive faster than motorway speed limit	MR, LDSC
1160	Sleep duration	MR, LDSC
1170	Getting up in morning	MR, LDSC
1180	Morning/evening person (chronotype)	MR, LDSC
1190	Nap during day	MR, LDSC
1200	Sleeplessness / insomnia	MR, LDSC
1210	Snoring	MR, LDSC
1220	Daytime dozing / sleeping (narcolepsy)	MR, LDSC
1239	Current tobacco smoking	MR, LDSC
1249	Past tobacco smoking	MR, LDSC
1259	Smoking/smokers in household	MR, LDSC
1269	Exposure to tobacco smoke at home	MR, LDSC
1279	Exposure to tobacco smoke outside home	MR, LDSC
1558	Alcohol intake frequency.	MR, LDSC
1568	Average weekly red wine intake	MR, LDSC
1578	Average weekly champagne plus white wine intake	MR, LDSC
1588	Average weekly beer plus cider intake	MR, LDSC
1598	Average weekly spirits intake	MR, LDSC
1608	Average weekly fortified wine intake	MR, LDSC

1618	Alcohol usually taken with meals	MR, LDSC
1628	Alcohol intake versus 10 years previously	MR, LDSC
1677	Breastfed as a baby	MR, LDSC
1687	Comparative body size at age 10	MR, LDSC
1697	Comparative height size at age 10	MR, LDSC
1767	Adopted as a child	MR, LDSC
1777	Part of a multiple birth	MR, LDSC
1787	Maternal smoking around birth	MR, LDSC
1797	Father still alive	MR, LDSC
1807	Father's age at death	MR, LDSC
1835	Mother still alive	MR, LDSC
1873	Number of full brothers	MR, LDSC
1883	Number of full sisters	MR, LDSC
1920	Mood swings	MR, LDSC
1930	Miserableness	MR, LDSC
1940	Irritability	MR, LDSC
1950	Sensitivity / hurt feelings	MR, LDSC
1960	Fed-up feelings	MR, LDSC
1970	Nervous feelings	MR, LDSC
1980	Worrier / anxious feelings	MR, LDSC
1990	Tense / 'highly strung'	MR, LDSC
2000	Worry too long after embarrassment	MR, LDSC
2010	Suffer from 'nerves'	MR, LDSC
2020	Loneliness, isolation	MR, LDSC
2030	Guilty feelings	MR, LDSC
2040	Risk taking	MR, LDSC
2050	Frequency of depressed mood in last 2 weeks	MR, LDSC
2060	Frequency of unenthusiasm / disinterest in last 2 weeks	MR, LDSC
2070	Frequency of tenseness / restlessness in last 2 weeks	MR, LDSC
2080	Frequency of tiredness / lethargy in last 2 weeks	MR, LDSC

2090	Seen doctor (GP) for nerves, anxiety, tension or depression	MR, LDSC
2100	Seen a psychiatrist for nerves, anxiety, tension or depression	MR, LDSC
2178	Overall health rating	MR, LDSC
2188	Long-standing illness, disability or infirmity	MR, LDSC
2207	Wears glasses or contact lenses	MR, LDSC
2227	Other eye problems	MR, LDSC
2257	Hearing difficulty/problems with background noise	MR, LDSC
2296	Falls in the last year	MR, LDSC
2306	Weight change compared with 1 year ago	MR, LDSC
2316	Wheeze or whistling in the chest in last year	MR, LDSC
2335	Chest pain or discomfort	MR, LDSC
2345	Ever had bowel cancer screening	MR, LDSC
2355	Most recent bowel cancer screening	MR, LDSC
2365	Ever had prostate specific antigen (PSA) test	MR, LDSC
2375	Relative age of first facial hair	MR, LDSC
2385	Relative age voice broke	MR, LDSC
2405	Number of children fathered	MR, LDSC
2415	Had major operations	MR, LDSC
2443	Diabetes diagnosed by doctor	MR, LDSC
2453	Cancer diagnosed by doctor	MR, LDSC
2463	Fractured/broken bones in last 5 years	MR, LDSC
2473	Other serious medical condition/disability diagnosed by doctor	MR, LDSC
2492	Taking other prescription medications	MR, LDSC
2624	Frequency of heavy DIY in last 4 weeks	MR, LDSC
2634	Duration of heavy DIY	MR, LDSC
2644	Light smokers, at least 100 smokes in lifetime	MR, LDSC
2674	Ever had breast cancer screening / mammogram	MR, LDSC
2684	Years since last breast cancer screening / mammogram	MR, LDSC
2694	Ever had cervical smear test	MR, LDSC
2704	Years since last cervical smear test	MR, LDSC

2714	Age when periods started (menarche)	MR, LDSC
2724	Had menopause	MR, LDSC
2734	Number of live births	MR, LDSC
2744	Birth weight of first child	MR, LDSC
2754	Age at first live birth	MR, LDSC
2764	Age at last live birth	MR, LDSC
2774	Ever had stillbirth, spontaneous miscarriage or termination	MR, LDSC
2784	Ever taken oral contraceptive pill	MR, LDSC
2794	Age started oral contraceptive pill	MR, LDSC
2814	Ever used hormone-replacement therapy (HRT)	MR, LDSC
2834	Bilateral oophorectomy (both ovaries removed)	MR, LDSC
2844	Had other major operations	MR, LDSC
2887	Number of cigarettes previously smoked daily	MR, LDSC
2907	Ever stopped smoking for 6+ months	MR, LDSC
2926	Number of unsuccessful stop-smoking attempts	MR, LDSC
2936	Likelihood of resuming smoking	MR, LDSC
2956	General pain for 3+ months	MR
2986	Started insulin within one year diagnosis of diabetes	MR, LDSC
3005	Fracture resulting from simple fall	MR, LDSC
3062	Forced vital capacity (FVC)	MR, LDSC
3063	Forced expiratory volume in 1-second (FEV1)	MR, LDSC
3064	Peak expiratory flow (PEF)	MR, LDSC
3079	Pace-maker	MR, LDSC
3090	Used an inhaler for chest within last hour	MR, LDSC
3159	Smoked cigarette or pipe within last hour	MR, LDSC
3393	Hearing aid user	MR, LDSC
3404	Neck/shoulder pain for 3+ months	MR, LDSC
3414	Hip pain for 3+ months	MR, LDSC
3456	Number of cigarettes currently smoked daily (current cigarette smokers)	MR, LDSC
3466	Time from waking to first cigarette	MR, LDSC

3476	Difficulty not smoking for 1 day	MR, LDSC
3486	Ever tried to stop smoking	MR, LDSC
3496	Wants to stop smoking	MR, LDSC
3526	Mother's age at death	MR, LDSC
3571	Back pain for 3+ months	MR, LDSC
3591	Ever had hysterectomy (womb removed)	MR, LDSC
3606	Chest pain or discomfort walking normally	MR, LDSC
3616	Chest pain due to walking ceases when standing still	MR, LDSC
3637	Frequency of other exercises in last 4 weeks	MR, LDSC
3647	Duration of other exercises	MR, LDSC
3710	Length of menstrual cycle	MR, LDSC
3731	Former alcohol drinker	MR, LDSC
3741	Stomach/abdominal pain for 3+ months	MR, LDSC
3751	Chest pain or discomfort when walking uphill or hurrying	MR, LDSC
3773	Knee pain for 3+ months	MR, LDSC
3799	Headaches for 3+ months	MR, LDSC
3829	Number of stillbirths	MR
3839	Number of spontaneous miscarriages	MR, LDSC
3849	Number of pregnancy terminations	MR, LDSC
3872	Age of primiparous women at birth of child	MR, LDSC
4041	Gestational diabetes only	MR, LDSC
4067	Facial pains for 3+ months	MR
4079	Diastolic blood pressure, automated reading	MR, LDSC
4080	Systolic blood pressure, automated reading	MR, LDSC
4106	Heel bone mineral density (BMD) T-score, automated (left)	MR, LDSC
4125	Heel bone mineral density (BMD) T-score, automated (right)	MR, LDSC
4194	Pulse rate	MR, LDSC
4195	Pulse wave reflection index	MR, LDSC
4196	Pulse wave peak to peak time	MR, LDSC
4407	Average monthly red wine intake	MR, LDSC

4418	Average monthly champagne plus white wine intake	MR, LDSC
4429	Average monthly beer plus cider intake	MR, LDSC
4440	Average monthly spirits intake	MR, LDSC
4451	Average monthly fortified wine intake	MR, LDSC
4462	Average monthly intake of other alcoholic drinks	MR, LDSC
4501	Non-accidental death in close genetic family	MR, LDSC
4526	Happiness	MR, LDSC
4537	Work/job satisfaction	MR, LDSC
4548	Health satisfaction	MR, LDSC
4559	Family relationship satisfaction	MR, LDSC
4570	Friendships satisfaction	MR, LDSC
4581	Financial situation satisfaction	MR, LDSC
4598	Ever depressed for a whole week	MR, LDSC
4609	Longest period of depression	MR, LDSC
4620	Number of depression episodes	MR, LDSC
4631	Ever unenthusiastic/disinterested for a whole week	MR, LDSC
4642	Ever manic/hyper for 2 days	MR, LDSC
4653	Ever highly irritable/argumentative for 2 days	MR, LDSC
4717	Shortness of breath walking on level ground	MR, LDSC
4728	Leg pain on walking	MR, LDSC
4792	Cochlear implant	MR, LDSC
4814	Tinnitus severity/nuisance	MR, LDSC
4825	Noisy workplace	MR, LDSC
4836	Loud music exposure frequency	MR, LDSC
5057	Number of older siblings	MR, LDSC
5364	Average weekly intake of other alcoholic drinks	MR, LDSC
5375	Longest period of unenthusiasm / disinterest	MR, LDSC
5386	Number of unenthusiastic/disinterested episodes	MR, LDSC
5452	Leg pain when standing still or sitting	MR, LDSC
5463	Leg pain in calf/calves	MR, LDSC

5474	Leg pain when walking uphill or hurrying	MR, LDSC
5485	Leg pain when walking normally	MR, LDSC
5496	Leg pain when walking ever disappears while walking	MR, LDSC
5507	Leg pain on walking: action taken	MR, LDSC
5518	Leg pain on walking: effect of standing still	MR, LDSC
5529	Surgery on leg arteries (other than for varicose veins)	MR, LDSC
5663	Length of longest manic/irritable episode	MR, LDSC
5674	Severity of manic/irritable episodes	MR
5959	Previously smoked cigarettes on most/all days	MR
6015	Chest pain felt during physical activity	MR, LDSC
6016	Chest pain felt outside physical activity	MR, LDSC
6017	Able to walk or cycle unaided for 10 minutes	MR, LDSC
6032	Maximum workload during fitness test	MR, LDSC
6033	Maximum heart rate during fitness test	MR, LDSC
6034	Target heart rate achieved	MR, LDSC
6038	Number of trend entries	MR, LDSC
6039	Duration of fitness test	MR, LDSC
20015	Sitting height	MR, LDSC
20016	Fluid intelligence score	MR, LDSC
20018	Prospective memory result	MR, LDSC
20022	Birth weight	MR, LDSC
20023	Mean time to correctly identify matches	MR, LDSC
20121	Cascot confidence score	MR
20122	Bipolar disorder status	MR, LDSC
20127	Neuroticism score	MR, LDSC
20150	Forced expiratory volume in 1-second (FEV1), Best measure	MR, LDSC
20151	Forced vital capacity (FVC), Best measure	MR, LDSC
20152	Reproduciblity of spirometry measurement using ERS/ATS criteria	MR, LDSC
20153	Forced expiratory volume in 1-second (FEV1), predicted	MR, LDSC
20154	Forced expiratory volume in 1-second (FEV1), predicted percentage	MR, LDSC

20160	Ever smoked	MR, LDSC
20161	Pack years of smoking PREVIEW ONLY	MR, LDSC
20162	Pack years adult smoking as proportion of life span exposed to smoking PREVIEW ONLY	MR, LDSC
21001	Body mass index (BMI)	MR, LDSC
21002	Weight	MR, LDSC
21021	Pulse wave Arterial Stiffness index	MR, LDSC
22126	Doctor diagnosed hayfever or allergic rhinitis	MR, LDSC
22127	Doctor diagnosed asthma	MR, LDSC
22128	Doctor diagnosed emphysema	MR, LDSC
22129	Doctor diagnosed chronic bronchitis	MR
22130	Doctor diagnosed COPD (chronic obstructive pulmonary disease)	MR, LDSC
22133	Doctor diagnosed sarcoidosis	MR, LDSC
22134	Doctor diagnosed bronchiectasis	MR, LDSC
22135	Doctor diagnosed idiopathic pulmonary fibrosis	MR, LDSC
22137	Doctor diagnosed tuberculosis	MR, LDSC
22140	Doctor diagnosed lung cancer (not mesothelioma)	MR
22502	Cough on most days	MR, LDSC
22503	Years of cough on most days	MR, LDSC
22504	Bring up phlegm/sputum/mucus on most days	MR, LDSC
23099	Body fat percentage	MR, LDSC
23100	Whole body fat mass	MR, LDSC
23101	Whole body fat-free mass	MR, LDSC
23102	Whole body water mass	MR, LDSC
23105	Basal metabolic rate	MR, LDSC
23106	Impedance of whole body	MR, LDSC
23107	Impedance of leg (right)	MR, LDSC
23108	Impedance of leg (left)	MR, LDSC
23109	Impedance of arm (right)	MR, LDSC
23110	Impedance of arm (left)	MR, LDSC
23111	Leg fat percentage (right)	MR, LDSC

23112	Leg fat mass (right)	MR, LDSC
23113	Leg fat-free mass (right)	MR, LDSC
23114	Leg predicted mass (right)	MR, LDSC
23115	Leg fat percentage (left)	MR, LDSC
23116	Leg fat mass (left)	MR, LDSC
23117	Leg fat-free mass (left)	MR, LDSC
23118	Leg predicted mass (left)	MR, LDSC
23119	Arm fat percentage (right)	MR, LDSC
23120	Arm fat mass (right)	MR, LDSC
23121	Arm fat-free mass (right)	MR, LDSC
23122	Arm predicted mass (right)	MR, LDSC
23123	Arm fat percentage (left)	MR, LDSC
23124	Arm fat mass (left)	MR, LDSC
23125	Arm fat-free mass (left)	MR, LDSC
23126	Arm predicted mass (left)	MR, LDSC
23127	Trunk fat percentage	MR, LDSC
23128	Trunk fat mass	MR, LDSC
23129	Trunk fat-free mass	MR, LDSC
23130	Trunk predicted mass	MR, LDSC
30510	Creatinine (enzymatic) in urine	MR, LDSC
30520	Potassium in urine	MR, LDSC
30530	Sodium in urine	MR, LDSC
40007	Age at death	MR
40009	Reported occurrences of cancer	MR, LDSC
90088	No-wear time bias adjusted acceleration standard deviation	MR, LDSC
1707_2	Handedness (chirality/laterality): Left-handed	MR, LDSC
1707_3	Handedness (chirality/laterality): Use both right and left hands equally	MR, LDSC
20001_1001	Cancer code, self-reported: lung cancer	MR, LDSC
20001_1002	Cancer code, self-reported: breast cancer	MR, LDSC
20001_1003	Cancer code, self-reported: skin cancer	MR, LDSC

20001_1004	Cancer code, self-reported: cancer of lip/mouth/pharynx/oral cavity	MR
20001_1006	Cancer code, self-reported: larynx/throat cancer	MR
20001_1011	Cancer code, self-reported: tongue cancer	MR, LDSC
20001_1017	Cancer code, self-reported: oesophageal cancer	MR, LDSC
20001_1018	Cancer code, self-reported: stomach cancer	MR
20001_1019	Cancer code, self-reported: small intestine/small bowel cancer	MR, LDSC
20001_1020	Cancer code, self-reported: large bowel cancer/colorectal cancer	MR
20001_1022	Cancer code, self-reported: colon cancer/sigmoid cancer	MR, LDSC
20001_1023	Cancer code, self-reported: rectal cancer	MR, LDSC
20001_1024	Cancer code, self-reported: liver/hepatocellular cancer	MR, LDSC
20001_1030	Cancer code, self-reported: eye and/or adnexal cancer	MR, LDSC
20001_1032	Cancer code, self-reported: brain cancer / primary malignant brain tumour	MR, LDSC
20001_1034	Cancer code, self-reported: kidney/renal cell cancer	MR, LDSC
20001_1035	Cancer code, self-reported: bladder cancer	MR, LDSC
20001_1039	Cancer code, self-reported: ovarian cancer	MR
20001_1040	Cancer code, self-reported: uterine/endometrial cancer	MR, LDSC
20001_1041	Cancer code, self-reported: cervical cancer	MR, LDSC
20001_1044	Cancer code, self-reported: prostate cancer	MR, LDSC
20001_1045	Cancer code, self-reported: testicular cancer	MR, LDSC
20001_1047	Cancer code, self-reported: lymphoma	MR
20001_1048	Cancer code, self-reported: leukaemia	MR
20001_1050	Cancer code, self-reported: multiple myeloma	MR
20001_1052	Cancer code, self-reported: hodgkins lymphoma / hodgkins disease	MR
20001_1053	Cancer code, self-reported: non-hodgkins lymphoma	MR
20001_1055	Cancer code, self-reported: chronic lymphocytic	MR, LDSC
20001_1056	Cancer code, self-reported: chronic myeloid	MR, LDSC
20001_1059	Cancer code, self-reported: malignant melanoma	MR, LDSC
20001_1060	Cancer code, self-reported: non-melanoma skin cancer	MR
20001_1061	Cancer code, self-reported: basal cell carcinoma	MR, LDSC
20001_1062	Cancer code, self-reported: squamous cell carcinoma	MR, LDSC

20001_1063	Cancer code, self-reported: primary bone cancer	MR
20001_1065	Cancer code, self-reported: thyroid cancer	MR, LDSC
20001_1068	Cancer code, self-reported: sarcoma/fibrosarcoma	MR, LDSC
20001_1070	Cancer code, self-reported: malignant lymph node, unspecified	MR
20001_1072	Cancer code, self-reported: cin/pre-cancer cells cervix	MR, LDSC
20001_1073	Cancer code, self-reported: rodent ulcer	MR, LDSC
20001_99999	Cancer code, self-reported: unclassifiable	MR, LDSC
20002_1065	Non-cancer illness code, self-reported: hypertension	MR, LDSC
20002_1066	Non-cancer illness code, self-reported: heart/cardiac problem	MR
20002_1067	Non-cancer illness code, self-reported: peripheral vascular disease	MR, LDSC
20002_1068	Non-cancer illness code, self-reported: venous thromboembolic disease	MR, LDSC
20002_1072	Non-cancer illness code, self-reported: essential hypertension	MR
20002_1073	Non-cancer illness code, self-reported: gestational hypertension/pre-eclampsia	MR, LDSC
20002_1074	Non-cancer illness code, self-reported: angina	MR, LDSC
20002_1075	Non-cancer illness code, self-reported: heart attack/myocardial infarction	MR, LDSC
20002_1076	Non-cancer illness code, self-reported: heart failure/pulmonary odema	MR, LDSC
20002_1077	Non-cancer illness code, self-reported: heart arrhythmia	MR
20002_1078	Non-cancer illness code, self-reported: heart valve problem/heart murmur	MR, LDSC
20002_1079	Non-cancer illness code, self-reported: cardiomyopathy	MR, LDSC
20002_1080	Non-cancer illness code, self-reported: pericardial problem	MR, LDSC
20002_1081	Non-cancer illness code, self-reported: stroke	MR, LDSC
20002_1082	Non-cancer illness code, self-reported: transient ischaemic attack (tia)	MR, LDSC
20002_1083	Non-cancer illness code, self-reported: subdural haemorrhage/haematoma	MR
20002_1086	Non-cancer illness code, self-reported: subarachnoid haemorrhage	MR
20002_1087	Non-cancer illness code, self-reported: leg claudication/ intermittent claudication	MR, LDSC
20002_1093	Non-cancer illness code, self-reported: pulmonary embolism +/- dvt	MR, LDSC
20002_1094	Non-cancer illness code, self-reported: deep venous thrombosis (dvt)	MR, LDSC
20002_1111	Non-cancer illness code, self-reported: asthma	MR, LDSC
20002_1112	Non-cancer illness code, self-reported: chronic obstructive airways disease/copd	MR, LDSC
20002_1113	Non-cancer illness code, self-reported: emphysema/chronic bronchitis	MR, LDSC

20002_1114	Non-cancer illness code, self-reported: bronchiectasis	MR, LDSC
20002_1115	Non-cancer illness code, self-reported: interstitial lung disease	MR, LDSC
20002_1117	Non-cancer illness code, self-reported: other respiratory problems	MR
20002_1120	Non-cancer illness code, self-reported: asbestosis	MR
20002_1121	Non-cancer illness code, self-reported: pulmonary fibrosis	MR, LDSC
20002_1122	Non-cancer illness code, self-reported: fibrosing alveolitis/unspecified alveolitis	MR, LDSC
20002_1123	Non-cancer illness code, self-reported: sleep apnoea	MR, LDSC
20002_1125	Non-cancer illness code, self-reported: pleurisy	MR, LDSC
20002_1126	Non-cancer illness code, self-reported: spontaneous pneumothorax/recurrent pneumothorax	MR, LDSC
20002_1134	Non-cancer illness code, self-reported: oesophageal disorder	MR, LDSC
20002_1135	Non-cancer illness code, self-reported: stomach disorder	MR, LDSC
20002_1136	Non-cancer illness code, self-reported: liver/biliary/pancreas problem	MR, LDSC
20002_1137	Non-cancer illness code, self-reported: other abdominal problem	MR, LDSC
20002_1138	Non-cancer illness code, self-reported: gastro-oesophageal reflux (gord) / gastric reflux	MR, LDSC
20002_1139	Non-cancer illness code, self-reported: oesophagitis/barretts oesophagus	MR, LDSC
20002_1140	Non-cancer illness code, self-reported: oesophageal stricture	MR
20002_1142	Non-cancer illness code, self-reported: gastric/stomach ulcers	MR, LDSC
20002_1143	Non-cancer illness code, self-reported: gastritis/gastric erosions	MR
20002_1154	Non-cancer illness code, self-reported: irritable bowel syndrome	MR, LDSC
20002_1155	Non-cancer illness code, self-reported: hepatitis	MR
20002_1156	Non-cancer illness code, self-reported: infective/viral hepatitis	MR
20002_1157	Non-cancer illness code, self-reported: non-infective hepatitis	MR
20002_1158	Non-cancer illness code, self-reported: liver failure/cirrhosis	MR, LDSC
20002_1161	Non-cancer illness code, self-reported: gall bladder disease	MR, LDSC
20002_1162	Non-cancer illness code, self-reported: cholelithiasis/gall stones	MR, LDSC
20002_1163	Non-cancer illness code, self-reported: cholecystitis	MR
20002_1165	Non-cancer illness code, self-reported: pancreatitis	MR, LDSC
20002_1190	Non-cancer illness code, self-reported: peritonitis	MR, LDSC
20002_1191	Non-cancer illness code, self-reported: gastrointestinal bleeding	MR, LDSC
20002_1192	Non-cancer illness code, self-reported: renal/kidney failure	MR

20002_1193	Non-cancer illness code, self-reported: renal failure requiring dialysis	MR, LDSC
20002_1194	Non-cancer illness code, self-reported: renal failure not requiring dialysis	MR, LDSC
20002_1196	Non-cancer illness code, self-reported: urinary tract infection/kidney infection	MR, LDSC
20002_1197	Non-cancer illness code, self-reported: kidney stone/ureter stone/bladder stone	MR, LDSC
20002_1200	Non-cancer illness code, self-reported: ureteric obstruction/hydronephrosis	MR, LDSC
20002_1201	Non-cancer illness code, self-reported: bladder problem (not cancer)	MR, LDSC
20002_1202	Non-cancer illness code, self-reported: urinary frequency / incontinence	MR, LDSC
20002_1207	Non-cancer illness code, self-reported: prostate problem (not cancer)	MR
20002_1214	Non-cancer illness code, self-reported: testicular problems (not cancer)	MR, LDSC
20002_1220	Non-cancer illness code, self-reported: diabetes	MR, LDSC
20002_1221	Non-cancer illness code, self-reported: gestational diabetes	MR
20002_1222	Non-cancer illness code, self-reported: type 1 diabetes	MR
20002_1223	Non-cancer illness code, self-reported: type 2 diabetes	MR, LDSC
20002_1224	Non-cancer illness code, self-reported: thyroid problem (not cancer)	MR
20002_1225	Non-cancer illness code, self-reported: hyperthyroidism/thyrotoxicosis	MR
20002_1226	Non-cancer illness code, self-reported: hypothyroidism/myxoedema	MR
20002_1228	Non-cancer illness code, self-reported: thyroid radioablation therapy	MR
20002_1229	Non-cancer illness code, self-reported: parathyroid gland problem (not cancer)	MR
20002_1230	Non-cancer illness code, self-reported: parathyroid hyperplasia/adenoma	MR
20002_1234	Non-cancer illness code, self-reported: adrenocortical insufficiency/addison's disease	MR
20002_1238	Non-cancer illness code, self-reported: pituitary adenoma/tumour	MR, LDSC
20002_1240	Non-cancer illness code, self-reported: neurological injury/trauma	MR
20002_1242	Non-cancer illness code, self-reported: eye/eyelid problem	MR, LDSC
20002_1243	Non-cancer illness code, self-reported: psychological/psychiatric problem	MR
20002_1246	Non-cancer illness code, self-reported: encephalitis	MR, LDSC
20002_1247	Non-cancer illness code, self-reported: meningitis	MR
20002_1249	Non-cancer illness code, self-reported: cranial nerve problem/palsy	MR
20002_1250	Non-cancer illness code, self-reported: bell's palsy/facial nerve palsy	MR, LDSC
20002_1251	Non-cancer illness code, self-reported: spinal cord disorder	MR, LDSC
20002_1254	Non-cancer illness code, self-reported: peripheral nerve disorder	MR, LDSC

20002_1255	Non-cancer illness code, self-reported: peripheral neuropathy	MR
20002_1256	Non-cancer illness code, self-reported: acute infective polyneuritis/guillain-barre syndrome	MR, LDSC
20002_1257	Non-cancer illness code, self-reported: trapped nerve/compressed nerve	MR, LDSC
20002_1258	Non-cancer illness code, self-reported: chronic/degenerative neurological problem	MR
20002_1261	Non-cancer illness code, self-reported: multiple sclerosis	MR, LDSC
20002_1262	Non-cancer illness code, self-reported: parkinsons disease	MR, LDSC
20002_1263	Non-cancer illness code, self-reported: dementia/alzheimers/cognitive impairment	MR
20002_1264	Non-cancer illness code, self-reported: epilepsy	MR, LDSC
20002_1265	Non-cancer illness code, self-reported: migraine	MR, LDSC
20002_1266	Non-cancer illness code, self-reported: head injury	MR, LDSC
20002_1267	Non-cancer illness code, self-reported: spinal injury	MR, LDSC
20002_1274	Non-cancer illness code, self-reported: eye infection	MR
20002_1275	Non-cancer illness code, self-reported: retinal problem	MR
20002_1276	Non-cancer illness code, self-reported: diabetic eye disease	MR, LDSC
20002_1277	Non-cancer illness code, self-reported: glaucoma	MR, LDSC
20002_1278	Non-cancer illness code, self-reported: cataract	MR, LDSC
20002_1279	Non-cancer illness code, self-reported: eye trauma	MR
20002_1281	Non-cancer illness code, self-reported: retinal detachment	MR, LDSC
20002_1282	Non-cancer illness code, self-reported: retinal artery/vein occlusion	MR, LDSC
20002_1286	Non-cancer illness code, self-reported: depression	MR, LDSC
20002_1287	Non-cancer illness code, self-reported: anxiety/panic attacks	MR, LDSC
20002_1288	Non-cancer illness code, self-reported: nervous breakdown	MR
20002_1289	Non-cancer illness code, self-reported: schizophrenia	MR
20002_1290	Non-cancer illness code, self-reported: deliberate self-harm/suicide attempt	MR
20002_1291	Non-cancer illness code, self-reported: mania/bipolar disorder/manic depression	MR, LDSC
20002_1293	Non-cancer illness code, self-reported: bone disorder	MR, LDSC
20002_1294	Non-cancer illness code, self-reported: back problem	MR, LDSC
20002_1295	Non-cancer illness code, self-reported: joint disorder	MR, LDSC
20002_1297	Non-cancer illness code, self-reported: muscle/soft tissue problem	MR, LDSC
20002_1308	Non-cancer illness code, self-reported: osteomyelitis	MR

20002_1309	Non-cancer illness code, self-reported: osteoporosis	MR, LDSC
20002_1311	Non-cancer illness code, self-reported: spine arthritis/spondylitis	MR, LDSC
20002_1312	Non-cancer illness code, self-reported: prolapsed disc/slipped disc	MR, LDSC
20002_1313	Non-cancer illness code, self-reported: ankylosing spondylitis	MR, LDSC
20002_1322	Non-cancer illness code, self-reported: myositis/myopathy	MR
20002_1327	Non-cancer illness code, self-reported: low platelets/platelet disorder	MR
20002_1330	Non-cancer illness code, self-reported: iron deficiency anaemia	MR, LDSC
20002_1331	Non-cancer illness code, self-reported: pernicious anaemia	MR, LDSC
20002_1340	Non-cancer illness code, self-reported: thalassaemia	MR
20002_1348	Non-cancer illness code, self-reported: gynaecological disorder (not cancer)	MR
20002_1349	Non-cancer illness code, self-reported: ovarian cyst or cysts	MR, LDSC
20002_1350	Non-cancer illness code, self-reported: polycystic ovaries/polycystic ovarian syndrome	MR, LDSC
20002_1351	Non-cancer illness code, self-reported: uterine fibroids	MR, LDSC
20002_1352	Non-cancer illness code, self-reported: uterine polyps	MR
20002_1353	Non-cancer illness code, self-reported: vaginal prolapse/uterine prolapse	MR, LDSC
20002_1364	Non-cancer illness code, self-reported: breast disease (not cancer)	MR
20002_1366	Non-cancer illness code, self-reported: fibrocystic disease	MR
20002_1367	Non-cancer illness code, self-reported: breast cysts	MR, LDSC
20002_1371	Non-cancer illness code, self-reported: sarcoidosis	MR, LDSC
20002_1372	Non-cancer illness code, self-reported: vasculitis	MR
20002_1373	Non-cancer illness code, self-reported: connective tissue disorder	MR
20002_1374	Non-cancer illness code, self-reported: allergy/hypersensitivity/anaphylaxis	MR
20002_1376	Non-cancer illness code, self-reported: giant cell/temporal arteritis	MR
20002_1377	Non-cancer illness code, self-reported: polymyalgia rheumatica	MR, LDSC
20002_1378	Non-cancer illness code, self-reported: wegners granulmatosis	MR
20002_1381	Non-cancer illness code, self-reported: systemic lupus erythematosis/sle	MR, LDSC
20002_1382	Non-cancer illness code, self-reported: sjogren's syndrome/sicca syndrome	MR
20002_1384	Non-cancer illness code, self-reported: scleroderma/systemic sclerosis	MR
20002_1385	Non-cancer illness code, self-reported: allergy or anaphylactic reaction to food	MR, LDSC
20002_1386	Non-cancer illness code, self-reported: allergy or anaphylactic reaction to drug	MR, LDSC

20002_1387	Non-cancer illness code, self-reported: hayfever/allergic rhinitis	MR, LDSC
20002_1394	Non-cancer illness code, self-reported: peripheral nerve injury	MR, LDSC
20002_1396	Non-cancer illness code, self-reported: enlarged prostate	MR, LDSC
20002_1398	Non-cancer illness code, self-reported: pneumonia	MR, LDSC
20002_1400	Non-cancer illness code, self-reported: peptic ulcer	MR, LDSC
20002_1402	Non-cancer illness code, self-reported: endometriosis	MR, LDSC
20002_1403	Non-cancer illness code, self-reported: female infertility	MR
20002_1405	Non-cancer illness code, self-reported: other renal/kidney problem	MR, LDSC
20002_1406	Non-cancer illness code, self-reported: muscle or soft tissue injuries	MR, LDSC
20002_1407	Non-cancer illness code, self-reported: burns	MR, LDSC
20002_1408	Non-cancer illness code, self-reported: alcohol dependency	MR, LDSC
20002_1412	Non-cancer illness code, self-reported: bronchitis	MR, LDSC
20002_1413	Non-cancer illness code, self-reported: nasal/sinus disorder	MR
20002_1414	Non-cancer illness code, self-reported: throat or larynx disorder	MR
20002_1415	Non-cancer illness code, self-reported: ear/vestibular disorder	MR, LDSC
20002_1416	Non-cancer illness code, self-reported: chronic sinusitis	MR
20002_1417	Non-cancer illness code, self-reported: nasal polyps	MR, LDSC
20002_1419	Non-cancer illness code, self-reported: vocal cord polyp	MR
20002_1420	Non-cancer illness code, self-reported: otosclerosis	MR, LDSC
20002_1421	Non-cancer illness code, self-reported: meniere's disease	MR, LDSC
20002_1425	Non-cancer illness code, self-reported: cerebral aneurysm	MR, LDSC
20002_1426	Non-cancer illness code, self-reported: myocarditis	MR
20002_1427	Non-cancer illness code, self-reported: polycystic kidney	MR, LDSC
20002_1428	Non-cancer illness code, self-reported: thyroiditis	MR
20002_1430	Non-cancer illness code, self-reported: hypopituitarism	MR, LDSC
20002_1433	Non-cancer illness code, self-reported: cerebral palsy	MR
20002_1434	Non-cancer illness code, self-reported: other neurological problem	MR
20002_1435	Non-cancer illness code, self-reported: optic neuritis	MR
20002_1436	Non-cancer illness code, self-reported: headaches (not migraine)	MR, LDSC
20002_1437	Non-cancer illness code, self-reported: myasthenia gravis	MR

20002_1438	Non-cancer illness code, self-reported: polycythaemia vera	MR, LDSC
20002_1439	Non-cancer illness code, self-reported: hiv/aids	MR, LDSC
20002_1440	Non-cancer illness code, self-reported: tuberculosis (tb)	MR
20002_1441	Non-cancer illness code, self-reported: malaria	MR, LDSC
20002_1442	Non-cancer illness code, self-reported: helicobacter pylori	MR, LDSC
20002_1445	Non-cancer illness code, self-reported: clotting disorder/excessive bleeding	MR, LDSC
20002_1446	Non-cancer illness code, self-reported: anaemia	MR
20002_1448	Non-cancer illness code, self-reported: neutropenia/lymphopenia	MR
20002_1451	Non-cancer illness code, self-reported: hereditary/genetic haematological disorder	MR, LDSC
20002_1452	Non-cancer illness code, self-reported: eczema/dermatitis	MR, LDSC
20002_1453	Non-cancer illness code, self-reported: psoriasis	MR, LDSC
20002_1454	Non-cancer illness code, self-reported: blistering/desquamating skin disorder	MR, LDSC
20002_1455	Non-cancer illness code, self-reported: chronic skin ulcers	MR
20002_1456	Non-cancer illness code, self-reported: malabsorption/coeliac disease	MR, LDSC
20002_1457	Non-cancer illness code, self-reported: duodenal ulcer	MR, LDSC
20002_1458	Non-cancer illness code, self-reported: diverticular disease/diverticulitis	MR, LDSC
20002_1459	Non-cancer illness code, self-reported: colitis/not crohns or ulcerative colitis	MR
20002_1460	Non-cancer illness code, self-reported: rectal or colon adenoma/polyps	MR
20002_1461	Non-cancer illness code, self-reported: inflammatory bowel disease	MR, LDSC
20002_1462	Non-cancer illness code, self-reported: crohns disease	MR, LDSC
20002_1463	Non-cancer illness code, self-reported: ulcerative colitis	MR, LDSC
20002_1464	Non-cancer illness code, self-reported: rheumatoid arthritis	MR, LDSC
20002_1465	Non-cancer illness code, self-reported: osteoarthritis	MR, LDSC
20002_1466	Non-cancer illness code, self-reported: gout	MR, LDSC
20002_1467	Non-cancer illness code, self-reported: other joint disorder	MR, LDSC
20002_1468	Non-cancer illness code, self-reported: diabetic neuropathy/ulcers	MR
20002_1469	Non-cancer illness code, self-reported: post-traumatic stress disorder	MR
20002_1470	Non-cancer illness code, self-reported: anorexia/bulimia/other eating disorder	MR, LDSC
20002_1471	Non-cancer illness code, self-reported: atrial fibrillation	MR, LDSC
20002_1472	Non-cancer illness code, self-reported: emphysema	MR, LDSC

20002_1473	Non-cancer illness code, self-reported: high cholesterol	MR, LDSC
20002_1474	Non-cancer illness code, self-reported: hiatus hernia	MR, LDSC
20002_1476	Non-cancer illness code, self-reported: sciatica	MR, LDSC
20002_1477	Non-cancer illness code, self-reported: psoriatic arthropathy	MR, LDSC
20002_1478	Non-cancer illness code, self-reported: cervical spondylosis	MR, LDSC
20002_1479	Non-cancer illness code, self-reported: rheumatic fever	MR, LDSC
20002_1482	Non-cancer illness code, self-reported: chronic fatigue syndrome	MR, LDSC
20002_1485	Non-cancer illness code, self-reported: irregular heart beat	MR
20002_1487	Non-cancer illness code, self-reported: svt / supraventricular tachycardia	MR
20002_1491	Non-cancer illness code, self-reported: brain haemorrhage	MR, LDSC
20002_1494	Non-cancer illness code, self-reported: varicose veins	MR, LDSC
20002_1495	Non-cancer illness code, self-reported: lymphoedema	MR
20002_1497	Non-cancer illness code, self-reported: pneumothorax	MR, LDSC
20002_1499	Non-cancer illness code, self-reported: labyrinthitis	MR, LDSC
20002_1500	Non-cancer illness code, self-reported: vertigo	MR, LDSC
20002_1501	Non-cancer illness code, self-reported: pyloric stenosis	MR, LDSC
20002_1502	Non-cancer illness code, self-reported: appendicitis	MR, LDSC
20002_1503	Non-cancer illness code, self-reported: anal problem	MR, LDSC
20002_1504	Non-cancer illness code, self-reported: anal fissure	MR
20002_1505	Non-cancer illness code, self-reported: haemorrhoids / piles	MR, LDSC
20002_1508	Non-cancer illness code, self-reported: jaundice (unknown cause)	MR
20002_1509	Non-cancer illness code, self-reported: gastroenteritis/dysentry	MR
20002_1510	Non-cancer illness code, self-reported: dyspepsia / indigestion	MR
20002_1511	Non-cancer illness code, self-reported: abdominal hernia	MR, LDSC
20002_1512	Non-cancer illness code, self-reported: umbilical hernia	MR, LDSC
20002_1513	Non-cancer illness code, self-reported: inguinal hernia	MR, LDSC
20002_1514	Non-cancer illness code, self-reported: cystitis	MR
20002_1516	Non-cancer illness code, self-reported: bph / benign prostatic hypertrophy	MR, LDSC
20002_1517	Non-cancer illness code, self-reported: prostatitis	MR
20002_1518	Non-cancer illness code, self-reported: erectile dysfunction / impotence	MR, LDSC

20002_1522	Non-cancer illness code, self-reported: grave's disease	MR
20002_1523	Non-cancer illness code, self-reported: trigemminal neuralgia	MR, LDSC
20002_1525	Non-cancer illness code, self-reported: benign / essential tremor	MR
20002_1526	Non-cancer illness code, self-reported: polio / poliomyelitis	MR, LDSC
20002_1528	Non-cancer illness code, self-reported: macular degeneration	MR
20002_1529	Non-cancer illness code, self-reported: dry eyes	MR, LDSC
20002_1530	Non-cancer illness code, self-reported: iritis	MR, LDSC
20002_1531	Non-cancer illness code, self-reported: post-natal depression	MR, LDSC
20002_1532	Non-cancer illness code, self-reported: disc problem	MR, LDSC
20002_1533	Non-cancer illness code, self-reported: disc degeneration	MR
20002_1534	Non-cancer illness code, self-reported: back pain	MR
20002_1535	Non-cancer illness code, self-reported: scoliosis	MR
20002_1536	Non-cancer illness code, self-reported: spinal stenosis	MR
20002_1537	Non-cancer illness code, self-reported: joint pain	MR
20002_1538	Non-cancer illness code, self-reported: arthritis (nos)	MR, LDSC
20002_1540	Non-cancer illness code, self-reported: plantar fascitis	MR
20002_1541	Non-cancer illness code, self-reported: carpal tunnel syndrome	MR, LDSC
20002_1542	Non-cancer illness code, self-reported: fibromyalgia	MR
20002_1544	Non-cancer illness code, self-reported: dupuytren's contracture	MR, LDSC
20002_1545	Non-cancer illness code, self-reported: neck problem/injury	MR
20002_1548	Non-cancer illness code, self-reported: acne/acne vulgaris	MR, LDSC
20002_1549	Non-cancer illness code, self-reported: lichen planus	MR
20002_1550	Non-cancer illness code, self-reported: lichen sclerosis	MR
20002_1554	Non-cancer illness code, self-reported: cervical intra-epithelial neoplasia (cin) / precancerous cells cervix	MR
20002_1555	Non-cancer illness code, self-reported: cervical polyps	MR, LDSC
20002_1556	Non-cancer illness code, self-reported: menorrhagia (unknown cause)	MR, LDSC
20002_1558	Non-cancer illness code, self-reported: ectopic pregnancy	MR
20002_1559	Non-cancer illness code, self-reported: miscarriage	MR, LDSC
20002_1560	Non-cancer illness code, self-reported: breast fibroadenoma	MR, LDSC
20002_1561	Non-cancer illness code, self-reported: raynaud's phenomenon/disease	MR, LDSC

20002_1562	Non-cancer illness code, self-reported: food intolerance	MR
20002_1563	Non-cancer illness code, self-reported: urticaria	MR, LDSC
20002_1567	Non-cancer illness code, self-reported: infectious mononucleosis / glandular fever / epstein barr virus (ebv)	MR, LDSC
20002_1568	Non-cancer illness code, self-reported: measles / morbillivirus	MR, LDSC
20002_1569	Non-cancer illness code, self-reported: mumps / epidemic parotitis	MR
20002_1570	Non-cancer illness code, self-reported: rubella / german measles	MR
20002_1571	Non-cancer illness code, self-reported: chickenpox	MR, LDSC
20002_1572	Non-cancer illness code, self-reported: whooping cough / pertussis	MR
20002_1573	Non-cancer illness code, self-reported: shingles	MR
20002_1575	Non-cancer illness code, self-reported: herpes simplex	MR
20002_1578	Non-cancer illness code, self-reported: hepatitis a	MR, LDSC
20002_1579	Non-cancer illness code, self-reported: hepatitis b	MR, LDSC
20002_1580	Non-cancer illness code, self-reported: hepatitis c	MR
20002_1585	Non-cancer illness code, self-reported: mitral regurgitation / incompetence	MR
20002_1587	Non-cancer illness code, self-reported: aortic regurgitation / incompetence	MR
20002_1588	Non-cancer illness code, self-reported: hypertrophic cardiomyopathy (hcm / hocm)	MR, LDSC
20002_1589	Non-cancer illness code, self-reported: pericarditis	MR, LDSC
20002_1594	Non-cancer illness code, self-reported: respiratory infection	MR
20002_1597	Non-cancer illness code, self-reported: tinnitus / tiniitis	MR
20002_1598	Non-cancer illness code, self-reported: tonsiltis	MR
20002_1599	Non-cancer illness code, self-reported: constipation	MR
20002_1602	Non-cancer illness code, self-reported: bowel / intestinal obstruction	MR, LDSC
20002_1603	Non-cancer illness code, self-reported: rectal prolapse	MR
20002_1605	Non-cancer illness code, self-reported: femoral hernia	MR, LDSC
20002_1606	Non-cancer illness code, self-reported: incisional hernia	MR
20002_1608	Non-cancer illness code, self-reported: nephritis	MR, LDSC
20002_1610	Non-cancer illness code, self-reported: thyroid goitre	MR, LDSC
20002_1613	Non-cancer illness code, self-reported: blepharitis / eyelid infection	MR
20002_1614	Non-cancer illness code, self-reported: stress	MR
20002_1615	Non-cancer illness code, self-reported: obsessive compulsive disorder (ocd)	MR, LDSC

20002_1616	Non-cancer illness code, self-reported: insomnia	MR
20002_1617	Non-cancer illness code, self-reported: osteopenia	MR, LDSC
20002_1618	Non-cancer illness code, self-reported: soft tissue inflammation	MR
20002_1619	Non-cancer illness code, self-reported: tendonitis / tendinitis / tenosynovitis	MR, LDSC
20002_1620	Non-cancer illness code, self-reported: bursitis	MR, LDSC
20002_1623	Non-cancer illness code, self-reported: tennis elbow / lateral epicondylitis	MR, LDSC
20002_1625	Non-cancer illness code, self-reported: cellulitis	MR, LDSC
20002_1626	Non-cancer illness code, self-reported: fracture skull / head	MR, LDSC
20002_1627	Non-cancer illness code, self-reported: fracture jaw	MR
20002_1628	Non-cancer illness code, self-reported: fracture nose	MR
20002_1629	Non-cancer illness code, self-reported: fracture face / orbit / eye socket	MR
20002_1631	Non-cancer illness code, self-reported: fracture clavicle / collar bone	MR
20002_1632	Non-cancer illness code, self-reported: fracture shoulder / scapula	MR, LDSC
20002_1633	Non-cancer illness code, self-reported: fracture upper arm / humerus / elbow	MR, LDSC
20002_1634	Non-cancer illness code, self-reported: fracture forearm / wrist	MR, LDSC
20002_1635	Non-cancer illness code, self-reported: fracture radius	MR
20002_1636	Non-cancer illness code, self-reported: fracture ulna	MR
20002_1637	Non-cancer illness code, self-reported: fracture wrist / colles fracture	MR
20002_1638	Non-cancer illness code, self-reported: fracture hand	MR
20002_1639	Non-cancer illness code, self-reported: fracture finger	MR, LDSC
20002_1640	Non-cancer illness code, self-reported: fracture thumb	MR
20002_1644	Non-cancer illness code, self-reported: fracture rib	MR
20002_1646	Non-cancer illness code, self-reported: fracture vertebra / crush fracture / vertebral collapse	MR, LDSC
20002_1647	Non-cancer illness code, self-reported: fracture pelvis	MR
20002_1648	Non-cancer illness code, self-reported: fracture neck of femur / hip	MR, LDSC
20002_1649	Non-cancer illness code, self-reported: fracture shaft of femur	MR, LDSC
20002_1650	Non-cancer illness code, self-reported: fracture patella / knee	MR
20002_1651	Non-cancer illness code, self-reported: fracture lower leg / ankle	MR, LDSC
20002_1652	Non-cancer illness code, self-reported: fracture tibia	MR
20002_1653	Non-cancer illness code, self-reported: fracture fibula	MR

20002_1654	Non-cancer illness code, self-reported: fracture foot	MR, LDSC
20002_1655	Non-cancer illness code, self-reported: fracture metatarsal	MR
20002_1656	Non-cancer illness code, self-reported: fracture toe	MR
20002_1657	Non-cancer illness code, self-reported: septicaemia / sepsis	MR
20002_1659	Non-cancer illness code, self-reported: meningioma / benign meningeal tumour	MR
20002_1660	Non-cancer illness code, self-reported: rosacea	MR, LDSC
20002_1661	Non-cancer illness code, self-reported: vitiligo	MR, LDSC
20002_1662	Non-cancer illness code, self-reported: cervical erosion	MR, LDSC
20002_1663	Non-cancer illness code, self-reported: abnormal smear (cervix)	MR, LDSC
20002_1664	Non-cancer illness code, self-reported: dysmenorrhoea / dysmenorrhea	MR
20002_1665	Non-cancer illness code, self-reported: menopausal symptoms / menopause	MR
20002_1666	Non-cancer illness code, self-reported: benign breast lump	MR, LDSC
20002_1667	Non-cancer illness code, self-reported: alopecia / hair loss	MR
20002_1668	Non-cancer illness code, self-reported: allergy to house dust mite	MR
20002_1677	Non-cancer illness code, self-reported: scarlet fever / scarlatina	MR, LDSC
20002_1679	Non-cancer illness code, self-reported: undescended testicle	MR
20002_1683	Non-cancer illness code, self-reported: benign neuroma	MR, LDSC
20002_99999	Non-cancer illness code, self-reported: unclassifiable	MR, LDSC
20003_1140851088	Treatment/medication code: senokot 7.5mg tablet	MR, LDSC
20003_1140851812	Treatment/medication code: gtn 400micrograms spray	MR, LDSC
20003_1140852756	Treatment/medication code: vitamin a	MR, LDSC
20003_1140852766	Treatment/medication code: vitamin a+d capsule	MR
20003_1140852948	Treatment/medication code: calcium+vitamin d 500units tablet	MR, LDSC
20003_1140852976	Treatment/medication code: multivitamins	MR, LDSC
20003_1140853200	Treatment/medication code: bromelains	MR
20003_1140854076	Treatment/medication code: macrogol ointment	MR, LDSC
20003_1140855380	Treatment/medication code: salbuvent 100micrograms inhaler	MR
20003_1140856342	Treatment/medication code: syndol tablet	MR
20003_1140857620	Treatment/medication code: depo-provera 50mg/1ml injection	MR
20003_1140857636	Treatment/medication code: prempak 0.625 tablet	MR, LDSC

20003_1140858452	Treatment/medication code: hepacon b12 1mg/1ml injection	MR, LDSC
20003_1140860690	Treatment/medication code: cardura 1mg tablet	MR, LDSC
20003_1140860696	Treatment/medication code: lisinopril	MR, LDSC
20003_1140860728	Treatment/medication code: quinapril	MR
20003_1140860750	Treatment/medication code: captopril	MR, LDSC
20003_1140860790	Treatment/medication code: enalapril maleate+hydrochlorothiazide 20mg/12.5mg tablet	MR, LDSC
20003_1140860802	Treatment/medication code: coversyl 2mg tablet	MR
20003_1140860806	Treatment/medication code: ramipril	MR, LDSC
20003_1140860834	Treatment/medication code: glyceryl trinitrate	MR, LDSC
20003_1140860840	Treatment/medication code: nitrolingual 400micrograms spray	MR, LDSC
20003_1140860904	Treatment/medication code: trandolapril	MR, LDSC
20003_1140860954	Treatment/medication code: isosorbide mononitrate	MR, LDSC
20003_1140860976	Treatment/medication code: imdur 60mg durule	MR, LDSC
20003_1140861008	Treatment/medication code: isosorbide dinitrate	MR, LDSC
20003_1140861088	Treatment/medication code: nifedipine	MR, LDSC
20003_1140861090	Treatment/medication code: adalat 5mg capsule	MR, LDSC
20003_1140861120	Treatment/medication code: coracten sr 10mg m/r capsule	MR
20003_1140861128	Treatment/medication code: tildiem 60mg m/r tablet	MR, LDSC
20003_1140861138	Treatment/medication code: adizem-60 m/r tablet	MR
20003_1140861166	Treatment/medication code: dilzem sr 60mg long acting m/r capsule	MR
20003_1140861202	Treatment/medication code: istin 5mg tablet	MR
20003_1140861276	Treatment/medication code: lacidipine	MR, LDSC
20003_1140861778	Treatment/medication code: dipyridamole	MR, LDSC
20003_1140861780	Treatment/medication code: persantin 25mg tablet	MR
20003_1140861806	Treatment/medication code: aspirin 75mg tablet	MR
20003_1140861832	Treatment/medication code: tranexamic acid	MR, LDSC
20003_1140861924	Treatment/medication code: bezafibrate	MR, LDSC
20003_1140861936	Treatment/medication code: questran 4g/sachet powder	MR
20003_1140861954	Treatment/medication code: fenofibrate	MR, LDSC
20003_1140861958	Treatment/medication code: simvastatin	MR, LDSC

20003_1140861998	Treatment/medication code: ventolin 100micrograms inhaler	MR, LDSC
20003_1140862060	Treatment/medication code: ventolin 2mg tablet	MR
20003_1140862086	Treatment/medication code: salamol 100micrograms inhaler	MR, LDSC
20003_1140862144	Treatment/medication code: salmeterol	MR, LDSC
20003_1140862148	Treatment/medication code: serevent 25mcg inhaler	MR, LDSC
20003_1140862168	Treatment/medication code: bricanyl 250mcg inhaler	MR
20003_1140862236	Treatment/medication code: atrovent 20micrograms inhaler	MR, LDSC
20003_1140862260	Treatment/medication code: aminophylline	MR, LDSC
20003_1140862266	Treatment/medication code: phyllocontin continus 225mg m/r tablet	MR
20003_1140862380	Treatment/medication code: becloforte 250micrograms inhaler	MR
20003_1140862382	Treatment/medication code: becotide 50 inhaler	MR, LDSC
20003_1140862438	Treatment/medication code: uniphyllin continus 200mg m/r tablet	MR, LDSC
20003_1140862476	Treatment/medication code: beclazone 50 inhaler	MR, LDSC
20003_1140862526	Treatment/medication code: sodium cromoglycate	MR
20003_1140862572	Treatment/medication code: budesonide	MR, LDSC
20003_1140862574	Treatment/medication code: pulmicort ls 50micrograms inhaler	MR
20003_1140862628	Treatment/medication code: piriton 4mg tablet	MR, LDSC
20003_1140862760	Treatment/medication code: acrivastine	MR, LDSC
20003_1140862770	Treatment/medication code: zirtek 10mg tablet	MR, LDSC
20003_1140862772	Treatment/medication code: loratadine	MR, LDSC
20003_1140862776	Treatment/medication code: clarityn 10mg tablet	MR
20003_1140862810	Treatment/medication code: phenergan 10mg tablet	MR
20003_1140862944	Treatment/medication code: carbocisteine	MR
20003_1140862952	Treatment/medication code: mucodyne 375mg capsule	MR
20003_1140863144	Treatment/medication code: zopiclone	MR
20003_1140863152	Treatment/medication code: diazepam	MR, LDSC
20003_1140863182	Treatment/medication code: nitrazepam	MR, LDSC
20003_1140863202	Treatment/medication code: temazepam	MR, LDSC
20003_1140863302	Treatment/medication code: lorazepam	MR
20003_1140864070	Treatment/medication code: kapake tablet	MR, LDSC

20003_1140864184	Treatment/medication code: dovonex 50micrograms/g cream	MR, LDSC
20003_1140864196	Treatment/medication code: climagest 1mg tablet	MR, LDSC
20003_1140864286	Treatment/medication code: flixotide 25micrograms inhaler	MR, LDSC
20003_1140864472	Treatment/medication code: xatral 2.5mg tablet	MR
20003_1140864566	Treatment/medication code: cabergoline	MR
20003_1140864618	Treatment/medication code: zestoretic 10 tablet	MR
20003_1140864734	Treatment/medication code: combivent inhaler	MR
20003_1140864752	Treatment/medication code: lansoprazole	MR, LDSC
20003_1140864950	Treatment/medication code: bisoprolol fumarate+hydrochlorothiazide 10mg/6.25mg tablet	MR
20003_1140864952	Treatment/medication code: lisinopril+hydrochlorothiazide 10mg/12.5mg tablet	MR, LDSC
20003_1140864966	Treatment/medication code: salbutamol+ipratropium 100micrograms/20micrograms inhaler	MR, LDSC
20003_1140864992	Treatment/medication code: tramadol	MR, LDSC
20003_1140865000	Treatment/medication code: zydol 50mg capsule	MR, LDSC
20003_1140865010	Treatment/medication code: viscotears liquid eye gel	MR
20003_1140865016	Treatment/medication code: zolpidem	MR
20003_1140865336	Treatment/medication code: spasmonal 60mg capsule	MR, LDSC
20003_1140865350	Treatment/medication code: fybogel mebeverine sachet	MR, LDSC
20003_1140865354	Treatment/medication code: gaviscon liquid	MR, LDSC
20003_1140865382	Treatment/medication code: merbentyl 10mg tablet	MR, LDSC
20003_1140865394	Treatment/medication code: hyoscine butylbromide	MR, LDSC
20003_1140865396	Treatment/medication code: buscopan 10mg tablet	MR, LDSC
20003_1140865414	Treatment/medication code: peppermint oil product	MR
20003_1140865416	Treatment/medication code: colpermin 0.2ml m/r gel e/c capsule	MR
20003_1140865426	Treatment/medication code: cimetidine	MR
20003_1140865564	Treatment/medication code: imodium 2mg capsule	MR
20003_1140865578	Treatment/medication code: mesalazine	MR, LDSC
20003_1140865580	Treatment/medication code: asacol 400mg e/c tablet	MR, LDSC
20003_1140865588	Treatment/medication code: pentasa sr 250mg m/r tablet	MR
20003_1140865618	Treatment/medication code: nizatidine	MR
20003_1140865634	Treatment/medication code: omeprazole	MR, LDSC

20003_1140865654	Treatment/medication code: codeine phosphate+kaolin 10mg/3g/10ml mixture	MR
20003_1140865668	Treatment/medication code: sulphasalazine	MR, LDSC
20003_1140865670	Treatment/medication code: salazopyrin 500mg tablet	MR, LDSC
20003_1140865716	Treatment/medication code: senna	MR, LDSC
20003_1140865752	Treatment/medication code: ispaghula husk	MR
20003_1140865762	Treatment/medication code: regulan 3.6g/sachet powder	MR
20003_1140865786	Treatment/medication code: bisacodyl	MR
20003_1140865800	Treatment/medication code: lactulose product	MR, LDSC
20003_1140865816	Treatment/medication code: anusol cream	MR, LDSC
20003_1140865872	Treatment/medication code: magnesium citrate	MR, LDSC
20003_1140866026	Treatment/medication code: creon e/c granules in capsule	MR, LDSC
20003_1140866078	Treatment/medication code: indapamide	MR, LDSC
20003_1140866116	Treatment/medication code: frusemide	MR
20003_1140866122	Treatment/medication code: bendrofluazide	MR, LDSC
20003_1140866236	Treatment/medication code: spironolactone	MR, LDSC
20003_1140866280	Treatment/medication code: bumetanide	MR
20003_1140866466	Treatment/medication code: securon 40mg tablet	MR, LDSC
20003_1140866738	Treatment/medication code: atenolol	MR, LDSC
20003_1140866800	Treatment/medication code: half-inderal la 80mg m/r capsule	MR, LDSC
20003_1140866804	Treatment/medication code: inderal 10mg tablet	MR
20003_1140867444	Treatment/medication code: risperidone	MR
20003_1140867490	Treatment/medication code: lithium product	MR
20003_1140867504	Treatment/medication code: priadel 200mg m/r tablet	MR, LDSC
20003_1140867624	Treatment/medication code: prothiaden 25mg capsule	MR
20003_1140867726	Treatment/medication code: lofepramine	MR, LDSC
20003_1140867818	Treatment/medication code: nortriptyline	MR, LDSC
20003_1140867876	Treatment/medication code: prozac 20mg capsule	MR, LDSC
20003_1140867878	Treatment/medication code: sertraline	MR
20003_1140867888	Treatment/medication code: paroxetine	MR, LDSC
20003_1140867948	Treatment/medication code: amitriptyline hydrochloride+perphenazine 10mg/2mg tablet	MR, LDSC

20003_1140867998	Treatment/medication code: motilium 10mg tablet	MR
20003_1140868064	Treatment/medication code: serc-8 tablet	MR
20003_1140868080	Treatment/medication code: cyclizine	MR, LDSC
20003_1140868120	Treatment/medication code: trifluoperazine	MR
20003_1140868170	Treatment/medication code: prochlorperazine	MR, LDSC
20003_1140868172	Treatment/medication code: stemetil 5mg tablet	MR
20003_1140868192	Treatment/medication code: buccastem 3mg tablet	MR
20003_1140868226	Treatment/medication code: aspirin	MR, LDSC
20003_1140868364	Treatment/medication code: prednisone	MR, LDSC
20003_1140868372	Treatment/medication code: climaval 1mg tablet	MR, LDSC
20003_1140868406	Treatment/medication code: conjugated oestrogens	MR, LDSC
20003_1140868408	Treatment/medication code: premarin 625micrograms tablet	MR, LDSC
20003_1140868426	Treatment/medication code: triamcinolone	MR
20003_1140868456	Treatment/medication code: oestradiol product	MR
20003_1140868458	Treatment/medication code: hormonin tablet	MR
20003_1140868460	Treatment/medication code: progynova 1mg tablet	MR, LDSC
20003_1140868472	Treatment/medication code: vagifem 25mcg pessary	MR, LDSC
20003_1140868482	Treatment/medication code: tibolone	MR, LDSC
20003_1140868514	Treatment/medication code: trisequens tablet	MR
20003_1140868518	Treatment/medication code: nuvelle tablet	MR
20003_1140868520	Treatment/medication code: estracombi tts patch	MR
20003_1140868532	Treatment/medication code: testosterone product	MR
20003_1140868538	Treatment/medication code: sustanon 100 oily injection	MR, LDSC
20003_1140868550	Treatment/medication code: finasteride	MR
20003_1140868580	Treatment/medication code: norethisterone	MR
20003_1140868588	Treatment/medication code: progesterone product	MR
20003_1140868772	Treatment/medication code: didronel 200mg tablet	MR
20003_1140868816	Treatment/medication code: desmopressin	MR
20003_1140869034	Treatment/medication code: ortho-gynest 500micrograms pessary	MR, LDSC
20003_1140869036	Treatment/medication code: ovestin 0.1% vaginal cream	MR

20003_1140869162	Treatment/medication code: marvelon tablet	MR
20003_1140869164	Treatment/medication code: mercilon tablet	MR, LDSC
20003_1140869176	Treatment/medication code: logynon tablet	MR, LDSC
20003_1140869180	Treatment/medication code: microgynon 30 tablet	MR, LDSC
20003_1140869276	Treatment/medication code: micronor tablet	MR
20003_1140869278	Treatment/medication code: noriday tablet	MR
20003_1140869324	Treatment/medication code: loestrin 20 tablet	MR
20003_1140869346	Treatment/medication code: cilest tablet	MR
20003_1140869362	Treatment/medication code: femulen tablet	MR
20003_1140869370	Treatment/medication code: norgeston tablet	MR, LDSC
20003_1140869848	Treatment/medication code: methotrexate	MR
20003_1140869930	Treatment/medication code: azathioprine	MR, LDSC
20003_1140870164	Treatment/medication code: tamoxifen	MR, LDSC
20003_1140870196	Treatment/medication code: zoladex 3.6mg implant	MR
20003_1140870306	Treatment/medication code: iron+folic acid	MR
20003_1140870390	Treatment/medication code: ferrous sulphate	MR
20003_1140870422	Treatment/medication code: folic acid product	MR
20003_1140870488	Treatment/medication code: forceval capsule	MR
20003_1140870504	Treatment/medication code: hydroxocobalamin product	MR, LDSC
20003_1140870512	Treatment/medication code: cyanocobalamin product	MR
20003_1140870570	Treatment/medication code: vitamin b12 preparation	MR, LDSC
20003_1140870788	Treatment/medication code: calcium salts	MR, LDSC
20003_1140870856	Treatment/medication code: calcichew 1.25g chewable tablet	MR, LDSC
20003_1140870862	Treatment/medication code: calcichew forte 2.5g chewable tablet	MR
20003_1140870932	Treatment/medication code: ascorbic acid product	MR
20003_1140870954	Treatment/medication code: vitamin d product	MR
20003_1140870956	Treatment/medication code: alfacalcidol	MR, LDSC
20003_1140871024	Treatment/medication code: vitamin b compound tablet	MR, LDSC
20003_1140871050	Treatment/medication code: calcichew d3 tablet	MR, LDSC
20003_1140871052	Treatment/medication code: calcium carbonate+cholecalciferol 1.25g/5micrograms tablet	MR, LDSC

20003_1140871112	Treatment/medication code: vitamin e product	MR
20003_1140871138	Treatment/medication code: vitamin k product	MR, LDSC
20003_1140871168	Treatment/medication code: voltarol 25mg e/c tablet	MR
20003_1140871180	Treatment/medication code: rhumalgan 25mg e/c tablet	MR, LDSC
20003_1140871188	Treatment/medication code: etodolac	MR, LDSC
20003_1140871196	Treatment/medication code: lodine 200mg tablet	MR, LDSC
20003_1140871266	Treatment/medication code: arthrotec tablet	MR
20003_1140871310	Treatment/medication code: ibuprofen	MR, LDSC
20003_1140871336	Treatment/medication code: indomethacin	MR
20003_1140871462	Treatment/medication code: naproxen	MR, LDSC
20003_1140871472	Treatment/medication code: naprosyn 250mg tablet	MR
20003_1140871506	Treatment/medication code: ketoprofen	MR
20003_1140871542	Treatment/medication code: mefenamic acid	MR
20003_1140871666	Treatment/medication code: piroxicam	MR, LDSC
20003_1140871680	Treatment/medication code: tylex capsule	MR
20003_1140871688	Treatment/medication code: solpadol caplet	MR, LDSC
20003_1140871692	Treatment/medication code: morphine	MR, LDSC
20003_1140871732	Treatment/medication code: buprenorphine	MR
20003_1140871984	Treatment/medication code: dixarit 25mcg tablet	MR, LDSC
20003_1140871996	Treatment/medication code: sanomigran 500micrograms tablet	MR
20003_1140872036	Treatment/medication code: paramax tablet	MR
20003_1140872058	Treatment/medication code: migril tablet	MR, LDSC
20003_1140872072	Treatment/medication code: tegretol 100mg tablet	MR
20003_1140872112	Treatment/medication code: epanutin 25mg capsule	MR
20003_1140872150	Treatment/medication code: clonazepam	MR, LDSC
20003_1140872198	Treatment/medication code: sodium valproate	MR
20003_1140872200	Treatment/medication code: epilim 100mg crushable tablet	MR, LDSC
20003_1140872228	Treatment/medication code: gabapentin	MR, LDSC
20003_1140872290	Treatment/medication code: lamotrigine	MR
20003_1140872338	Treatment/medication code: madopar 62.5 capsule	MR

20003_1140872492	Treatment/medication code: nicotine product	MR
20003_1140872590	Treatment/medication code: phenoxymethylpenicillin	MR, LDSC
20003_1140872694	Treatment/medication code: flucloxacillin	MR, LDSC
20003_1140873394	Treatment/medication code: doxycycline	MR, LDSC
20003_1140873450	Treatment/medication code: tetracycline	MR
20003_1140873474	Treatment/medication code: lymecycline	MR
20003_1140873476	Treatment/medication code: tetralysal 300 capsule	MR, LDSC
20003_1140873480	Treatment/medication code: minocycline	MR, LDSC
20003_1140873548	Treatment/medication code: oxytetracycline	MR, LDSC
20003_1140873608	Treatment/medication code: erythromycin	MR, LDSC
20003_1140873634	Treatment/medication code: clarithromycin	MR, LDSC
20003_1140873642	Treatment/medication code: azithromycin	MR
20003_1140873966	Treatment/medication code: trimethoprim	MR, LDSC
20003_1140874014	Treatment/medication code: metronidazole	MR, LDSC
20003_1140874126	Treatment/medication code: nitrofurantoin	MR, LDSC
20003_1140874138	Treatment/medication code: ciprofloxacin	MR
20003_1140874370	Treatment/medication code: aciclovir	MR, LDSC
20003_1140874420	Treatment/medication code: quinine	MR, LDSC
20003_1140874686	Treatment/medication code: glucophage 500mg tablet	MR, LDSC
20003_1140874718	Treatment/medication code: glibenclamide	MR
20003_1140874744	Treatment/medication code: gliclazide	MR, LDSC
20003_1140874790	Treatment/medication code: betamethasone	MR
20003_1140874816	Treatment/medication code: dexamethasone	MR
20003_1140874852	Treatment/medication code: thyroxine sodium	MR, LDSC
20003_1140874866	Treatment/medication code: carbimazole	MR, LDSC
20003_1140874896	Treatment/medication code: hydrocortisone	MR
20003_1140874930	Treatment/medication code: prednisolone	MR, LDSC
20003_1140875336	Treatment/medication code: nabumetone	MR
20003_1140875392	Treatment/medication code: plaquenil 200mg tablet	MR
20003_1140875408	Treatment/medication code: allopurinol	MR, LDSC

20003_1140875420	Treatment/medication code: baclofen	MR
20003_1140875486	Treatment/medication code: colchicine	MR, LDSC
20003_1140875630	Treatment/medication code: movelat cream	MR, LDSC
20003_1140875632	Treatment/medication code: movelat gel	MR
20003_1140875816	Treatment/medication code: pilocarpine	MR, LDSC
20003_1140875840	Treatment/medication code: timolol 0.25% eye drops	MR, LDSC
20003_1140875990	Treatment/medication code: hypromellose	MR, LDSC
20003_1140876006	Treatment/medication code: polyvinyl alcohol 1% eye drops	MR, LDSC
20003_1140876076	Treatment/medication code: flixonase 50micrograms aqueous nasal spray	MR, LDSC
20003_1140876136	Treatment/medication code: beconase 50micrograms nasal spray	MR, LDSC
20003_1140876146	Treatment/medication code: rhinocort 50micrograms nasal spray	MR, LDSC
20003_1140876384	Treatment/medication code: glandosane plain spray	MR
20003_1140876404	Treatment/medication code: aqueous cream bp	MR, LDSC
20003_1140876592	Treatment/medication code: multivitamin+mineral preparations	MR, LDSC
20003_1140877630	Treatment/medication code: calcium+ergocalciferol tablet	MR, LDSC
20003_1140877826	Treatment/medication code: sodium bicarbonate	MR
20003_1140877892	Treatment/medication code: voltarol emulgel	MR, LDSC
20003_1140878036	Treatment/medication code: diclofenac sodium+misoprostol	MR, LDSC
20003_1140878172	Treatment/medication code: tears naturale eye drops	MR
20003_1140878184	Treatment/medication code: sno-tears eye drops	MR
20003_1140878186	Treatment/medication code: liquifilm tears 1.4% eye drops	MR, LDSC
20003_1140878190	Treatment/medication code: minims artificial tears single-use eye drops	MR, LDSC
20003_1140878226	Treatment/medication code: diprobase cream	MR
20003_1140878248	Treatment/medication code: oilatum emollient bath additive	MR
20003_1140878304	Treatment/medication code: e45 cream	MR, LDSC
20003_1140878324	Treatment/medication code: oilatum cream	MR, LDSC
20003_1140878378	Treatment/medication code: dovonex 50micrograms/g ointment	MR
20003_1140878498	Treatment/medication code: polytar liquid	MR
20003_1140878512	Treatment/medication code: capasal shampoo	MR, LDSC
20003_1140878530	Treatment/medication code: canesten 1% cream	MR, LDSC

20003_1140879392	Treatment/medication code: manevac granules	MR
20003_1140879404	Treatment/medication code: docusate sodium	MR
20003_1140879406	Treatment/medication code: ranitidine	MR, LDSC
20003_1140879424	Treatment/medication code: alverine	MR
20003_1140879428	Treatment/medication code: mebeverine	MR, LDSC
20003_1140879430	Treatment/medication code: domperidone	MR
20003_1140879464	Treatment/medication code: loperamide	MR, LDSC
20003_1140879494	Treatment/medication code: metoclopramide	MR, LDSC
20003_1140879540	Treatment/medication code: fluoxetine	MR, LDSC
20003_1140879616	Treatment/medication code: amitriptyline	MR, LDSC
20003_1140879620	Treatment/medication code: clomipramine	MR, LDSC
20003_1140879628	Treatment/medication code: dothiepin	MR, LDSC
20003_1140879630	Treatment/medication code: imipramine	MR
20003_1140879634	Treatment/medication code: trazodone	MR, LDSC
20003_1140879644	Treatment/medication code: amantadine	MR, LDSC
20003_1140879658	Treatment/medication code: chlorpromazine	MR
20003_1140879668	Treatment/medication code: selegiline	MR
20003_1140879760	Treatment/medication code: bisoprolol	MR, LDSC
20003_1140879762	Treatment/medication code: celiprolol	MR, LDSC
20003_1140879774	Treatment/medication code: alfuzosin	MR
20003_1140879778	Treatment/medication code: doxazosin	MR, LDSC
20003_1140879782	Treatment/medication code: indoramin	MR, LDSC
20003_1140879792	Treatment/medication code: terbutaline	MR
20003_1140879798	Treatment/medication code: terazosin	MR
20003_1140879802	Treatment/medication code: amlodipine	MR, LDSC
20003_1140879806	Treatment/medication code: diltiazem	MR
20003_1140879818	Treatment/medication code: metoprolol	MR
20003_1140879842	Treatment/medication code: propranolol	MR, LDSC
20003_1140879854	Treatment/medication code: sotalol	MR, LDSC
20003_1140879866	Treatment/medication code: timolol	MR

20003_1140880072	Treatment/medication code: gamolenic acid	MR
20003_1140880086	Treatment/medication code: calcipotriol	MR
20003_1140880288	Treatment/medication code: clotrimazole	MR
20003_1140880462	Treatment/medication code: metrogel 0.75% gel	MR
20003_1140880956	Treatment/medication code: fentanyl	MR, LDSC
20003_1140881320	Treatment/medication code: magnesium carbonate	MR
20003_1140881414	Treatment/medication code: gastrocote liquid	MR
20003_1140881446	Treatment/medication code: proctosedyl ointment	MR
20003_1140881472	Treatment/medication code: lacri-lube eye ointment	MR
20003_1140881474	Treatment/medication code: normacol granules	MR
20003_1140881702	Treatment/medication code: adalate 10mg capsule	MR, LDSC
20003_1140881856	Treatment/medication code: salbutamol	MR, LDSC
20003_1140881882	Treatment/medication code: timoptol 0.25% eye drops	MR
20003_1140881938	Treatment/medication code: beclomethasone dipropionate+salbutamol	MR
20003_1140882082	Treatment/medication code: promethazine product	MR
20003_1140882112	Treatment/medication code: co-careldopa	MR, LDSC
20003_1140882236	Treatment/medication code: seroxat 20mg tablet	MR
20003_1140882272	Treatment/medication code: oramorph 10mg/5ml oral solution	MR
20003_1140882374	Treatment/medication code: co-amoxiclav	MR, LDSC
20003_1140882394	Treatment/medication code: paracetamol + codeine	MR, LDSC
20003_1140882498	Treatment/medication code: penicillin	MR, LDSC
20003_1140882618	Treatment/medication code: diprosalic ointment	MR, LDSC
20003_1140882626	Treatment/medication code: betnesol 0.1% eye/ear/nose drops	MR
20003_1140882694	Treatment/medication code: betnovate cream	MR, LDSC
20003_1140882722	Treatment/medication code: maxidex eye drops	MR
20003_1140882728	Treatment/medication code: otomize ear spray	MR
20003_1140882756	Treatment/medication code: timodine cream	MR
20003_1140882776	Treatment/medication code: fucibet cream	MR, LDSC
20003_1140882782	Treatment/medication code: dermovate cream	MR
20003_1140882800	Treatment/medication code: eumovate cream	MR, LDSC

20003_1140882910	Treatment/medication code: daktacort cream	MR, LDSC
20003_1140882946	Treatment/medication code: livial 2.5mg tablet	MR, LDSC
20003_1140883066	Treatment/medication code: insulin product	MR, LDSC
20003_1140883468	Treatment/medication code: clonidine	MR, LDSC
20003_1140883476	Treatment/medication code: procyclidine	MR, LDSC
20003_1140883504	Treatment/medication code: cetirizine	MR, LDSC
20003_1140883520	Treatment/medication code: chlorpheniramine	MR, LDSC
20003_1140883524	Treatment/medication code: cinnarizine	MR, LDSC
20003_1140883548	Treatment/medication code: ipratropium	MR, LDSC
20003_1140883568	Treatment/medication code: oxybutynin	MR, LDSC
20003_1140883656	Treatment/medication code: hydroxyzine	MR
20003_1140883664	Treatment/medication code: pizotifen	MR, LDSC
20003_1140883706	Treatment/medication code: nedocromil	MR
20003_1140883748	Treatment/medication code: selenium product	MR, LDSC
20003_1140883968	Treatment/medication code: carmellose	MR
20003_1140884308	Treatment/medication code: hydroxychloroquine	MR
20003_1140884412	Treatment/medication code: sumatriptan	MR, LDSC
20003_1140884444	Treatment/medication code: codeine	MR, LDSC
20003_1140884464	Treatment/medication code: dihydrocodeine	MR, LDSC
20003_1140884488	Treatment/medication code: diclofenac	MR, LDSC
20003_1140884512	Treatment/medication code: liothyronine	MR, LDSC
20003_1140884516	Treatment/medication code: thyroxine product	MR, LDSC
20003_1140884560	Treatment/medication code: nefopam	MR
20003_1140884600	Treatment/medication code: metformin	MR, LDSC
20003_1140884622	Treatment/medication code: oestrogen product	MR, LDSC
20003_1140884654	Treatment/medication code: beclomethasone	MR, LDSC
20003_1140884672	Treatment/medication code: fludrocortisone	MR, LDSC
20003_1140884696	Treatment/medication code: clobetasone	MR
20003_1140884700	Treatment/medication code: trimovate ointment	MR
20003_1140888074	Treatment/medication code: clobetasol	MR, LDSC

20003_1140888092	Treatment/medication code: elocon cream	MR
20003_1140888098	Treatment/medication code: fluticasone	MR, LDSC
20003_1140888172	Treatment/medication code: mometasone	MR, LDSC
20003_1140888176	Treatment/medication code: mometasone furoate 0.1% ointment	MR
20003_1140888266	Treatment/medication code: warfarin	MR, LDSC
20003_1140888362	Treatment/medication code: pyridoxine preparation	MR
20003_1140888366	Treatment/medication code: thiamine preparation	MR, LDSC
20003_1140888386	Treatment/medication code: iron product	MR, LDSC
20003_1140888390	Treatment/medication code: ferrous salt product	MR, LDSC
20003_1140888432	Treatment/medication code: potassium product	MR
20003_1140888438	Treatment/medication code: glucose product	MR
20003_1140888502	Treatment/medication code: amiodarone	MR, LDSC
20003_1140888510	Treatment/medication code: verapamil	MR, LDSC
20003_1140888512	Treatment/medication code: amiloride	MR, LDSC
20003_1140888538	Treatment/medication code: zinc product	MR, LDSC
20003_1140888552	Treatment/medication code: enalapril	MR, LDSC
20003_1140888556	Treatment/medication code: fosinopril	MR, LDSC
20003_1140888560	Treatment/medication code: perindopril	MR, LDSC
20003_1140888570	Treatment/medication code: flecainide	MR, LDSC
20003_1140888578	Treatment/medication code: antihypertensive	MR
20003_1140888594	Treatment/medication code: fluvastatin	MR
20003_1140888646	Treatment/medication code: felodipine	MR, LDSC
20003_1140888648	Treatment/medication code: pravastatin	MR
20003_1140888688	Treatment/medication code: betahistine	MR, LDSC
20003_1140888758	Treatment/medication code: penicillin v	MR
20003_1140888762	Treatment/medication code: ismn - isosorbide mononitrate	MR
20003_1140909368	Treatment/medication code: carvedilol	MR, LDSC
20003_1140909482	Treatment/medication code: opticrom allergy eye drops	MR, LDSC
20003_1140909578	Treatment/medication code: losec 10mg capsule	MR
20003_1140909674	Treatment/medication code: cod liver oil capsule	MR, LDSC

20003_1140909702	Treatment/medication code: sulfasalazine	MR
20003_1140909708	Treatment/medication code: furosemide	MR, LDSC
20003_1140909726	Treatment/medication code: vitamin c product	MR, LDSC
20003_1140909766	Treatment/medication code: multivitamins capsule	MR
20003_1140909780	Treatment/medication code: colestyramine	MR, LDSC
20003_1140909786	Treatment/medication code: beclometasone	MR, LDSC
20003_1140909788	Treatment/medication code: sodium cromoglicate	MR, LDSC
20003_1140909790	Treatment/medication code: chlorphenamine	MR, LDSC
20003_1140909806	Treatment/medication code: dosulepin	MR
20003_1140909872	Treatment/medication code: vitamin b1 preparation	MR
20003_1140909874	Treatment/medication code: vitamin b6 preparation	MR, LDSC
20003_1140909936	Treatment/medication code: indometacin	MR, LDSC
20003_1140909954	Treatment/medication code: acyclovir	MR
20003_1140910494	Treatment/medication code: b12 - hydroxocobalamin prep	MR
20003_1140910498	Treatment/medication code: flu - influenza vaccine	MR
20003_1140910512	Treatment/medication code: ismo - isosorbide mononitrate	MR, LDSC
20003_1140910548	Treatment/medication code: iron sulphate	MR, LDSC
20003_1140910640	Treatment/medication code: luteine	MR
20003_1140910698	Treatment/medication code: oil of peppermint	MR
20003_1140910706	Treatment/medication code: phenobarbital	MR, LDSC
20003_1140910766	Treatment/medication code: nicorandil	MR, LDSC
20003_1140910814	Treatment/medication code: sodium thyroxine	MR, LDSC
20003_1140910832	Treatment/medication code: sodium warfarin	MR
20003_1140910884	Treatment/medication code: dovonex scalp solution	MR
20003_1140911636	Treatment/medication code: kalms tablet	MR, LDSC
20003_1140911638	Treatment/medication code: kelp+garlic product	MR, LDSC
20003_1140911640	Treatment/medication code: lecithin product	MR
20003_1140911642	Treatment/medication code: tacrolimus	MR, LDSC
20003_1140911658	Treatment/medication code: imigran 50mg tablet	MR, LDSC
20003_1140911680	Treatment/medication code: starflower oil	MR

20003_1140911682	Treatment/medication code: selenium ace tablet	MR
20003_1140911698	Treatment/medication code: slozem 120mg m/r capsule	MR, LDSC
20003_1140911730	Treatment/medication code: flax oil tablet	MR, LDSC
20003_1140911732	Treatment/medication code: garlic product	MR, LDSC
20003_1140911734	Treatment/medication code: ginkgo forte tablet	MR, LDSC
20003_1140911736	Treatment/medication code: ginseng product	MR
20003_1140911750	Treatment/medication code: deep relief ibuprofen gel	MR, LDSC
20003_1140911754	Treatment/medication code: anadin tablet	MR, LDSC
20003_1140912212	Treatment/medication code: menophase tablet	MR
20003_1140912228	Treatment/medication code: b12 - cyanocobalamin prep	MR, LDSC
20003_1140913292	Treatment/medication code: synalar 1:10 cream	MR, LDSC
20003_1140916282	Treatment/medication code: venlafaxine	MR, LDSC
20003_1140916288	Treatment/medication code: efexor 37.5mg tablet	MR
20003_1140916356	Treatment/medication code: losartan	MR, LDSC
20003_1140916682	Treatment/medication code: evening primrose oil	MR, LDSC
20003_1140916790	Treatment/medication code: evorel 25 patch	MR, LDSC
20003_1140916980	Treatment/medication code: zantac 75 tablet	MR
20003_1140917034	Treatment/medication code: airomir 100micrograms cfc-free inhaler	MR
20003_1140917056	Treatment/medication code: kliofem tablet	MR
20003_1140917394	Treatment/medication code: diclomax sr 75mg m/r capsule	MR, LDSC
20003_1140917428	Treatment/medication code: angitil sr 90 m/r capsule	MR, LDSC
20003_1140917450	Treatment/medication code: oestrogel 1.25g gel	MR
20003_1140921088	Treatment/medication code: tridestra tablet	MR
20003_1140921600	Treatment/medication code: citalopram	MR, LDSC
20003_1140921814	Treatment/medication code: mirena 52mg intrauterine system	MR
20003_1140921822	Treatment/medication code: mirena 20mcg/24hrs intrauterine system	MR
20003_1140921828	Treatment/medication code: dicloflex 25mg e/c tablet	MR, LDSC
20003_1140922174	Treatment/medication code: alendronate sodium	MR, LDSC
20003_1140922562	Treatment/medication code: femoston 1/10 tablet	MR, LDSC
20003_1140922714	Treatment/medication code: dorzolamide	MR

20003_1140922804	Treatment/medication code: premique 0.625mg/5mg tablet	MR, LDSC
20003_1140922806	Treatment/medication code: premique cycle 10mg tablet	MR
20003_1140923018	Treatment/medication code: anastrozole	MR, LDSC
20003_1140923022	Treatment/medication code: arimidex 1mg tablet	MR
20003_1140923276	Treatment/medication code: co-amilozide	MR
20003_1140923336	Treatment/medication code: co-tenidone	MR, LDSC
20003_1140923346	Treatment/medication code: co-codamol	MR, LDSC
20003_1140923348	Treatment/medication code: co-proxamol	MR, LDSC
20003_1140923350	Treatment/medication code: co-dydramol	MR, LDSC
20003_1140923402	Treatment/medication code: co-amilofruse	MR
20003_1140923484	Treatment/medication code: topiramate	MR
20003_1140923572	Treatment/medication code: adipine mr 10 m/r tablet	MR, LDSC
20003_1140923670	Treatment/medication code: gtn - glyceryl trinitrate	MR
20003_1140923688	Treatment/medication code: zoton 15mg capsule	MR
20003_1140923738	Treatment/medication code: femseven 50 patch	MR
20003_1140923852	Treatment/medication code: elleste-solo 1mg tablet	MR, LDSC
20003_1140923920	Treatment/medication code: volsaid retard 75mg m/r tablet	MR, LDSC
20003_1140925800	Treatment/medication code: movicol oral powder	MR, LDSC
20003_1140925978	Treatment/medication code: mycophenolate	MR, LDSC
20003_1140926430	Treatment/medication code: climesse tablet	MR
20003_1140926592	Treatment/medication code: estraderm mx 25 patch	MR, LDSC
20003_1140926606	Treatment/medication code: salbutamol 100micrograms spacehaler	MR, LDSC
20003_1140926732	Treatment/medication code: meloxicam	MR, LDSC
20003_1140926780	Treatment/medication code: adizem-xl plus m/r capsule	MR, LDSC
20003_1140926922	Treatment/medication code: antihistamine 60mg tablet	MR, LDSC
20003_1140926934	Treatment/medication code: tamsulosin	MR, LDSC
20003_1140926940	Treatment/medication code: flomax mr 400micrograms m/r capsule	MR
20003_1140927086	Treatment/medication code: arthrotec 50 tablet	MR, LDSC
20003_1140927320	Treatment/medication code: dermol 500 lotion	MR, LDSC
20003_1140927328	Treatment/medication code: terbinafine	MR, LDSC

20003_1140927730	Treatment/medication code: epaderm ointment	MR, LDSC
20003_1140928004	Treatment/medication code: zimovane ls 3.75mg tablet	MR, LDSC
20003_1140928274	Treatment/medication code: ropinirole	MR
20003_1140928284	Treatment/medication code: moxonidine	MR, LDSC
20003_1140928384	Treatment/medication code: ritonavir	MR
20003_1140928878	Treatment/medication code: zumenon 1mg tablet	MR
20003_1140928916	Treatment/medication code: olanzapine	MR
20003_1140929012	Treatment/medication code: pantoprazole	MR, LDSC
20003_1141145630	Treatment/medication code: nitromin 400micrograms cfc-free spray	MR, LDSC
20003_1141145638	Treatment/medication code: nasobec aqueous 50micrograms nasal spray	MR, LDSC
20003_1141145660	Treatment/medication code: valsartan	MR, LDSC
20003_1141145668	Treatment/medication code: diovan 40mg capsule	MR, LDSC
20003_1141145812	Treatment/medication code: minerals - magnesium	MR
20003_1141145896	Treatment/medication code: letrozole	MR, LDSC
20003_1141145900	Treatment/medication code: femara 2.5mg tablet	MR
20003_1141146138	Treatment/medication code: lipitor 10mg tablet	MR, LDSC
20003_1141146188	Treatment/medication code: latanoprost	MR, LDSC
20003_1141146198	Treatment/medication code: xalatan 0.005% eye drops	MR, LDSC
20003_1141146234	Treatment/medication code: atorvastatin	MR, LDSC
20003_1141146378	Treatment/medication code: natrilix sr 1.5mg m/r tablet	MR
20003_1141146428	Treatment/medication code: fexofenadine	MR
20003_1141146508	Treatment/medication code: nasacort 55micrograms aqueous nasal spray	MR
20003_1141146606	Treatment/medication code: calcium carbonate+cholecalciferol 1.25g/200iu tablet	MR, LDSC
20003_1141146612	Treatment/medication code: calceos chewable tablet	MR
20003_1141150620	Treatment/medication code: zolmitriptan	MR, LDSC
20003_1141150624	Treatment/medication code: zomig 2.5mg tablet	MR, LDSC
20003_1141150750	Treatment/medication code: brimonidine tartrate	MR, LDSC
20003_1141150754	Treatment/medication code: alphagan 0.2% eye drops	MR
20003_1141150944	Treatment/medication code: nasonex 0.05% aqueous nasal spray	MR, LDSC
20003_1141151016	Treatment/medication code: losartan potassium+hydrochlorothiazide 50mg/12.5mg tablet	MR, LDSC

20003_1141151018	Treatment/medication code: cozaar-comp 50mg/12.5mg tablet	MR, LDSC
20003_1141151284	Treatment/medication code: naratriptan	MR
20003_1141151288	Treatment/medication code: naramig 2.5mg tablet	MR
20003_1141151368	Treatment/medication code: sandrena 0.5mg gel	MR, LDSC
20003_1141151474	Treatment/medication code: viazem xl 120mg m/r capsule	MR, LDSC
20003_1141151718	Treatment/medication code: evorel conti patch	MR
20003_1141151946	Treatment/medication code: cipramil 10mg tablet	MR
20003_1141152590	Treatment/medication code: glimepiride	MR
20003_1141152732	Treatment/medication code: mirtazapine	MR, LDSC
20003_1141152848	Treatment/medication code: quetiapine	MR, LDSC
20003_1141152998	Treatment/medication code: irbesartan	MR, LDSC
20003_1141153006	Treatment/medication code: aprovel 75mg tablet	MR
20003_1141153026	Treatment/medication code: lercanidipine	MR
20003_1141153032	Treatment/medication code: zanidip 10mg tablet	MR
20003_1141153236	Treatment/medication code: oxis 6micrograms turbohaler	MR
20003_1141153242	Treatment/medication code: balsalazide disodium	MR
20003_1141153490	Treatment/medication code: amisulpride	MR
20003_1141156644	Treatment/medication code: elleste duet conti tablet	MR
20003_1141156836	Treatment/medication code: candesartan cilexetil	MR, LDSC
20003_1141156846	Treatment/medication code: amias 2mg tablet	MR, LDSC
20003_1141157126	Treatment/medication code: montelukast product	MR
20003_1141157132	Treatment/medication code: singulair 10mg tablet	MR, LDSC
20003_1141157178	Treatment/medication code: senna product	MR, LDSC
20003_1141157252	Treatment/medication code: glyceryl trinitrate product	MR
20003_1141157264	Treatment/medication code: salmeterol product	MR, LDSC
20003_1141157294	Treatment/medication code: hydrocortisone product	MR
20003_1141157324	Treatment/medication code: loratadine product	MR
20003_1141157364	Treatment/medication code: metronidazole product	MR, LDSC
20003_1141157402	Treatment/medication code: prednisolone product	MR, LDSC
20003_1141157412	Treatment/medication code: ibuprofen product	MR

20003_1141157418	Treatment/medication code: budesonide product	MR
20003_1141157458	Treatment/medication code: hypromellose product	MR
20003_1141157486	Treatment/medication code: salbutamol product	MR
20003_1141157494	Treatment/medication code: ispaghula husk product	MR
20003_1141162526	Treatment/medication code: benadryl allergy relief 8mg capsule	MR
20003_1141162544	Treatment/medication code: lipantil micro 67mg capsule	MR, LDSC
20003_1141162764	Treatment/medication code: tolterodine l-tartrate	MR, LDSC
20003_1141162824	Treatment/medication code: detrusitol 1mg tablet	MR, LDSC
20003_1141164060	Treatment/medication code: pramipexole	MR
20003_1141164068	Treatment/medication code: mirapexin 0.088mg tablet	MR, LDSC
20003_1141164086	Treatment/medication code: salmeterol+fluticasone propionate	MR, LDSC
20003_1141164276	Treatment/medication code: nebivolol	MR
20003_1141164616	Treatment/medication code: protium 20mg e/c tablet	MR
20003_1141164828	Treatment/medication code: adcal-d3 1.5g/10micrograms chewable tablet	MR, LDSC
20003_1141164872	Treatment/medication code: sinemet-62.5 tablet	MR
20003_1141165512	Treatment/medication code: kapake capsule	MR
20003_1141166006	Treatment/medication code: telmisartan	MR, LDSC
20003_1141166086	Treatment/medication code: rennie duo oral suspension	MR
20003_1141166200	Treatment/medication code: implanon 68mg subdermal implant	MR, LDSC
20003_1141166294	Treatment/medication code: leflunomide	MR, LDSC
20003_1141167206	Treatment/medication code: oestrogel 0.06% gel	MR
20003_1141167334	Treatment/medication code: colofac-100 tablet	MR
20003_1141167594	Treatment/medication code: qvar 50 inhaler	MR, LDSC
20003_1141167708	Treatment/medication code: beclomist 50micrograms nasal spray	MR
20003_1141167848	Treatment/medication code: asasantin retard m/r capsule	MR
20003_1141167932	Treatment/medication code: rizatriptan	MR, LDSC
20003_1141167940	Treatment/medication code: maxalt 5mg tablet	MR
20003_1141168108	Treatment/medication code: isotard 25xl m/r tablet	MR, LDSC
20003_1141168122	Treatment/medication code: solpadol capsule	MR
20003_1141168318	Treatment/medication code: clopidogrel	MR, LDSC

20003_1141168322	Treatment/medication code: plavix 75mg tablet	MR
20003_1141168326	Treatment/medication code: kliovance 1mg/0.5mg tablet	MR, LDSC
20003_1141168554	Treatment/medication code: migraleve tablet	MR
20003_1141168574	Treatment/medication code: raloxifene hydrochloride	MR
20003_1141168578	Treatment/medication code: evista 60mg tablet	MR
20003_1141168584	Treatment/medication code: rabeprazole sodium	MR, LDSC
20003_1141168590	Treatment/medication code: pariet 10mg e/c tablet	MR, LDSC
20003_1141168648	Treatment/medication code: solpadeine tablet	MR
20003_1141168650	Treatment/medication code: solpadeine capsule	MR, LDSC
20003_1141168680	Treatment/medication code: orlistat	MR, LDSC
20003_1141168684	Treatment/medication code: xenical 120mg capsule	MR
20003_1141168752	Treatment/medication code: peptac liquid	MR, LDSC
20003_1141168936	Treatment/medication code: sildenafil	MR, LDSC
20003_1141168944	Treatment/medication code: viagra 25mg tablet	MR
20003_1141168946	Treatment/medication code: viagra 50mg tablet	MR, LDSC
20003_1141168948	Treatment/medication code: viagra 100mg tablet	MR
20003_1141169516	Treatment/medication code: dorzolamide+timolol	MR
20003_1141169520	Treatment/medication code: cosopt 2%/0.5% eye drops	MR, LDSC
20003_1141169844	Treatment/medication code: dexamethasone+hypromellose 0.1%/0.5% eye drops	MR
20003_1141171038	Treatment/medication code: oxycodone hydrochloride	MR
20003_1141171100	Treatment/medication code: exemestane	MR
20003_1141171152	Treatment/medication code: cardicor 1.25mg tablet	MR
20003_1141171336	Treatment/medication code: eprosartan	MR, LDSC
20003_1141171496	Treatment/medication code: trospium	MR
20003_1141171646	Treatment/medication code: pioglitazone	MR, LDSC
20003_1141171932	Treatment/medication code: levetiracetam	MR
20003_1141171940	Treatment/medication code: keppra 250mg tablet	MR
20003_1141171948	Treatment/medication code: adcal 600mg chewable tablet	MR, LDSC
20003_1141172436	Treatment/medication code: indivina 1mg/2.5mg tablet	MR, LDSC
20003_1141172492	Treatment/medication code: micardis 20mg tablet	MR

20003_1141172682	Treatment/medication code: irbesartan+hydrochlorothiazide 150mg/12.5mg tablet	MR
20003_1141172686	Treatment/medication code: coaprovel 150mg/12.5mg tablet	MR
20003_1141172698	Treatment/medication code: monomax xl 60mg m/r tablet	MR, LDSC
20003_1141172728	Treatment/medication code: almotriptan	MR, LDSC
20003_1141172838	Treatment/medication code: depakote 250mg e/c tablet	MR, LDSC
20003_1141172918	Treatment/medication code: celluvisc 1% single-use eye drops	MR
20003_1141172924	Treatment/medication code: desloratadine	MR
20003_1141172928	Treatment/medication code: neoclarityn 5mg tablet	MR, LDSC
20003_1141173328	Treatment/medication code: ursodeoxycholic acid	MR
20003_1141173348	Treatment/medication code: hydroxocobalamin	MR, LDSC
20003_1141174032	Treatment/medication code: doublebase gel	MR
20003_1141174424	Treatment/medication code: solaraze 3% gel	MR
20003_1141174508	Treatment/medication code: reductil 10mg capsule	MR, LDSC
20003_1141174520	Treatment/medication code: symbicort 100/6 turbohaler	MR, LDSC
20003_1141175684	Treatment/medication code: risedronate sodium	MR, LDSC
20003_1141175690	Treatment/medication code: actonel 5mg tablet	MR
20003_1141176284	Treatment/medication code: brinzolamide	MR, LDSC
20003_1141176288	Treatment/medication code: azopt 10mg/ml eye drops	MR, LDSC
20003_1141176570	Treatment/medication code: fosamax 5mg tablet	MR
20003_1141176662	Treatment/medication code: celecoxib	MR, LDSC
20003_1141176668	Treatment/medication code: celebrex 100mg capsule	MR
20003_1141176670	Treatment/medication code: celebrex 200mg capsule	MR
20003_1141176732	Treatment/medication code: carbomers	MR, LDSC
20003_1141176832	Treatment/medication code: seretide 50 evohaler	MR, LDSC
20003_1141177526	Treatment/medication code: esomeprazole	MR, LDSC
20003_1141177532	Treatment/medication code: nexium 20mg tablet	MR, LDSC
20003_1141177600	Treatment/medication code: rosiglitazone	MR, LDSC
20003_1141178036	Treatment/medication code: eltroxin 25micrograms tablet	MR
20003_1141178052	Treatment/medication code: zapain caplet	MR, LDSC
20003_1141179764	Treatment/medication code: tenofovir	MR

20003_1141179824	Treatment/medication code: yasmin tablet	MR
20003_1141179830	Treatment/medication code: piriteze 10mg tablet	MR
20003_1141179846	Treatment/medication code: protopic 0.03% ointment	MR, LDSC
20003_1141179914	Treatment/medication code: bimatoprost	MR, LDSC
20003_1141179920	Treatment/medication code: lumigan 0.3mg/ml eye drops	MR, LDSC
20003_1141179974	Treatment/medication code: cozaar 25mg tablet	MR, LDSC
20003_1141179992	Treatment/medication code: dovobet ointment	MR, LDSC
20003_1141180036	Treatment/medication code: fybogel orange s/f granules	MR
20003_1141180140	Treatment/medication code: etoricoxib	MR
20003_1141180148	Treatment/medication code: arcoxia 60mg tablet	MR, LDSC
20003_1141180150	Treatment/medication code: arcoxia 90mg tablet	MR, LDSC
20003_1141180212	Treatment/medication code: escitalopram	MR, LDSC
20003_1141180226	Treatment/medication code: amoxicillin	MR
20003_1141180314	Treatment/medication code: ibandronic acid	MR
20003_1141180342	Treatment/medication code: beclometasone dipropionate+salbutamol	MR
20003_1141180392	Treatment/medication code: cefalexin	MR
20003_1141180592	Treatment/medication code: perindopril+indapamide	MR, LDSC
20003_1141180598	Treatment/medication code: coversyl plus 4mg/1.25mg tablet	MR, LDSC
20003_1141180662	Treatment/medication code: zirtek allergy 10mg tablet	MR
20003_1141180936	Treatment/medication code: calcium carbonate+colecalciferol 1.25g/5micrograms tablet	MR
20003_1141181594	Treatment/medication code: estriol product	MR
20003_1141181700	Treatment/medication code: estradiol product	MR
20003_1141181868	Treatment/medication code: omacor 1g capsule	MR, LDSC
20003_1141182628	Treatment/medication code: tiotropium	MR, LDSC
20003_1141182632	Treatment/medication code: spiriva 18micrograms inhalation capsule	MR, LDSC
20003_1141182800	Treatment/medication code: cerazette 75micrograms tablet	MR, LDSC
20003_1141184726	Treatment/medication code: xalacom 0.005%/0.5% eye drops	MR, LDSC
20003_1141184748	Treatment/medication code: levocetirizine	MR, LDSC
20003_1141185316	Treatment/medication code: travoprost	MR, LDSC
20003_1141185326	Treatment/medication code: travatan 40micrograms/ml eye drops	MR, LDSC

20003_1141185986	Treatment/medication code: cetraben emollient cream	MR
20003_1141187230	Treatment/medication code: care cetirizine hayfever relief 10mg tablet	MR
20003_1141187304	Treatment/medication code: codipar caplet	MR
20003_1141187776	Treatment/medication code: nurofen 200mg tablet	MR, LDSC
20003_1141187810	Treatment/medication code: tadalafil	MR
20003_1141187814	Treatment/medication code: cialis 10mg tablet	MR
20003_1141187818	Treatment/medication code: cialis 20mg tablet	MR, LDSC
20003_1141188146	Treatment/medication code: simvador 10mg tablet	MR, LDSC
20003_1141188210	Treatment/medication code: berocca effervescent tablet	MR, LDSC
20003_1141188442	Treatment/medication code: glucosamine product	MR, LDSC
20003_1141188594	Treatment/medication code: humira 40mg injection solution 0.8ml prefilled syringe	MR, LDSC
20003_1141188658	Treatment/medication code: asacol mr 400mg e/c tablet	MR, LDSC
20003_1141188836	Treatment/medication code: felendil xl 5mg m/r tablet	MR
20003_1141189090	Treatment/medication code: rosiglitazone 1mg / metformin 500mg tablet	MR
20003_1141189094	Treatment/medication code: avandamet 1mg / 500mg tablet	MR
20003_1141190158	Treatment/medication code: cipralex 5mg tablet	MR, LDSC
20003_1141190160	Treatment/medication code: vascalpha 5mg m/r tablet	MR
20003_1141190960	Treatment/medication code: tramacet 325mg/37.5mg tablet	MR
20003_1141191044	Treatment/medication code: levothyroxine sodium	MR, LDSC
20003_1141191312	Treatment/medication code: calfovit d3 powder for oral suspension	MR
20003_1141191748	Treatment/medication code: respiratory mometasone	MR
20003_1141192000	Treatment/medication code: dutasteride	MR
20003_1141192004	Treatment/medication code: avodart 500micrograms capsule	MR
20003_1141192248	Treatment/medication code: vardenafil	MR
20003_1141192410	Treatment/medication code: rosuvastatin	MR, LDSC
20003_1141192414	Treatment/medication code: crestor 10mg tablet	MR, LDSC
20003_1141192736	Treatment/medication code: ezetimibe	MR, LDSC
20003_1141192740	Treatment/medication code: ezetrol 10mg tablet	MR
20003_1141192916	Treatment/medication code: rino clenil 50micrograms nasal spray	MR, LDSC
20003_1141193272	Treatment/medication code: testogel 50mg gel 5g sachet	MR, LDSC

20003_1141193282	Treatment/medication code: olmesartan	MR, LDSC
20003_1141193346	Treatment/medication code: olmetec 10mg tablet	MR
20003_1141194224	Treatment/medication code: allergy relief antihistamine 4mg tablet	MR
20003_1141194386	Treatment/medication code: telfast 30 tablet	MR, LDSC
20003_1141194794	Treatment/medication code: bendroflumethiazide	MR, LDSC
20003_1141194800	Treatment/medication code: bendroflumethiazide+potassium 2.5mg/7.7mmol m/r tablet	MR
20003_1141195044	Treatment/medication code: hydroxycarbamide	MR
20003_1141195224	Treatment/medication code: formoterol	MR
20003_1141195232	Treatment/medication code: budesonide+formoterol	MR
20003_1141199858	Treatment/medication code: cardioplen xl 5mg m/r tablet	MR
20003_1141200004	Treatment/medication code: pregabalin	MR, LDSC
20003_1141200072	Treatment/medication code: lyrica 25mg capsule	MR
20003_1141200108	Treatment/medication code: cymalon cranberry 1.5g/5ml liquid	MR, LDSC
20003_1141200322	Treatment/medication code: solifenacin	MR, LDSC
20003_1141200384	Treatment/medication code: vesicare 5mg tablet	MR
20003_1141200564	Treatment/medication code: duloxetine	MR, LDSC
20003_1141200708	Treatment/medication code: strontium product	MR, LDSC
20003_1141200768	Treatment/medication code: protelos 2g sachets	MR, LDSC
20003_1141202030	Treatment/medication code: estradot 25micrograms patch	MR, LDSC
20003_1187	Treatment/medication code: chondroitin product	MR, LDSC
20003_1189	Treatment/medication code: co-enzyme q10/ubiquinone/bio-quinone/coenzyme q10	MR, LDSC
20003_1193	Treatment/medication code: omega-3/fish oil supplement	MR, LDSC
20003_1195	Treatment/medication code: vitamin c product_dup	MR
20003_1197	Treatment/medication code: evening primrose oil product	MR
20003_1199	Treatment/medication code: food supplement/plant/herbal extract	MR, LDSC
20003_1201	Treatment/medication code: st john's wort/hypericum [ctsu]	MR, LDSC
20003_1203	Treatment/medication code: aloe vera product	MR
20003_1205	Treatment/medication code: saw palmetto product	MR
20003_2038459704	Treatment/medication code: carbamazepine	MR, LDSC
20003_2038459814	Treatment/medication code: digoxin	MR, LDSC

20003_2038460068	Treatment/medication code: phenobarbitone	MR, LDSC
20003_2038460076	Treatment/medication code: phenytoin	MR, LDSC
20003_2038460150	Treatment/medication code: paracetamol	MR, LDSC
20003_99999	Treatment/medication code: Free-text entry, unable to be coded	MR, LDSC
20107_1	Illnesses of father: Heart disease	MR, LDSC
20107_10	Illnesses of father: Alzheimer's disease/dementia	MR, LDSC
20107_100	Illnesses of father: None of the above (group 1)	MR, LDSC
20107_101	Illnesses of father: None of the above (group 2)	MR, LDSC
20107_11	Illnesses of father: Parkinson's disease	MR, LDSC
20107_12	Illnesses of father: Severe depression	MR, LDSC
20107_13	Illnesses of father: Prostate cancer	MR, LDSC
20107_2	Illnesses of father: Stroke	MR, LDSC
20107_3	Illnesses of father: Lung cancer	MR, LDSC
20107_4	Illnesses of father: Bowel cancer	MR, LDSC
20107_6	Illnesses of father: Chronic bronchitis/emphysema	MR, LDSC
20107_8	Illnesses of father: High blood pressure	MR, LDSC
20107_9	Illnesses of father: Diabetes	MR, LDSC
20110_1	Illnesses of mother: Heart disease	MR, LDSC
20110_10	Illnesses of mother: Alzheimer's disease/dementia	MR, LDSC
20110_100	Illnesses of mother: None of the above (group 1)	MR, LDSC
20110_101	Illnesses of mother: None of the above (group 2)	MR, LDSC
20110_11	Illnesses of mother: Parkinson's disease	MR, LDSC
20110_12	Illnesses of mother: Severe depression	MR, LDSC
20110_2	Illnesses of mother: Stroke	MR, LDSC
20110_3	Illnesses of mother: Lung cancer	MR, LDSC
20110_4	Illnesses of mother: Bowel cancer	MR, LDSC
20110_5	Illnesses of mother: Breast cancer	MR, LDSC
20110_6	Illnesses of mother: Chronic bronchitis/emphysema	MR, LDSC
20110_8	Illnesses of mother: High blood pressure	MR, LDSC
20110_9	Illnesses of mother: Diabetes	MR, LDSC

20111_1	Illnesses of siblings: Heart disease	MR, LDSC
20111_10	Illnesses of siblings: Alzheimer's disease/dementia	MR
20111_100	Illnesses of siblings: None of the above (group 1)	MR, LDSC
20111_101	Illnesses of siblings: None of the above (group 2)	MR, LDSC
20111_11	Illnesses of siblings: Parkinson's disease	MR, LDSC
20111_12	Illnesses of siblings: Severe depression	MR, LDSC
20111_13	Illnesses of siblings: Prostate cancer	MR, LDSC
20111_2	Illnesses of siblings: Stroke	MR, LDSC
20111_3	Illnesses of siblings: Lung cancer	MR, LDSC
20111_4	Illnesses of siblings: Bowel cancer	MR, LDSC
20111_5	Illnesses of siblings: Breast cancer	MR, LDSC
20111_6	Illnesses of siblings: Chronic bronchitis/emphysema	MR, LDSC
20111_8	Illnesses of siblings: High blood pressure	MR, LDSC
20111_9	Illnesses of siblings: Diabetes	MR, LDSC
20116_1	Smoking status: Previous	MR, LDSC
20116_2	Smoking status: Current	MR, LDSC
20117_0	Alcohol drinker status: Never	MR, LDSC
20117_1	Alcohol drinker status: Previous	MR, LDSC
20118_11	Home area population density - urban or rural: Scotland - Large Urban Area	MR, LDSC
20118_12	Home area population density - urban or rural: Scotland - Other Urban Area	MR, LDSC
20118_13	Home area population density - urban or rural: Scotland - Accessible Small Town	MR
20118_16	Home area population density - urban or rural: Scotland - Accessible Rural	MR, LDSC
20118_17	Home area population density - urban or rural: Scotland - Remote Rural	MR, LDSC
20118_3	Home area population density - urban or rural: England/Wales - Village - sparse	MR
20118_6	Home area population density - urban or rural: England/Wales - Town and Fringe - less sparse	MR, LDSC
20118_7	Home area population density - urban or rural: England/Wales - Village - less sparse	MR, LDSC
20118_8	Home area population density - urban or rural: England/Wales - Hamlet and Isolated Dwelling - less sparse	MR, LDSC
20118_9	Home area population density - urban or rural: Postcode not linkable	MR
2247_1	Hearing difficulty/problems: Yes	MR, LDSC
2247_99	Hearing difficulty/problems: I am completely deaf	MR

22506_111	Tobacco smoking: Smokes on most or all days	MR, LDSC
22506_112	Tobacco smoking: Occasionally	MR, LDSC
22506_113	Tobacco smoking: Ex-smoker	MR, LDSC
2395_2	Hair/balding pattern: Pattern 2	MR, LDSC
2395_3	Hair/balding pattern: Pattern 3	MR, LDSC
2395_4	Hair/balding pattern: Pattern 4	MR, LDSC
2664_1	Reason for reducing amount of alcohol drunk: Illness or ill health	MR, LDSC
2664_2	Reason for reducing amount of alcohol drunk: Doctor's advice	MR, LDSC
2664_3	Reason for reducing amount of alcohol drunk: Health precaution	MR, LDSC
2664_4	Reason for reducing amount of alcohol drunk: Financial reasons	MR, LDSC
2877_2	Type of tobacco previously smoked: Hand-rolled cigarettes	MR, LDSC
2877_3	Type of tobacco previously smoked: Cigars or pipes	MR, LDSC
3446_2	Type of tobacco currently smoked: Hand-rolled cigarettes	MR, LDSC
3446_3	Type of tobacco currently smoked: Cigars or pipes	MR, LDSC
3859_1	Reason former drinker stopped drinking alcohol: Illness or ill health	MR
3859_2	Reason former drinker stopped drinking alcohol: Doctor's advice	MR, LDSC
3859_3	Reason former drinker stopped drinking alcohol: Health precaution	MR
3859_4	Reason former drinker stopped drinking alcohol: Financial reasons	MR, LDSC
40001_A419	Underlying (primary) cause of death: ICD10: A41.9 Septicaemia, unspecified	MR
40001_A810	Underlying (primary) cause of death: ICD10: A81.0 Creutzfeldt-Jakob disease	MR, LDSC
40001_C029	Underlying (primary) cause of death: ICD10: C02.9 Tongue, unspecified	MR, LDSC
40001_C109	Underlying (primary) cause of death: ICD10: C10.9 Oropharynx, unspecified	MR, LDSC
40001_C140	Underlying (primary) cause of death: ICD10: C14.0 Pharynx, unspecified	MR, LDSC
40001_C159	Underlying (primary) cause of death: ICD10: C15.9 Oesophagus, unspecified	MR, LDSC
40001_C160	Underlying (primary) cause of death: ICD10: C16.0 Cardia	MR, LDSC
40001_C169	Underlying (primary) cause of death: ICD10: C16.9 Stomach, unspecified	MR, LDSC
40001_C170	Underlying (primary) cause of death: ICD10: C17.0 Duodenum	MR
40001_C180	Underlying (primary) cause of death: ICD10: C18.0 Caecum	MR
40001_C181	Underlying (primary) cause of death: ICD10: C18.1 Appendix	MR
40001_C187	Underlying (primary) cause of death: ICD10: C18.7 Sigmoid colon	MR

40001_C189	Underlying (primary) cause of death: ICD10: C18.9 Colon, unspecified	MR
40001_C19	Underlying (primary) cause of death: ICD10: C19 Malignant neoplasm of rectosigmoid junction	MR
40001_C20	Underlying (primary) cause of death: ICD10: C20 Malignant neoplasm of rectum	MR
40001_C210	Underlying (primary) cause of death: ICD10: C21.0 Anus, unspecified	MR
40001_C220	Underlying (primary) cause of death: ICD10: C22.0 Liver cell carcinoma	MR, LDSC
40001_C221	Underlying (primary) cause of death: ICD10: C22.1 Intrahepatic bile duct carcinoma	MR, LDSC
40001_C23	Underlying (primary) cause of death: ICD10: C23 Malignant neoplasm of gallbladder	MR, LDSC
40001_C259	Underlying (primary) cause of death: ICD10: C25.9 Pancreas, unspecified	MR, LDSC
40001_C260	Underlying (primary) cause of death: ICD10: C26.0 Intestinal tract, part unspecified	MR, LDSC
40001_C269	Underlying (primary) cause of death: ICD10: C26.9 Ill-defined sites within the digestive system	MR
40001_C329	Underlying (primary) cause of death: ICD10: C32.9 Larynx, unspecified	MR, LDSC
40001_C439	Underlying (primary) cause of death: ICD10: C43.9 Malignant melanoma of skin, unspecified	MR
40001_C450	Underlying (primary) cause of death: ICD10: C45.0 Mesothelioma of pleura	MR, LDSC
40001_C459	Underlying (primary) cause of death: ICD10: C45.9 Mesothelioma, unspecified	MR, LDSC
40001_C482	Underlying (primary) cause of death: ICD10: C48.2 Peritoneum, unspecified	MR
40001_C499	Underlying (primary) cause of death: ICD10: C49.9 Connective and soft tissue, unspecified	MR
40001_C509	Underlying (primary) cause of death: ICD10: C50.9 Breast, unspecified	MR
40001_C539	Underlying (primary) cause of death: ICD10: C53.9 Cervix uteri, unspecified	MR, LDSC
40001_C541	Underlying (primary) cause of death: ICD10: C54.1 Endometrium	MR
40001_C55	Underlying (primary) cause of death: ICD10: C55 Malignant neoplasm of uterus, part unspecified	MR
40001_C56	Underlying (primary) cause of death: ICD10: C56 Malignant neoplasm of ovary	MR
40001_C61	Underlying (primary) cause of death: ICD10: C61 Malignant neoplasm of prostate	MR
40001_C64	Underlying (primary) cause of death: ICD10: C64 Malignant neoplasm of kidney, except renal pelvis	MR, LDSC
40001_C66	Underlying (primary) cause of death: ICD10: C66 Malignant neoplasm of ureter	MR, LDSC
40001_C679	Underlying (primary) cause of death: ICD10: C67.9 Bladder, unspecified	MR, LDSC
40001_C689	Underlying (primary) cause of death: ICD10: C68.9 Urinary organ, unspecified	MR
40001_C710	Underlying (primary) cause of death: ICD10: C71.0 Cerebrum, except lobes and ventricles	MR, LDSC
40001_C719	Underlying (primary) cause of death: ICD10: C71.9 Brain, unspecified	MR
40001_C73	Underlying (primary) cause of death: ICD10: C73 Malignant neoplasm of thyroid gland	MR, LDSC

40001_C786	Underlying (primary) cause of death: ICD10: C78.6 Secondary malignant neoplasm of retroperitoneum and peritoneum	MR, LDSC
40001_C80	Underlying (primary) cause of death: ICD10: C80 Malignant neoplasm without specification of site	MR
40001_C800	Underlying (primary) cause of death: ICD10: C80.0 Malignant neoplasm, primary site unknown, so stated	MR
40001_C809	Underlying (primary) cause of death: ICD10: C80.9 Malignant neoplasm, unspecified	MR, LDSC
40001_C819	Underlying (primary) cause of death: ICD10: C81.9 Hodgkin's disease, unspecified	MR
40001_C829	Underlying (primary) cause of death: ICD10: C82.9 Follicular non-Hodgkin's lymphoma, unspecified	MR
40001_C833	Underlying (primary) cause of death: ICD10: C83.3 Large cell (diffuse)	MR, LDSC
40001_C845	Underlying (primary) cause of death: ICD10: C84.5 Other and unspecified T-cell lymphomas	MR
40001_C851	Underlying (primary) cause of death: ICD10: C85.1 B-cell lymphoma, unspecified	MR
40001_C859	Underlying (primary) cause of death: ICD10: C85.9 Non-Hodgkin's lymphoma, unspecified type	MR
40001_C900	Underlying (primary) cause of death: ICD10: C90.0 Multiple myeloma	MR, LDSC
40001_C910	Underlying (primary) cause of death: ICD10: C91.0 Acute lymphoblastic leukaemia	MR, LDSC
40001_C911	Underlying (primary) cause of death: ICD10: C91.1 Chronic lymphocytic leukaemia	MR, LDSC
40001_C920	Underlying (primary) cause of death: ICD10: C92.0 Acute myeloid leukaemia	MR, LDSC
40001_C921	Underlying (primary) cause of death: ICD10: C92.1 Chronic myeloid leukaemia	MR
40001_C97	Underlying (primary) cause of death: ICD10: C97 Malignant neoplasms of independent (primary) multiple sites	MR
40001_D432	Underlying (primary) cause of death: ICD10: D43.2 Brain, unspecified	MR
40001_D469	Underlying (primary) cause of death: ICD10: D46.9 Myelodysplastic syndrome, unspecified	MR
40001_E149	Underlying (primary) cause of death: ICD10: E14.9 Without complications	MR
40001_E854	Underlying (primary) cause of death: ICD10: E85.4 Organ-limited amyloidosis	MR, LDSC
40001_F019	Underlying (primary) cause of death: ICD10: F01.9 Vascular dementia, unspecified	MR
40001_F03	Underlying (primary) cause of death: ICD10: F03 Unspecified dementia	MR, LDSC
40001_G122	Underlying (primary) cause of death: ICD10: G12.2 Motor neuron disease	MR
40001_G20	Underlying (primary) cause of death: ICD10: G20 Parkinson's disease	MR, LDSC
40001_G309	Underlying (primary) cause of death: ICD10: G30.9 Alzheimer's disease, unspecified	MR
40001_G318	Underlying (primary) cause of death: ICD10: G31.8 Other specified degenerative diseases of nervous system	MR, LDSC
40001_G35	Underlying (primary) cause of death: ICD10: G35 Multiple sclerosis	MR, LDSC
40001_G409	Underlying (primary) cause of death: ICD10: G40.9 Epilepsy, unspecified	MR
40001_G903	Underlying (primary) cause of death: ICD10: G90.3 Multisystem degeneration	MR, LDSC

40001_I110	Underlying (primary) cause of death: ICD10: I11.0 Hypertensive heart disease with (congestive) heart failure	MR, LDSC
40001_I119	Underlying (primary) cause of death: ICD10: I11.9 Hypertensive heart disease without (congestive) heart failure	MR
40001_I219	Underlying (primary) cause of death: ICD10: I21.9 Acute myocardial infarction, unspecified	MR
40001_I251	Underlying (primary) cause of death: ICD10: I25.1 Atherosclerotic heart disease	MR
40001_I255	Underlying (primary) cause of death: ICD10: I25.5 Ischaemic cardiomyopathy	MR
40001_I259	Underlying (primary) cause of death: ICD10: I25.9 Chronic ischaemic heart disease, unspecified	MR
40001_I269	Underlying (primary) cause of death: ICD10: I26.9 Pulmonary embolism without mention of acute cor pulmonale	MR
40001_I330	Underlying (primary) cause of death: ICD10: I33.0 Acute and subacute infective endocarditis	MR
40001_I350	Underlying (primary) cause of death: ICD10: I35.0 Aortic (valve) stenosis	MR
40001_I359	Underlying (primary) cause of death: ICD10: I35.9 Aortic valve disorder, unspecified	MR
40001_I38	Underlying (primary) cause of death: ICD10: I38 Endocarditis, valve unspecified	MR
40001_I420	Underlying (primary) cause of death: ICD10: I42.0 Dilated cardiomyopathy	MR, LDSC
40001_I429	Underlying (primary) cause of death: ICD10: I42.9 Cardiomyopathy, unspecified	MR, LDSC
40001_I48	Underlying (primary) cause of death: ICD10: I48 Atrial fibrillation and flutter	MR
40001_I500	Underlying (primary) cause of death: ICD10: I50.0 Congestive heart failure	MR
40001_I509	Underlying (primary) cause of death: ICD10: I50.9 Heart failure, unspecified	MR, LDSC
40001_I517	Underlying (primary) cause of death: ICD10: I51.7 Cardiomegaly	MR, LDSC
40001_I607	Underlying (primary) cause of death: ICD10: I60.7 Subarachnoid haemorrhage from intracranial artery, unspecified	MR
40001_I609	Underlying (primary) cause of death: ICD10: I60.9 Subarachnoid haemorrhage, unspecified	MR
40001_I619	Underlying (primary) cause of death: ICD10: I61.9 Intracerebral haemorrhage, unspecified	MR, LDSC
40001_I629	Underlying (primary) cause of death: ICD10: I62.9 Intracranial haemorrhage (nontraumatic), unspecified	MR, LDSC
40001_I639	Underlying (primary) cause of death: ICD10: I63.9 Cerebral infarction, unspecified	MR
40001_I64	Underlying (primary) cause of death: ICD10: I64 Stroke, not specified as haemorrhage or infarction	MR
40001_I679	Underlying (primary) cause of death: ICD10: I67.9 Cerebrovascular disease, unspecified	MR
40001_I710	Underlying (primary) cause of death: ICD10: I71.0 Dissection of aorta [any part]	MR, LDSC
40001_I711	Underlying (primary) cause of death: ICD10: I71.1 Thoracic aortic aneurysm, ruptured	MR
40001_I713	Underlying (primary) cause of death: ICD10: I71.3 Abdominal aortic aneurysm, ruptured	MR
40001_I739	Underlying (primary) cause of death: ICD10: I73.9 Peripheral vascular disease, unspecified	MR, LDSC
40001_I802	Underlying (primary) cause of death: ICD10: I80.2 Phlebitis and thrombophlebitis of other deep vessels of lower extremities	MR

40001_J180	Underlying (primary) cause of death: ICD10: J18.0 Bronchopneumonia, unspecified	MR
40001_J181	Underlying (primary) cause of death: ICD10: J18.1 Lobar pneumonia, unspecified	MR, LDSC
40001_J189	Underlying (primary) cause of death: ICD10: J18.9 Pneumonia, unspecified	MR
40001_J439	Underlying (primary) cause of death: ICD10: J43.9 Emphysema, unspecified	MR, LDSC
40001_J440	Underlying (primary) cause of death: ICD10: J44.0 Chronic obstructive pulmonary disease with acute lower respiratory infection	MR
40001_J441	Underlying (primary) cause of death: ICD10: J44.1 Chronic obstructive pulmonary disease with acute exacerbation, unspecified	MR
40001_J449	Underlying (primary) cause of death: ICD10: J44.9 Chronic obstructive pulmonary disease, unspecified	MR, LDSC
40001_J459	Underlying (primary) cause of death: ICD10: J45.9 Asthma, unspecified	MR, LDSC
40001_J47	Underlying (primary) cause of death: ICD10: J47 Bronchiectasis	MR
40001_J690	Underlying (primary) cause of death: ICD10: J69.0 Pneumonitis due to food and vomit	MR
40001_J841	Underlying (primary) cause of death: ICD10: J84.1 Other interstitial pulmonary diseases with fibrosis	MR, LDSC
40001_J849	Underlying (primary) cause of death: ICD10: J84.9 Interstitial pulmonary disease, unspecified	MR
40001_J988	Underlying (primary) cause of death: ICD10: J98.8 Other specified respiratory disorders	MR
40001_K264	Underlying (primary) cause of death: ICD10: K26.4 Chronic or unspecified with haemorrhage	MR
40001_K550	Underlying (primary) cause of death: ICD10: K55.0 Acute vascular disorders of intestine	MR
40001_K559	Underlying (primary) cause of death: ICD10: K55.9 Vascular disorder of intestine, unspecified	MR, LDSC
40001_K566	Underlying (primary) cause of death: ICD10: K56.6 Other and unspecified intestinal obstruction	MR
40001_K578	Underlying (primary) cause of death: ICD10: K57.8 Diverticular disease of intestine, part unspecified, with perforation and abscess	MR
40001_K631	Underlying (primary) cause of death: ICD10: K63.1 Perforation of intestine (nontraumatic)	MR, LDSC
40001_K701	Underlying (primary) cause of death: ICD10: K70.1 Alcoholic hepatitis	MR, LDSC
40001_K703	Underlying (primary) cause of death: ICD10: K70.3 Alcoholic cirrhosis of liver	MR, LDSC
40001_K704	Underlying (primary) cause of death: ICD10: K70.4 Alcoholic hepatic failure	MR, LDSC
40001_K709	Underlying (primary) cause of death: ICD10: K70.9 Alcoholic liver disease, unspecified	MR
40001_K746	Underlying (primary) cause of death: ICD10: K74.6 Other and unspecified cirrhosis of liver	MR, LDSC
40001_K760	Underlying (primary) cause of death: ICD10: K76.0 Fatty (change of) liver, not elsewhere classified	MR, LDSC
40001_K802	Underlying (primary) cause of death: ICD10: K80.2 Calculus of gallbladder without cholecystitis	MR
40001_K830	Underlying (primary) cause of death: ICD10: K83.0 Cholangitis	MR
40001_K859	Underlying (primary) cause of death: ICD10: K85.9 Acute pancreatitis, unspecified	MR

40001_K922	Underlying (primary) cause of death: ICD10: K92.2 Gastro-intestinal haemorrhage, unspecified	MR
40001_M069	Underlying (primary) cause of death: ICD10: M06.9 Rheumatoid arthritis, unspecified	MR, LDSC
40001_N390	Underlying (primary) cause of death: ICD10: N39.0 Urinary tract infection, site not specified	MR, LDSC
40001_R99	Underlying (primary) cause of death: ICD10: R99 Other ill-defined and unspecified causes of mortality	MR
40001_U509	Underlying (primary) cause of death: ICD10: U50.9 Inquest Adjourned Death	MR
40001_V499	Underlying (primary) cause of death: ICD10: V49.9 Car occupant [any] injured in unspecified traffic accident	MR
40001_W100	Underlying (primary) cause of death: ICD10: W10.0 Home	MR
40001_W190	Underlying (primary) cause of death: ICD10: W19.0 Home	MR
40001_X420	Underlying (primary) cause of death: ICD10: X42.0 Home	MR, LDSC
40001_X590	Underlying (primary) cause of death: ICD10: X59.0 Home	MR
40001_X599	Underlying (primary) cause of death: ICD10: X59.9 Unspecified place	MR
40001_X700	Underlying (primary) cause of death: ICD10: X70.0 Home	MR
40001_X708	Underlying (primary) cause of death: ICD10: X70.8 Other specified place	MR, LDSC
40001_X780	Underlying (primary) cause of death: ICD10: X78.0 Home	MR
4803_11	Tinnitus: Yes, now most or all of the time	MR, LDSC
4803_12	Tinnitus: Yes, now a lot of the time	MR, LDSC
4803_13	Tinnitus: Yes, now some of the time	MR, LDSC
4803_14	Tinnitus: Yes, but not now, but have in the past	MR, LDSC
5441_1	Which eye(s) are affected by cataract: Right eye	MR
5441_2	Which eye(s) are affected by cataract: Left eye	MR
5540_1	Surgery/amputation of toe or leg: Yes, toes	MR, LDSC
5540_2	Surgery/amputation of toe or leg: Yes, leg below the knee	MR
5540_3	Surgery/amputation of toe or leg: Yes, leg above the knee	MR
5610_1	Which eye(s) affected by presbyopia: Right eye	MR, LDSC
5610_2	Which eye(s) affected by presbyopia: Left eye	MR
5832_1	Which eye(s) affected by hypermetropia (long sight): Right eye	MR, LDSC
5832_2	Which eye(s) affected by hypermetropia (long sight): Left eye	MR
5843_1	Which eye(s) affected by myopia (short sight): Right eye	MR, LDSC
5843_2	Which eye(s) affected by myopia (short sight): Left eye	MR
5855_1	Which eye(s) affected by astigmatism: Right eye	MR, LDSC

5855_2	Which eye(s) affected by astigmatism: Left eye	MR, LDSC
6138_1	Qualifications: College or University degree	MR, LDSC
6138_100	Qualifications: None of the above	MR, LDSC
6138_2	Qualifications: A levels/AS levels or equivalent	MR, LDSC
6138_3	Qualifications: O levels/GCSEs or equivalent	MR, LDSC
6138_4	Qualifications: CSEs or equivalent	MR, LDSC
6138_5	Qualifications: NVQ or HND or HNC or equivalent	MR, LDSC
6138_6	Qualifications: Other professional qualifications eg: nursing, teaching	MR, LDSC
6142_1	Current employment status: In paid employment or self-employed	MR, LDSC
6142_100	Current employment status: None of the above	MR
6142_2	Current employment status: Retired	MR, LDSC
6142_3	Current employment status: Looking after home and/or family	MR, LDSC
6142_4	Current employment status: Unable to work because of sickness or disability	MR, LDSC
6142_5	Current employment status: Unemployed	MR, LDSC
6142_6	Current employment status: Doing unpaid or voluntary work	MR, LDSC
6142_7	Current employment status: Full or part-time student	MR, LDSC
6143_1	Transport type for commuting to job workplace: Car/motor vehicle	MR, LDSC
6143_100	Transport type for commuting to job workplace: None of the above	MR
6143_2	Transport type for commuting to job workplace: Walk	MR, LDSC
6143_3	Transport type for commuting to job workplace: Public transport	MR, LDSC
6143_4	Transport type for commuting to job workplace: Cycle	MR, LDSC
6145_1	Illness, injury, bereavement, stress in last 2 years: Serious illness, injury or assault to yourself	MR, LDSC
6145_100	Illness, injury, bereavement, stress in last 2 years: None of the above	MR, LDSC
6145_2	Illness, injury, bereavement, stress in last 2 years: Serious illness, injury or assault of a close relative	MR, LDSC
6145_3	Illness, injury, bereavement, stress in last 2 years: Death of a close relative	MR, LDSC
6145_4	Illness, injury, bereavement, stress in last 2 years: Death of a spouse or partner	MR, LDSC
6145_5	Illness, injury, bereavement, stress in last 2 years: Marital separation/divorce	MR, LDSC
6145_6	Illness, injury, bereavement, stress in last 2 years: Financial difficulties	MR, LDSC
6147_1	Reason for glasses/contact lenses: For short-sightedness, i.e. only or mainly for distance viewing such as driving, cinema etc (called 'myopia')	MR, LDSC

6147_2	Reason for glasses/contact lenses: For long-sightedness, i.e. for distance and near, but particularly for near tasks like reading (called 'hypermetropia')	MR, LDSC
6147_3	Reason for glasses/contact lenses: For just reading/near work as you are getting older (called 'presbyopia')	MR, LDSC
6147_4	Reason for glasses/contact lenses: For 'astigmatism'	MR, LDSC
6147_5	Reason for glasses/contact lenses: For a 'squint' or 'turn' in an eye since childhood (called 'strabismus')	MR, LDSC
6147_6	Reason for glasses/contact lenses: For a 'lazy' eye or an eye with poor vision since childhood (called 'amblyopia')	MR, LDSC
6147_7	Reason for glasses/contact lenses: Other eye condition	MR, LDSC
6148_1	Eye problems/disorders: Diabetes related eye disease	MR, LDSC
6148_100	Eye problems/disorders: None of the above	MR, LDSC
6148_2	Eye problems/disorders: Glaucoma	MR, LDSC
6148_3	Eye problems/disorders: Injury or trauma resulting in loss of vision	MR, LDSC
6148_4	Eye problems/disorders: Cataract	MR, LDSC
6148_5	Eye problems/disorders: Macular degeneration	MR, LDSC
6148_6	Eye problems/disorders: Other serious eye condition	MR, LDSC
6149_1	Mouth/teeth dental problems: Mouth ulcers	MR, LDSC
6149_100	Mouth/teeth dental problems: None of the above	MR, LDSC
6149_2	Mouth/teeth dental problems: Painful gums	MR, LDSC
6149_3	Mouth/teeth dental problems: Bleeding gums	MR, LDSC
6149_4	Mouth/teeth dental problems: Loose teeth	MR, LDSC
6149_5	Mouth/teeth dental problems: Toothache	MR, LDSC
6149_6	Mouth/teeth dental problems: Dentures	MR, LDSC
6150_1	Vascular/heart problems diagnosed by doctor: Heart attack	MR, LDSC
6150_100	Vascular/heart problems diagnosed by doctor: None of the above	MR, LDSC
6150_2	Vascular/heart problems diagnosed by doctor: Angina	MR, LDSC
6150_3	Vascular/heart problems diagnosed by doctor: Stroke	MR, LDSC
6150_4	Vascular/heart problems diagnosed by doctor: High blood pressure	MR, LDSC
6151_1	Fractured bone site(s): Ankle	MR, LDSC
6151_2	Fractured bone site(s): Leg	MR, LDSC
6151_3	Fractured bone site(s): Hip	MR, LDSC
6151_4	Fractured bone site(s): Spine	MR, LDSC

6151_5	Fractured bone site(s): Wrist	MR, LDSC
6151_6	Fractured bone site(s): Arm	MR, LDSC
6151_7	Fractured bone site(s): Other bones	MR, LDSC
6152_100	Blood clot, DVT, bronchitis, emphysema, asthma, rhinitis, eczema, allergy diagnosed by doctor: None of the above	MR, LDSC
6152_5	Blood clot, DVT, bronchitis, emphysema, asthma, rhinitis, eczema, allergy diagnosed by doctor: Blood clot in the leg (DVT)	MR, LDSC
6152_6	Blood clot, DVT, bronchitis, emphysema, asthma, rhinitis, eczema, allergy diagnosed by doctor: Emphysema/chronic bronchitis	MR, LDSC
6152_7	Blood clot, DVT, bronchitis, emphysema, asthma, rhinitis, eczema, allergy diagnosed by doctor: Blood clot in the lung	MR, LDSC
6152_8	Blood clot, DVT, bronchitis, emphysema, asthma, rhinitis, eczema, allergy diagnosed by doctor: Asthma	MR, LDSC
6152_9	Blood clot, DVT, bronchitis, emphysema, asthma, rhinitis, eczema, allergy diagnosed by doctor: Hayfever, allergic rhinitis or eczema	MR, LDSC
6153_1	Medication for cholesterol, blood pressure, diabetes, or take exogenous hormones: Cholesterol lowering medication	MR, LDSC
6153_100	Medication for cholesterol, blood pressure, diabetes, or take exogenous hormones: None of the above	MR, LDSC
6153_2	Medication for cholesterol, blood pressure, diabetes, or take exogenous hormones: Blood pressure medication	MR, LDSC
6153_3	Medication for cholesterol, blood pressure, diabetes, or take exogenous hormones: Insulin	MR, LDSC
6153_4	Medication for cholesterol, blood pressure, diabetes, or take exogenous hormones: Hormone replacement therapy	MR, LDSC
6153_5	Medication for cholesterol, blood pressure, diabetes, or take exogenous hormones: Oral contraceptive pill or minipill	MR, LDSC
6154_1	Medication for pain relief, constipation, heartburn: Aspirin	MR, LDSC
6154_100	Medication for pain relief, constipation, heartburn: None of the above	MR, LDSC
6154_2	Medication for pain relief, constipation, heartburn: Ibuprofen (e.g. Nurofen)	MR, LDSC
6154_3	Medication for pain relief, constipation, heartburn: Paracetamol	MR, LDSC
6154_4	Medication for pain relief, constipation, heartburn: Ranitidine (e.g. Zantac)	MR, LDSC
6154_5	Medication for pain relief, constipation, heartburn: Omeprazole (e.g. Zanprol)	MR, LDSC
6154_6	Medication for pain relief, constipation, heartburn: Laxatives (e.g. Dulcolax, Senokot)	MR, LDSC
6155_1	Vitamin and mineral supplements: Vitamin A	MR, LDSC
6155_100	Vitamin and mineral supplements: None of the above	MR, LDSC
6155_2	Vitamin and mineral supplements: Vitamin B	MR, LDSC
6155_3	Vitamin and mineral supplements: Vitamin C	MR, LDSC
6155_4	Vitamin and mineral supplements: Vitamin D	MR, LDSC

6155_5	Vitamin and mineral supplements: Vitamin E	MR, LDSC
6155_6	Vitamin and mineral supplements: Folic acid or Folate (Vit B9)	MR, LDSC
6155_7	Vitamin and mineral supplements: Multivitamins +/- minerals	MR, LDSC
6156_100	Manic/hyper symptoms: None of the above	MR, LDSC
6156_11	Manic/hyper symptoms: I was more active than usual	MR, LDSC
6156_12	Manic/hyper symptoms: I was more talkative than usual	MR, LDSC
6156_13	Manic/hyper symptoms: I needed less sleep than usual	MR, LDSC
6156_14	Manic/hyper symptoms: I was more creative or had more ideas than usual	MR, LDSC
6156_15	Manic/hyper symptoms: All of the above	MR, LDSC
6157_1	Why stopped smoking: Illness or ill health	MR, LDSC
6157_100	Why stopped smoking: None of the above	MR, LDSC
6157_2	Why stopped smoking: Doctor's advice	MR, LDSC
6157_3	Why stopped smoking: Health precaution	MR, LDSC
6157_4	Why stopped smoking: Financial reasons	MR, LDSC
6158_1	Why reduced smoking: Illness or ill health	MR, LDSC
6158_100	Why reduced smoking: None of the above	MR, LDSC
6158_2	Why reduced smoking: Doctor's advice	MR, LDSC
6158_3	Why reduced smoking: Health precaution	MR, LDSC
6158_4	Why reduced smoking: Financial reasons	MR, LDSC
6159_1	Pain type(s) experienced in last month: Headache	MR, LDSC
6159_100	Pain type(s) experienced in last month: None of the above	MR, LDSC
6159_2	Pain type(s) experienced in last month: Facial pain	MR, LDSC
6159_3	Pain type(s) experienced in last month: Neck or shoulder pain	MR, LDSC
6159_4	Pain type(s) experienced in last month: Back pain	MR, LDSC
6159_5	Pain type(s) experienced in last month: Stomach or abdominal pain	MR, LDSC
6159_6	Pain type(s) experienced in last month: Hip pain	MR, LDSC
6159_7	Pain type(s) experienced in last month: Knee pain	MR, LDSC
6159_8	Pain type(s) experienced in last month: Pain all over the body	MR, LDSC
6162_1	Types of transport used (excluding work): Car/motor vehicle	MR, LDSC
6162_100	Types of transport used (excluding work): None of the above	MR, LDSC

6162_2	Types of transport used (excluding work): Walk	MR, LDSC
6162_3	Types of transport used (excluding work): Public transport	MR, LDSC
6162_4	Types of transport used (excluding work): Cycle	MR, LDSC
6164_1	Types of physical activity in last 4 weeks: Walking for pleasure (not as a means of transport)	MR, LDSC
6164_100	Types of physical activity in last 4 weeks: None of the above	MR, LDSC
6164_2	Types of physical activity in last 4 weeks: Other exercises (eg: swimming, cycling, keep fit, bowling)	MR, LDSC
6164_3	Types of physical activity in last 4 weeks: Strenuous sports	MR, LDSC
6164_4	Types of physical activity in last 4 weeks: Light DIY (eg: pruning, watering the lawn)	MR, LDSC
6164_5	Types of physical activity in last 4 weeks: Heavy DIY (eg: weeding, lawn mowing, carpentry, digging)	MR, LDSC
6177_1	Medication for cholesterol, blood pressure or diabetes: Cholesterol lowering medication	MR, LDSC
6177_100	Medication for cholesterol, blood pressure or diabetes: None of the above	MR, LDSC
6177_2	Medication for cholesterol, blood pressure or diabetes: Blood pressure medication	MR, LDSC
6177_3	Medication for cholesterol, blood pressure or diabetes: Insulin	MR, LDSC
6179_1	Mineral and other dietary supplements: Fish oil (including cod liver oil)	MR, LDSC
6179_100	Mineral and other dietary supplements: None of the above	MR, LDSC
6179_2	Mineral and other dietary supplements: Glucosamine	MR, LDSC
6179_3	Mineral and other dietary supplements: Calcium	MR, LDSC
6179_4	Mineral and other dietary supplements: Zinc	MR, LDSC
6179_5	Mineral and other dietary supplements: Iron	MR, LDSC
6179_6	Mineral and other dietary supplements: Selenium	MR, LDSC
A04	Diagnoses - main ICD10: A04 Other bacterial intestinal infections	MR, LDSC
A08	Diagnoses - main ICD10: A08 Viral and other specified intestinal infections	MR
A09	Diagnoses - main ICD10: A09 Diarrhoea and gastro-enteritis of presumed infectious origin	MR
A41	Diagnoses - main ICD10: A41 Other septicaemia	MR
A63	Diagnoses - main ICD10: A63 Other predominantly sexually transmitted diseases, not elsewhere classified	MR
A87	Diagnoses - main ICD10: A87 Viral meningitis	MR, LDSC
B02	Diagnoses - main ICD10: B02 Zoster [herpes zoster]	MR
B07	Diagnoses - main ICD10: B07 Viral warts	MR
B18	Diagnoses - main ICD10: B18 Chronic viral hepatitis	MR
B34	Diagnoses - main ICD10: B34 Viral infection of unspecified site	MR, LDSC

B37	Diagnoses - main ICD10: B37 Candidiasis	MR
B50	Diagnoses - main ICD10: B50 Plasmodium falciparum malaria	MR
C02	Diagnoses - main ICD10: C02 Malignant neoplasm of other and unspecified parts of tongue	MR, LDSC
C09	Diagnoses - main ICD10: C09 Malignant neoplasm of tonsil	MR, LDSC
C15	Diagnoses - main ICD10: C15 Malignant neoplasm of oesophagus	MR, LDSC
C16	Diagnoses - main ICD10: C16 Malignant neoplasm of stomach	MR
C18	Diagnoses - main ICD10: C18 Malignant neoplasm of colon	MR, LDSC
C19	Diagnoses - main ICD10: C19 Malignant neoplasm of rectosigmoid junction	MR, LDSC
C20	Diagnoses - main ICD10: C20 Malignant neoplasm of rectum	MR, LDSC
C21	Diagnoses - main ICD10: C21 Malignant neoplasm of anus and anal canal	MR, LDSC
C22	Diagnoses - main ICD10: C22 Malignant neoplasm of liver and intrahepatic bile ducts	MR, LDSC
C25	Diagnoses - main ICD10: C25 Malignant neoplasm of pancreas	MR, LDSC
C32	Diagnoses - main ICD10: C32 Malignant neoplasm of larynx	MR
C34	Diagnoses - main ICD10: C34 Malignant neoplasm of bronchus and lung	MR, LDSC
C43	Diagnoses - main ICD10: C43 Malignant melanoma of skin	MR, LDSC
C44	Diagnoses - main ICD10: C44 Other malignant neoplasms of skin	MR, LDSC
C45	Diagnoses - main ICD10: C45 Mesothelioma	MR
C49	Diagnoses - main ICD10: C49 Malignant neoplasm of other connective and soft tissue	MR
C50	Diagnoses - main ICD10: C50 Malignant neoplasm of breast	MR, LDSC
C53	Diagnoses - main ICD10: C53 Malignant neoplasm of cervix uteri	MR, LDSC
C54	Diagnoses - main ICD10: C54 Malignant neoplasm of corpus uteri	MR
C56	Diagnoses - main ICD10: C56 Malignant neoplasm of ovary	MR
C61	Diagnoses - main ICD10: C61 Malignant neoplasm of prostate	MR, LDSC
C62	Diagnoses - main ICD10: C62 Malignant neoplasm of testis	MR, LDSC
C64	Diagnoses - main ICD10: C64 Malignant neoplasm of kidney, except renal pelvis	MR
C67	Diagnoses - main ICD10: C67 Malignant neoplasm of bladder	MR, LDSC
C69	Diagnoses - main ICD10: C69 Malignant neoplasm of eye and adnexa	MR, LDSC
C71	Diagnoses - main ICD10: C71 Malignant neoplasm of brain	MR
C73	Diagnoses - main ICD10: C73 Malignant neoplasm of thyroid gland	MR
C77	Diagnoses - main ICD10: C77 Secondary and unspecified malignant neoplasm of lymph nodes	MR

C78	Diagnoses - main ICD10: C78 Secondary malignant neoplasm of respiratory and digestive organs	MR, LDSC
C79	Diagnoses - main ICD10: C79 Secondary malignant neoplasm of other sites	MR
C80	Diagnoses - main ICD10: C80 Malignant neoplasm without specification of site	MR, LDSC
C81	Diagnoses - main ICD10: C81 Hodgkin's disease	MR
C82	Diagnoses - main ICD10: C82 Follicular [nodular] non-Hodgkin's lymphoma	MR, LDSC
C83	Diagnoses - main ICD10: C83 Diffuse non-Hodgkin's lymphoma	MR
C85	Diagnoses - main ICD10: C85 Other and unspecified types of non-Hodgkin's lymphoma	MR
C90	Diagnoses - main ICD10: C90 Multiple myeloma and malignant plasma cell neoplasms	MR
C91	Diagnoses - main ICD10: C91 Lymphoid leukaemia	MR
C92	Diagnoses - main ICD10: C92 Myeloid leukaemia	MR, LDSC
D03	Diagnoses - main ICD10: D03 Melanoma in situ	MR
D04	Diagnoses - main ICD10: D04 Carcinoma in situ of skin	MR
D05	Diagnoses - main ICD10: D05 Carcinoma in situ of breast	MR, LDSC
D06	Diagnoses - main ICD10: D06 Carcinoma in situ of cervix uteri	MR
D07	Diagnoses - main ICD10: D07 Carcinoma in situ of other and unspecified genital organs	MR
D09	Diagnoses - main ICD10: D09 Carcinoma in situ of other and unspecified sites	MR
D10	Diagnoses - main ICD10: D10 Benign neoplasm of mouth and pharynx	MR
D11	Diagnoses - main ICD10: D11 Benign neoplasm of major salivary glands	MR
D12	Diagnoses - main ICD10: D12 Benign neoplasm of colon, rectum, anus and anal canal	MR, LDSC
D13	Diagnoses - main ICD10: D13 Benign neoplasm of other and ill-defined parts of digestive system	MR
D14	Diagnoses - main ICD10: D14 Benign neoplasm of middle ear and respiratory system	MR
D16	Diagnoses - main ICD10: D16 Benign neoplasm of bone and articular cartilage	MR, LDSC
D17	Diagnoses - main ICD10: D17 Benign lipomatous neoplasm	MR, LDSC
D18	Diagnoses - main ICD10: D18 Haemangioma and lymphangioma, any site	MR, LDSC
D21	Diagnoses - main ICD10: D21 Other benign neoplasms of connective and other soft tissue	MR, LDSC
D22	Diagnoses - main ICD10: D22 Melanocytic naevi	MR, LDSC
D23	Diagnoses - main ICD10: D23 Other benign neoplasms of skin	MR, LDSC
D24	Diagnoses - main ICD10: D24 Benign neoplasm of breast	MR, LDSC
D25	Diagnoses - main ICD10: D25 Leiomyoma of uterus	MR, LDSC
D26	Diagnoses - main ICD10: D26 Other benign neoplasms of uterus	MR

D27	Diagnoses - main ICD10: D27 Benign neoplasm of ovary	MR, LDSC
D28	Diagnoses - main ICD10: D28 Benign neoplasm of other and unspecified female genital organs	MR, LDSC
D30	Diagnoses - main ICD10: D30 Benign neoplasm of urinary organs	MR
D32	Diagnoses - main ICD10: D32 Benign neoplasm of meninges	MR
D33	Diagnoses - main ICD10: D33 Benign neoplasm of brain and other parts of central nervous system	MR
D34	Diagnoses - main ICD10: D34 Benign neoplasm of thyroid gland	MR
D35	Diagnoses - main ICD10: D35 Benign neoplasm of other and unspecified endocrine glands	MR
D36	Diagnoses - main ICD10: D36 Benign neoplasm of other and unspecified sites	MR, LDSC
D37	Diagnoses - main ICD10: D37 Neoplasm of uncertain or unknown behaviour of oral cavity and digestive organs	MR, LDSC
D38	Diagnoses - main ICD10: D38 Neoplasm of uncertain or unknown behaviour of middle ear and respiratory and intrathoracic organs	MR
D39	Diagnoses - main ICD10: D39 Neoplasm of uncertain or unknown behaviour of female genital organs	MR
D41	Diagnoses - main ICD10: D41 Neoplasm of uncertain or unknown behaviour of urinary organs	MR
D44	Diagnoses - main ICD10: D44 Neoplasm of uncertain or unknown behaviour of endocrine glands	MR
D45	Diagnoses - main ICD10: D45 Polycythaemia vera	MR, LDSC
D46	Diagnoses - main ICD10: D46 Myelodysplastic syndromes	MR, LDSC
D47	Diagnoses - main ICD10: D47 Other neoplasms of uncertain or unknown behaviour of lymphoid, haematopoietic and related tissue	MR, LDSC
D48	Diagnoses - main ICD10: D48 Neoplasm of uncertain or unknown behaviour of other and unspecified sites	MR, LDSC
D50	Diagnoses - main ICD10: D50 Iron deficiency anaemia	MR, LDSC
D61	Diagnoses - main ICD10: D61 Other aplastic anaemias	MR
D64	Diagnoses - main ICD10: D64 Other anaemias	MR, LDSC
D68	Diagnoses - main ICD10: D68 Other coagulation defects	MR
D69	Diagnoses - main ICD10: D69 Purpura and other haemorrhagic conditions	MR, LDSC
D70	Diagnoses - main ICD10: D70 Agranulocytosis	MR, LDSC
D75	Diagnoses - main ICD10: D75 Other diseases of blood and blood-forming organs	MR
D86	Diagnoses - main ICD10: D86 Sarcoidosis	MR, LDSC
D89	Diagnoses - main ICD10: D89 Other disorders involving the immune mechanism, not elsewhere classified	MR
E03	Diagnoses - main ICD10: E03 Other hypothyroidism	MR, LDSC
E04	Diagnoses - main ICD10: E04 Other non-toxic goitre	MR, LDSC
E05	Diagnoses - main ICD10: E05 Thyrotoxicosis [hyperthyroidism]	MR

E07	Diagnoses - main ICD10: E07 Other disorders of thyroid	MR, LDSC
E10	Diagnoses - main ICD10: E10 Insulin-dependent diabetes mellitus	MR, LDSC
E11	Diagnoses - main ICD10: E11 Non-insulin-dependent diabetes mellitus	MR, LDSC
E14	Diagnoses - main ICD10: E14 Unspecified diabetes mellitus	MR
E16	Diagnoses - main ICD10: E16 Other disorders of pancreatic internal secretion	MR, LDSC
E21	Diagnoses - main ICD10: E21 Hyperparathyroidism and other disorders of parathyroid gland	MR
E22	Diagnoses - main ICD10: E22 Hyperfunction of pituitary gland	MR
E23	Diagnoses - main ICD10: E23 Hypofunction and other disorders of pituitary gland	MR, LDSC
E27	Diagnoses - main ICD10: E27 Other disorders of adrenal gland	MR
E66	Diagnoses - main ICD10: E66 Obesity	MR, LDSC
E83	Diagnoses - main ICD10: E83 Disorders of mineral metabolism	MR
E86	Diagnoses - main ICD10: E86 Volume depletion	MR
E87	Diagnoses - main ICD10: E87 Other disorders of fluid, electrolyte and acid-base balance	MR, LDSC
F10	Diagnoses - main ICD10: F10 Mental and behavioural disorders due to use of alcohol	MR, LDSC
F20	Diagnoses - main ICD10: F20 Schizophrenia	MR, LDSC
F23	Diagnoses - main ICD10: F23 Acute and transient psychotic disorders	MR
F25	Diagnoses - main ICD10: F25 Schizoaffective disorders	MR
F30	Diagnoses - main ICD10: F30 Manic episode	MR, LDSC
F31	Diagnoses - main ICD10: F31 Bipolar affective disorder	MR, LDSC
F32	Diagnoses - main ICD10: F32 Depressive episode	MR, LDSC
F33	Diagnoses - main ICD10: F33 Recurrent depressive disorder	MR, LDSC
F41	Diagnoses - main ICD10: F41 Other anxiety disorders	MR
F43	Diagnoses - main ICD10: F43 Reaction to severe stress, and adjustment disorders	MR, LDSC
F45	Diagnoses - main ICD10: F45 Somatoform disorders	MR, LDSC
F52	Diagnoses - main ICD10: F52 Sexual dysfunction, not caused by organic disorder or disease	MR
F60	Diagnoses - main ICD10: F60 Specific personality disorders	MR, LDSC
F99	Diagnoses - main ICD10: F99 Mental disorder, not otherwise specified	MR
G20	Diagnoses - main ICD10: G20 Parkinson's disease	MR
G24	Diagnoses - main ICD10: G24 Dystonia	MR, LDSC
G35	Diagnoses - main ICD10: G35 Multiple sclerosis	MR, LDSC

G37	Diagnoses - main ICD10: G37 Other demyelinating diseases of central nervous system	MR, LDSC
G40	Diagnoses - main ICD10: G40 Epilepsy	MR
G43	Diagnoses - main ICD10: G43 Migraine	MR, LDSC
G44	Diagnoses - main ICD10: G44 Other headache syndromes	MR, LDSC
G45	Diagnoses - main ICD10: G45 Transient cerebral ischaemic attacks and related syndromes	MR
G47	Diagnoses - main ICD10: G47 Sleep disorders	MR, LDSC
G50	Diagnoses - main ICD10: G50 Disorders of trigeminal nerve	MR, LDSC
G51	Diagnoses - main ICD10: G51 Facial nerve disorders	MR, LDSC
G54	Diagnoses - main ICD10: G54 Nerve root and plexus disorders	MR
G56	Diagnoses - main ICD10: G56 Mononeuropathies of upper limb	MR, LDSC
G57	Diagnoses - main ICD10: G57 Mononeuropathies of lower limb	MR, LDSC
G58	Diagnoses - main ICD10: G58 Other mononeuropathies	MR, LDSC
G61	Diagnoses - main ICD10: G61 Inflammatory polyneuropathy	MR, LDSC
G62	Diagnoses - main ICD10: G62 Other polyneuropathies	MR, LDSC
G70	Diagnoses - main ICD10: G70 Myasthenia gravis and other myoneural disorders	MR, LDSC
G81	Diagnoses - main ICD10: G81 Hemiplegia	MR
G82	Diagnoses - main ICD10: G82 Paraplegia and tetraplegia	MR
G93	Diagnoses - main ICD10: G93 Other disorders of brain	MR, LDSC
G95	Diagnoses - main ICD10: G95 Other diseases of spinal cord	MR
H00	Diagnoses - main ICD10: H00 Hordeolum and chalazion	MR, LDSC
H01	Diagnoses - main ICD10: H01 Other inflammation of eyelid	MR
H02	Diagnoses - main ICD10: H02 Other disorders of eyelid	MR, LDSC
H04	Diagnoses - main ICD10: H04 Disorders of lachrymal system	MR
H05	Diagnoses - main ICD10: H05 Disorders of orbit	MR
H11	Diagnoses - main ICD10: H11 Other disorders of conjunctiva	MR, LDSC
H16	Diagnoses - main ICD10: H16 Keratitis	MR
H18	Diagnoses - main ICD10: H18 Other disorders of cornea	MR
H25	Diagnoses - main ICD10: H25 Senile cataract	MR, LDSC
H26	Diagnoses - main ICD10: H26 Other cataract	MR, LDSC
H27	Diagnoses - main ICD10: H27 Other disorders of lens	MR

H33	Diagnoses - main ICD10: H33 Retinal detachments and breaks	MR, LDSC
H34	Diagnoses - main ICD10: H34 Retinal vascular occlusions	MR, LDSC
H35	Diagnoses - main ICD10: H35 Other retinal disorders	MR
H40	Diagnoses - main ICD10: H40 Glaucoma	MR, LDSC
H43	Diagnoses - main ICD10: H43 Disorders of vitreous body	MR, LDSC
H49	Diagnoses - main ICD10: H49 Paralytic strabismus	MR
H50	Diagnoses - main ICD10: H50 Other strabismus	MR
H52	Diagnoses - main ICD10: H52 Disorders of refraction and accommodation	MR
H53	Diagnoses - main ICD10: H53 Visual disturbances	MR, LDSC
H57	Diagnoses - main ICD10: H57 Other disorders of eye and adnexa	MR, LDSC
H59	Diagnoses - main ICD10: H59 Postprocedural disorders of eye and adnexa, not elsewhere classified	MR, LDSC
H60	Diagnoses - main ICD10: H60 Otitis externa	MR, LDSC
H61	Diagnoses - main ICD10: H61 Other disorders of external ear	MR
H65	Diagnoses - main ICD10: H65 Nonsuppurative otitis media	MR, LDSC
H66	Diagnoses - main ICD10: H66 Suppurative and unspecified otitis media	MR
H69	Diagnoses - main ICD10: H69 Other disorders of Eustachian tube	MR
H71	Diagnoses - main ICD10: H71 Cholesteatoma of middle ear	MR, LDSC
H72	Diagnoses - main ICD10: H72 Perforation of tympanic membrane	MR, LDSC
H73	Diagnoses - main ICD10: H73 Other disorders of tympanic membrane	MR
H74	Diagnoses - main ICD10: H74 Other disorders of middle ear and mastoid	MR
H80	Diagnoses - main ICD10: H80 Otosclerosis	MR, LDSC
H81	Diagnoses - main ICD10: H81 Disorders of vestibular function	MR, LDSC
H83	Diagnoses - main ICD10: H83 Other diseases of inner ear	MR
H90	Diagnoses - main ICD10: H90 Conductive and sensorineural hearing loss	MR
H91	Diagnoses - main ICD10: H91 Other hearing loss	MR, LDSC
H92	Diagnoses - main ICD10: H92 Otalgia and effusion of ear	MR
H93	Diagnoses - main ICD10: H93 Other disorders of ear, not elsewhere classified	MR
I05	Diagnoses - main ICD10: I05 Rheumatic mitral valve diseases	MR, LDSC
I08	Diagnoses - main ICD10: I08 Multiple valve diseases	MR
I10	Diagnoses - main ICD10: I10 Essential (primary) hypertension	MR, LDSC

I12	Diagnoses - main ICD10: I12 Hypertensive renal disease	MR, LDSC
I20	Diagnoses - main ICD10: I20 Angina pectoris	MR, LDSC
I21	Diagnoses - main ICD10: I21 Acute myocardial infarction	MR, LDSC
I22	Diagnoses - main ICD10: I22 Subsequent myocardial infarction	MR, LDSC
I24	Diagnoses - main ICD10: I24 Other acute ischaemic heart diseases	MR, LDSC
I25	Diagnoses - main ICD10: I25 Chronic ischaemic heart disease	MR, LDSC
I26	Diagnoses - main ICD10: I26 Pulmonary embolism	MR, LDSC
I30	Diagnoses - main ICD10: I30 Acute pericarditis	MR, LDSC
I31	Diagnoses - main ICD10: I31 Other diseases of pericardium	MR, LDSC
I33	Diagnoses - main ICD10: I33 Acute and subacute endocarditis	MR
I34	Diagnoses - main ICD10: I34 Nonrheumatic mitral valve disorders	MR
I35	Diagnoses - main ICD10: I35 Nonrheumatic aortic valve disorders	MR
I42	Diagnoses - main ICD10: I42 Cardiomyopathy	MR, LDSC
I44	Diagnoses - main ICD10: I44 Atrioventricular and left bundle-branch block	MR
I45	Diagnoses - main ICD10: I45 Other conduction disorders	MR, LDSC
I46	Diagnoses - main ICD10: I46 Cardiac arrest	MR
I47	Diagnoses - main ICD10: I47 Paroxysmal tachycardia	MR, LDSC
I48	Diagnoses - main ICD10: I48 Atrial fibrillation and flutter	MR, LDSC
I49	Diagnoses - main ICD10: I49 Other cardiac arrhythmias	MR, LDSC
I50	Diagnoses - main ICD10: I50 Heart failure	MR, LDSC
I51	Diagnoses - main ICD10: I51 Complications and ill-defined descriptions of heart disease	MR, LDSC
I60	Diagnoses - main ICD10: I60 Subarachnoid haemorrhage	MR
I61	Diagnoses - main ICD10: I61 Intracerebral haemorrhage	MR
I62	Diagnoses - main ICD10: I62 Other nontraumatic intracranial haemorrhage	MR, LDSC
I63	Diagnoses - main ICD10: I63 Cerebral infarction	MR, LDSC
I64	Diagnoses - main ICD10: I64 Stroke, not specified as haemorrhage or infarction	MR
I65	Diagnoses - main ICD10: I65 Occlusion and stenosis of precerebral arteries, not resulting in cerebral infarction	MR, LDSC
I67	Diagnoses - main ICD10: I67 Other cerebrovascular diseases	MR
I70	Diagnoses - main ICD10: I70 Atherosclerosis	MR, LDSC
I71	Diagnoses - main ICD10: I71 Aortic aneurysm and dissection	MR

I72	Diagnoses - main ICD10: I72 Other aneurysm	MR
I73	Diagnoses - main ICD10: I73 Other peripheral vascular diseases	MR, LDSC
I74	Diagnoses - main ICD10: I74 Arterial embolism and thrombosis	MR
I77	Diagnoses - main ICD10: I77 Other disorders of arteries and arterioles	MR
I78	Diagnoses - main ICD10: I78 Diseases of capillaries	MR
I80	Diagnoses - main ICD10: I80 Phlebitis and thrombophlebitis	MR, LDSC
I82	Diagnoses - main ICD10: I82 Other venous embolism and thrombosis	MR
I83	Diagnoses - main ICD10: I83 Varicose veins of lower extremities	MR, LDSC
I84	Diagnoses - main ICD10: I84 Haemorrhoids	MR, LDSC
I85	Diagnoses - main ICD10: I85 Oesophageal varices	MR
I86	Diagnoses - main ICD10: I86 Varicose veins of other sites	MR, LDSC
I87	Diagnoses - main ICD10: I87 Other disorders of veins	MR
I89	Diagnoses - main ICD10: I89 Other non-infective disorders of lymphatic vessels and lymph nodes	MR
I95	Diagnoses - main ICD10: I95 Hypotension	MR, LDSC
J01	Diagnoses - main ICD10: J01 Acute sinusitis	MR, LDSC
J02	Diagnoses - main ICD10: J02 Acute pharyngitis	MR, LDSC
J03	Diagnoses - main ICD10: J03 Acute tonsillitis	MR, LDSC
J06	Diagnoses - main ICD10: J06 Acute upper respiratory infections of multiple and unspecified sites	MR
J13	Diagnoses - main ICD10: J13 Pneumonia due to Streptococcus pneumoniae	MR, LDSC
J15	Diagnoses - main ICD10: J15 Bacterial pneumonia, not elsewhere classified	MR
J18	Diagnoses - main ICD10: J18 Pneumonia, organism unspecified	MR, LDSC
J22	Diagnoses - main ICD10: J22 Unspecified acute lower respiratory infection	MR, LDSC
J31	Diagnoses - main ICD10: J31 Chronic rhinitis, nasopharyngitis and pharyngitis	MR
J32	Diagnoses - main ICD10: J32 Chronic sinusitis	MR
J33	Diagnoses - main ICD10: J33 Nasal polyp	MR, LDSC
J34	Diagnoses - main ICD10: J34 Other disorders of nose and nasal sinuses	MR, LDSC
J35	Diagnoses - main ICD10: J35 Chronic diseases of tonsils and adenoids	MR, LDSC
J36	Diagnoses - main ICD10: J36 Peritonsillar abscess	MR, LDSC
J38	Diagnoses - main ICD10: J38 Diseases of vocal cords and larynx, not elsewhere classified	MR, LDSC
J39	Diagnoses - main ICD10: J39 Other diseases of upper respiratory tract	MR, LDSC

J40	Diagnoses - main ICD10: J40 Bronchitis, not specified as acute or chronic	MR
J43	Diagnoses - main ICD10: J43 Emphysema	MR, LDSC
J44	Diagnoses - main ICD10: J44 Other chronic obstructive pulmonary disease	MR, LDSC
J45	Diagnoses - main ICD10: J45 Asthma	MR, LDSC
J46	Diagnoses - main ICD10: J46 Status asthmaticus	MR, LDSC
J47	Diagnoses - main ICD10: J47 Bronchiectasis	MR, LDSC
J84	Diagnoses - main ICD10: J84 Other interstitial pulmonary diseases	MR, LDSC
J86	Diagnoses - main ICD10: J86 Pyothorax	MR, LDSC
J90	Diagnoses - main ICD10: J90 Pleural effusion, not elsewhere classified	MR, LDSC
J93	Diagnoses - main ICD10: J93 Pneumothorax	MR
J96	Diagnoses - main ICD10: J96 Respiratory failure, not elsewhere classified	MR
J98	Diagnoses - main ICD10: J98 Other respiratory disorders	MR
K00	Diagnoses - main ICD10: K00 Disorders of tooth development and eruption	MR, LDSC
K01	Diagnoses - main ICD10: K01 Embedded and impacted teeth	MR, LDSC
K02	Diagnoses - main ICD10: K02 Dental caries	MR, LDSC
K04	Diagnoses - main ICD10: K04 Diseases of pulp and periapical tissues	MR
K05	Diagnoses - main ICD10: K05 Gingivitis and periodontal diseases	MR, LDSC
K06	Diagnoses - main ICD10: K06 Other disorders of gingiva and edentulous alveolar ridge	MR
K07	Diagnoses - main ICD10: K07 Dentofacial anomalies [including malocclusion]	MR
K08	Diagnoses - main ICD10: K08 Other disorders of teeth and supporting structures	MR, LDSC
K09	Diagnoses - main ICD10: K09 Cysts of oral region, not elsewhere classified	MR, LDSC
K10	Diagnoses - main ICD10: K10 Other diseases of jaws	MR
K11	Diagnoses - main ICD10: K11 Diseases of salivary glands	MR
K12	Diagnoses - main ICD10: K12 Stomatitis and related lesions	MR
K13	Diagnoses - main ICD10: K13 Other diseases of lip and oral mucosa	MR, LDSC
K14	Diagnoses - main ICD10: K14 Diseases of tongue	MR, LDSC
K20	Diagnoses - main ICD10: K20 Oesophagitis	MR, LDSC
K21	Diagnoses - main ICD10: K21 Gastro-oesophageal reflux disease	MR, LDSC
K22	Diagnoses - main ICD10: K22 Other diseases of oesophagus	MR, LDSC
K25	Diagnoses - main ICD10: K25 Gastric ulcer	MR, LDSC

K26	Diagnoses - main ICD10: K26 Duodenal ulcer	MR
K27	Diagnoses - main ICD10: K27 Peptic ulcer, site unspecified	MR
K29	Diagnoses - main ICD10: K29 Gastritis and duodenitis	MR, LDSC
K30	Diagnoses - main ICD10: K30 Dyspepsia	MR, LDSC
K31	Diagnoses - main ICD10: K31 Other diseases of stomach and duodenum	MR
K35	Diagnoses - main ICD10: K35 Acute appendicitis	MR, LDSC
K37	Diagnoses - main ICD10: K37 Unspecified appendicitis	MR
K40	Diagnoses - main ICD10: K40 Inguinal hernia	MR, LDSC
K41	Diagnoses - main ICD10: K41 Femoral hernia	MR, LDSC
K42	Diagnoses - main ICD10: K42 Umbilical hernia	MR, LDSC
K43	Diagnoses - main ICD10: K43 Ventral hernia	MR, LDSC
K44	Diagnoses - main ICD10: K44 Diaphragmatic hernia	MR, LDSC
K46	Diagnoses - main ICD10: K46 Unspecified abdominal hernia	MR
K50	Diagnoses - main ICD10: K50 Crohn's disease [regional enteritis]	MR, LDSC
K51	Diagnoses - main ICD10: K51 Ulcerative colitis	MR, LDSC
K52	Diagnoses - main ICD10: K52 Other non-infective gastro-enteritis and colitis	MR, LDSC
K55	Diagnoses - main ICD10: K55 Vascular disorders of intestine	MR
K56	Diagnoses - main ICD10: K56 Paralytic ileus and intestinal obstruction without hernia	MR
K57	Diagnoses - main ICD10: K57 Diverticular disease of intestine	MR, LDSC
K58	Diagnoses - main ICD10: K58 Irritable bowel syndrome	MR, LDSC
K59	Diagnoses - main ICD10: K59 Other functional intestinal disorders	MR, LDSC
K60	Diagnoses - main ICD10: K60 Fissure and fistula of anal and rectal regions	MR, LDSC
K61	Diagnoses - main ICD10: K61 Abscess of anal and rectal regions	MR, LDSC
K62	Diagnoses - main ICD10: K62 Other diseases of anus and rectum	MR, LDSC
K63	Diagnoses - main ICD10: K63 Other diseases of intestine	MR, LDSC
K65	Diagnoses - main ICD10: K65 Peritonitis	MR, LDSC
K66	Diagnoses - main ICD10: K66 Other disorders of peritoneum	MR
K70	Diagnoses - main ICD10: K70 Alcoholic liver disease	MR
K74	Diagnoses - main ICD10: K74 Fibrosis and cirrhosis of liver	MR
K75	Diagnoses - main ICD10: K75 Other inflammatory liver diseases	MR

K76	Diagnoses - main ICD10: K76 Other diseases of liver	MR, LDSC
K80	Diagnoses - main ICD10: K80 Cholelithiasis	MR, LDSC
K81	Diagnoses - main ICD10: K81 Cholecystitis	MR, LDSC
K82	Diagnoses - main ICD10: K82 Other diseases of gallbladder	MR
K83	Diagnoses - main ICD10: K83 Other diseases of biliary tract	MR
K85	Diagnoses - main ICD10: K85 Acute pancreatitis	MR, LDSC
K86	Diagnoses - main ICD10: K86 Other diseases of pancreas	MR, LDSC
K90	Diagnoses - main ICD10: K90 Intestinal malabsorption	MR, LDSC
K91	Diagnoses - main ICD10: K91 Postprocedural disorders of digestive system, not elsewhere classified	MR, LDSC
K92	Diagnoses - main ICD10: K92 Other diseases of digestive system	MR, LDSC
L02	Diagnoses - main ICD10: L02 Cutaneous abscess, furuncle and carbuncle	MR
L03	Diagnoses - main ICD10: L03 Cellulitis	MR, LDSC
L05	Diagnoses - main ICD10: L05 Pilonidal cyst	MR, LDSC
L08	Diagnoses - main ICD10: L08 Other local infections of skin and subcutaneous tissue	MR, LDSC
L27	Diagnoses - main ICD10: L27 Dermatitis due to substances taken internally	MR, LDSC
L28	Diagnoses - main ICD10: L28 Lichen simplex chronicus and prurigo	MR
L29	Diagnoses - main ICD10: L29 Pruritus	MR
L30	Diagnoses - main ICD10: L30 Other dermatitis	MR, LDSC
L40	Diagnoses - main ICD10: L40 Psoriasis	MR, LDSC
L43	Diagnoses - main ICD10: L43 Lichen planus	MR, LDSC
L50	Diagnoses - main ICD10: L50 Urticaria	MR, LDSC
L53	Diagnoses - main ICD10: L53 Other erythematous conditions	MR
L57	Diagnoses - main ICD10: L57 Skin changes due to chronic exposure to nonionising radiation	MR, LDSC
L60	Diagnoses - main ICD10: L60 Nail disorders	MR, LDSC
L72	Diagnoses - main ICD10: L72 Follicular cysts of skin and subcutaneous tissue	MR, LDSC
L73	Diagnoses - main ICD10: L73 Other follicular disorders	MR, LDSC
L81	Diagnoses - main ICD10: L81 Other disorders of pigmentation	MR
L82	Diagnoses - main ICD10: L82 Seborrhoeic keratosis	MR, LDSC
L85	Diagnoses - main ICD10: L85 Other epidermal thickening	MR, LDSC
L90	Diagnoses - main ICD10: L90 Atrophic disorders of skin	MR, LDSC

L91	Diagnoses - main ICD10: L91 Hypertrophic disorders of skin	MR, LDSC
L92	Diagnoses - main ICD10: L92 Granulomatous disorders of skin and subcutaneous tissue	MR
L97	Diagnoses - main ICD10: L97 Ulcer of lower limb, not elsewhere classified	MR
L98	Diagnoses - main ICD10: L98 Other disorders of skin and subcutaneous tissue, not elsewhere classified	MR, LDSC
M00	Diagnoses - main ICD10: M00 Pyogenic arthritis	MR
M05	Diagnoses - main ICD10: M05 Seropositive rheumatoid arthritis	MR, LDSC
M06	Diagnoses - main ICD10: M06 Other rheumatoid arthritis	MR, LDSC
M10	Diagnoses - main ICD10: M10 Gout	MR, LDSC
M13	Diagnoses - main ICD10: M13 Other arthritis	MR, LDSC
M15	Diagnoses - main ICD10: M15 Polyarthrosis	MR, LDSC
M16	Diagnoses - main ICD10: M16 Coxarthrosis [arthrosis of hip]	MR, LDSC
M17	Diagnoses - main ICD10: M17 Gonarthrosis [arthrosis of knee]	MR, LDSC
M18	Diagnoses - main ICD10: M18 Arthrosis of first carpometacarpal joint	MR, LDSC
M19	Diagnoses - main ICD10: M19 Other arthrosis	MR, LDSC
M20	Diagnoses - main ICD10: M20 Acquired deformities of fingers and toes	MR, LDSC
M21	Diagnoses - main ICD10: M21 Other acquired deformities of limbs	MR
M22	Diagnoses - main ICD10: M22 Disorders of patella	MR
M23	Diagnoses - main ICD10: M23 Internal derangement of knee	MR, LDSC
M24	Diagnoses - main ICD10: M24 Other specific joint derangements	MR, LDSC
M25	Diagnoses - main ICD10: M25 Other joint disorders, not elsewhere classified	MR, LDSC
M31	Diagnoses - main ICD10: M31 Other necrotising vasculopathies	MR
M32	Diagnoses - main ICD10: M32 Systemic lupus erythematosus	MR
M34	Diagnoses - main ICD10: M34 Systemic sclerosis	MR
M35	Diagnoses - main ICD10: M35 Other systemic involvement of connective tissue	MR
M41	Diagnoses - main ICD10: M41 Scoliosis	MR
M43	Diagnoses - main ICD10: M43 Other deforming dorsopathies	MR
M45	Diagnoses - main ICD10: M45 Ankylosing spondylitis	MR, LDSC
M46	Diagnoses - main ICD10: M46 Other inflammatory spondylopathies	MR, LDSC
M47	Diagnoses - main ICD10: M47 Spondylosis	MR, LDSC
M48	Diagnoses - main ICD10: M48 Other spondylopathies	MR, LDSC

M50	Diagnoses - main ICD10: M50 Cervical disk disorders	MR, LDSC
M51	Diagnoses - main ICD10: M51 Other intervertebral disk disorders	MR, LDSC
M53	Diagnoses - main ICD10: M53 Other dorsopathies, not elsewhere classified	MR, LDSC
M54	Diagnoses - main ICD10: M54 Dorsalgia	MR, LDSC
M60	Diagnoses - main ICD10: M60 Myositis	MR, LDSC
M62	Diagnoses - main ICD10: M62 Other disorders of muscle	MR, LDSC
M65	Diagnoses - main ICD10: M65 Synovitis and tenosynovitis	MR, LDSC
M66	Diagnoses - main ICD10: M66 Spontaneous rupture of synovium and tendon	MR
M67	Diagnoses - main ICD10: M67 Other disorders of synovium and tendon	MR, LDSC
M70	Diagnoses - main ICD10: M70 Soft tissue disorders related to use, overuse and pressure	MR, LDSC
M71	Diagnoses - main ICD10: M71 Other bursopathies	MR
M72	Diagnoses - main ICD10: M72 Fibroblastic disorders	MR, LDSC
M75	Diagnoses - main ICD10: M75 Shoulder lesions	MR, LDSC
M76	Diagnoses - main ICD10: M76 Enthesopathies of lower limb, excluding foot	MR
M77	Diagnoses - main ICD10: M77 Other enthesopathies	MR, LDSC
M79	Diagnoses - main ICD10: M79 Other soft tissue disorders, not elsewhere classified	MR, LDSC
M80	Diagnoses - main ICD10: M80 Osteoporosis with pathological fracture	MR, LDSC
M81	Diagnoses - main ICD10: M81 Osteoporosis without pathological fracture	MR
M84	Diagnoses - main ICD10: M84 Disorders of continuity of bone	MR, LDSC
M85	Diagnoses - main ICD10: M85 Other disorders of bone density and structure	MR
M86	Diagnoses - main ICD10: M86 Osteomyelitis	MR
M87	Diagnoses - main ICD10: M87 Osteonecrosis	MR
M89	Diagnoses - main ICD10: M89 Other disorders of bone	MR
M94	Diagnoses - main ICD10: M94 Other disorders of cartilage	MR
M95	Diagnoses - main ICD10: M95 Other acquired deformities of musculoskeletal system and connective tissue	MR, LDSC
M96	Diagnoses - main ICD10: M96 Postprocedural musculoskeletal disorders, not elsewhere classified	MR
N02	Diagnoses - main ICD10: N02 Recurrent and persistent haematuria	MR
N05	Diagnoses - main ICD10: N05 Unspecified nephritic syndrome	MR, LDSC
N10	Diagnoses - main ICD10: N10 Acute tubulo-interstitial nephritis	MR
N12	Diagnoses - main ICD10: N12 Tubulo-interstitial nephritis, not specified as acute or chronic	MR

N13	Diagnoses - main ICD10: N13 Obstructive and reflux uropathy	MR, LDSC
N17	Diagnoses - main ICD10: N17 Acute renal failure	MR, LDSC
N18	Diagnoses - main ICD10: N18 Chronic renal failure	MR, LDSC
N19	Diagnoses - main ICD10: N19 Unspecified renal failure	MR, LDSC
N20	Diagnoses - main ICD10: N20 Calculus of kidney and ureter	MR, LDSC
N21	Diagnoses - main ICD10: N21 Calculus of lower urinary tract	MR, LDSC
N23	Diagnoses - main ICD10: N23 Unspecified renal colic	MR, LDSC
N28	Diagnoses - main ICD10: N28 Other disorders of kidney and ureter, not elsewhere classified	MR, LDSC
N30	Diagnoses - main ICD10: N30 Cystitis	MR, LDSC
N31	Diagnoses - main ICD10: N31 Neuromuscular dysfunction of bladder, not elsewhere classified	MR
N32	Diagnoses - main ICD10: N32 Other disorders of bladder	MR, LDSC
N34	Diagnoses - main ICD10: N34 Urethritis and urethral syndrome	MR, LDSC
N35	Diagnoses - main ICD10: N35 Urethral stricture	MR, LDSC
N36	Diagnoses - main ICD10: N36 Other disorders of urethra	MR, LDSC
N39	Diagnoses - main ICD10: N39 Other disorders of urinary system	MR, LDSC
N40	Diagnoses - main ICD10: N40 Hyperplasia of prostate	MR, LDSC
N41	Diagnoses - main ICD10: N41 Inflammatory diseases of prostate	MR, LDSC
N42	Diagnoses - main ICD10: N42 Other disorders of prostate	MR
N43	Diagnoses - main ICD10: N43 Hydrocele and spermatocele	MR, LDSC
N45	Diagnoses - main ICD10: N45 Orchitis and epididymitis	MR
N47	Diagnoses - main ICD10: N47 Redundant prepuce, phimosis and paraphimosis	MR, LDSC
N48	Diagnoses - main ICD10: N48 Other disorders of penis	MR
N49	Diagnoses - main ICD10: N49 Inflammatory disorders of male genital organs, not elsewhere classified	MR, LDSC
N50	Diagnoses - main ICD10: N50 Other disorders of male genital organs	MR, LDSC
N60	Diagnoses - main ICD10: N60 Benign mammary dysplasia	MR, LDSC
N61	Diagnoses - main ICD10: N61 Inflammatory disorders of breast	MR
N62	Diagnoses - main ICD10: N62 Hypertrophy of breast	MR
N63	Diagnoses - main ICD10: N63 Unspecified lump in breast	MR, LDSC
N64	Diagnoses - main ICD10: N64 Other disorders of breast	MR, LDSC
N70	Diagnoses - main ICD10: N70 Salpingitis and oophoritis	MR, LDSC

N71	Diagnoses - main ICD10: N71 Inflammatory disease of uterus, except cervix	MR
N72	Diagnoses - main ICD10: N72 Inflammatory disease of cervix uteri	MR
N73	Diagnoses - main ICD10: N73 Other female pelvic inflammatory diseases	MR
N75	Diagnoses - main ICD10: N75 Diseases of Bartholin's gland	MR, LDSC
N76	Diagnoses - main ICD10: N76 Other inflammation of vagina and vulva	MR, LDSC
N80	Diagnoses - main ICD10: N80 Endometriosis	MR, LDSC
N81	Diagnoses - main ICD10: N81 Female genital prolapse	MR, LDSC
N82	Diagnoses - main ICD10: N82 Fistulae involving female genital tract	MR, LDSC
N83	Diagnoses - main ICD10: N83 Noninflammatory disorders of ovary, Fallopian tube and broad ligament	MR, LDSC
N84	Diagnoses - main ICD10: N84 Polyp of female genital tract	MR, LDSC
N85	Diagnoses - main ICD10: N85 Other noninflammatory disorders of uterus, except cervix	MR
N86	Diagnoses - main ICD10: N86 Erosion and ectropion of cervix uteri	MR, LDSC
N87	Diagnoses - main ICD10: N87 Dysplasia of cervix uteri	MR
N88	Diagnoses - main ICD10: N88 Other noninflammatory disorders of cervix uteri	MR
N89	Diagnoses - main ICD10: N89 Other noninflammatory disorders of vagina	MR
N90	Diagnoses - main ICD10: N90 Other noninflammatory disorders of vulva and perineum	MR
N92	Diagnoses - main ICD10: N92 Excessive, frequent and irregular menstruation	MR, LDSC
N93	Diagnoses - main ICD10: N93 Other abnormal uterine and vaginal bleeding	MR, LDSC
N94	Diagnoses - main ICD10: N94 Pain and other conditions associated with female genital organs and menstrual cycle	MR, LDSC
N95	Diagnoses - main ICD10: N95 Menopausal and other perimenopausal disorders	MR
N97	Diagnoses - main ICD10: N97 Female infertility	MR, LDSC
N99	Diagnoses - main ICD10: N99 Postprocedural disorders of genito-urinary system, not elsewhere classified	MR
O00	Diagnoses - main ICD10: O00 Ectopic pregnancy	MR, LDSC
O02	Diagnoses - main ICD10: O02 Other abnormal products of conception	MR, LDSC
O03	Diagnoses - main ICD10: O03 Spontaneous abortion	MR, LDSC
O04	Diagnoses - main ICD10: O04 Medical abortion	MR, LDSC
O13	Diagnoses - main ICD10: O13 Gestational [pregnancy-induced] hypertension without significant proteinuria	MR
O14	Diagnoses - main ICD10: O14 Gestational [pregnancy-induced] hypertension with significant proteinuria	MR
O16	Diagnoses - main ICD10: O16 Unspecified maternal hypertension	MR, LDSC
O20	Diagnoses - main ICD10: O20 Haemorrhage in early pregnancy	MR, LDSC

O21	Diagnoses - main ICD10: O21 Excessive vomiting in pregnancy	MR
O23	Diagnoses - main ICD10: O23 Infections of genito-urinary tract in pregnancy	MR, LDSC
O24	Diagnoses - main ICD10: O24 Diabetes mellitus in pregnancy	MR
O26	Diagnoses - main ICD10: O26 Maternal care for other conditions predominantly related to pregnancy	MR
O32	Diagnoses - main ICD10: O32 Maternal care for known or suspected malpresentation of foetus	MR, LDSC
O34	Diagnoses - main ICD10: O34 Maternal care for known or suspected abnormality of pelvic organs	MR
O35	Diagnoses - main ICD10: O35 Maternal care for known or suspected foetal abnormality and damage	MR
O36	Diagnoses - main ICD10: O36 Maternal care for other known or suspected foetal problems	MR, LDSC
O41	Diagnoses - main ICD10: O41 Other disorders of amniotic fluid and membranes	MR
O42	Diagnoses - main ICD10: O42 Premature rupture of membranes	MR, LDSC
O44	Diagnoses - main ICD10: O44 Placenta praevia	MR
O46	Diagnoses - main ICD10: O46 Antepartum haemorrhage, not elsewhere classified	MR
O47	Diagnoses - main ICD10: O47 False labour	MR, LDSC
O48	Diagnoses - main ICD10: O48 Prolonged pregnancy	MR
O60	Diagnoses - main ICD10: O60 Preterm delivery	MR, LDSC
O62	Diagnoses - main ICD10: O62 Abnormalities of forces of labour	MR
O63	Diagnoses - main ICD10: O63 Long labour	MR
O64	Diagnoses - main ICD10: O64 Obstructed labour due to malposition and malpresentation of foetus	MR, LDSC
O66	Diagnoses - main ICD10: O66 Other obstructed labour	MR
O68	Diagnoses - main ICD10: O68 Labour and delivery complicated by foetal stress [distress]	MR, LDSC
O69	Diagnoses - main ICD10: O69 Labour and delivery complicated by umbilical cord complications	MR, LDSC
O70	Diagnoses - main ICD10: O70 Perineal laceration during delivery	MR, LDSC
O72	Diagnoses - main ICD10: O72 Postpartum haemorrhage	MR
O73	Diagnoses - main ICD10: O73 Retained placenta and membranes, without haemorrhage	MR
O75	Diagnoses - main ICD10: O75 Other complications of labour and delivery, not elsewhere classified	MR, LDSC
O80	Diagnoses - main ICD10: O80 Single spontaneous delivery	MR, LDSC
O81	Diagnoses - main ICD10: O81 Single delivery by forceps and vacuum extractor	MR
O82	Diagnoses - main ICD10: O82 Single delivery by Caesarean section	MR
O90	Diagnoses - main ICD10: O90 Complications of the puerperium, not elsewhere classified	MR, LDSC

O99	Diagnoses - main ICD10: O99 Other maternal diseases classifiable elsewhere but complicating pregnancy, childbirth and the puerperium	MR
Q18	Diagnoses - main ICD10: Q18 Other congenital malformations of face and neck	MR
Q21	Diagnoses - main ICD10: Q21 Congenital malformations of cardiac septa	MR
Q28	Diagnoses - main ICD10: Q28 Other congenital malformations of circulatory system	MR
Q38	Diagnoses - main ICD10: Q38 Other congenital malformations of tongue, mouth and pharynx	MR, LDSC
Q82	Diagnoses - main ICD10: Q82 Other congenital malformations of skin	MR
R00	Diagnoses - main ICD10: R00 Abnormalities of heart beat	MR
R04	Diagnoses - main ICD10: R04 Haemorrhage from respiratory passages	MR, LDSC
R05	Diagnoses - main ICD10: R05 Cough	MR, LDSC
R06	Diagnoses - main ICD10: R06 Abnormalities of breathing	MR, LDSC
R07	Diagnoses - main ICD10: R07 Pain in throat and chest	MR, LDSC
R09	Diagnoses - main ICD10: R09 Other symptoms and signs involving the circulatory and respiratory systems	MR
R10	Diagnoses - main ICD10: R10 Abdominal and pelvic pain	MR, LDSC
R11	Diagnoses - main ICD10: R11 Nausea and vomiting	MR, LDSC
R12	Diagnoses - main ICD10: R12 Heartburn	MR, LDSC
R13	Diagnoses - main ICD10: R13 Dysphagia	MR, LDSC
R14	Diagnoses - main ICD10: R14 Flatulence and related conditions	MR, LDSC
R15	Diagnoses - main ICD10: R15 Faecal incontinence	MR
R17	Diagnoses - main ICD10: R17 Unspecified jaundice	MR
R18	Diagnoses - main ICD10: R18 Ascites	MR
R19	Diagnoses - main ICD10: R19 Other symptoms and signs involving the digestive system and abdomen	MR, LDSC
R20	Diagnoses - main ICD10: R20 Disturbances of skin sensation	MR, LDSC
R21	Diagnoses - main ICD10: R21 Rash and other nonspecific skin eruption	MR
R22	Diagnoses - main ICD10: R22 Localised swelling, mass and lump of skin and subcutaneous tissue	MR
R23	Diagnoses - main ICD10: R23 Other skin changes	MR, LDSC
R25	Diagnoses - main ICD10: R25 Abnormal involuntary movements	MR, LDSC
R26	Diagnoses - main ICD10: R26 Abnormalities of gait and mobility	MR, LDSC
R29	Diagnoses - main ICD10: R29 Other symptoms and signs involving the nervous and musculoskeletal systems	MR
R30	Diagnoses - main ICD10: R30 Pain associated with micturition	MR, LDSC

R31	Diagnoses - main ICD10: R31 Unspecified haematuria	MR, LDSC
R32	Diagnoses - main ICD10: R32 Unspecified urinary incontinence	MR, LDSC
R33	Diagnoses - main ICD10: R33 Retention of urine	MR, LDSC
R35	Diagnoses - main ICD10: R35 Polyuria	MR, LDSC
R39	Diagnoses - main ICD10: R39 Other symptoms and signs involving the urinary system	MR
R40	Diagnoses - main ICD10: R40 Somnolence, stupor and coma	MR, LDSC
R41	Diagnoses - main ICD10: R41 Other symptoms and signs involving cognitive functions and awareness	MR
R42	Diagnoses - main ICD10: R42 Dizziness and giddiness	MR, LDSC
R47	Diagnoses - main ICD10: R47 Speech disturbances, not elsewhere classified	MR, LDSC
R49	Diagnoses - main ICD10: R49 Voice disturbances	MR
R50	Diagnoses - main ICD10: R50 Fever of unknown origin	MR, LDSC
R51	Diagnoses - main ICD10: R51 Headache	MR, LDSC
R52	Diagnoses - main ICD10: R52 Pain, not elsewhere classified	MR
R53	Diagnoses - main ICD10: R53 Malaise and fatigue	MR, LDSC
R54	Diagnoses - main ICD10: R54 Senility	MR, LDSC
R55	Diagnoses - main ICD10: R55 Syncope and collapse	MR, LDSC
R56	Diagnoses - main ICD10: R56 Convulsions, not elsewhere classified	MR
R59	Diagnoses - main ICD10: R59 Enlarged lymph nodes	MR, LDSC
R60	Diagnoses - main ICD10: R60 Oedema, not elsewhere classified	MR
R61	Diagnoses - main ICD10: R61 Hyperhidrosis	MR, LDSC
R63	Diagnoses - main ICD10: R63 Symptoms and signs concerning food and fluid intake	MR
R68	Diagnoses - main ICD10: R68 Other general symptoms and signs	MR
R69	Diagnoses - main ICD10: R69 Unknown and unspecified causes of morbidity	MR, LDSC
R73	Diagnoses - main ICD10: R73 Elevated blood glucose level	MR
R76	Diagnoses - main ICD10: R76 Other abnormal immunological findings in serum	MR
R79	Diagnoses - main ICD10: R79 Other abnormal findings of blood chemistry	MR, LDSC
R86	Diagnoses - main ICD10: R86 Abnormal findings in specimens from male genital organs	MR, LDSC
R87	Diagnoses - main ICD10: R87 Abnormal findings in specimens from female genital organs	MR
R90	Diagnoses - main ICD10: R90 Abnormal findings on diagnostic imaging of central nervous system	MR
R91	Diagnoses - main ICD10: R91 Abnormal findings on diagnostic imaging of lung	MR

R93	Diagnoses - main ICD10: R93 Abnormal findings on diagnostic imaging of other body structures	MR
R94	Diagnoses - main ICD10: R94 Abnormal results of function studies	MR
S00	Diagnoses - main ICD10: S00 Superficial injury of head	MR, LDSC
S01	Diagnoses - main ICD10: S01 Open wound of head	MR, LDSC
S02	Diagnoses - main ICD10: S02 Fracture of skull and facial bones	MR
S05	Diagnoses - main ICD10: S05 Injury of eye and orbit	MR
S06	Diagnoses - main ICD10: S06 Intracranial injury	MR
S09	Diagnoses - main ICD10: S09 Other and unspecified injuries of head	MR, LDSC
S12	Diagnoses - main ICD10: S12 Fracture of neck	MR, LDSC
S13	Diagnoses - main ICD10: S13 Dislocation, sprain and strain of joints and ligaments at neck level	MR, LDSC
S20	Diagnoses - main ICD10: S20 Superficial injury of thorax	MR, LDSC
S22	Diagnoses - main ICD10: S22 Fracture of rib(s), sternum and thoracic spine	MR, LDSC
S27	Diagnoses - main ICD10: S27 Injury of other and unspecified intrathoracic organs	MR
S30	Diagnoses - main ICD10: S30 Superficial injury of abdomen, lower back and pelvis	MR, LDSC
S31	Diagnoses - main ICD10: S31 Open wound of abdomen, lower back and pelvis	MR
S32	Diagnoses - main ICD10: S32 Fracture of lumbar spine and pelvis	MR
S39	Diagnoses - main ICD10: S39 Other and unspecified injuries of abdomen, lower back and pelvis	MR
S42	Diagnoses - main ICD10: S42 Fracture of shoulder and upper arm	MR, LDSC
S43	Diagnoses - main ICD10: S43 Dislocation, sprain and strain of joints and ligaments of shoulder girdle	MR
S46	Diagnoses - main ICD10: S46 Injury of muscle and tendon at shoulder and upper arm level	MR, LDSC
S51	Diagnoses - main ICD10: S51 Open wound of forearm	MR
S52	Diagnoses - main ICD10: S52 Fracture of forearm	MR, LDSC
S56	Diagnoses - main ICD10: S56 Injury of muscle and tendon at forearm level	MR, LDSC
S60	Diagnoses - main ICD10: S60 Superficial injury of wrist and hand	MR
S61	Diagnoses - main ICD10: S61 Open wound of wrist and hand	MR, LDSC
S62	Diagnoses - main ICD10: S62 Fracture at wrist and hand level	MR, LDSC
S63	Diagnoses - main ICD10: S63 Dislocation, sprain and strain of joints and ligaments at wrist and hand level	MR
S64	Diagnoses - main ICD10: S64 Injury of nerves at wrist and hand level	MR
S66	Diagnoses - main ICD10: S66 Injury of muscle and tendon at wrist and hand level	MR, LDSC
S67	Diagnoses - main ICD10: S67 Crushing injury of wrist and hand	MR

S68	Diagnoses - main ICD10: S68 Traumatic amputation of wrist and hand	MR
S69	Diagnoses - main ICD10: S69 Other and unspecified injuries of wrist and hand	MR, LDSC
S72	Diagnoses - main ICD10: S72 Fracture of femur	MR, LDSC
S76	Diagnoses - main ICD10: S76 Injury of muscle and tendon at hip and thigh level	MR, LDSC
S80	Diagnoses - main ICD10: S80 Superficial injury of lower leg	MR
S81	Diagnoses - main ICD10: S81 Open wound of lower leg	MR
S82	Diagnoses - main ICD10: S82 Fracture of lower leg, including ankle	MR, LDSC
S83	Diagnoses - main ICD10: S83 Dislocation, sprain and strain of joints and ligaments of knee	MR, LDSC
S86	Diagnoses - main ICD10: S86 Injury of muscle and tendon at lower leg level	MR, LDSC
S89	Diagnoses - main ICD10: S89 Other and unspecified injuries of lower leg	MR
S91	Diagnoses - main ICD10: S91 Open wound of ankle and foot	MR
S92	Diagnoses - main ICD10: S92 Fracture of foot, except ankle	MR, LDSC
S93	Diagnoses - main ICD10: S93 Dislocation, sprain and strain of joints and ligaments at ankle and foot level	MR
T14	Diagnoses - main ICD10: T14 Injury of unspecified body region	MR
T17	Diagnoses - main ICD10: T17 Foreign body in respiratory tract	MR, LDSC
T18	Diagnoses - main ICD10: T18 Foreign body in alimentary tract	MR, LDSC
T39	Diagnoses - main ICD10: T39 Poisoning by nonopioid analgesics, antipyretics and antirheumatics	MR, LDSC
T40	Diagnoses - main ICD10: T40 Poisoning by narcotics and psychodysleptics [hallucinogens]	MR, LDSC
T42	Diagnoses - main ICD10: T42 Poisoning by antiepileptic, sedative-hypnotic and anti-Parkinsonism drugs	MR
T43	Diagnoses - main ICD10: T43 Poisoning by psychotropic drugs, not elsewhere classified	MR, LDSC
T50	Diagnoses - main ICD10: T50 Poisoning by diuretics and other and unspecified drugs, medicaments and biological substances	MR, LDSC
T78	Diagnoses - main ICD10: T78 Adverse effects, not elsewhere classified	MR
T79	Diagnoses - main ICD10: T79 Certain early complications of trauma, not elsewhere classified	MR, LDSC
T81	Diagnoses - main ICD10: T81 Complications of procedures, not elsewhere classified	MR, LDSC
T82	Diagnoses - main ICD10: T82 Complications of cardiac and vascular prosthetic devices, implants and grafts	MR
T83	Diagnoses - main ICD10: T83 Complications of genito-urinary prosthetic devices, implants and grafts	MR, LDSC
T84	Diagnoses - main ICD10: T84 Complications of internal orthopaedic prosthetic devices, implants and grafts	MR, LDSC
T85	Diagnoses - main ICD10: T85 Complications of other internal prosthetic devices, implants and grafts	MR
T86	Diagnoses - main ICD10: T86 Failure and rejection of transplanted organs and tissues	MR, LDSC

T87	Diagnoses - main ICD10: T87 Complications peculiar to reattachment and amputation	MR
T88	Diagnoses - main ICD10: T88 Other complications of surgical and medical care, not elsewhere classified	MR
Z00	Diagnoses - main ICD10: Z00 General examination and investigation of persons without complaint or reported diagnosis	MR, LDSC
Z01	Diagnoses - main ICD10: Z01 Other special examinations and investigations of persons without complaint or reported diagnosis	MR, LDSC
Z03	Diagnoses - main ICD10: Z03 Medical observation and evaluation for suspected diseases and conditions	MR, LDSC
Z04	Diagnoses - main ICD10: Z04 Examination and observation for other reasons	MR
Z08	Diagnoses - main ICD10: Z08 Follow-up examination after treatment for malignant neoplasm	MR, LDSC
Z09	Diagnoses - main ICD10: Z09 Follow-up examination after treatment for conditions other than malignant neoplasms	MR, LDSC
Z11	Diagnoses - main ICD10: Z11 Special screening examination for infectious and parasitic diseases	MR
Z12	Diagnoses - main ICD10: Z12 Special screening examination for neoplasms	MR, LDSC
Z13	Diagnoses - main ICD10: Z13 Special screening examination for other diseases and disorders	MR
Z30	Diagnoses - main ICD10: Z30 Contraceptive management	MR, LDSC
Z31	Diagnoses - main ICD10: Z31 Procreative management	MR
Z32	Diagnoses - main ICD10: Z32 Pregnancy examination and test	MR
Z34	Diagnoses - main ICD10: Z34 Supervision of normal pregnancy	MR, LDSC
Z35	Diagnoses - main ICD10: Z35 Supervision of high-risk pregnancy	MR, LDSC
Z36	Diagnoses - main ICD10: Z36 Antenatal screening	MR, LDSC
Z39	Diagnoses - main ICD10: Z39 Postpartum care and examination	MR
Z40	Diagnoses - main ICD10: Z40 Prophylactic surgery	MR
Z41	Diagnoses - main ICD10: Z41 Procedures for purposes other than remedying health state	MR
Z42	Diagnoses - main ICD10: Z42 Follow-up care involving plastic surgery	MR, LDSC
Z43	Diagnoses - main ICD10: Z43 Attention to artificial openings	MR
Z44	Diagnoses - main ICD10: Z44 Fitting and adjustment of external prosthetic device	MR, LDSC
Z45	Diagnoses - main ICD10: Z45 Adjustment and management of implanted device	MR, LDSC
Z46	Diagnoses - main ICD10: Z46 Fitting and adjustment of other devices	MR, LDSC
Z47	Diagnoses - main ICD10: Z47 Other orthopaedic follow-up care	MR, LDSC
Z48	Diagnoses - main ICD10: Z48 Other surgical follow-up care	MR, LDSC
Z50	Diagnoses - main ICD10: Z50 Care involving use of rehabilitation procedures	MR
Z51	Diagnoses - main ICD10: Z51 Other medical care	MR, LDSC

Z52	Diagnoses - main ICD10: Z52 Donors of organs and tissues	MR, LDSC
Z53	Diagnoses - main ICD10: Z53 Persons encountering health services for specifie procedures, not carried out	MR, LDSC
Z71	Diagnoses - main ICD10: Z71 Persons encountering health services for other counselling and medical advice, not elsewhere classified	MR, LDSC
Z76	Diagnoses - main ICD10: Z76 Persons encountering health services in other circumstances	MR
Z80	Diagnoses - main ICD10: Z80 Family history of malignant neoplasm	MR, LDSC
Z85	Diagnoses - main ICD10: Z85 Personal history of malignant neoplasm	MR
Z87	Diagnoses - main ICD10: Z87 Personal history of other diseases and conditions	MR

Table S3: List of the MR methods used in the study.

**Method**
Egger fixed effects - steiger
Egger fixed effects - tophits
Egger random effects - steiger
Egger random effects - tophits
IVW fixed effects - steiger
IVW fixed effects - tophits
IVW random effects - steiger
IVW random effects - tophits
Penalised median - steiger
Penalised median - tophits
Rucker mean (JK) - steiger
Rucker mean (JK) - tophits
Rucker median (JK) - steiger
Rucker median (JK) - tophits
Rucker point estimate - steiger
Rucker point estimate - tophits
Simple median - steiger
Simple median - tophits
Simple mode - steiger
Simple mode - tophits
Simple mode (NOME) - steiger
Simple mode (NOME) - tophits
Weighted median - steiger
Weighted median - tophits
Weighted mode - steiger
Weighted mode - tophits
Weighted mode (NOME) - steiger
Weighted mode (NOME) - tophits

Table S4: Significant causal associations identified using TSH genetic instrument (FDR $q < 0.05$).

Phenotypes	Method	Estimate	SE	P	FDR
Self-reported: hypothyroidism	Penalised median - steiger	0.020	0.0023	2.39E-17	1.68E-12
ICD10: non-toxic goitre	IVW fixed effects - tophits	-0.002	0.0001	9.45E-14	8.29E-10
Impedance of arm (left)	IVW fixed effects - tophits	-0.029	0.0017	2.22E-13	1.30E-09
Treatment/medication code: levothyroxine sodium	IVW fixed effects - tophits	0.017	0.0008	2.41E-13	1.30E-09
Impedance of arm (right)	IVW fixed effects - tophits	-0.024	0.0018	9.60E-12	3.74E-08
Self-reported deep venous thrombosis (DVT)	Egger fixed effects - tophits	-0.009	0.0007	1.70E-10	5.42E-07
Standing height	IVW fixed effects - tophits	-0.023	0.0019	2.76E-10	8.07E-07
Blood clot in the leg diagnosed by doctor	IVW fixed effects - tophits	0.003	0.0003	3.09E-10	8.34E-07
Comparative height size at age 10	IVW fixed effects - tophits	-0.028	0.0026	1.74E-09	4.21E-06
Pulse rate, automated reading	IVW fixed effects - tophits	-0.037	0.0041	2.84E-08	5.24E-05
Treatment/medication code: thyroxine product	IVW fixed effects - tophits	0.005	0.0005	3.34E-08	5.86E-05
Impedance of whole body	IVW fixed effects - tophits	-0.022	0.0027	4.43E-08	7.06E-05
ICD10: Phlebitis and thrombophlebitis	Egger fixed effects - tophits	-0.003	0.0004	5.94E-08	8.55E-05
Self-reported hyperthyroidism	IVW fixed effects - tophits	-0.004	0.0004	7.49E-08	1.01E-04
ICD10: I26 Pulmonary embolism	Egger fixed effects - tophits	-0.003	0.0005	1.01E-06	0.001
Self-reported pulmonary embolism +/- DVT	Egger fixed effects - tophits	-0.004	0.0005	2.65E-06	0.003
Self-reported thyroid problem (not cancer)	IVW fixed effects - tophits	-0.001	0.0002	3.71E-06	0.004
Trunk predicted mass	Egger fixed effects - tophits	0.032	0.0050	3.89E-06	0.004
Trunk fat-free mass	Egger fixed effects - tophits	0.032	0.0049	4.07E-06	0.004
ICD10: Female infertility	IVW random effects - tophits	0.001	0.0002	4.91E-06	0.005
Blood clot in the lung diagnosed by doctor	Egger fixed effects - tophits	-0.003	0.0005	5.76E-06	0.005
Arm fat-free mass (left)	Egger fixed effects - tophits	0.031	0.0053	1.45E-05	0.012
Hip circumference	IVW fixed effects - tophits	-0.022	0.0039	1.51E-05	0.012
Trunk fat percentage	Egger fixed effects - tophits	-0.048	0.0087	2.23E-05	0.017
Arm predicted mass (right)	Egger fixed effects - tophits	0.028	0.0051	3.07E-05	0.021
Arm fat-free mass (right)	Egger fixed effects - tophits	0.026	0.0050	4.59E-05	0.031
Arm predicted mass (left)	Egger fixed effects - tophits	0.027	0.0052	5.01E-05	0.033
Hearing aid user	Rucker median (JK) - tophits	-0.005	0.0010	7.10E-05	0.045
self-reported high cholesterol	IVW fixed effects - tophits	0.006	0.0011	7.48E-05	0.046

Table S5. Significant causal associations identified using TSH genetic instrument with the corresponding sensitivity analyses.

Phenotype (MR-Egger: Intercept, p-value)	Method	Beta (postive=red; negative=blue)	SE	P
Arm fat-free mass (left) (-0.002497, 0.229)	Egger fixed effects - steiger	**3.07E-02**	5.32E-03	1.45E-05
	Egger fixed effects - tophits	**3.07E-02**	5.32E-03	1.45E-05
	Egger random effects - steiger	**3.07E-02**	2.12E-02	1.63E-01
	Egger random effects - tophits	**3.07E-02**	2.12E-02	1.63E-01
	IVW fixed effects - steiger	**6.46E-03**	2.03E-03	4.65E-03
	IVW fixed effects - tophits	**6.46E-03**	2.03E-03	4.65E-03
	IVW random effects - steiger	**6.46E-03**	8.30E-03	4.45E-01
	IVW random effects - tophits	**6.46E-03**	8.30E-03	4.45E-01
	Penalised median - steiger	**-1.28E-03**	6.74E-03	8.49E-01
	Penalised median - tophits	**-1.28E-03**	6.87E-03	8.52E-01
	Rucker mean (JK) - steiger	**3.05E-02**	2.28E-02	1.97E-01
	Rucker mean (JK) - tophits	**3.00E-02**	2.41E-02	2.29E-01
	Rucker median (JK) - steiger	**2.93E-02**	2.62E-02	2.77E-01
	Rucker median (JK) - tophits	**2.95E-02**	2.71E-02	2.90E-01
	Rucker point estimate - steiger	**3.07E-02**	2.12E-02	1.63E-01
	Rucker point estimate - tophits	**3.07E-02**	2.12E-02	1.63E-01
	Simple median - steiger	**-4.61E-04**	7.62E-03	9.52E-01
	Simple median - tophits	**-4.61E-04**	7.72E-03	9.52E-01
	Simple mode - steiger	**1.53E-03**	1.41E-02	9.14E-01
	Simple mode - tophits	**1.53E-03**	1.27E-02	9.05E-01
	Simple mode (NOME) - steiger	**1.53E-03**	1.39E-02	9.13E-01
	Simple mode (NOME) - tophits	**1.53E-03**	1.26E-02	9.04E-01
	Weighted median - steiger	**-7.68E-04**	6.77E-03	9.10E-01
	Weighted median - tophits	**-7.68E-04**	6.96E-03	9.12E-01
	Weighted mode - steiger	**-2.76E-03**	8.06E-03	7.35E-01
	Weighted mode - tophits	**-2.76E-03**	7.40E-03	7.13E-01
	Weighted mode (NOME) - steiger	**-2.15E-03**	8.04E-03	7.92E-01

	Weighted mode (NOME) - tophits	**-2.15E-03**	7.67E-03	7.82E-01
Arm fat-free mass (right) (-0.002126, 0.314)	Egger fixed effects - steiger	**2.62E-02**	5.00E-03	4.59E-05
	Egger fixed effects - tophits	**2.62E-02**	5.00E-03	4.59E-05
	Egger random effects - steiger	**2.62E-02**	2.16E-02	2.40E-01
	Egger random effects - tophits	**2.62E-02**	2.16E-02	2.40E-01
	IVW fixed effects - steiger	**5.58E-03**	1.93E-03	9.04E-03
	IVW fixed effects - tophits	**5.58E-03**	1.93E-03	9.04E-03
	IVW random effects - steiger	**5.58E-03**	8.38E-03	5.13E-01
	IVW random effects - tophits	**5.58E-03**	8.38E-03	5.13E-01
	Penalised median - steiger	**-4.28E-03**	6.47E-03	5.08E-01
	Penalised median - tophits	**-4.28E-03**	6.72E-03	5.24E-01
	Rucker mean (JK) - steiger	**2.61E-02**	2.26E-02	2.62E-01
	Rucker mean (JK) - tophits	**2.49E-02**	2.24E-02	2.78E-01
	Rucker median (JK) - steiger	**2.23E-02**	2.40E-02	3.64E-01
	Rucker median (JK) - tophits	**1.97E-02**	2.32E-02	4.06E-01
	Rucker point estimate - steiger	**2.62E-02**	2.16E-02	2.40E-01
	Rucker point estimate - tophits	**2.62E-02**	2.16E-02	2.40E-01
	Simple median - steiger	**1.07E-02**	7.81E-03	1.73E-01
	Simple median - tophits	**1.07E-02**	7.72E-03	1.68E-01
	Simple mode - steiger	**8.83E-03**	1.46E-02	5.51E-01
	Simple mode - tophits	**8.83E-03**	1.45E-02	5.48E-01
	Simple mode (NOME) - steiger	**8.83E-03**	1.64E-02	5.96E-01
	Simple mode (NOME) - tophits	**8.83E-03**	1.58E-02	5.81E-01
	Weighted median - steiger	**1.83E-04**	6.85E-03	9.79E-01
	Weighted median - tophits	**1.83E-04**	6.74E-03	9.78E-01
	Weighted mode - steiger	**-2.99E-03**	7.92E-03	7.09E-01
	Weighted mode - tophits	**-2.99E-03**	7.69E-03	7.01E-01
	Weighted mode (NOME) - steiger	**-2.40E-03**	8.00E-03	7.67E-01
	Weighted mode (NOME) - tophits	**-2.40E-03**	8.58E-03	7.82E-01
Arm predicted mass (left) (-0.002323, 0.271)	Egger fixed effects - steiger	**2.70E-02**	5.18E-03	5.01E-05

	Egger fixed effects - tophits	**2.70E-02**	5.18E-03	5.01E-05
	Egger random effects - steiger	**2.70E-02**	2.16E-02	2.26E-01
	Egger random effects - tophits	**2.70E-02**	2.16E-02	2.26E-01
	IVW fixed effects - steiger	**4.42E-03**	1.99E-03	3.79E-02
	IVW fixed effects - tophits	**4.42E-03**	1.99E-03	3.79E-02
	IVW random effects - steiger	**4.42E-03**	8.40E-03	6.04E-01
	IVW random effects - tophits	**4.42E-03**	8.40E-03	6.04E-01
	Penalised median - steiger	**-5.37E-03**	6.28E-03	3.92E-01
	Penalised median - tophits	**-5.37E-03**	6.30E-03	3.93E-01
	Rucker mean (JK) - steiger	**2.68E-02**	2.33E-02	2.63E-01
	Rucker mean (JK) - tophits	**2.59E-02**	2.27E-02	2.67E-01
	Rucker median (JK) - steiger	**2.38E-02**	2.60E-02	3.71E-01
	Rucker median (JK) - tophits	**2.26E-02**	2.51E-02	3.78E-01
	Rucker point estimate - steiger	**2.70E-02**	2.16E-02	2.26E-01
	Rucker point estimate - tophits	**2.70E-02**	2.16E-02	2.26E-01
	Simple median - steiger	**4.84E-03**	7.39E-03	5.13E-01
	Simple median - tophits	**4.84E-03**	7.85E-03	5.37E-01
	Simple mode - steiger	**1.52E-03**	1.37E-02	9.13E-01
	Simple mode - tophits	**1.52E-03**	1.39E-02	9.14E-01
	Simple mode (NOME) - steiger	**1.52E-03**	1.46E-02	9.18E-01
	Simple mode (NOME) - tophits	**1.52E-03**	1.40E-02	9.15E-01
	Weighted median - steiger	**-2.11E-03**	7.01E-03	7.63E-01
	Weighted median - tophits	**-2.11E-03**	6.74E-03	7.54E-01
	Weighted mode - steiger	**-5.62E-03**	7.43E-03	4.59E-01
	Weighted mode - tophits	**-5.62E-03**	7.70E-03	4.74E-01
	Weighted mode (NOME) - steiger	**-5.62E-03**	8.33E-03	5.08E-01
	Weighted mode (NOME) - tophits	**-5.62E-03**	7.91E-03	4.86E-01
Arm predicted mass (right) (-0.002210, 0.286)	Egger fixed effects - steiger	**2.75E-02**	5.07E-03	3.07E-05
	Egger fixed effects - tophits	**2.75E-02**	5.07E-03	3.07E-05
	Egger random effects - steiger	**2.75E-02**	2.12E-02	2.09E-01

	Egger random effects - tophits	**2.75E-02**	2.12E-02	2.09E-01
	IVW fixed effects - steiger	**6.06E-03**	1.95E-03	5.52E-03
	IVW fixed effects - tophits	**6.06E-03**	1.95E-03	5.52E-03
	IVW random effects - steiger	**6.06E-03**	8.23E-03	4.70E-01
	IVW random effects - tophits	**6.06E-03**	8.23E-03	4.70E-01
	Penalised median - steiger	**-1.39E-03**	6.72E-03	8.36E-01
	Penalised median - tophits	**-1.39E-03**	6.85E-03	8.39E-01
	Rucker mean (JK) - steiger	**2.73E-02**	2.10E-02	2.10E-01
	Rucker mean (JK) - tophits	**2.78E-02**	2.26E-02	2.34E-01
	Rucker median (JK) - steiger	**2.62E-02**	2.33E-02	2.74E-01
	Rucker median (JK) - tophits	**2.54E-02**	2.49E-02	3.19E-01
	Rucker point estimate - steiger	**2.75E-02**	2.12E-02	2.09E-01
	Rucker point estimate - tophits	**2.75E-02**	2.12E-02	2.09E-01
	Simple median - steiger	**8.24E-03**	7.63E-03	2.81E-01
	Simple median - tophits	**8.24E-03**	7.39E-03	2.65E-01
	Simple mode - steiger	**6.65E-03**	1.35E-02	6.28E-01
	Simple mode - tophits	**6.65E-03**	1.39E-02	6.38E-01
	Simple mode (NOME) - steiger	**6.65E-03**	1.44E-02	6.50E-01
	Simple mode (NOME) - tophits	**6.65E-03**	1.38E-02	6.34E-01
	Weighted median - steiger	**3.95E-03**	6.91E-03	5.68E-01
	Weighted median - tophits	**3.95E-03**	6.65E-03	5.53E-01
	Weighted mode - steiger	**-2.50E-03**	8.02E-03	7.59E-01
	Weighted mode - tophits	**-2.50E-03**	8.43E-03	7.70E-01
	Weighted mode (NOME) - steiger	**-2.50E-03**	8.63E-03	7.75E-01
	Weighted mode (NOME) - tophits	**-2.50E-03**	8.19E-03	7.64E-01
Blood clot, DVT, bronchitis, emphysema, asthma, rhinitis, eczema, allergy diagnosed by doctor: Blood clot in the leg (DVT) (0.001002, 0.230)	Egger fixed effects - steiger	**-6.88E-03**	6.56E-04	2.42E-09
	Egger fixed effects - tophits	**-6.88E-03**	6.56E-04	2.42E-09
	Egger random effects - steiger	**-6.88E-03**	8.59E-03	4.33E-01
	Egger random effects - tophits	**-6.88E-03**	8.59E-03	4.33E-01
	IVW fixed effects - steiger	**2.91E-03**	2.54E-04	3.09E-10

	IVW fixed effects - tophits	2.91E-03	2.54E-04	3.09E-10
	IVW random effects - steiger	2.91E-03	3.42E-03	4.05E-01
	IVW random effects - tophits	2.91E-03	3.42E-03	4.05E-01
	Penalised median - steiger	2.07E-03	1.31E-03	1.15E-01
	Penalised median - tophits	2.07E-03	1.32E-03	1.17E-01
	Rucker mean (JK) - steiger	-6.87E-03	7.49E-03	3.70E-01
	Rucker mean (JK) - tophits	-6.76E-03	7.56E-03	3.81E-01
	Rucker median (JK) - steiger	-6.23E-03	9.29E-03	5.10E-01
	Rucker median (JK) - tophits	-6.06E-03	8.90E-03	5.04E-01
	Rucker point estimate - steiger	-6.88E-03	8.59E-03	4.33E-01
	Rucker point estimate - tophits	-6.88E-03	8.59E-03	4.33E-01
	Simple median - steiger	1.77E-03	1.42E-03	2.13E-01
	Simple median - tophits	1.77E-03	1.43E-03	2.15E-01
	Simple mode - steiger	1.17E-03	2.12E-03	5.87E-01
	Simple mode - tophits	1.17E-03	2.06E-03	5.76E-01
	Simple mode (NOME) - steiger	1.17E-03	2.23E-03	6.07E-01
	Simple mode (NOME) - tophits	1.17E-03	2.15E-03	5.92E-01
	Weighted median - steiger	1.87E-03	1.33E-03	1.59E-01
	Weighted median - tophits	1.87E-03	1.27E-03	1.41E-01
	Weighted mode - steiger	1.64E-03	1.85E-03	3.86E-01
	Weighted mode - tophits	1.64E-03	1.88E-03	3.93E-01
	Weighted mode (NOME) - steiger	1.64E-03	1.96E-03	4.11E-01
	Weighted mode (NOME) - tophits	1.64E-03	1.83E-03	3.81E-01
Blood clot, DVT, bronchitis, emphysema, asthma, rhinitis, eczema, allergy diagnosed by doctor: Blood clot in the lung (0.000424, 0.310)	Egger fixed effects - steiger	-3.30E-03	5.32E-04	5.76E-06
	Egger fixed effects - tophits	-3.30E-03	5.32E-04	5.76E-06
	Egger random effects - steiger	-3.30E-03	4.33E-03	4.54E-01
	Egger random effects - tophits	-3.30E-03	4.33E-03	4.54E-01
	IVW fixed effects - steiger	8.43E-04	2.09E-04	6.40E-04
	IVW fixed effects - tophits	8.43E-04	2.09E-04	6.40E-04
	IVW random effects - steiger	8.43E-04	1.70E-03	6.26E-01

	IVW random effects - tophits	**8.43E-04**	1.70E-03	6.26E-01
	Penalised median - steiger	**-1.03E-04**	8.82E-04	9.07E-01
	Penalised median - tophits	**-1.03E-04**	8.66E-04	9.05E-01
	Rucker mean (JK) - steiger	**-3.19E-03**	3.52E-03	3.76E-01
	Rucker mean (JK) - tophits	**-3.38E-03**	3.85E-03	3.90E-01
	Rucker median (JK) - steiger	**-2.51E-03**	3.64E-03	4.99E-01
	Rucker median (JK) - tophits	**-2.85E-03**	4.05E-03	4.90E-01
	Rucker point estimate - steiger	**-3.30E-03**	4.33E-03	4.54E-01
	Rucker point estimate - tophits	**-3.30E-03**	4.33E-03	4.54E-01
	Simple median - steiger	**-3.80E-04**	9.36E-04	6.85E-01
	Simple median - tophits	**-3.80E-04**	9.67E-04	6.95E-01
	Simple mode - steiger	**-5.87E-04**	1.42E-03	6.84E-01
	Simple mode - tophits	**-5.87E-04**	1.53E-03	7.05E-01
	Simple mode (NOME) - steiger	**-5.87E-04**	1.44E-03	6.88E-01
	Simple mode (NOME) - tophits	**-5.87E-04**	1.34E-03	6.65E-01
	Weighted median - steiger	**-5.36E-05**	8.88E-04	9.52E-01
	Weighted median - tophits	**-5.36E-05**	8.75E-04	9.51E-01
	Weighted mode - steiger	**2.41E-04**	1.07E-03	8.25E-01
	Weighted mode - tophits	**2.41E-04**	1.09E-03	8.27E-01
	Weighted mode (NOME) - steiger	**2.41E-04**	1.11E-03	8.31E-01
	Weighted mode (NOME) - tophits	**2.41E-04**	1.09E-03	8.27E-01
**Comparative height size at age 10 (-0.000089, 0.964)**	Egger fixed effects - steiger	**-2.71E-02**	6.44E-03	5.32E-04
	Egger fixed effects - tophits	**-2.71E-02**	6.44E-03	5.32E-04
	Egger random effects - steiger	**-2.71E-02**	2.05E-02	2.04E-01
	Egger random effects - tophits	**-2.71E-02**	2.05E-02	2.04E-01
	IVW fixed effects - steiger	**-2.79E-02**	2.61E-03	1.74E-09
	IVW fixed effects - tophits	**-2.79E-02**	2.61E-03	1.74E-09
	IVW random effects - steiger	**-2.79E-02**	7.88E-03	2.17E-03
	IVW random effects - tophits	**-2.79E-02**	7.88E-03	2.17E-03
	Penalised median - steiger	**-2.22E-02**	7.99E-03	5.40E-03

	Penalised median - tophits	**-2.22E-02**	7.62E-03	3.51E-03
	Rucker mean (JK) - steiger	**-2.40E-02**	2.13E-02	2.74E-01
	Rucker mean (JK) - tophits	**-2.45E-02**	2.01E-02	2.37E-01
	Rucker median (JK) - steiger	**-2.60E-02**	1.31E-02	6.20E-02
	Rucker median (JK) - tophits	**-2.67E-02**	1.23E-02	4.29E-02
	Rucker point estimate - steiger	**-2.79E-02**	7.88E-03	2.17E-03
	Rucker point estimate - tophits	**-2.79E-02**	7.88E-03	2.17E-03
	Simple median - steiger	**-2.47E-02**	7.99E-03	2.05E-03
	Simple median - tophits	**-2.47E-02**	7.86E-03	1.71E-03
	Simple mode - steiger	**-2.65E-02**	1.45E-02	8.39E-02
	Simple mode - tophits	**-2.65E-02**	1.50E-02	9.37E-02
	Simple mode (NOME) - steiger	**-2.65E-02**	1.45E-02	8.24E-02
	Simple mode (NOME) - tophits	**-2.65E-02**	1.51E-02	9.44E-02
	Weighted median - steiger	**-2.50E-02**	7.95E-03	1.65E-03
	Weighted median - tophits	**-2.50E-02**	8.37E-03	2.82E-03
	Weighted mode - steiger	**-2.25E-02**	1.51E-02	1.53E-01
	Weighted mode - tophits	**-2.25E-02**	1.55E-02	1.65E-01
	Weighted mode (NOME) - steiger	**-2.48E-02**	1.45E-02	1.03E-01
	Weighted mode (NOME) - tophits	**-2.48E-02**	1.42E-02	9.80E-02
**Diagnoses - main ICD10: E04 Other non-toxic goitre (-0.000196, 0.240)**	Egger fixed effects - steiger	**-6.10E-04**	3.67E-04	1.13E-01
	Egger fixed effects - tophits	**-6.10E-04**	3.67E-04	1.13E-01
	Egger random effects - steiger	**-6.10E-04**	1.67E-03	7.19E-01
	Egger random effects - tophits	**-6.10E-04**	1.67E-03	7.19E-01
	IVW fixed effects - steiger	**-2.48E-03**	1.39E-04	9.45E-14
	IVW fixed effects - tophits	**-2.48E-03**	1.39E-04	9.45E-14
	IVW random effects - steiger	**-2.48E-03**	6.49E-04	1.06E-03
	IVW random effects - tophits	**-2.48E-03**	6.49E-04	1.06E-03
	Penalised median - steiger	**-8.10E-04**	5.70E-04	1.56E-01
	Penalised median - tophits	**-8.10E-04**	5.81E-04	1.64E-01
	Rucker mean (JK) - steiger	**-1.09E-03**	1.91E-03	5.75E-01

	Rucker mean (JK) - tophits	**-9.64E-04**	1.95E-03	6.27E-01
	Rucker median (JK) - steiger	**-1.01E-03**	2.32E-03	6.70E-01
	Rucker median (JK) - tophits	**-7.18E-04**	2.26E-03	7.54E-01
	Rucker point estimate - steiger	**-6.10E-04**	1.67E-03	7.19E-01
	Rucker point estimate - tophits	**-6.10E-04**	1.67E-03	7.19E-01
	Simple median - steiger	**-3.79E-03**	6.37E-04	2.74E-09
	Simple median - tophits	**-3.79E-03**	6.27E-04	1.51E-09
	Simple mode - steiger	**-5.36E-03**	1.78E-03	6.99E-03
	Simple mode - tophits	**-5.36E-03**	1.90E-03	1.05E-02
	Simple mode (NOME) - steiger	**-5.36E-03**	1.68E-03	4.57E-03
	Simple mode (NOME) - tophits	**-5.36E-03**	1.54E-03	2.41E-03
	Weighted median - steiger	**-1.25E-03**	5.82E-04	3.22E-02
	Weighted median - tophits	**-1.25E-03**	5.93E-04	3.56E-02
	Weighted mode - steiger	**-6.18E-04**	8.60E-04	4.81E-01
	Weighted mode - tophits	**-6.18E-04**	8.23E-04	4.62E-01
	Weighted mode (NOME) - steiger	**-6.59E-04**	1.11E-03	5.59E-01
	Weighted mode (NOME) - tophits	**-6.59E-04**	1.08E-03	5.47E-01
Diagnoses - main ICD10: I26 Pulmonary embolism (0.000359, 0.112)	Egger fixed effects - steiger	**-3.18E-03**	4.50E-04	1.01E-06
	Egger fixed effects - tophits	**-3.18E-03**	4.50E-04	1.01E-06
	Egger random effects - steiger	**-3.18E-03**	2.23E-03	1.69E-01
	Egger random effects - tophits	**-3.18E-03**	2.23E-03	1.69E-01
	IVW fixed effects - steiger	**2.49E-04**	1.66E-04	1.49E-01
	IVW fixed effects - tophits	**2.49E-04**	1.66E-04	1.49E-01
	IVW random effects - steiger	**2.49E-04**	8.91E-04	7.83E-01
	IVW random effects - tophits	**2.49E-04**	8.91E-04	7.83E-01
	Penalised median - steiger	**-4.10E-04**	5.42E-04	4.49E-01
	Penalised median - tophits	**-4.10E-04**	5.36E-04	4.44E-01
	Rucker mean (JK) - steiger	**-2.82E-03**	2.48E-03	2.70E-01
	Rucker mean (JK) - tophits	**-2.69E-03**	2.29E-03	2.54E-01
	Rucker median (JK) - steiger	**-2.83E-03**	3.41E-03	4.17E-01

	Rucker median (JK) - tophits	**-2.90E-03**	3.31E-03	3.90E-01
	Rucker point estimate - steiger	**-3.18E-03**	2.23E-03	1.69E-01
	Rucker point estimate - tophits	**-3.18E-03**	2.23E-03	1.69E-01
	Simple median - steiger	**-3.24E-04**	5.83E-04	5.78E-01
	Simple median - tophits	**-3.24E-04**	5.66E-04	5.67E-01
	Simple mode - steiger	**-9.89E-04**	7.79E-04	2.19E-01
	Simple mode - tophits	**-9.89E-04**	8.71E-04	2.70E-01
	Simple mode (NOME) - steiger	**-9.89E-04**	8.63E-04	2.65E-01
	Simple mode (NOME) - tophits	**-9.89E-04**	8.98E-04	2.84E-01
	Weighted median - steiger	**-3.98E-04**	5.31E-04	4.54E-01
	Weighted median - tophits	**-3.98E-04**	5.27E-04	4.50E-01
	Weighted mode - steiger	**-6.61E-04**	6.47E-04	3.19E-01
	Weighted mode - tophits	**-6.61E-04**	7.07E-04	3.61E-01
	Weighted mode (NOME) - steiger	**-7.27E-04**	6.63E-04	2.86E-01
	Weighted mode (NOME) - tophits	**-7.27E-04**	6.64E-04	2.87E-01
Diagnoses - main ICD10: I80 Phlebitis and thrombophlebitis (0.000395, 0.185)	Egger fixed effects - steiger	**-3.44E-03**	4.22E-04	5.94E-08
	Egger fixed effects - tophits	**-3.44E-03**	4.22E-04	5.94E-08
	Egger random effects - steiger	**-3.44E-03**	3.05E-03	2.72E-01
	Egger random effects - tophits	**-3.44E-03**	3.05E-03	2.72E-01
	IVW fixed effects - steiger	**4.22E-04**	1.57E-04	1.35E-02
	IVW fixed effects - tophits	**4.22E-04**	1.57E-04	1.35E-02
	IVW random effects - steiger	**4.22E-04**	1.18E-03	7.24E-01
	IVW random effects - tophits	**4.22E-04**	1.18E-03	7.24E-01
	Penalised median - steiger	**-3.47E-04**	6.57E-04	5.97E-01
	Penalised median - tophits	**-3.47E-04**	6.93E-04	6.17E-01
	Rucker mean (JK) - steiger	**-3.23E-03**	2.94E-03	2.84E-01
	Rucker mean (JK) - tophits	**-3.25E-03**	2.98E-03	2.87E-01
	Rucker median (JK) - steiger	**-2.99E-03**	3.59E-03	4.13E-01
	Rucker median (JK) - tophits	**-3.14E-03**	3.64E-03	3.97E-01
	Rucker point estimate - steiger	**-3.44E-03**	3.05E-03	2.72E-01

	Rucker point estimate - tophits	**-3.44E-03**	3.05E-03	2.72E-01
	Simple median - steiger	**-1.05E-04**	7.45E-04	8.87E-01
	Simple median - tophits	**-1.05E-04**	6.87E-04	8.78E-01
	Simple mode - steiger	**-6.30E-04**	1.24E-03	6.16E-01
	Simple mode - tophits	**-6.30E-04**	1.16E-03	5.94E-01
	Simple mode (NOME) - steiger	**-6.30E-04**	1.17E-03	5.97E-01
	Simple mode (NOME) - tophits	**-6.30E-04**	1.13E-03	5.82E-01
	Weighted median - steiger	**-3.65E-04**	6.87E-04	5.95E-01
	Weighted median - tophits	**-3.65E-04**	6.47E-04	5.73E-01
	Weighted mode - steiger	**-5.33E-04**	8.67E-04	5.45E-01
	Weighted mode - tophits	**-5.33E-04**	8.47E-04	5.35E-01
	Weighted mode (NOME) - steiger	**-5.33E-04**	9.30E-04	5.72E-01
	Weighted mode (NOME) - tophits	**-5.33E-04**	9.00E-04	5.60E-01
**Diagnoses - main ICD10: N97 Female infertility (0.000020, 0.772)**	Egger fixed effects - steiger	**1.20E-03**	8.02E-04	1.50E-01
	Egger fixed effects - tophits	**1.20E-03**	8.02E-04	1.50E-01
	Egger random effects - steiger	**1.20E-03**	6.00E-04	5.95E-02
	Egger random effects - tophits	**1.20E-03**	6.00E-04	5.95E-02
	IVW fixed effects - steiger	**1.39E-03**	3.15E-04	2.66E-04
	IVW fixed effects - tophits	**1.39E-03**	3.15E-04	2.66E-04
	IVW random effects - steiger	**1.39E-03**	2.25E-04	4.91E-06
	IVW random effects - tophits	**1.39E-03**	2.25E-04	4.91E-06
	Penalised median - steiger	**1.57E-03**	3.82E-04	3.75E-05
	Penalised median - tophits	**1.57E-03**	3.89E-04	5.25E-05
	Rucker mean (JK) - steiger	**1.40E-03**	2.47E-04	1.52E-05
	Rucker mean (JK) - tophits	**1.40E-03**	2.49E-04	1.66E-05
	Rucker median (JK) - steiger	**1.39E-03**	2.56E-04	2.52E-05
	Rucker median (JK) - tophits	**1.40E-03**	2.46E-04	1.41E-05
	Rucker point estimate - steiger	**1.39E-03**	3.15E-04	2.66E-04
	Rucker point estimate - tophits	**1.39E-03**	3.15E-04	2.66E-04
	Simple median - steiger	**1.69E-03**	4.03E-04	2.91E-05

	Simple median - tophits	**1.69E-03**	4.03E-04	2.85E-05
	Simple mode - steiger	**1.90E-03**	6.42E-04	7.83E-03
	Simple mode - tophits	**1.90E-03**	6.98E-04	1.33E-02
	Simple mode (NOME) - steiger	**1.90E-03**	6.60E-04	9.35E-03
	Simple mode (NOME) - tophits	**1.90E-03**	6.62E-04	9.60E-03
	Weighted median - steiger	**1.53E-03**	3.81E-04	6.12E-05
	Weighted median - tophits	**1.53E-03**	3.85E-04	7.34E-05
	Weighted mode - steiger	**1.84E-03**	6.62E-04	1.14E-02
	Weighted mode - tophits	**1.84E-03**	7.04E-04	1.64E-02
	Weighted mode (NOME) - steiger	**1.86E-03**	6.40E-04	8.69E-03
	Weighted mode (NOME) - tophits	**1.86E-03**	6.68E-04	1.14E-02
**Hearing aid user (-0.000145, 0.749)**	Egger fixed effects - steiger	**-3.73E-03**	6.23E-03	5.56E-01
	Egger fixed effects - tophits	**-3.73E-03**	6.23E-03	5.56E-01
	Egger random effects - steiger	**-3.73E-03**	3.61E-03	3.14E-01
	Egger random effects - tophits	**-3.73E-03**	3.61E-03	3.14E-01
	IVW fixed effects - steiger	**-5.15E-03**	2.48E-03	5.13E-02
	IVW fixed effects - tophits	**-5.15E-03**	2.48E-03	5.13E-02
	IVW random effects - steiger	**-5.15E-03**	1.38E-03	1.31E-03
	IVW random effects - tophits	**-5.15E-03**	1.38E-03	1.31E-03
	Penalised median - steiger	**-4.34E-03**	2.41E-03	7.11E-02
	Penalised median - tophits	**-4.34E-03**	2.46E-03	7.75E-02
	Rucker mean (JK) - steiger	**-5.19E-03**	1.14E-03	2.03E-04
	Rucker mean (JK) - tophits	**-5.16E-03**	1.09E-03	1.32E-04
	Rucker median (JK) - steiger	**-5.16E-03**	1.07E-03	1.04E-04
	Rucker median (JK) - tophits	**-5.12E-03**	1.03E-03	7.10E-05
	Rucker point estimate - steiger	**-5.15E-03**	2.48E-03	5.13E-02
	Rucker point estimate - tophits	**-5.15E-03**	2.48E-03	5.13E-02
	Simple median - steiger	**-4.67E-03**	2.63E-03	7.54E-02
	Simple median - tophits	**-4.67E-03**	2.51E-03	6.25E-02
	Simple mode - steiger	**-4.74E-03**	3.64E-03	2.08E-01

	Simple mode - tophits	**-4.74E-03**	3.74E-03	2.19E-01
	Simple mode (NOME) - steiger	**-4.74E-03**	3.46E-03	1.86E-01
	Simple mode (NOME) - tophits	**-4.74E-03**	3.89E-03	2.37E-01
	Weighted median - steiger	**-4.34E-03**	2.40E-03	7.07E-02
	Weighted median - tophits	**-4.34E-03**	2.42E-03	7.30E-02
	Weighted mode - steiger	**-4.65E-03**	3.03E-03	1.41E-01
	Weighted mode - tophits	**-4.65E-03**	3.07E-03	1.45E-01
	Weighted mode (NOME) - steiger	**-4.65E-03**	3.03E-03	1.41E-01
	Weighted mode (NOME) - tophits	**-4.65E-03**	3.07E-03	1.46E-01
**Hip circumference (-0.001832, 0.514)**	Egger fixed effects - steiger	**-4.63E-03**	9.66E-03	6.38E-01
	Egger fixed effects - tophits	**-4.63E-03**	9.66E-03	6.38E-01
	Egger random effects - steiger	**-4.63E-03**	2.88E-02	8.74E-01
	Egger random effects - tophits	**-4.63E-03**	2.88E-02	8.74E-01
	IVW fixed effects - steiger	**-2.23E-02**	3.87E-03	1.51E-05
	IVW fixed effects - tophits	**-2.23E-02**	3.87E-03	1.51E-05
	IVW random effects - steiger	**-2.23E-02**	1.12E-02	6.13E-02
	IVW random effects - tophits	**-2.23E-02**	1.12E-02	6.13E-02
	Penalised median - steiger	**-3.22E-02**	1.02E-02	1.61E-03
	Penalised median - tophits	**-3.22E-02**	9.75E-03	9.49E-04
	Rucker mean (JK) - steiger	**-6.31E-03**	2.80E-02	8.24E-01
	Rucker mean (JK) - tophits	**-6.47E-03**	2.76E-02	8.17E-01
	Rucker median (JK) - steiger	**-1.57E-02**	1.94E-02	4.30E-01
	Rucker median (JK) - tophits	**-1.64E-02**	1.72E-02	3.51E-01
	Rucker point estimate - steiger	**-2.23E-02**	1.12E-02	6.13E-02
	Rucker point estimate - tophits	**-2.23E-02**	1.12E-02	6.13E-02
	Simple median - steiger	**-2.76E-02**	1.08E-02	1.08E-02
	Simple median - tophits	**-2.76E-02**	1.02E-02	6.79E-03
	Simple mode - steiger	**-3.11E-02**	1.70E-02	8.26E-02
	Simple mode - tophits	**-3.11E-02**	1.73E-02	8.80E-02
	Simple mode (NOME) - steiger	**-3.11E-02**	1.62E-02	6.95E-02

	Simple mode (NOME) - tophits	**-3.11E-02**	1.63E-02	7.24E-02
	Weighted median - steiger	**-3.14E-02**	9.75E-03	1.26E-03
	Weighted median - tophits	**-3.14E-02**	9.88E-03	1.46E-03
	Weighted mode - steiger	**-3.02E-02**	1.16E-02	1.75E-02
	Weighted mode - tophits	**-3.02E-02**	1.17E-02	1.86E-02
	Weighted mode (NOME) - steiger	**-3.02E-02**	1.13E-02	1.54E-02
	Weighted mode (NOME) - tophits	**-3.02E-02**	1.06E-02	1.03E-02
**Impedance of arm (left) (0.004166, 0.165)**	Egger fixed effects - steiger	**-6.98E-02**	4.58E-03	4.07E-12
	Egger fixed effects - tophits	**-6.98E-02**	4.58E-03	4.07E-12
	Egger random effects - steiger	**-6.98E-02**	3.03E-02	3.28E-02
	Egger random effects - tophits	**-6.98E-02**	3.03E-02	3.28E-02
	IVW fixed effects - steiger	**-2.94E-02**	1.72E-03	2.22E-13
	IVW fixed effects - tophits	**-2.94E-02**	1.72E-03	2.22E-13
	IVW random effects - steiger	**-2.94E-02**	1.20E-02	2.42E-02
	IVW random effects - tophits	**-2.94E-02**	1.20E-02	2.42E-02
	Penalised median - steiger	**-2.50E-02**	8.14E-03	2.17E-03
	Penalised median - tophits	**-2.50E-02**	7.51E-03	8.84E-04
	Rucker mean (JK) - steiger	**-6.87E-02**	2.89E-02	2.74E-02
	Rucker mean (JK) - tophits	**-6.69E-02**	2.92E-02	3.30E-02
	Rucker median (JK) - steiger	**-6.74E-02**	2.99E-02	3.52E-02
	Rucker median (JK) - tophits	**-6.65E-02**	3.48E-02	7.01E-02
	Rucker point estimate - steiger	**-6.98E-02**	3.03E-02	3.28E-02
	Rucker point estimate - tophits	**-6.98E-02**	3.03E-02	3.28E-02
	Simple median - steiger	**-2.50E-02**	8.32E-03	2.64E-03
	Simple median - tophits	**-2.50E-02**	8.20E-03	2.30E-03
	Simple mode - steiger	**-2.90E-02**	1.36E-02	4.59E-02
	Simple mode - tophits	**-2.90E-02**	1.35E-02	4.36E-02
	Simple mode (NOME) - steiger	**-2.90E-02**	1.48E-02	6.33E-02
	Simple mode (NOME) - tophits	**-2.90E-02**	1.43E-02	5.54E-02
	Weighted median - steiger	**-2.51E-02**	7.84E-03	1.36E-03

	Weighted median - tophits	**-2.51E-02**	8.02E-03	1.76E-03
	Weighted mode - steiger	**-3.10E-02**	9.01E-03	2.62E-03
	Weighted mode - tophits	**-3.10E-02**	9.06E-03	2.73E-03
	Weighted mode (NOME) - steiger	**-3.19E-02**	9.62E-03	3.41E-03
	Weighted mode (NOME) - tophits	**-3.19E-02**	9.51E-03	3.12E-03
**Impedance of arm (right) (0.003967, 0.156)**	Egger fixed effects - steiger	**-6.32E-02**	4.73E-03	2.02E-11
	Egger fixed effects - tophits	**-6.32E-02**	4.73E-03	2.02E-11
	Egger random effects - steiger	**-6.32E-02**	2.89E-02	4.07E-02
	Egger random effects - tophits	**-6.32E-02**	2.89E-02	4.07E-02
	IVW fixed effects - steiger	**-2.40E-02**	1.79E-03	9.60E-12
	IVW fixed effects - tophits	**-2.40E-02**	1.79E-03	9.60E-12
	IVW random effects - steiger	**-2.40E-02**	1.16E-02	5.03E-02
	IVW random effects - tophits	**-2.40E-02**	1.16E-02	5.03E-02
	Penalised median - steiger	**-2.84E-02**	7.85E-03	2.99E-04
	Penalised median - tophits	**-2.84E-02**	7.80E-03	2.73E-04
	Rucker mean (JK) - steiger	**-6.16E-02**	2.73E-02	3.52E-02
	Rucker mean (JK) - tophits	**-5.93E-02**	2.68E-02	3.83E-02
	Rucker median (JK) - steiger	**-6.30E-02**	2.85E-02	3.81E-02
	Rucker median (JK) - tophits	**-6.17E-02**	3.00E-02	5.23E-02
	Rucker point estimate - steiger	**-6.32E-02**	2.89E-02	4.07E-02
	Rucker point estimate - tophits	**-6.32E-02**	2.89E-02	4.07E-02
	Simple median - steiger	**-2.51E-02**	8.07E-03	1.89E-03
	Simple median - tophits	**-2.51E-02**	8.10E-03	1.98E-03
	Simple mode - steiger	**-1.73E-02**	1.56E-02	2.81E-01
	Simple mode - tophits	**-1.73E-02**	1.51E-02	2.64E-01
	Simple mode (NOME) - steiger	**-1.73E-02**	1.62E-02	2.97E-01
	Simple mode (NOME) - tophits	**-1.73E-02**	1.64E-02	3.04E-01
	Weighted median - steiger	**-2.79E-02**	7.62E-03	2.54E-04
	Weighted median - tophits	**-2.79E-02**	7.71E-03	3.00E-04
	Weighted mode - steiger	**-2.76E-02**	9.46E-03	8.28E-03

	Weighted mode - tophits	**-2.76E-02**	9.41E-03	7.96E-03
	Weighted mode (NOME) - steiger	**-2.85E-02**	1.02E-02	1.06E-02
	Weighted mode (NOME) - tophits	**-2.85E-02**	9.49E-03	6.71E-03
**Impedance of whole body (0.003868, 0.077)**	Egger fixed effects - steiger	**-6.03E-02**	7.22E-03	5.97E-08
	Egger fixed effects - tophits	**-6.03E-02**	7.22E-03	5.97E-08
	Egger random effects - steiger	**-6.03E-02**	2.22E-02	1.34E-02
	Egger random effects - tophits	**-6.03E-02**	2.22E-02	1.34E-02
	IVW fixed effects - steiger	**-2.21E-02**	2.66E-03	4.43E-08
	IVW fixed effects - tophits	**-2.21E-02**	2.66E-03	4.43E-08
	IVW random effects - steiger	**-2.21E-02**	9.16E-03	2.50E-02
	IVW random effects - tophits	**-2.21E-02**	9.16E-03	2.50E-02
	Penalised median - steiger	**-2.28E-02**	8.05E-03	4.57E-03
	Penalised median - tophits	**-2.28E-02**	7.79E-03	3.38E-03
	Rucker mean (JK) - steiger	**-5.77E-02**	2.40E-02	2.56E-02
	Rucker mean (JK) - tophits	**-5.77E-02**	2.42E-02	2.64E-02
	Rucker median (JK) - steiger	**-5.93E-02**	2.15E-02	1.17E-02
	Rucker median (JK) - tophits	**-5.92E-02**	2.05E-02	8.80E-03
	Rucker point estimate - steiger	**-6.03E-02**	2.22E-02	1.34E-02
	Rucker point estimate - tophits	**-6.03E-02**	2.22E-02	1.34E-02
	Simple median - steiger	**-2.40E-02**	8.65E-03	5.54E-03
	Simple median - tophits	**-2.40E-02**	8.48E-03	4.63E-03
	Simple mode - steiger	**-2.71E-02**	1.42E-02	7.06E-02
	Simple mode - tophits	**-2.71E-02**	1.52E-02	8.85E-02
	Simple mode (NOME) - steiger	**-2.71E-02**	1.51E-02	8.68E-02
	Simple mode (NOME) - tophits	**-2.71E-02**	1.47E-02	8.03E-02
	Weighted median - steiger	**-2.38E-02**	8.16E-03	3.53E-03
	Weighted median - tophits	**-2.38E-02**	8.16E-03	3.51E-03
	Weighted mode - steiger	**-2.57E-02**	9.53E-03	1.37E-02
	Weighted mode - tophits	**-2.57E-02**	9.57E-03	1.40E-02
	Weighted mode (NOME) - steiger	**-2.64E-02**	9.83E-03	1.39E-02

	Weighted mode (NOME) - tophits	-2.64E-02	9.13E-03	8.80E-03
Non-cancer illness code, self-reported: deep venous thrombosis (dvt) (0.001213, 0.195)	Egger fixed effects - steiger	-9.46E-03	7.03E-04	1.70E-10
	Egger fixed effects - tophits	-9.46E-03	7.03E-04	1.70E-10
	Egger random effects - steiger	-9.46E-03	9.83E-03	3.49E-01
	Egger random effects - tophits	-9.46E-03	9.83E-03	3.49E-01
	IVW fixed effects - steiger	2.83E-03	2.58E-04	2.16E-09
	IVW fixed effects - tophits	2.83E-03	2.58E-04	2.16E-09
	IVW random effects - steiger	2.83E-03	3.77E-03	4.63E-01
	IVW random effects - tophits	2.83E-03	3.77E-03	4.63E-01
	Penalised median - steiger	-8.68E-05	1.35E-03	9.49E-01
	Penalised median - tophits	-8.68E-05	1.43E-03	9.52E-01
	Rucker mean (JK) - steiger	-8.98E-03	8.99E-03	3.31E-01
	Rucker mean (JK) - tophits	-9.50E-03	9.11E-03	3.11E-01
	Rucker median (JK) - steiger	-9.10E-03	1.26E-02	4.78E-01
	Rucker median (JK) - tophits	-9.09E-03	1.19E-02	4.56E-01
	Rucker point estimate - steiger	-9.46E-03	9.83E-03	3.49E-01
	Rucker point estimate - tophits	-9.46E-03	9.83E-03	3.49E-01
	Simple median - steiger	1.84E-03	1.50E-03	2.21E-01
	Simple median - tophits	1.84E-03	1.55E-03	2.37E-01
	Simple mode - steiger	2.27E-03	2.22E-03	3.21E-01
	Simple mode - tophits	2.27E-03	2.15E-03	3.04E-01
	Simple mode (NOME) - steiger	2.27E-03	2.12E-03	2.98E-01
	Simple mode (NOME) - tophits	2.27E-03	2.08E-03	2.90E-01
	Weighted median - steiger	-6.65E-05	1.37E-03	9.61E-01
	Weighted median - tophits	-6.65E-05	1.38E-03	9.62E-01
	Weighted mode - steiger	-7.06E-04	1.88E-03	7.11E-01
	Weighted mode - tophits	-7.06E-04	1.88E-03	7.11E-01
	Weighted mode (NOME) - steiger	-7.06E-04	1.79E-03	6.98E-01
	Weighted mode (NOME) - tophits	-7.06E-04	1.91E-03	7.15E-01
	Egger fixed effects - steiger	-1.26E-02	3.13E-03	8.75E-04

Non-cancer illness code, self-reported: high cholesterol (0.001806, 0.118)	Egger fixed effects - tophits	**-1.26E-02**	3.13E-03	8.75E-04
	Egger random effects - steiger	**-1.26E-02**	1.20E-02	3.08E-01
	Egger random effects - tophits	**-1.26E-02**	1.20E-02	3.08E-01
	IVW fixed effects - steiger	**5.72E-03**	1.12E-03	7.48E-05
	IVW fixed effects - tophits	**5.72E-03**	1.12E-03	7.48E-05
	IVW random effects - steiger	**5.72E-03**	4.71E-03	2.40E-01
	IVW random effects - tophits	**5.72E-03**	4.71E-03	2.40E-01
	Penalised median - steiger	**3.90E-03**	3.62E-03	2.82E-01
	Penalised median - tophits	**3.90E-03**	3.64E-03	2.84E-01
	Rucker mean (JK) - steiger	**-1.05E-02**	1.19E-02	3.90E-01
	Rucker mean (JK) - tophits	**-1.14E-02**	1.21E-02	3.60E-01
	Rucker median (JK) - steiger	**-1.16E-02**	1.30E-02	3.86E-01
	Rucker median (JK) - tophits	**-1.25E-02**	1.22E-02	3.19E-01
	Rucker point estimate - steiger	**-1.26E-02**	1.20E-02	3.08E-01
	Rucker point estimate - tophits	**-1.26E-02**	1.20E-02	3.08E-01
	Simple median - steiger	**4.83E-03**	4.05E-03	2.33E-01
	Simple median - tophits	**4.83E-03**	3.99E-03	2.25E-01
	Simple mode - steiger	**5.49E-03**	6.47E-03	4.07E-01
	Simple mode - tophits	**5.49E-03**	5.94E-03	3.67E-01
	Simple mode (NOME) - steiger	**5.49E-03**	6.05E-03	3.76E-01
	Simple mode (NOME) - tophits	**5.49E-03**	5.84E-03	3.60E-01
	Weighted median - steiger	**3.54E-03**	3.54E-03	3.17E-01
	Weighted median - tophits	**3.54E-03**	3.78E-03	3.49E-01
	Weighted mode - steiger	**4.11E-03**	4.34E-03	3.56E-01
	Weighted mode - tophits	**4.11E-03**	4.08E-03	3.27E-01
	Weighted mode (NOME) - steiger	**4.11E-03**	4.18E-03	3.38E-01
	Weighted mode (NOME) - tophits	**4.11E-03**	4.20E-03	3.40E-01
**Non-cancer illness code, self-reported: hyperthyroidism/thyrotoxicosis (0.000233, 0.278)**	Egger fixed effects - steiger	**-6.10E-03**	1.16E-03	6.10E-05
	Egger fixed effects - tophits	**-6.10E-03**	1.16E-03	6.10E-05
	Egger random effects - steiger	**-6.10E-03**	2.27E-03	1.58E-02

	Egger random effects - tophits	**-6.10E-03**	2.27E-03	1.58E-02
	IVW fixed effects - steiger	**-3.74E-03**	4.30E-04	7.49E-08
	IVW fixed effects - tophits	**-3.74E-03**	4.30E-04	7.49E-08
	IVW random effects - steiger	**-3.74E-03**	8.60E-04	3.89E-04
	IVW random effects - tophits	**-3.74E-03**	8.60E-04	3.89E-04
	Penalised median - steiger	**-3.45E-03**	9.57E-04	3.07E-04
	Penalised median - tophits	**-3.45E-03**	9.49E-04	2.70E-04
	Rucker mean (JK) - steiger	**-5.17E-03**	2.27E-03	3.52E-02
	Rucker mean (JK) - tophits	**-5.08E-03**	2.19E-03	3.24E-02
	Rucker median (JK) - steiger	**-4.31E-03**	1.60E-03	1.49E-02
	Rucker median (JK) - tophits	**-4.27E-03**	1.40E-03	6.94E-03
	Rucker point estimate - steiger	**-3.74E-03**	8.60E-04	3.89E-04
	Rucker point estimate - tophits	**-3.74E-03**	8.60E-04	3.89E-04
	Simple median - steiger	**-3.18E-03**	1.01E-03	1.63E-03
	Simple median - tophits	**-3.18E-03**	9.96E-04	1.40E-03
	Simple mode - steiger	**-2.76E-03**	1.67E-03	1.17E-01
	Simple mode - tophits	**-2.76E-03**	1.62E-03	1.06E-01
	Simple mode (NOME) - steiger	**-2.76E-03**	1.65E-03	1.12E-01
	Simple mode (NOME) - tophits	**-2.76E-03**	1.68E-03	1.17E-01
	Weighted median - steiger	**-3.44E-03**	9.36E-04	2.40E-04
	Weighted median - tophits	**-3.44E-03**	9.51E-04	3.00E-04
	Weighted mode - steiger	**-3.26E-03**	1.26E-03	1.86E-02
	Weighted mode - tophits	**-3.26E-03**	1.32E-03	2.36E-02
	Weighted mode (NOME) - steiger	**-3.38E-03**	1.32E-03	1.94E-02
	Weighted mode (NOME) - tophits	**-3.38E-03**	1.34E-03	2.18E-02
**Non-cancer illness code, self-reported: hypothyroidism/myxoedema (0.001270, 0.102)**	Egger fixed effects - steiger	**6.36E-03**	1.98E-03	5.11E-03
	Egger fixed effects - tophits	**6.36E-03**	1.98E-03	5.11E-03
	Egger random effects - steiger	**6.36E-03**	8.04E-03	4.40E-01
	Egger random effects - tophits	**6.36E-03**	8.04E-03	4.40E-01
	IVW fixed effects - steiger	**1.92E-02**	7.05E-04	4.26E-16

	IVW fixed effects - tophits	**1.92E-02**	7.05E-04	4.26E-16
	IVW random effects - steiger	**1.92E-02**	3.18E-03	1.01E-05
	IVW random effects - tophits	**1.92E-02**	3.18E-03	1.01E-05
	Penalised median - steiger	**1.98E-02**	2.34E-03	2.39E-17
	Penalised median - tophits	**1.98E-02**	2.57E-03	1.18E-14
	Rucker mean (JK) - steiger	**6.94E-03**	8.97E-03	4.49E-01
	Rucker mean (JK) - tophits	**6.79E-03**	8.81E-03	4.51E-01
	Rucker median (JK) - steiger	**7.09E-03**	9.73E-03	4.76E-01
	Rucker median (JK) - tophits	**6.93E-03**	8.56E-03	4.29E-01
	Rucker point estimate - steiger	**6.36E-03**	8.04E-03	4.40E-01
	Rucker point estimate - tophits	**6.36E-03**	8.04E-03	4.40E-01
	Simple median - steiger	**2.23E-02**	2.68E-03	8.44E-17
	Simple median - tophits	**2.23E-02**	2.71E-03	1.74E-16
	Simple mode - steiger	**2.31E-02**	3.76E-03	8.72E-06
	Simple mode - tophits	**2.31E-02**	4.14E-03	2.77E-05
	Simple mode (NOME) - steiger	**2.31E-02**	3.61E-03	5.11E-06
	Simple mode (NOME) - tophits	**2.31E-02**	3.62E-03	5.36E-06
	Weighted median - steiger	**1.89E-02**	2.69E-03	2.14E-12
	Weighted median - tophits	**1.89E-02**	2.55E-03	1.24E-13
	Weighted mode - steiger	**2.00E-02**	3.12E-03	4.86E-06
	Weighted mode - tophits	**2.00E-02**	3.15E-03	5.43E-06
	Weighted mode (NOME) - steiger	**2.02E-02**	2.89E-03	1.52E-06
	Weighted mode (NOME) - tophits	**2.02E-02**	2.65E-03	4.70E-07
Non-cancer illness code, self-reported: pulmonary embolism +/- dvt (0.000462, 0.287)	Egger fixed effects - steiger	**-3.56E-03**	5.30E-04	2.65E-06
	Egger fixed effects - tophits	**-3.56E-03**	5.30E-04	2.65E-06
	Egger random effects - steiger	**-3.56E-03**	4.40E-03	4.28E-01
	Egger random effects - tophits	**-3.56E-03**	4.40E-03	4.28E-01
	IVW fixed effects - steiger	**8.75E-04**	2.06E-04	4.27E-04
	IVW fixed effects - tophits	**8.75E-04**	2.06E-04	4.27E-04
	IVW random effects - steiger	**8.75E-04**	1.72E-03	6.18E-01

	IVW random effects - tophits	8.75E-04	1.72E-03	6.18E-01
	Penalised median - steiger	3.89E-04	8.41E-04	6.44E-01
	Penalised median - tophits	3.89E-04	8.47E-04	6.46E-01
	Rucker mean (JK) - steiger	-3.82E-03	3.98E-03	3.49E-01
	Rucker mean (JK) - tophits	-3.55E-03	3.69E-03	3.48E-01
	Rucker median (JK) - steiger	-3.40E-03	4.62E-03	4.70E-01
	Rucker median (JK) - tophits	-3.22E-03	4.30E-03	4.63E-01
	Rucker point estimate - steiger	-3.56E-03	4.40E-03	4.28E-01
	Rucker point estimate - tophits	-3.56E-03	4.40E-03	4.28E-01
	Simple median - steiger	-7.99E-05	8.71E-04	9.27E-01
	Simple median - tophits	-7.99E-05	8.85E-04	9.28E-01
	Simple mode - steiger	1.07E-04	1.33E-03	9.37E-01
	Simple mode - tophits	1.07E-04	1.26E-03	9.33E-01
	Simple mode (NOME) - steiger	1.07E-04	1.27E-03	9.34E-01
	Simple mode (NOME) - tophits	1.07E-04	1.25E-03	9.33E-01
	Weighted median - steiger	4.01E-04	8.95E-04	6.55E-01
	Weighted median - tophits	4.01E-04	8.38E-04	6.33E-01
	Weighted mode - steiger	3.67E-04	9.99E-04	7.17E-01
	Weighted mode - tophits	3.67E-04	9.92E-04	7.15E-01
	Weighted mode (NOME) - steiger	3.67E-04	9.74E-04	7.10E-01
	Weighted mode (NOME) - tophits	3.67E-04	9.68E-04	7.09E-01
Non-cancer illness code, self-reported: thyroid problem (not cancer) (-0.000003, 0.984)	Egger fixed effects - steiger	-1.27E-03	5.05E-04	2.14E-02
	Egger fixed effects - tophits	-1.27E-03	5.05E-04	2.14E-02
	Egger random effects - steiger	-1.27E-03	1.47E-03	3.97E-01
	Egger random effects - tophits	-1.27E-03	1.47E-03	3.97E-01
	IVW fixed effects - steiger	-1.30E-03	2.03E-04	3.71E-06
	IVW fixed effects - tophits	-1.30E-03	2.03E-04	3.71E-06
	IVW random effects - steiger	-1.30E-03	5.57E-04	3.06E-02
	IVW random effects - tophits	-1.30E-03	5.57E-04	3.06E-02
	Penalised median - steiger	-9.14E-04	5.11E-04	7.36E-02

	Penalised median - tophits	**-9.14E-04**	5.22E-04	7.99E-02
	Rucker mean (JK) - steiger	**-1.49E-03**	1.61E-03	3.66E-01
	Rucker mean (JK) - tophits	**-1.54E-03**	1.65E-03	3.64E-01
	Rucker median (JK) - steiger	**-1.27E-03**	7.52E-04	1.09E-01
	Rucker median (JK) - tophits	**-1.31E-03**	7.65E-04	1.03E-01
	Rucker point estimate - steiger	**-1.30E-03**	5.57E-04	3.06E-02
	Rucker point estimate - tophits	**-1.30E-03**	5.57E-04	3.06E-02
	Simple median - steiger	**-1.10E-03**	5.38E-04	4.10E-02
	Simple median - tophits	**-1.10E-03**	5.36E-04	4.04E-02
	Simple mode - steiger	**-9.23E-04**	7.15E-04	2.12E-01
	Simple mode - tophits	**-9.23E-04**	7.47E-04	2.32E-01
	Simple mode (NOME) - steiger	**-9.23E-04**	7.40E-04	2.27E-01
	Simple mode (NOME) - tophits	**-9.23E-04**	7.46E-04	2.31E-01
	Weighted median - steiger	**-8.85E-04**	4.98E-04	7.58E-02
	Weighted median - tophits	**-8.85E-04**	5.02E-04	7.78E-02
	Weighted mode - steiger	**-8.90E-04**	5.63E-04	1.31E-01
	Weighted mode - tophits	**-8.90E-04**	6.06E-04	1.59E-01
	Weighted mode (NOME) - steiger	**-8.90E-04**	5.83E-04	1.44E-01
	Weighted mode (NOME) - tophits	**-8.90E-04**	5.76E-04	1.39E-01
**Pulse rate, automated reading (-0.002068, 0.470)**	Egger fixed effects - steiger	**-1.69E-02**	1.03E-02	1.17E-01
	Egger fixed effects - tophits	**-1.69E-02**	1.03E-02	1.17E-01
	Egger random effects - steiger	**-1.69E-02**	2.94E-02	5.72E-01
	Egger random effects - tophits	**-1.69E-02**	2.94E-02	5.72E-01
	IVW fixed effects - steiger	**-3.68E-02**	4.10E-03	2.84E-08
	IVW fixed effects - tophits	**-3.68E-02**	4.10E-03	2.84E-08
	IVW random effects - steiger	**-3.68E-02**	1.14E-02	4.51E-03
	IVW random effects - tophits	**-3.68E-02**	1.14E-02	4.51E-03
	Penalised median - steiger	**-3.19E-02**	9.54E-03	8.24E-04
	Penalised median - tophits	**-3.19E-02**	1.00E-02	1.47E-03
	Rucker mean (JK) - steiger	**-2.22E-02**	2.21E-02	3.29E-01

	Rucker mean (JK) - tophits	**-2.12E-02**	2.34E-02	3.77E-01
	Rucker median (JK) - steiger	**-2.96E-02**	1.48E-02	6.05E-02
	Rucker median (JK) - tophits	**-2.98E-02**	1.63E-02	8.37E-02
	Rucker point estimate - steiger	**-3.68E-02**	1.14E-02	4.51E-03
	Rucker point estimate - tophits	**-3.68E-02**	1.14E-02	4.51E-03
	Simple median - steiger	**-3.53E-02**	1.06E-02	8.87E-04
	Simple median - tophits	**-3.53E-02**	1.03E-02	6.27E-04
	Simple mode - steiger	**-2.80E-02**	1.43E-02	6.56E-02
	Simple mode - tophits	**-2.80E-02**	1.40E-02	5.89E-02
	Simple mode (NOME) - steiger	**-2.80E-02**	1.42E-02	6.22E-02
	Simple mode (NOME) - tophits	**-2.80E-02**	1.45E-02	6.88E-02
	Weighted median - steiger	**-3.18E-02**	9.66E-03	9.90E-04
	Weighted median - tophits	**-3.18E-02**	9.73E-03	1.08E-03
	Weighted mode - steiger	**-2.97E-02**	9.70E-03	6.40E-03
	Weighted mode - tophits	**-2.97E-02**	9.94E-03	7.51E-03
	Weighted mode (NOME) - steiger	**-3.06E-02**	1.00E-02	6.59E-03
	Weighted mode (NOME) - tophits	**-3.06E-02**	1.09E-02	1.16E-02
**Standing height (-0.001012, 0.733)**	Egger fixed effects - steiger	**-1.29E-02**	4.69E-03	1.31E-02
	Egger fixed effects - tophits	**-1.29E-02**	4.69E-03	1.31E-02
	Egger random effects - steiger	**-1.29E-02**	3.05E-02	6.77E-01
	Egger random effects - tophits	**-1.29E-02**	3.05E-02	6.77E-01
	IVW fixed effects - steiger	**-2.26E-02**	1.89E-03	2.76E-10
	IVW fixed effects - tophits	**-2.26E-02**	1.89E-03	2.76E-10
	IVW random effects - steiger	**-2.26E-02**	1.18E-02	6.92E-02
	IVW random effects - tophits	**-2.26E-02**	1.18E-02	6.92E-02
	Penalised median - steiger	**-2.43E-02**	8.06E-03	2.57E-03
	Penalised median - tophits	**-2.43E-02**	8.03E-03	2.48E-03
	Rucker mean (JK) - steiger	**-9.39E-03**	2.92E-02	7.52E-01
	Rucker mean (JK) - tophits	**-1.07E-02**	3.00E-02	7.26E-01
	Rucker median (JK) - steiger	**-1.53E-02**	2.27E-02	5.10E-01

	Rucker median (JK) - tophits	-1.65E-02	2.29E-02	4.79E-01
	Rucker point estimate - steiger	-2.26E-02	1.18E-02	6.92E-02
	Rucker point estimate - tophits	-2.26E-02	1.18E-02	6.92E-02
	Simple median - steiger	-8.41E-03	8.57E-03	3.27E-01
	Simple median - tophits	-8.41E-03	8.65E-03	3.31E-01
	Simple mode - steiger	-7.74E-03	1.50E-02	6.11E-01
	Simple mode - tophits	-7.74E-03	1.52E-02	6.16E-01
	Simple mode (NOME) - steiger	-7.74E-03	1.45E-02	6.00E-01
	Simple mode (NOME) - tophits	-7.74E-03	1.51E-02	6.13E-01
	Weighted median - steiger	-2.31E-02	7.80E-03	3.04E-03
	Weighted median - tophits	-2.31E-02	8.21E-03	4.88E-03
	Weighted mode - steiger	-2.62E-02	8.85E-03	8.00E-03
	Weighted mode - tophits	-2.62E-02	9.28E-03	1.08E-02
	Weighted mode (NOME) - steiger	-2.71E-02	9.24E-03	8.65E-03
	Weighted mode (NOME) - tophits	-2.71E-02	9.07E-03	7.67E-03
**Treatment/medication code: levothyroxine sodium (0.000347, 0.564)**	Egger fixed effects - steiger	1.32E-02	2.04E-03	7.56E-06
	Egger fixed effects - tophits	1.32E-02	2.04E-03	7.56E-06
	Egger random effects - steiger	1.32E-02	6.50E-03	5.94E-02
	Egger random effects - tophits	1.32E-02	6.50E-03	5.94E-02
	IVW fixed effects - steiger	1.67E-02	8.25E-04	2.41E-13
	IVW fixed effects - tophits	1.67E-02	8.25E-04	2.41E-13
	IVW random effects - steiger	1.67E-02	2.53E-03	4.51E-06
	IVW random effects - tophits	1.67E-02	2.53E-03	4.51E-06
	Penalised median - steiger	1.08E-02	2.31E-03	2.88E-06
	Penalised median - tophits	1.08E-02	2.40E-03	6.62E-06
	Rucker mean (JK) - steiger	1.27E-02	8.43E-03	1.49E-01
	Rucker mean (JK) - tophits	1.29E-02	8.30E-03	1.39E-01
	Rucker median (JK) - steiger	1.51E-02	6.08E-03	2.41E-02
	Rucker median (JK) - tophits	1.48E-02	6.95E-03	4.78E-02
	Rucker point estimate - steiger	1.67E-02	2.53E-03	4.51E-06

	Rucker point estimate - tophits	**1.67E-02**	2.53E-03	4.51E-06
	Simple median - steiger	**1.41E-02**	2.61E-03	6.90E-08
	Simple median - tophits	**1.41E-02**	2.47E-03	1.18E-08
	Simple mode - steiger	**1.09E-02**	3.67E-03	8.86E-03
	Simple mode - tophits	**1.09E-02**	3.65E-03	8.56E-03
	Simple mode (NOME) - steiger	**1.09E-02**	3.84E-03	1.17E-02
	Simple mode (NOME) - tophits	**1.09E-02**	3.66E-03	8.70E-03
	Weighted median - steiger	**1.35E-02**	2.49E-03	5.75E-08
	Weighted median - tophits	**1.35E-02**	2.46E-03	4.28E-08
	Weighted mode - steiger	**1.07E-02**	2.97E-03	2.23E-03
	Weighted mode - tophits	**1.07E-02**	2.94E-03	2.03E-03
	Weighted mode (NOME) - steiger	**1.09E-02**	3.25E-03	3.85E-03
	Weighted mode (NOME) - tophits	**1.09E-02**	2.87E-03	1.47E-03
**Treatment/medication code: thyroxine product (-0.000155, 0.604)**	Egger fixed effects - steiger	**6.24E-03**	1.21E-03	9.69E-05
	Egger fixed effects - tophits	**6.24E-03**	1.21E-03	9.69E-05
	Egger random effects - steiger	**6.24E-03**	3.24E-03	7.22E-02
	Egger random effects - tophits	**6.24E-03**	3.24E-03	7.22E-02
	IVW fixed effects - steiger	**4.67E-03**	4.92E-04	3.34E-08
	IVW fixed effects - tophits	**4.67E-03**	4.92E-04	3.34E-08
	IVW random effects - steiger	**4.67E-03**	1.26E-03	1.77E-03
	IVW random effects - tophits	**4.67E-03**	1.26E-03	1.77E-03
	Penalised median - steiger	**3.19E-03**	1.29E-03	1.35E-02
	Penalised median - tophits	**3.19E-03**	1.31E-03	1.47E-02
	Rucker mean (JK) - steiger	**5.27E-03**	3.40E-03	1.40E-01
	Rucker mean (JK) - tophits	**5.10E-03**	3.37E-03	1.49E-01
	Rucker median (JK) - steiger	**4.82E-03**	2.28E-03	4.95E-02
	Rucker median (JK) - tophits	**4.70E-03**	2.18E-03	4.57E-02
	Rucker point estimate - steiger	**4.67E-03**	1.26E-03	1.77E-03
	Rucker point estimate - tophits	**4.67E-03**	1.26E-03	1.77E-03
	Simple median - steiger	**2.41E-03**	1.30E-03	6.36E-02

	Simple median - tophits	**2.41E-03**	1.30E-03	6.46E-02
	Simple mode - steiger	**2.55E-04**	2.45E-03	9.18E-01
	Simple mode - tophits	**2.55E-04**	2.24E-03	9.11E-01
	Simple mode (NOME) - steiger	**2.55E-04**	2.21E-03	9.09E-01
	Simple mode (NOME) - tophits	**2.55E-04**	2.16E-03	9.07E-01
	Weighted median - steiger	**4.53E-03**	1.28E-03	4.04E-04
	Weighted median - tophits	**4.53E-03**	1.26E-03	3.12E-04
	Weighted mode - steiger	**4.01E-03**	2.05E-03	6.77E-02
	Weighted mode - tophits	**4.01E-03**	2.03E-03	6.51E-02
	Weighted mode (NOME) - steiger	**4.15E-03**	2.12E-03	6.62E-02
	Weighted mode (NOME) - tophits	**4.15E-03**	2.10E-03	6.48E-02
**Trunk fat percentage (0.003114, 0.242)**	Egger fixed effects - steiger	**-4.83E-02**	8.65E-03	2.23E-05
	Egger fixed effects - tophits	**-4.83E-02**	8.65E-03	2.23E-05
	Egger random effects - steiger	**-4.83E-02**	2.72E-02	9.17E-02
	Egger random effects - tophits	**-4.83E-02**	2.72E-02	9.17E-02
	IVW fixed effects - steiger	**-1.80E-02**	3.31E-03	2.48E-05
	IVW fixed effects - tophits	**-1.80E-02**	3.31E-03	2.48E-05
	IVW random effects - steiger	**-1.80E-02**	1.06E-02	1.05E-01
	IVW random effects - tophits	**-1.80E-02**	1.06E-02	1.05E-01
	Penalised median - steiger	**-3.14E-02**	9.17E-03	6.07E-04
	Penalised median - tophits	**-3.14E-02**	9.03E-03	5.01E-04
	Rucker mean (JK) - steiger	**-4.03E-02**	2.80E-02	1.67E-01
	Rucker mean (JK) - tophits	**-4.18E-02**	2.83E-02	1.55E-01
	Rucker median (JK) - steiger	**-3.16E-02**	2.90E-02	2.89E-01
	Rucker median (JK) - tophits	**-3.42E-02**	2.95E-02	2.60E-01
	Rucker point estimate - steiger	**-4.83E-02**	2.72E-02	9.17E-02
	Rucker point estimate - tophits	**-4.83E-02**	2.72E-02	9.17E-02
	Simple median - steiger	**-1.57E-02**	1.12E-02	1.62E-01
	Simple median - tophits	**-1.57E-02**	1.13E-02	1.65E-01
	Simple mode - steiger	**-2.93E-02**	1.57E-02	7.61E-02

	Simple mode - tophits	-2.93E-02	1.44E-02	5.48E-02
	Simple mode (NOME) - steiger	-2.93E-02	1.64E-02	8.88E-02
	Simple mode (NOME) - tophits	-2.93E-02	1.53E-02	6.90E-02
	Weighted median - steiger	-3.13E-02	9.13E-03	6.05E-04
	Weighted median - tophits	-3.13E-02	8.87E-03	4.17E-04
	Weighted mode - steiger	-3.98E-02	1.09E-02	1.56E-03
	Weighted mode - tophits	-3.98E-02	1.01E-02	8.29E-04
	Weighted mode (NOME) - steiger	-3.98E-02	9.86E-03	6.39E-04
	Weighted mode (NOME) - tophits	-3.98E-02	1.08E-02	1.47E-03
Trunk fat-free mass (-0.003005, 0.173)	Egger fixed effects - steiger	3.15E-02	4.94E-03	4.07E-06
	Egger fixed effects - tophits	3.15E-02	4.94E-03	4.07E-06
	Egger random effects - steiger	3.15E-02	2.24E-02	1.75E-01
	Egger random effects - tophits	3.15E-02	2.24E-02	1.75E-01
	IVW fixed effects - steiger	2.33E-03	1.86E-03	2.26E-01
	IVW fixed effects - tophits	2.33E-03	1.86E-03	2.26E-01
	IVW random effects - steiger	2.33E-03	8.86E-03	7.95E-01
	IVW random effects - tophits	2.33E-03	8.86E-03	7.95E-01
	Penalised median - steiger	-7.18E-03	6.60E-03	2.77E-01
	Penalised median - tophits	-7.18E-03	6.37E-03	2.59E-01
	Rucker mean (JK) - steiger	3.21E-02	2.44E-02	2.04E-01
	Rucker mean (JK) - tophits	3.21E-02	2.50E-02	2.13E-01
	Rucker median (JK) - steiger	3.14E-02	2.67E-02	2.54E-01
	Rucker median (JK) - tophits	3.16E-02	2.76E-02	2.66E-01
	Rucker point estimate - steiger	3.15E-02	2.24E-02	1.75E-01
	Rucker point estimate - tophits	3.15E-02	2.24E-02	1.75E-01
	Simple median - steiger	3.97E-03	7.34E-03	5.89E-01
	Simple median - tophits	3.97E-03	7.43E-03	5.93E-01
	Simple mode - steiger	-1.72E-04	1.29E-02	9.90E-01
	Simple mode - tophits	-1.72E-04	1.31E-02	9.90E-01
	Simple mode (NOME) - steiger	-1.72E-04	1.32E-02	9.90E-01

	Simple mode (NOME) - tophits	**-1.72E-04**	1.32E-02	9.90E-01
	Weighted median - steiger	**-2.58E-03**	6.80E-03	7.05E-01
	Weighted median - tophits	**-2.58E-03**	6.77E-03	7.03E-01
	Weighted mode - steiger	**-6.47E-03**	7.42E-03	3.94E-01
	Weighted mode - tophits	**-6.47E-03**	7.18E-03	3.78E-01
	Weighted mode (NOME) - steiger	**-6.47E-03**	8.11E-03	4.35E-01
	Weighted mode (NOME) - tophits	**-6.47E-03**	7.03E-03	3.69E-01
Trunk predicted mass (-0.003006, 0.170)	Egger fixed effects - steiger	**3.17E-02**	4.95E-03	3.89E-06
	Egger fixed effects - tophits	**3.17E-02**	4.95E-03	3.89E-06
	Egger random effects - steiger	**3.17E-02**	2.22E-02	1.69E-01
	Egger random effects - tophits	**3.17E-02**	2.22E-02	1.69E-01
	IVW fixed effects - steiger	**2.50E-03**	1.87E-03	1.95E-01
	IVW fixed effects - tophits	**2.50E-03**	1.87E-03	1.95E-01
	IVW random effects - steiger	**2.50E-03**	8.79E-03	7.79E-01
	IVW random effects - tophits	**2.50E-03**	8.79E-03	7.79E-01
	Penalised median - steiger	**-6.11E-03**	6.52E-03	3.49E-01
	Penalised median - tophits	**-6.11E-03**	6.62E-03	3.56E-01
	Rucker mean (JK) - steiger	**3.14E-02**	2.39E-02	2.03E-01
	Rucker mean (JK) - tophits	**3.27E-02**	2.48E-02	2.02E-01
	Rucker median (JK) - steiger	**3.20E-02**	2.59E-02	2.30E-01
	Rucker median (JK) - tophits	**3.17E-02**	2.72E-02	2.58E-01
	Rucker point estimate - steiger	**3.17E-02**	2.22E-02	1.69E-01
	Rucker point estimate - tophits	**3.17E-02**	2.22E-02	1.69E-01
	Simple median - steiger	**6.77E-03**	7.58E-03	3.72E-01
	Simple median - tophits	**6.77E-03**	7.53E-03	3.69E-01
	Simple mode - steiger	**1.96E-03**	1.24E-02	8.76E-01
	Simple mode - tophits	**1.96E-03**	1.22E-02	8.74E-01
	Simple mode (NOME) - steiger	**1.96E-03**	1.35E-02	8.86E-01
	Simple mode (NOME) - tophits	**1.96E-03**	1.35E-02	8.86E-01
	Weighted median - steiger	**-1.72E-03**	6.67E-03	7.97E-01

	Weighted median - tophits	**-1.72E-03**	6.88E-03	8.03E-01
	Weighted mode - steiger	**-6.02E-03**	7.34E-03	4.22E-01
	Weighted mode - tophits	**-6.02E-03**	7.53E-03	4.34E-01
	Weighted mode (NOME) - steiger	**-6.02E-03**	8.54E-03	4.89E-01
	Weighted mode (NOME) - tophits	**-6.02E-03**	7.27E-03	4.18E-01

Table S6: Genomic risk loci and independent variants (LD $r^2 < 0.1$) identified in hyperthyroidism GWAS.

Genomic Risk Locus	rsID	CHR	BP	Alleles	P	Consequence	SYMBOL	Region Biotype
1	rs2476601	1	114377568	A/G	3.15E-14	missense_variant	*PTPN22*	protein_coding
2	rs3087243	2	204738919	A/G	3.80E-21	downstream_gene_variant	*CTLA4*	protein_coding
3	rs34158769	6	26336572	A/G	4.60E-19	regulatory_region_variant	-	promoter_flanking_region
3	rs1796518	6	26388672	C/T	9.38E-09	intron_variant	*BTN2A2*	protein_coding
3	rs13217620	6	27653120	C/T	7.75E-27	intergenic_variant	-	-
3	rs238883	6	29346226	A/G	1.22E-08	intron_variant	*OR5V1*	protein_coding
3	rs969931	6	29494897	G/T	4.30E-13	upstream_gene_variant	*LINC01015*	antisense
3	rs1233396	6	29546799	A/G	1.78E-36	intron_variant	*GABBR1*	protein_coding
3	rs29232	6	29611431	C/T	4.53E-08	intergenic_variant	-	-
4	rs34859217	6	33196397	A/C	4.15E-35	intergenic_variant	-	-
4	rs80031348	6	33956364	A/G	3.09E-11	regulatory_region_variant	-	TF_binding_site
5	rs2160215	14	81461472	C/T	8.90E-31	intron_variant	*TSHR*	protein_coding

Table S7: Genomic risk loci and independent variants (LD $r^2 < 0.1$) identified in hypothyroidism GWAS.

Genomic Risk Locus	rsID	CHR	BP	Alleles	P	Consequence	Gene	Region Biotype
1	rs4081335	1	1140504	C/T	3.55E-08	intron_variant	*TNFRSF18*	protein_coding
2	rs12089835	1	19771438	C/T	8.87E-09	intron_variant	*CAPZB*	protein_coding
2	rs6426808	1	19835415	A/G	3.13E-10	upstream_gene_variant	*RNU4-28P*	snRNA
3	rs17484960	1	108337108	A/G	5.99E-10	intron_variant	*VAV3*	protein_coding
3	rs17020149	1	108375289	A/G	1.35E-36	intron_variant	*VAV3*	protein_coding
4	rs12061333	1	113860779	A/C	8.68E-14	intergenic_variant	-	-
4	rs72685697	1	113869057	C/T	2.71E-09	intergenic_variant	-	-
4	rs12128454	1	113910209	C/T	3.11E-16	intergenic_variant	-	-
4	rs4839324	1	114071617	A/G	2.30E-09	intron_variant	*MAGI3*	protein_coding
4	rs115985944	1	114154300	C/T	6.22E-17	intron_variant	*MAGI3*	protein_coding
4	rs3789599	1	114323318	C/T	9.58E-10	intron_variant	*RSBN1*	protein_coding
4	rs2476601	1	114377568	A/G	3.49E-124	missense_variant	*PTPN22*	protein_coding
4	rs1310183	1	114439522	C/T	3.49E-18	intron_variant	*AP4B1*	protein_coding
4	rs36064592	1	114462662	A/G	6.87E-19	downstream_gene_variant	*HIPK1-AS1*	retained_intron
4	rs139269023	1	114559407	A/T	6.49E-11	intron_variant,non_coding_transcript_variant	*OLFML3*	processed_transcript
4	rs61819212	1	114589211	A/G	1.37E-10	intergenic_variant	-	-
4	rs138244061	1	114610124	C/T	1.38E-10	intergenic_variant	-	-
5	rs926103	1	156784982	C/T	4.92E-08	missense_variant	*SH2D2A*	protein_coding
6	rs12117927	1	236629134	A/C	8.18E-10	intron_variant	*EDARADD*	protein_coding
7	rs11675342	2	1407628	C/T	1.67E-18	intron_variant,non_coding_transcript_variant	*TPO*	processed_transcript
8	rs1534430	2	12644736	C/T	3.99E-14	intron_variant,non_coding_transcript_variant	*MIR3681HG*	lincRNA
9	rs13399762	2	55880308	A/G	4.25E-09	intron_variant,NMD_transcript_variant	*PNPT1*	nonsense_mediated_decay
10	rs41368446	2	162998113	G/T	3.82E-09	downstream_gene_variant	*GCG*	protein_coding
10	rs2111485	2	163110536	A/G	7.84E-13	intergenic_variant	-	-
10	rs78456138	2	163132346	C/T	1.46E-08	intron_variant	*IFIH1*	protein_coding
11	rs1155061	2	191566381	A/G	5.67E-10	intron_variant,non_coding_transcript_variant	*AC006460.1*	antisense
11	rs149218447	2	191963305	A/G	1.09E-08	intron_variant	*STAT4*	protein_coding

11	rs7582694	2	191970120	C/G	2.61E-29	intron_variant	*STAT4*	protein_coding
12	rs2160316	2	202885167	C/T	4.14E-08	intergenic_variant	-	-
13	rs11678714	2	204633514	A/T	7.43E-12	upstream_gene_variant	*KRT18P39*	processed_pseudogene
13	rs11571297	2	204745003	C/T	5.34E-61	intergenic_variant	-	-
13	rs79570815	2	204748650	A/G	1.76E-09	intergenic_variant	-	-
13	rs77130284	2	204805211	C/T	3.86E-17	intron_variant	*ICOS*	protein_coding
13	rs11673985	2	204986334	C/T	2.64E-10	intergenic_variant	-	-
14	rs7649344	3	37006396	C/T	1.23E-08	downstream_gene_variant	*RNU6ATAC4P*	snRNA
15	rs11706511	3	39344438	A/G	2.47E-09	regulatory_region_variant	-	CTCF_binding_site
16	rs13090803	3	105934953	G/T	2.09E-15	intergenic_variant	-	-
17	rs1599795	3	119243855	A/T	8.17E-09	downstream_gene_variant	*TIMMDC1*	nonsense_mediated_decay
18	rs113229608	3	121743159	A/C	1.17E-09	upstream_gene_variant	*ILDR1*	protein_coding
19	rs9815073	3	188115682	A/C	1.94E-35	intron_variant	*LPP*	protein_coding
20	rs4276275	4	10723685	C/T	5.84E-11	intergenic_variant	-	-
21	rs7441808	4	26090375	A/G	3.53E-10	intron_variant,non_coding_transcript_variant	*LINC02357*	lincRNA
22	rs4444866	4	40307533	C/T	2.85E-08	regulatory_region_variant	-	promoter_flanking_region
23	rs6833591	4	123546282	A/G	6.10E-09	upstream_gene_variant	*IL21*	protein_coding
24	rs13145888	4	149634572	C/T	2.45E-21	intergenic_variant	-	-
25	rs35782497	4	187001230	A/G	5.77E-12	intron_variant	*TLR3*	protein_coding
26	rs10036386	5	76543603	C/T	1.75E-09	intron_variant	*PDE8B*	protein_coding
27	rs28157	5	102595837	G/T	4.16E-10	intron_variant	*C5orf30*	protein_coding
28	rs114378220	5	110566360	C/T	2.58E-08	intron_variant	*CAMK4*	protein_coding
29	rs244672	5	133419283	C/T	4.84E-09	upstream_gene_variant	*AC008608.1*	processed_pseudogene
30	rs4263621	6	435471	A/G	1.48E-08	intergenic_variant	-	-
31	rs72834698	6	26176517	A/G	4.27E-08	downstream_gene_variant	*HIST1H2BD*	protein_coding
31	rs6939978	6	26328462	C/G	5.81E-12	regulatory_region_variant	-	promoter_flanking_region
31	rs200949	6	27835435	A/G	2.95E-12	upstream_gene_variant	*HIST1H1B*	protein_coding
31	rs17404424	6	29275298	A/G	4.99E-08	missense_variant	*OR14J1*	protein_coding
31	rs17184142	6	29475761	C/T	2.52E-11	intron_variant,non_coding_transcript_variant	*AL662860.1*	lincRNA
31	rs1233478	6	29477821	G/T	1.57E-15	intron_variant,non_coding_transcript_variant	*AL662860.1*	lincRNA

31	rs1233410	6	29515494	C/T	2.37E-15	upstream_gene_variant	*OR2I1P*	protein_coding
31	rs362520	6	29558364	A/G	4.71E-08	intron_variant	*GABBR1*	protein_coding
31	rs29232	6	29611431	C/T	8.38E-09	intergenic_variant	-	-
32	rs2854027	6	33180045	C/T	7.06E-10	downstream_gene_variant	*MIR219A1*	miRNA
32	rs213211	6	33187355	C/T	1.94E-20	downstream_gene_variant	*ZNF70P1*	processed_pseudogene
32	rs1704995	6	33187688	C/T	1.03E-31	downstream_gene_variant	*ZNF70P1*	processed_pseudogene
32	rs147497774	6	33596031	C/G	1.30E-08	intron_variant	*ITPR3*	protein_coding
33	rs9380522	6	35537964	C/T	5.12E-09	downstream_gene_variant	*FKBP5*	protein_coding
34	rs654537	6	90990050	A/G	2.13E-29	intron_variant	*BACH2*	protein_coding
34	rs755178	6	90999463	A/G	3.76E-16	intron_variant	*BACH2*	protein_coding
34	rs911409	6	91022755	C/G	3.70E-08	downstream_gene_variant	*MIR4464*	miRNA
35	rs761357	6	135902599	A/T	6.81E-10	intron_variant,non_coding_transcript_variant	*LINC00271*	lincRNA
36	rs9497965	6	148521292	C/T	5.19E-12	intergenic_variant	-	-
37	rs9295378	6	167334067	C/G	1.30E-08	intron_variant	*AL159163.1*	protein_coding
37	rs933243	6	167403873	A/C	3.90E-24	intron_variant,non_coding_transcript_variant	*Z94721.1*	antisense
38	rs60600003	7	37382465	G/T	7.19E-09	intron_variant	*ELMO1*	protein_coding
39	rs221786	7	100266081	C/T	1.53E-09	intergenic_variant	-	-
40	rs2921053	8	8319963	C/G	3.34E-19	upstream_gene_variant	*AC103957.2*	lincRNA
40	rs11786541	8	9789549	G/T	9.26E-10	intergenic_variant	-	-
41	rs1531576	8	11338146	A/G	2.90E-12	intron_variant,non_coding_transcript_variant	*AF131216.5*	lincRNA
42	rs6992869	8	61395832	C/T	2.56E-08	intron_variant,non_coding_transcript_variant	*LINC01301*	lincRNA
43	rs1032129	8	119951900	A/C	2.67E-08	intron_variant	*TNFRSF11B*	protein_coding
44	rs2445610	8	128197088	A/G	3.55E-11	downstream_gene_variant	*CASC19*	retained_intron
45	rs10956412	8	129162497	A/C	5.84E-13	downstream_gene_variant	*MIR1208*	miRNA
46	rs1561924	8	129569371	A/G	9.68E-10	downstream_gene_variant	*LINC00824*	lincRNA
47	rs853303	8	133932818	A/G	2.30E-12	intron_variant	*TG*	protein_coding
47	rs7006080	8	134214224	G/T	1.66E-09	intron_variant	*WISP1*	protein_coding
48	rs11783023	8	141639262	C/T	1.06E-08	intron_variant	*AGO2*	protein_coding
49	rs911760	9	5438435	A/C	5.10E-10	upstream_gene_variant	*PLGRKT*	protein_coding
50	rs2123340	9	21589041	A/G	7.76E-12	intergenic_variant	-	-

50	rs13285496	9	21654957	C/G	6.34E-09	intergenic_variant	-	-
51	rs10817778	9	100330004	A/G	9.94E-13	intron_variant	*TMOD1*	protein_coding
51	rs2805791	9	100354298	A/T	3.62E-12	intron_variant	*TMOD1*	protein_coding
51	rs2808667	9	100442806	C/T	7.64E-11	intron_variant	*XPA*	protein_coding
51	rs12552830	9	100517724	C/G	1.98E-10	intergenic_variant	-	-
51	rs62573972	9	100532265	A/G	2.46E-11	intergenic_variant	-	-
51	rs925489	9	100546600	C/T	1.52E-71	intergenic_variant	-	-
51	rs76293252	9	100550482	A/G	6.63E-11	intergenic_variant	-	-
51	rs7870125	9	100650737	C/G	2.21E-20	intergenic_variant	-	-
51	rs7044744	9	100769276	A/C	7.97E-12	intron_variant	*ANP32B*	protein_coding
51	rs7849424	9	100876668	C/T	2.00E-09	intron_variant	*TRIM14*	protein_coding
52	rs11256442	10	6079344	C/T	3.84E-11	intron_variant	*IL2RA*	protein_coding
52	rs7090530	10	6110875	A/C	2.94E-15	downstream_gene_variant	*RPL32P23*	processed_pseudogene
52	rs41295121	10	6129643	C/T	2.97E-13	upstream_gene_variant	*RBM17*	protein_coding
52	rs11258303	10	6405534	A/C	5.59E-10	regulatory_region_variant	-	open_chromatin_region
53	rs71508903	10	63779871	C/T	3.21E-21	intron_variant	*ARID5B*	protein_coding
53	rs74654624	10	63906102	C/G	8.86E-09	intergenic_variant	-	-
53	rs148672683	10	63910344	C/T	3.53E-08	intergenic_variant	-	-
53	rs7905731	10	64018472	C/T	2.66E-09	intron_variant	*RTKN2*	protein_coding
54	rs534182	10	89810979	C/T	2.70E-08	upstream_gene_variant	*MED6P1*	processed_pseudogene
55	rs10748781	10	101283330	A/C	5.62E-11	upstream_gene_variant	*AL513542.1*	lincRNA
56	rs3850765	10	124139910	C/T	2.76E-09	intron_variant	*PLEKHA1*	protein_coding
57	rs11033031	11	35244643	A/C	2.40E-09	intron_variant	*CD44*	protein_coding
57	rs736374	11	35266944	A/G	2.48E-14	intergenic_variant	-	-
58	rs4409785	11	95311422	C/T	4.31E-21	intergenic_variant	-	-
59	rs11052877	12	9905690	A/G	9.31E-27	3_prime_UTR_variant	*CD69*	protein_coding
60	rs705702	12	56390636	A/G	1.96E-11	upstream_gene_variant	*SUOX*	protein_coding
61	rs12578952	12	103755097	A/G	2.03E-08	intron_variant	*C12orf42*	protein_coding
61	rs12582330	12	103892941	G/T	1.10E-12	upstream_gene_variant	*C12orf42*	protein_coding
62	rs12184466	12	111281636	C/T	3.55E-09	upstream_gene_variant	*CCDC63*	protein_coding

62	rs10082818	12	111663177	C/G	1.14E-09	intron_variant	*CUX2*	protein_coding
62	rs79065604	12	111877979	A/T	1.03E-10	intron_variant	*SH2B3*	protein_coding
62	rs3184504	12	111884608	C/T	1.10E-81	missense_variant	*SH2B3*	protein_coding
62	rs615134	12	112003695	A/T	1.29E-11	intron_variant	*ATXN2*	protein_coding
62	rs117532831	12	112037450	A/G	2.10E-10	5_prime_UTR_variant	*ATXN2*	protein_coding
62	rs6490291	12	112177775	A/T	2.82E-08	intron_variant	*ACAD10*	protein_coding
62	rs11066320	12	112906415	A/G	4.60E-53	intron_variant	*PTPN11*	protein_coding
62	rs232935	12	113115628	C/T	4.84E-08	intron_variant	*RPH3A*	protein_coding
62	rs6489855	12	113164767	A/G	2.55E-09	intron_variant	*RPH3A*	protein_coding
63	rs7318364	13	24772892	C/T	4.68E-14	intron_variant	*SPATA13*	protein_coding
63	rs9511151	13	24786576	A/G	3.38E-27	intron_variant	*SPATA13*	protein_coding
64	rs76428106	13	28604007	C/T	4.25E-30	intron_variant	*FLT3*	protein_coding
65	rs66749983	13	43063831	A/T	1.39E-10	downstream_gene_variant	*LINC02341*	lincRNA
65	rs116926994	13	43077245	A/G	4.81E-08	intergenic_variant	-	-
66	rs8008961	14	68752643	C/T	5.54E-09	intron_variant,non_coding_transcript_variant	*RAD51B*	processed_transcript
67	rs1045853	14	106208086	A/C	1.10E-09	missense_variant	*IGHG1*	IG_C_gene
68	rs8043085	15	38828140	G/T	4.47E-12	intron_variant	*RASGRP1*	protein_coding
69	rs142997491	16	50729820	A/G	2.81E-10	upstream_gene_variant	*NOD2*	protein_coding
70	rs13333582	16	67549547	C/T	1.80E-08	upstream_gene_variant	*RIPOR1*	protein_coding
71	rs8054578	16	79316815	A/G	8.95E-09	intergenic_variant	-	-
72	rs35703946	16	86021505	A/G	4.89E-08	downstream_gene_variant	*AC092723.1*	lincRNA
73	rs61759532	17	7240391	C/T	1.26E-11	intron_variant	*ACAP1*	protein_coding
74	rs1088898	17	8876505	G/T	4.22E-08	intron_variant,non_coding_transcript_variant	*AC002091.1*	lincRNA
75	rs12325861	17	40289412	C/T	3.08E-12	intron_variant	*RAB5C*	protein_coding
76	rs62076510	17	45518583	G/T	3.46E-13	3_prime_UTR_variant	*EFCAB13*	protein_coding
77	rs28665408	18	67516846	A/C	7.63E-11	downstream_gene_variant	*CD226*	protein_coding
78	rs10424978	19	4837557	A/C	7.21E-15	downstream_gene_variant	*PLIN3*	protein_coding
79	rs7248104	19	7224431	A/G	1.14E-09	intron_variant	*INSR*	protein_coding
80	rs34536443	19	10463118	C/G	6.69E-11	missense_variant	*TYK2*	protein_coding
81	rs1549142	19	18383794	C/T	3.90E-11	intron_variant	*IQCN*	protein_coding

82	rs17272847	19	49979398	A/G	1.61E-08	splice_region_variant,intron_variant	*FLT3LG*	protein_coding
82	rs12980063	19	50196992	A/G	1.74E-10	intron_variant,non_coding_transcript_variant	*CPT1C*	retained_intron
83	rs2745803	20	17859706	A/G	9.31E-09	regulatory_region_variant	-	CTCF_binding_site
84	rs2242830	21	16801277	C/G	6.60E-10	intron_variant,non_coding_transcript_variant	*AJ009632.2*	lincRNA
85	rs2412973	22	30529631	A/C	9.80E-11	intron_variant	*HORMAD2*	protein_coding
86	rs229540	22	37591290	G/T	8.83E-22	intron_variant,non_coding_transcript_variant	*C1QTNF6*	processed_transcript
86	rs6000632	22	37644115	C/T	3.88E-08	upstream_gene_variant	*RAC2*	protein_coding

Table S8: Genetic correlation of hyperthyroidism and hypothyroidism calculated with respect to 1578 phenotypic traits.

Description	Hyperthyroidism		Hypothyroidism		Difference	
	rg	*p*	*rg*	*p*	*z*	*p*
Treatment/medication code: levothyroxine sodium	0.49	5.72E-07	1.00	4.50E-308	-5.24	1.64E-07
Taking other prescription medications	0.16	0.015	0.49	5.09E-52	-4.38	1.18E-05
Types of physical activity in last 4 weeks: Walking for pleasure (not as a means of transport)	0.18	0.014	-0.12	0.003	3.57	3.61E-04
Mouth/teeth dental problems: Mouth ulcers	-0.29	6.00E-04	0.03	0.513	-3.31	9.17E-04
Long-standing illness, disability or infirmity	0.14	0.026	0.36	8.63E-30	-3.21	0.001
Pulse rate	0.19	0.006	-0.05	0.121	3.16	0.002
Pulse rate, automated reading	0.11	0.024	-0.06	0.015	3.10	0.002
Diagnoses - main ICD10: R07 Pain in throat and chest	-0.13	0.228	0.23	1.10E-06	-3.06	0.002
Treatment/medication code: thyroxine product	0.51	0.002	1.03	2.15E-52	-2.96	0.003
Fed-up feelings	-0.06	0.417	0.18	1.66E-05	-2.92	0.004
Illness, injury, bereavement, stress in last 2 years: None of the above	0.06	0.488	-0.21	1.42E-07	2.89	0.004
Non-cancer illness code, self-reported: acne/acne vulgaris	0.49	0.091	-0.47	0.011	2.81	0.005
Number of self-reported non-cancer illnesses	0.17	0.008	0.36	2.53E-36	-2.78	0.005
Mood swings	-0.03	0.585	0.16	2.80E-05	-2.68	0.007
Medication for pain relief, constipation, heartburn: Laxatives (e.g. Dulcolax, Senokot)	-0.17	0.143	0.17	0.002	-2.67	0.008
Number of depression episodes	-0.32	0.120	0.30	0.008	-2.65	0.008
Age at first live birth	0.07	0.254	-0.12	0.002	2.63	0.008
Body fat percentage	0.01	0.841	0.14	3.28E-08	-2.54	0.011
Leg fat percentage (right)	0.00	0.970	0.13	8.15E-07	-2.53	0.011
Age completed full time education	0.07	0.242	-0.11	0.002	2.52	0.012
Leg fat percentage (left)	0.01	0.904	0.14	2.68E-07	-2.49	0.013
Diagnoses - main ICD10: R35 Polyuria	-0.42	0.109	0.33	0.032	-2.47	0.014
Non-cancer illness code, self-reported: urinary tract infection/kidney infection	-0.39	0.051	0.15	0.088	-2.47	0.014
Sleeplessness / insomnia	-0.06	0.294	0.11	0.001	-2.46	0.014
Arm fat percentage (right)	0.01	0.737	0.14	1.81E-07	-2.44	0.015
Leg fat mass (right)	0.01	0.794	0.14	8.55E-07	-2.43	0.015

Waist circumference	0.04	0.381	0.17	6.94E-11	-2.42	0.015
Trunk fat percentage	0.01	0.771	0.14	1.94E-08	-2.42	0.016
Treatment/medication code: tramadol	-0.16	0.209	0.19	0.009	-2.41	0.016
Leg fat mass (left)	0.02	0.744	0.15	4.90E-07	-2.41	0.016
Arm fat percentage (left)	0.01	0.770	0.14	4.23E-07	-2.40	0.016
Whole body fat mass	0.02	0.653	0.15	9.32E-08	-2.39	0.017
Non-cancer illness code, self-reported: irritable bowel syndrome	-0.20	0.248	0.27	0.004	-2.39	0.017
Body mass index (BMI)	0.01	0.816	0.14	9.77E-06	-2.37	0.018
Overall health rating	0.07	0.166	0.21	8.86E-10	-2.36	0.018
Types of physical activity in last 4 weeks: Other exercises (eg: swimming, cycling, keep fit, bowling)	0.02	0.760	-0.15	1.42E-05	2.35	0.019
Arm fat mass (right)	0.02	0.619	0.15	5.56E-07	-2.35	0.019
Time spent watching television (TV)	-0.05	0.345	0.09	8.00E-04	-2.35	0.019
Frequency of tiredness / lethargy in last 2 weeks	0.07	0.278	0.24	1.25E-14	-2.31	0.021
Diagnoses - main ICD10: F10 Mental and behavioural disorders due to use of alcohol	-0.57	0.049	0.15	0.205	-2.30	0.022
Alcohol usually taken with meals	0.11	0.113	-0.07	0.058	2.28	0.022
Non-cancer illness code, self-reported: depression	-0.09	0.300	0.14	0.003	-2.27	0.023
Smoking/smokers in household	-0.16	0.195	0.15	0.012	-2.27	0.023
Shortness of breath walking on level ground	-0.02	0.841	0.23	1.05E-05	-2.23	0.026
Number of treatments/medications taken	0.18	0.002	0.32	1.43E-22	-2.23	0.026
Seen doctor (GP) for nerves, anxiety, tension or depression	0.01	0.896	0.15	1.24E-07	-2.21	0.027
Neuroticism score	0.00	0.971	0.17	7.49E-05	-2.21	0.027
Trunk fat mass	0.03	0.516	0.14	3.00E-08	-2.20	0.028
Arm fat mass (left)	0.03	0.548	0.15	7.97E-07	-2.19	0.029
Alcohol intake frequency.	-0.02	0.719	0.12	8.46E-06	-2.16	0.031
Mouth/teeth dental problems: None of the above	0.08	0.255	-0.11	0.031	2.15	0.032
Irritability	-0.05	0.533	0.14	0.004	-2.13	0.033
Treatment/medication code: migril tablet	-0.56	0.039	0.09	0.520	-2.13	0.034
Mouth/teeth dental problems: Painful gums	-0.16	0.248	0.17	0.020	-2.10	0.035
Treatment/medication code: fluoxetine	-0.20	0.268	0.22	0.015	-2.10	0.036

Diagnoses - main ICD10: I80 Phlebitis and thrombophlebitis	0.43	0.003	0.07	0.427	2.09	0.037
Treatment/medication code: beclazone 50 inhaler	0.58	0.013	0.06	0.565	2.04	0.041
Average weekly red wine intake	0.06	0.417	-0.11	0.002	2.04	0.042
Treatment/medication code: co-codamol	-0.04	0.759	0.23	1.00E-04	-2.03	0.042
Treatment/medication code: mebeverine	-0.25	0.275	0.31	0.042	-2.03	0.043
Non-cancer illness code, self-reported: heart attack/myocardial infarction	-0.07	0.483	0.15	0.004	-2.02	0.044
Diagnoses - main ICD10: I26 Pulmonary embolism	-0.47	0.044	0.05	0.659	-2.01	0.044
Treatment/medication code: phenobarbital	-0.42	0.063	0.08	0.445	-2.00	0.045
Sensitivity / hurt feelings	0.01	0.834	0.16	2.67E-05	-2.00	0.045
Diagnoses - main ICD10: L43 Lichen planus	-0.37	0.236	0.39	0.070	-2.00	0.045
Diagnoses - main ICD10: K58 Irritable bowel syndrome	-0.13	0.584	0.47	0.008	-1.99	0.047
Treatment/medication code: indoramin	-0.12	0.618	0.45	0.006	-1.98	0.048
Vascular/heart problems diagnosed by doctor: Heart attack	-0.05	0.576	0.16	0.002	-1.98	0.048
Non-cancer illness code, self-reported: prolapsed disc/slipped disc	-0.16	0.312	0.19	0.020	-1.98	0.048
Falls in the last year	0.02	0.824	0.18	1.34E-06	-1.97	0.049
Qualifications: College or University degree	0.01	0.840	-0.10	7.29E-05	1.97	0.049
Types of physical activity in last 4 weeks: None of the above	0.03	0.768	0.22	3.58E-06	-1.97	0.049
Duration of heavy DIY	-0.15	0.189	0.10	0.083	-1.96	0.050
Diagnoses - main ICD10: R10 Abdominal and pelvic pain	-0.03	0.801	0.21	2.00E-04	-1.96	0.050
Non-cancer illness code, self-reported: gout	-0.06	0.554	0.16	0.004	-1.95	0.051
Treatment/medication code: ezetimibe	0.51	0.007	0.10	0.236	1.94	0.052
Diagnoses - main ICD10: K85 Acute pancreatitis	-0.16	0.333	0.20	0.022	-1.94	0.052
Frequency of unenthusiasm / disinterest in last 2 weeks	0.01	0.865	0.18	1.57E-05	-1.92	0.055
Treatment/medication code: chondroitin product	-0.36	0.064	0.05	0.573	-1.92	0.055
Diagnoses - main ICD10: K29 Gastritis and duodenitis	-0.14	0.332	0.17	0.029	-1.90	0.057
Diagnoses - main ICD10: M19 Other arthrosis	-0.29	0.208	0.24	0.126	-1.90	0.057
Diagnoses - main ICD10: N32 Other disorders of bladder	-0.16	0.319	0.19	0.039	-1.89	0.058
Treatment/medication code: nitrazepam	-0.51	0.076	0.08	0.524	-1.88	0.060
Treatment/medication code: paroxetine	-0.28	0.239	0.22	0.064	-1.88	0.060
Ever unenthusiastic/disinterested for a whole week	-0.02	0.869	0.19	1.00E-04	-1.88	0.061

Ever manic/hyper for 2 days	-0.04	0.791	0.29	0.001	-1.87	0.062
Usual walking pace	0.01	0.830	-0.10	7.00E-04	1.86	0.062
Average weekly champagne plus white wine intake	0.14	0.121	-0.05	0.281	1.86	0.062
Diagnoses - main ICD10: M72 Fibroblastic disorders	0.14	0.275	-0.13	0.066	1.86	0.063
Qualifications: None of the above	-0.02	0.717	0.10	0.001	-1.86	0.064
Reason for reducing amount of alcohol drunk: Financial reasons	-0.25	0.203	0.15	0.100	-1.85	0.064
Diagnoses - main ICD10: E04 Other non-toxic goitre	0.16	0.320	-0.20	0.073	1.84	0.066
Exposure to tobacco smoke at home	-0.11	0.374	0.14	0.021	-1.83	0.067
Target heart rate achieved	0.22	0.164	-0.10	0.231	1.79	0.073
Leg pain on walking : action taken	0.17	0.297	-0.16	0.063	1.79	0.073
Illnesses of siblings: Prostate cancer	0.40	0.120	-0.11	0.369	1.79	0.074
Diagnoses - main ICD10: M54 Dorsalgia	-0.12	0.329	0.14	0.078	-1.78	0.076
Treatment/medication code: allopurinol	-0.06	0.565	0.14	0.011	-1.77	0.076
Non-cancer illness code, self-reported: back problem	-0.25	0.119	0.07	0.402	-1.77	0.077
Maternal smoking around birth	-0.06	0.412	0.08	0.023	-1.77	0.078
Guilty feelings	0.02	0.721	0.17	8.12E-05	-1.76	0.078
Diagnoses - main ICD10: J47 Bronchiectasis	0.58	0.028	0.05	0.700	1.76	0.078
Diagnoses - main ICD10: O04 Medical abortion	-1.03	0.063	-0.03	0.810	-1.75	0.081
Frequency of travelling from home to job workplace	-0.19	0.088	0.02	0.657	-1.74	0.082
Weight	0.04	0.413	0.13	2.18E-06	-1.74	0.082
Diagnoses - main ICD10: M75 Shoulder lesions	-0.09	0.543	0.21	0.009	-1.73	0.084
Mineral and other dietary supplements: Selenium	0.32	0.040	0.00	0.989	1.73	0.084
Hip circumference	0.03	0.480	0.13	6.31E-06	-1.73	0.084
Treatment/medication code: ipratropium	0.68	0.050	0.00	0.983	1.72	0.085
Why stopped smoking: Illness or ill health	-0.19	0.308	0.16	0.048	-1.72	0.086
Diagnoses - main ICD10: K60 Fissure and fistula of anal and rectal regions	0.47	0.005	0.15	0.085	1.71	0.087
Treatment/medication code: diclofenac sodium+misoprostol	0.73	0.008	0.21	0.124	1.71	0.088
Mineral and other dietary supplements: Fish oil (including cod liver oil)	0.10	0.225	-0.07	0.204	1.71	0.088
Diagnoses - main ICD10: M17 Gonarthrosis [arthrosis of knee]	-0.11	0.319	0.10	0.078	-1.70	0.089
Suffer from 'nerves'	-0.03	0.731	0.13	0.006	-1.70	0.089

Ever had prostate specific antigen (PSA) test	0.11	0.298	-0.09	0.085	1.69	0.091
Drive faster than motorway speed limit	0.02	0.707	-0.10	0.006	1.69	0.091
Home area population density - urban or rural: England/Wales - Village - less sparse	0.24	0.351	-0.25	0.055	1.69	0.091
Qualifications: A levels/AS levels or equivalent	0.02	0.756	-0.09	0.004	1.68	0.092
Diagnoses - main ICD10: S83 Dislocation, sprain and strain of joints and ligaments of knee	-0.56	0.125	0.11	0.493	-1.68	0.093
Frequency of depressed mood in last 2 weeks	0.05	0.508	0.19	2.76E-06	-1.68	0.094
Noisy workplace	-0.04	0.669	0.13	0.003	-1.67	0.094
Non-cancer illness code, self-reported: hayfever/allergic rhinitis	-0.07	0.435	0.10	0.053	-1.66	0.097
Treatment/medication code: tetralysal 300 capsule	0.38	0.114	-0.11	0.531	1.65	0.098
Diagnoses - main ICD10: N19 Unspecified renal failure	0.41	0.091	-0.05	0.725	1.65	0.099
Diagnoses - main ICD10: S56 Injury of muscle and tendon at forearm level	-0.36	0.316	0.42	0.174	-1.65	0.099
Diagnoses - main ICD10: K21 Gastro-oesophageal reflux disease	0.27	0.104	-0.03	0.684	1.65	0.100
Treatment/medication code: atorvastatin	-0.01	0.933	0.19	7.00E-04	-1.63	0.104
Diagnoses - main ICD10: R05 Cough	-0.25	0.317	0.21	0.124	-1.63	0.104
Forced expiratory volume in 1-second (FEV1), predicted percentage	-0.14	0.031	-0.02	0.625	-1.62	0.104
Age started oral contraceptive pill	0.14	0.131	-0.03	0.538	1.62	0.105
Treatment/medication code: salazopyrin 500mg tablet	0.56	0.026	0.10	0.434	1.62	0.106
Treatment/medication code: sumatriptan	-0.26	0.198	0.11	0.305	-1.62	0.106
Diagnoses - main ICD10: R69 Unknown and unspecified causes of morbidity	-0.15	0.420	0.18	0.028	-1.62	0.106
Treatment/medication code: monomax xl 60mg m/r tablet	-0.58	0.220	0.27	0.236	-1.62	0.106
Number of children fathered	-0.17	0.102	0.02	0.713	-1.61	0.107
Diagnoses - main ICD10: R32 Unspecified urinary incontinence	-0.54	0.187	0.20	0.344	-1.60	0.109
Diagnoses - main ICD10: N97 Female infertility	-0.39	0.170	0.10	0.365	-1.60	0.109
Non-cancer illness code, self-reported: retinal detachment	0.33	0.163	-0.10	0.428	1.60	0.109
Treatment/medication code: senokot 7.5mg tablet	0.49	0.120	-0.05	0.670	1.60	0.109
Diagnoses - main ICD10: M48 Other spondylopathies	-0.33	0.163	0.08	0.414	-1.60	0.109
Frequency of tenseness / restlessness in last 2 weeks	-0.02	0.781	0.11	0.002	-1.60	0.109
Surgery/amputation of toe or leg: Yes, toes	0.54	0.124	-0.06	0.647	1.60	0.109
Tobacco smoking: Occasionally	0.21	0.399	-0.25	0.083	1.60	0.110

Ever used hormone-replacement therapy (HRT)	-0.07	0.474	0.10	0.048	-1.60	0.110
Non-cancer illness code, self-reported: anxiety/panic attacks	-0.18	0.292	0.12	0.122	-1.60	0.110
Duration of walks	-0.13	0.093	0.00	0.898	-1.60	0.110
Age at last live birth	0.06	0.401	-0.09	0.123	1.59	0.111
Qualifications: Other professional qualifications eg: nursing, teaching	0.07	0.346	-0.06	0.084	1.59	0.111
Treatment/medication code: insulin product	0.15	0.337	0.42	6.26E-07	-1.59	0.112
Pain type(s) experienced in last month: Facial pain	-0.02	0.902	0.25	0.005	-1.59	0.113
Treatment/medication code: tibolone	0.50	0.268	-0.33	0.213	1.59	0.113
Qualifications: CSEs or equivalent	0.00	0.988	0.17	1.00E-04	-1.58	0.114
Friendships satisfaction	0.21	0.016	0.06	0.214	1.58	0.115
Non-cancer illness code, self-reported: nasal polyps	-0.24	0.231	0.10	0.219	-1.58	0.115
Handedness (chirality/laterality): Left-handed	0.02	0.902	-0.20	6.00E-04	1.57	0.116
Ever highly irritable/argumentative for 2 days	-0.02	0.807	0.15	0.005	-1.57	0.117
Treatment/medication code: travatan 40micrograms/ml eye drops	-0.67	0.186	0.18	0.359	-1.57	0.117
Medication for cholesterol, blood pressure or diabetes: Cholesterol lowering medication	0.03	0.690	0.17	3.65E-05	-1.56	0.119
Diagnoses - main ICD10: N05 Unspecified nephritic syndrome	-0.37	0.284	0.22	0.156	-1.56	0.119
Diagnoses - main ICD10: H40 Glaucoma	0.55	0.148	-0.08	0.566	1.56	0.120
Treatment/medication code: movicol oral powder	-0.31	0.322	0.25	0.165	-1.55	0.121
Sleep duration	0.04	0.466	-0.07	0.094	1.55	0.122
Other serious medical condition/disability diagnosed by doctor	0.32	8.65E-05	0.46	7.43E-38	-1.54	0.122
Treatment/medication code: betahistine	-0.44	0.196	0.15	0.392	-1.54	0.123
Illnesses of father: Severe depression	-0.21	0.158	0.04	0.536	-1.54	0.123
Diagnoses - main ICD10: K80 Cholelithiasis	-0.08	0.578	0.17	0.021	-1.54	0.123
Diagnoses - main ICD10: D48 Neoplasm of uncertain or unknown behaviour of other and unspecified sites	0.18	0.439	-0.23	0.079	1.54	0.125
Cancer code, self-reported: prostate cancer	0.25	0.116	-0.02	0.793	1.54	0.125
Non-cancer illness code, self-reported: abdominal hernia	0.61	0.274	-0.36	0.223	1.53	0.125
Treatment/medication code: tiotropium	0.47	0.040	0.05	0.733	1.52	0.128
Illness, injury, bereavement, stress in last 2 years: Serious illness, injury or assault to yourself	0.11	0.345	0.30	1.99E-08	-1.52	0.129
Diagnoses - main ICD10: I20 Angina pectoris	0.08	0.571	0.31	1.33E-05	-1.52	0.129

Miserableness	0.03	0.694	0.15	6.00E-04	-1.51	0.131
Townsend deprivation index at recruitment	-0.03	0.728	0.10	0.007	-1.51	0.132
Illness, injury, bereavement, stress in last 2 years: Financial difficulties	0.06	0.447	0.19	8.82E-07	-1.50	0.134
Treatment/medication code: half-inderal la 80mg m/r capsule	0.58	0.195	-0.14	0.430	1.50	0.134
Non-cancer illness code, self-reported: diverticular disease/diverticulitis	-0.14	0.218	0.05	0.393	-1.49	0.137
Diagnoses - main ICD10: K22 Other diseases of oesophagus	0.28	0.149	-0.07	0.595	1.49	0.137
Non-cancer illness code, self-reported: breast fibroadenoma	0.28	0.284	-0.16	0.246	1.48	0.139
Relative age voice broke	0.02	0.840	-0.11	0.008	1.48	0.139
Treatment/medication code: venlafaxine	-0.08	0.654	0.22	0.027	-1.48	0.139
Duration of light DIY	-0.05	0.598	0.10	0.034	-1.48	0.140
Treatment/medication code: flecainide	0.25	0.267	-0.11	0.241	1.47	0.142
Types of transport used (excluding work): Cycle	0.05	0.577	-0.09	0.037	1.47	0.142
Non-cancer illness code, self-reported: other renal/kidney problem	-0.40	0.341	0.37	0.242	-1.46	0.144
Non-cancer illness code, self-reported: tennis elbow / lateral epicondylitis	-0.36	0.211	0.12	0.450	-1.46	0.145
Financial situation satisfaction	0.00	0.987	0.15	0.002	-1.45	0.146
Treatment/medication code: cetirizine	-0.11	0.505	0.15	0.043	-1.45	0.148
Blood clot, DVT, bronchitis, emphysema, asthma, rhinitis, eczema, allergy diagnosed by doctor: Blood clot in the lung	-0.06	0.691	0.18	0.010	-1.45	0.148
Number of operations, self-reported	0.05	0.483	0.18	3.45E-05	-1.44	0.149
Seen a psychiatrist for nerves, anxiety, tension or depression	0.02	0.763	0.15	5.25E-05	-1.44	0.149
Underlying (primary) cause of death: ICD10: C64 Malignant neoplasm of kidney, except renal pelvis	-0.40	0.190	0.08	0.550	-1.44	0.150
Diagnoses - main ICD10: S69 Other and unspecified injuries of wrist and hand	-0.36	0.226	0.10	0.430	-1.43	0.153
Diagnoses - main ICD10: Z48 Other surgical follow-up care	0.53	0.070	0.08	0.530	1.42	0.155
Treatment/medication code: liothyronine	-0.27	0.508	0.62	0.194	-1.42	0.156
Treatment/medication code: ferrous salt product	0.57	0.180	-0.07	0.647	1.42	0.156
Non-cancer illness code, self-reported: acute infective polyneuritis/guillain-barre syndrome	0.20	0.430	-0.19	0.092	1.41	0.157
Had major operations	0.01	0.919	0.19	2.00E-04	-1.41	0.158
Medication for cholesterol, blood pressure or diabetes: Insulin	0.12	0.540	0.46	3.00E-04	-1.41	0.158
Treatment/medication code: acrivastine	-0.44	0.258	0.16	0.384	-1.39	0.164

Home area population density - urban or rural: England/Wales - Hamlet and Isolated Dwelling - less sparse	0.16	0.343	-0.10	0.225	1.39	0.164
Non-cancer illness code, self-reported: blistering/desquamating skin disorder	0.15	0.487	-0.18	0.092	1.39	0.164
Fractured bone site(s): Other bones	0.32	0.029	0.10	0.110	1.39	0.165
Treatment/medication code: prednisolone product	0.57	0.098	0.06	0.656	1.38	0.166
Diagnoses - main ICD10: M46 Other inflammatory spondylopathies	0.28	0.295	-0.18	0.361	1.38	0.166
Non-cancer illness code, self-reported: pancreatitis	-0.47	0.328	0.29	0.284	-1.38	0.169
Underlying (primary) cause of death: ICD10: C15.9 Oesophagus, unspecified	0.36	0.356	-0.24	0.215	1.38	0.169
Transport type for commuting to job workplace: Public transport	0.16	0.121	0.00	0.987	1.37	0.169
Diagnoses - main ICD10: O03 Spontaneous abortion	0.30	0.282	-0.14	0.379	1.37	0.170
Maximum heart rate during fitness test	0.10	0.408	-0.11	0.247	1.37	0.171
Diagnoses - main ICD10: R26 Abnormalities of gait and mobility	-0.28	0.342	0.20	0.298	-1.36	0.172
Diagnoses - main ICD10: Z09 Follow-up examination after treatment for conditions other than malignant neoplasms	0.30	0.181	-0.03	0.738	1.36	0.173
Started insulin within one year diagnosis of diabetes	0.10	0.617	0.40	4.00E-04	-1.36	0.174
Treatment/medication code: iron product	-0.13	0.691	0.41	0.079	-1.36	0.175
Peak expiratory flow (PEF)	-0.07	0.288	0.03	0.394	-1.36	0.175
Treatment/medication code: anastrozole	-0.46	0.286	0.17	0.339	-1.35	0.176
Non-cancer illness code, self-reported: gastric/stomach ulcers	0.65	0.132	0.02	0.903	1.35	0.176
Diagnoses - main ICD10: K57 Diverticular disease of intestine	-0.15	0.155	0.02	0.798	-1.35	0.177
Underlying (primary) cause of death: ICD10: I71.0 Dissection of aorta [any part]	0.86	0.187	-0.06	0.773	1.35	0.178
Diagnoses - main ICD10: T18 Foreign body in alimentary tract	-0.12	0.622	0.25	0.051	-1.35	0.178
Forced expiratory volume in 1-second (FEV1)	-0.09	0.099	-0.01	0.831	-1.34	0.179
Leg pain when walking normally	-0.13	0.630	0.29	0.071	-1.34	0.180
Diagnoses - main ICD10: M05 Seropositive rheumatoid arthritis	0.54	0.073	0.08	0.593	1.34	0.180
Diagnoses - main ICD10: N64 Other disorders of breast	-0.20	0.534	0.30	0.122	-1.34	0.180
Types of transport used (excluding work): Car/motor vehicle	-0.07	0.441	0.06	0.115	-1.34	0.181
Non-cancer illness code, self-reported: miscarriage	-0.43	0.242	0.12	0.518	-1.34	0.181
Treatment/medication code: nortriptyline	0.52	0.243	-0.18	0.515	1.34	0.182
Treatment/medication code: minocycline	0.39	0.213	-0.08	0.619	1.33	0.182
Nervous feelings	-0.03	0.673	0.09	0.080	-1.32	0.186

Mother still alive	0.13	0.519	-0.17	0.104	1.32	0.186
Non-cancer illness code, self-reported: spine arthritis/spondylitis	-0.12	0.533	0.16	0.067	-1.32	0.187
Non-cancer illness code, self-reported: bell's palsy/facial nerve palsy	0.13	0.603	-0.26	0.081	1.32	0.188
Types of physical activity in last 4 weeks: Light DIY (eg: pruning, watering the lawn)	0.02	0.778	-0.08	0.032	1.32	0.188
Alcohol drinker status: Previous	-0.09	0.406	0.07	0.205	-1.32	0.188
Diagnoses - main ICD10: O60 Preterm delivery	0.18	0.643	-0.46	0.120	1.32	0.188
Mouth/teeth dental problems: Dentures	-0.03	0.664	0.08	0.088	-1.32	0.189
Diagnoses - main ICD10: Z71 Persons encountering health services for other counselling and medical advice, not elsewhere classified	0.53	0.296	-0.19	0.383	1.31	0.191
Treatment/medication code: indivina 1mg/2.5mg tablet	-0.34	0.213	0.05	0.679	-1.31	0.191
Diagnoses - main ICD10: C22 Malignant neoplasm of liver and intrahepatic bile ducts	-0.09	0.714	0.27	0.045	-1.30	0.192
Non-cancer illness code, self-reported: psoriasis	-0.03	0.854	0.21	0.022	-1.30	0.193
Ever had bowel cancer screening	-0.04	0.666	0.10	0.042	-1.30	0.194
Vitamin and mineral supplements: Vitamin B	-0.10	0.481	0.11	0.142	-1.30	0.194
Illness, injury, bereavement, stress in last 2 years: Death of a close relative	-0.19	0.247	0.04	0.564	-1.29	0.196
Non-cancer illness code, self-reported: emphysema	-0.48	0.188	0.02	0.879	-1.29	0.196
Ever depressed for a whole week	0.00	0.988	0.14	0.006	-1.29	0.198
Non-cancer illness code, self-reported: enlarged prostate	0.15	0.376	-0.09	0.243	1.29	0.198
Treatment/medication code: merbentyl 10mg tablet	0.51	0.365	-0.40	0.351	1.29	0.198
Tense / 'highly strung'	0.01	0.934	0.12	0.006	-1.29	0.198
Treatment/medication code: valsartan	-0.22	0.194	0.03	0.757	-1.29	0.199
Diagnoses - main ICD10: H25 Senile cataract	0.06	0.779	-0.25	0.057	1.29	0.199
Able to walk or cycle unaided for 10 minutes	0.04	0.892	-0.35	0.022	1.28	0.200
Diagnoses - main ICD10: H80 Otosclerosis	-0.33	0.104	-0.03	0.768	-1.28	0.200
Illnesses of mother: Chronic bronchitis/emphysema	0.00	0.966	0.15	0.002	-1.27	0.202
Treatment/medication code: betnovate cream	-1.05	0.271	0.30	0.519	-1.27	0.203
Diagnoses - main ICD10: L50 Urticaria	-0.33	0.231	0.06	0.660	-1.27	0.204
Treatment/medication code: amias 2mg tablet	0.53	0.273	-0.12	0.496	1.27	0.205
Vitamin and mineral supplements: Folic acid or Folate (Vit B9)	0.08	0.728	0.44	0.005	-1.27	0.205
Non-cancer illness code, self-reported: kidney stone/ureter stone/bladder stone	-0.06	0.614	0.12	0.110	-1.26	0.206

Diagnoses - main ICD10: K90 Intestinal malabsorption	-0.01	0.976	0.25	0.025	-1.26	0.207
Exposure to tobacco smoke outside home	-0.02	0.801	0.10	0.021	-1.26	0.207
Sodium in urine	-0.06	0.353	0.03	0.361	-1.26	0.208
Cancer code, self-reported: tongue cancer	-0.11	0.611	0.21	0.088	-1.26	0.209
Diagnoses - main ICD10: N28 Other disorders of kidney and ureter, not elsewhere classified	-0.41	0.204	0.12	0.663	-1.26	0.209
Diagnoses - main ICD10: L85 Other epidermal thickening	0.65	0.164	-0.02	0.926	1.26	0.209
Non-cancer illness code, self-reported: cerebral aneurysm	-0.38	0.139	-0.03	0.814	-1.26	0.209
Fractured bone site(s): Arm	-0.08	0.683	0.20	0.060	-1.25	0.210
Cancer code, self-reported: rectal cancer	0.34	0.173	-0.01	0.925	1.25	0.210
Doctor diagnosed bronchiectasis	0.60	0.080	0.13	0.396	1.25	0.211
Diagnoses - main ICD10: D24 Benign neoplasm of breast	0.56	0.265	-0.12	0.562	1.25	0.212
Treatment/medication code: indapamide	0.57	0.109	0.09	0.534	1.25	0.213
Why reduced smoking: Illness or ill health	0.45	0.025	0.15	0.245	1.24	0.213
Non-cancer illness code, self-reported: pulmonary embolism +/- dvt	-0.05	0.768	0.17	0.023	-1.24	0.215
Cancer code, self-reported: malignant melanoma	-0.35	0.053	-0.09	0.345	-1.23	0.217
Non-accidental death in close genetic family	0.31	0.057	0.07	0.447	1.23	0.217
Number of days/week walked 10+ minutes	0.02	0.747	-0.07	0.046	1.23	0.217
Treatment/medication code: strontium product	-0.35	0.228	0.04	0.749	-1.23	0.217
Treatment/medication code: beclometasone	-0.06	0.745	0.19	0.057	-1.23	0.218
Health satisfaction	0.10	0.186	0.21	2.46E-05	-1.23	0.219
Non-cancer illness code, self-reported: post-natal depression	-0.53	0.358	0.28	0.384	-1.23	0.220
Treatment/medication code: clonidine	-0.04	0.848	0.26	0.029	-1.22	0.222
Diagnoses - main ICD10: Z42 Follow-up care involving plastic surgery	-0.33	0.220	0.04	0.777	-1.22	0.222
Diagnoses - main ICD10: S52 Fracture of forearm	-0.15	0.369	0.09	0.389	-1.22	0.223
Duration of moderate activity	-0.10	0.277	0.02	0.574	-1.22	0.223
Mineral and other dietary supplements: Zinc	-0.12	0.302	0.04	0.517	-1.22	0.223
Types of transport used (excluding work): None of the above	-0.11	0.507	0.11	0.151	-1.21	0.224
Number of unenthusiastic/disinterested episodes	-0.09	0.700	0.22	0.053	-1.21	0.225
Forced expiratory volume in 1-second (FEV1), Best measure	-0.08	0.135	-0.01	0.815	-1.21	0.226

Pulse wave reflection index	-0.11	0.268	0.03	0.625	-1.21	0.227
Why stopped smoking: Health precaution	0.08	0.462	-0.07	0.207	1.21	0.227
Diagnoses - main ICD10: N39 Other disorders of urinary system	0.01	0.967	0.25	0.008	-1.20	0.229
Diagnoses - main ICD10: D70 Agranulocytosis	-0.83	0.193	-0.03	0.885	-1.20	0.230
Non-cancer illness code, self-reported: helicobacter pylori	-0.23	0.385	0.12	0.322	-1.20	0.231
Ever stopped smoking for 6+ months	-0.06	0.743	0.17	0.029	-1.20	0.232
Eye problems/disorders: Cataract	0.16	0.213	-0.01	0.851	1.19	0.232
Diagnoses - main ICD10: R06 Abnormalities of breathing	-0.30	0.246	0.04	0.734	-1.19	0.235
Medication for pain relief, constipation, heartburn: None of the above	-0.08	0.173	-0.16	1.41E-07	1.19	0.235
Reason for glasses/contact lenses: For long-sightedness, i.e. for distance and near, but particularly for near tasks like reading (called 'hypermetropia')	-0.02	0.843	0.14	0.027	-1.18	0.236
Diagnoses - main ICD10: K30 Dyspepsia	0.29	0.165	0.02	0.844	1.18	0.236
Treatment/medication code: felodipine	0.19	0.352	-0.09	0.459	1.18	0.237
Treatment/medication code: tamoxifen	-0.36	0.264	0.06	0.689	-1.18	0.237
Non-cancer illness code, self-reported: ear/vestibular disorder	-0.01	0.963	0.38	0.043	-1.17	0.241
Treatment/medication code: capasal shampoo	-0.23	0.330	0.10	0.515	-1.17	0.241
Pain type(s) experienced in last month: Stomach or abdominal pain	0.08	0.426	0.20	5.36E-06	-1.17	0.243
Treatment/medication code: isosorbide mononitrate	-0.12	0.500	0.12	0.225	-1.17	0.243
Diagnoses - main ICD10: G62 Other polyneuropathies	0.23	0.395	-0.13	0.382	1.17	0.244
Treatment/medication code: amiloride	0.60	0.065	0.18	0.204	1.16	0.244
Treatment/medication code: trimethoprim	-0.17	0.656	0.43	0.221	-1.16	0.245
Diagnoses - main ICD10: R11 Nausea and vomiting	0.56	0.128	0.09	0.604	1.16	0.245
Diagnoses - main ICD10: H65 Nonsuppurative otitis media	0.07	0.799	0.46	0.037	-1.16	0.245
Manic/hyper symptoms: I was more talkative than usual	-0.46	0.177	-0.03	0.820	-1.16	0.245
Treatment/medication code: olmesartan	0.29	0.355	-0.11	0.455	1.16	0.247
Diagnoses - main ICD10: C50 Malignant neoplasm of breast	-0.19	0.138	-0.02	0.711	-1.16	0.247
Diagnoses - main ICD10: K35 Acute appendicitis	-0.13	0.626	0.24	0.159	-1.16	0.247
Treatment/medication code: gaviscon liquid	0.55	0.045	0.21	0.070	1.16	0.248
Diagnoses - main ICD10: D36 Benign neoplasm of other and unspecified sites	-0.59	0.252	0.04	0.828	-1.16	0.248
Diagnoses - main ICD10: H59 Postprocedural disorders of eye and adnexa, not elsewhere classified	0.27	0.453	-0.21	0.325	1.15	0.250

Diagnoses - main ICD10: G70 Myasthenia gravis and other myoneural disorders	0.86	0.105	0.20	0.346	1.15	0.251
Cancer code, self-reported: thyroid cancer	0.21	0.363	-0.10	0.490	1.14	0.255
Treatment/medication code: trandolapril	-0.25	0.321	0.07	0.586	-1.13	0.257
Non-cancer illness code, self-reported: epilepsy	-0.21	0.410	0.11	0.382	-1.13	0.258
Diagnoses - main ICD10: I31 Other diseases of pericardium	-0.13	0.654	0.27	0.177	-1.13	0.258
Underlying (primary) cause of death: ICD10: C91.0 Acute lymphoblastic leukaemia	0.33	0.495	-0.34	0.323	1.13	0.258
Non-cancer illness code, self-reported: oesophageal disorder	0.30	0.235	-0.01	0.925	1.13	0.259
Diagnoses - main ICD10: C78 Secondary malignant neoplasm of respiratory and digestive organs	0.42	0.259	-0.03	0.831	1.13	0.259
Treatment/medication code: terbinafine	0.38	0.134	0.07	0.507	1.13	0.259
Underlying (primary) cause of death: ICD10: C67.9 Bladder, unspecified	-0.29	0.346	0.09	0.508	-1.13	0.259
Treatment/medication code: etodolac	0.46	0.160	0.05	0.737	1.12	0.261
Number of trend entries	0.17	0.348	-0.06	0.535	1.12	0.263
Diagnoses - main ICD10: Z30 Contraceptive management	-0.65	0.127	-0.14	0.387	-1.11	0.265
Job involves mainly walking or standing	-0.04	0.572	0.05	0.142	-1.11	0.267
Treatment/medication code: rosiglitazone	0.20	0.331	-0.06	0.598	1.11	0.269
Treatment/medication code: kapake tablet	0.11	0.600	-0.17	0.202	1.10	0.271
Diagnoses - main ICD10: I47 Paroxysmal tachycardia	0.32	0.189	0.03	0.817	1.10	0.272
Back pain for 3+ months	0.00	0.982	0.16	0.018	-1.10	0.273
Diagnoses - main ICD10: K42 Umbilical hernia	-0.03	0.866	0.21	0.073	-1.09	0.274
Treatment/medication code: doxazosin	0.30	0.025	0.12	0.280	1.09	0.274
Diagnoses - main ICD10: H26 Other cataract	0.23	0.076	0.07	0.261	1.09	0.275
Frequency of walking for pleasure in last 4 weeks	-0.06	0.477	0.05	0.296	-1.09	0.275
Underlying (primary) cause of death: ICD10: J45.9 Asthma, unspecified	-0.31	0.196	-0.02	0.902	-1.09	0.277
Number of spontaneous miscarriages	-0.25	0.251	0.01	0.928	-1.09	0.277
Diagnoses - main ICD10: J33 Nasal polyp	-0.17	0.455	0.10	0.350	-1.09	0.278
Diagnoses - main ICD10: M20 Acquired deformities of fingers and toes	-0.21	0.068	-0.07	0.276	-1.08	0.278
Non-cancer illness code, self-reported: head injury	-0.12	0.549	0.12	0.206	-1.08	0.278
Diagnoses - main ICD10: K08 Other disorders of teeth and supporting structures	0.24	0.259	-0.02	0.880	1.08	0.279
Illnesses of father: Chronic bronchitis/emphysema	0.02	0.872	0.15	0.011	-1.08	0.279

Treatment/medication code: daktacort cream	-0.39	0.186	-0.04	0.794	-1.08	0.280
Treatment/medication code: canesten 1% cream	-0.18	0.617	0.27	0.209	-1.08	0.282
Light smokers, at least 100 smokes in lifetime	0.12	0.166	0.02	0.624	1.07	0.283
Diagnoses - main ICD10: N34 Urethritis and urethral syndrome	-0.66	0.182	-0.10	0.577	-1.07	0.283
Blood clot, DVT, bronchitis, emphysema, asthma, rhinitis, eczema, allergy diagnosed by doctor: None of the above	0.01	0.842	-0.07	0.068	1.07	0.283
Mineral and other dietary supplements: None of the above	-0.06	0.425	0.04	0.453	-1.07	0.283
Non-cancer illness code, self-reported: spinal cord disorder	0.17	0.632	-0.30	0.233	1.07	0.285
Diagnoses - main ICD10: G51 Facial nerve disorders	0.74	0.410	-0.30	0.421	1.07	0.285
Job involves heavy manual or physical work	-0.03	0.642	0.05	0.119	-1.07	0.285
Treatment/medication code: alendronate sodium	0.02	0.871	0.18	0.029	-1.07	0.286
Pain type(s) experienced in last month: Hip pain	0.11	0.183	0.21	4.94E-05	-1.07	0.286
Blood clot, DVT, bronchitis, emphysema, asthma, rhinitis, eczema, allergy diagnosed by doctor: Blood clot in the leg (DVT)	-0.04	0.738	0.12	0.147	-1.06	0.287
Diagnoses - main ICD10: S01 Open wound of head	0.79	0.251	0.04	0.828	1.06	0.287
Fractured/broken bones in last 5 years	0.27	0.011	0.14	0.002	1.06	0.290
Treatment/medication code: nifedipine	0.49	0.102	0.13	0.431	1.06	0.290
Home area population density - urban or rural: Scotland - Large Urban Area	0.06	0.657	-0.11	0.176	1.06	0.291
Diagnoses - main ICD10: Z03 Medical observation and evaluation for suspected diseases and conditions	0.28	0.203	0.01	0.938	1.06	0.291
Diagnoses - main ICD10: K76 Other diseases of liver	-0.22	0.281	0.02	0.859	-1.05	0.292
Non-cancer illness code, self-reported: pulmonary fibrosis	0.54	0.223	0.05	0.773	1.05	0.293
Treatment/medication code: multivitamin+mineral preparations	-0.05	0.800	0.17	0.071	-1.05	0.294
Diagnoses - main ICD10: C02 Malignant neoplasm of other and unspecified parts of tongue	-0.07	0.703	0.15	0.132	-1.05	0.294
Diagnoses - main ICD10: K00 Disorders of tooth development and eruption	0.57	0.208	0.07	0.653	1.05	0.295
Diagnoses - main ICD10: D05 Carcinoma in situ of breast	-0.28	0.513	0.25	0.348	-1.05	0.295
Non-cancer illness code, self-reported: spinal injury	0.20	0.402	-0.13	0.527	1.05	0.295
Reason for reducing amount of alcohol drunk: Illness or ill health	0.07	0.737	0.30	0.002	-1.04	0.299
Treatment/medication code: beconase 50micrograms nasal spray	-0.10	0.690	0.21	0.190	-1.04	0.299
Treatment/medication code: gtn 400micrograms spray	-0.16	0.405	0.06	0.523	-1.03	0.302

Non-cancer illness code, self-reported: carpal tunnel syndrome	0.37	0.104	0.09	0.502	1.03	0.303
Forced vital capacity (FVC)	-0.08	0.107	-0.02	0.382	-1.03	0.304
Diagnoses - main ICD10: D23 Other benign neoplasms of skin	-0.28	0.286	0.01	0.906	-1.03	0.304
Diagnoses - main ICD10: K41 Femoral hernia	0.64	0.226	0.03	0.909	1.03	0.305
Treatment/medication code: telmisartan	-0.11	0.635	0.16	0.220	-1.03	0.305
Number of unsuccessful stop-smoking attempts	-0.09	0.450	0.05	0.473	-1.02	0.306
Non-cancer illness code, self-reported: gastrointestinal bleeding	-0.28	0.561	0.33	0.347	-1.02	0.306
Treatment/medication code: doxycycline	0.75	0.235	0.08	0.653	1.02	0.309
Current employment status: Unable to work because of sickness or disability	0.07	0.367	0.17	6.00E-04	-1.02	0.309
Fractured bone site(s): Hip	0.45	0.083	0.14	0.369	1.02	0.310
Which eye(s) affected by myopia (short sight): Right eye	0.13	0.528	-0.10	0.299	1.01	0.310
Cancer code, self-reported: brain cancer / primary malignant brain tumour	-0.92	0.390	0.22	0.524	-1.01	0.311
Snoring	0.00	0.964	-0.07	0.041	1.01	0.312
Non-cancer illness code, self-reported: cataract	-0.03	0.893	0.19	0.047	-1.01	0.312
Treatment/medication code: flucloxacillin	0.38	0.456	-0.19	0.438	1.01	0.313
Treatment/medication code: penicillin	0.44	0.515	-0.36	0.387	1.01	0.314
Treatment/medication code: deep relief ibuprofen gel	-0.15	0.437	0.07	0.489	-1.01	0.314
Diagnoses - main ICD10: D22 Melanocytic naevi	-0.93	0.375	0.17	0.580	-1.01	0.315
Arm fat-free mass (left)	0.05	0.321	0.11	2.00E-04	-1.00	0.318
Diagnoses - main ICD10: R40 Somnolence, stupor and coma	0.21	0.514	-0.16	0.390	1.00	0.318
Diagnoses - main ICD10: R30 Pain associated with micturition	-0.24	0.378	0.06	0.638	-1.00	0.319
Ever taken oral contraceptive pill	-0.10	0.424	0.04	0.523	-1.00	0.319
Treatment/medication code: liquifilm tears 1.4% eye drops	-0.50	0.439	0.19	0.462	-1.00	0.319
Treatment/medication code: calcium carbonate+cholecalciferol 1.25g/5micrograms tablet	0.01	0.960	0.26	0.036	-1.00	0.319
Treatment/medication code: citalopram	0.04	0.808	0.22	0.015	-1.00	0.320
Treatment/medication code: loperamide	-0.16	0.597	0.19	0.277	-1.00	0.320
Non-cancer illness code, self-reported: hepatitis a	-0.60	0.484	0.32	0.372	-0.99	0.320
Non-cancer illness code, self-reported: sciatica	0.49	0.119	0.15	0.295	0.99	0.320
Treatment/medication code: clobetasol	0.53	0.121	0.16	0.270	0.99	0.320

Arm predicted mass (left)	0.05	0.321	0.11	2.00E-04	-0.99	0.321
Blood clot, DVT, bronchitis, emphysema, asthma, rhinitis, eczema, allergy diagnosed by doctor: Emphysema/chronic bronchitis	0.32	0.003	0.18	0.051	0.99	0.321
Non-cancer illness code, self-reported: fracture skull / head	-0.36	0.198	-0.05	0.731	-0.99	0.323
Illnesses of father: Stroke	-0.07	0.700	0.14	0.158	-0.99	0.323
Diagnoses - main ICD10: I10 Essential (primary) hypertension	0.45	0.093	0.16	0.224	0.99	0.324
Illnesses of father: Alzheimer's disease/dementia	0.62	0.449	-0.27	0.479	0.99	0.324
Diagnoses - main ICD10: A04 Other bacterial intestinal infections	0.39	0.099	0.13	0.277	0.98	0.325
Treatment/medication code: e45 cream	-0.56	0.341	0.04	0.804	-0.98	0.325
Diagnoses - main ICD10: D50 Iron deficiency anaemia	0.48	0.037	0.23	0.032	0.98	0.326
Ever had cervical smear test	0.30	0.192	0.05	0.610	0.98	0.327
Underlying (primary) cause of death: ICD10: C45.9 Mesothelioma, unspecified	0.29	0.286	0.00	0.976	0.98	0.327
Why reduced smoking: Doctor's advice	0.30	0.411	-0.14	0.595	0.98	0.329
Diagnoses - main ICD10: N70 Salpingitis and oophoritis	-0.13	0.708	0.32	0.283	-0.97	0.331
Underlying (primary) cause of death: ICD10: A81.0 Creutzfeldt-Jakob disease	0.25	0.287	-0.02	0.880	0.97	0.331
Leg pain in calf/calves	-0.19	0.479	0.10	0.460	-0.97	0.332
Diagnoses - main ICD10: M06 Other rheumatoid arthritis	0.22	0.401	0.54	0.008	-0.97	0.332
Diagnoses - main ICD10: D18 Haemangioma and lymphangioma, any site	0.26	0.480	-0.14	0.456	0.97	0.333
Diagnoses - main ICD10: L82 Seborrhoeic keratosis	0.13	0.777	-0.57	0.311	0.97	0.333
Diagnoses - main ICD10: O47 False labour	0.36	0.346	-0.04	0.807	0.97	0.334
Why reduced smoking: None of the above	-0.27	0.493	0.18	0.469	-0.97	0.334
Pain type(s) experienced in last month: Knee pain	0.05	0.465	0.12	0.001	-0.97	0.334
Treatment/medication code: logynon tablet	-0.32	0.243	-0.03	0.790	-0.96	0.335
Treatment/medication code: asacol mr 400mg e/c tablet	-0.03	0.905	0.26	0.109	-0.96	0.336
Reported occurrences of cancer	-0.42	0.152	-0.12	0.339	-0.96	0.337
Non-cancer illness code, self-reported: benign breast lump	-0.22	0.346	0.03	0.803	-0.96	0.338
Treatment/medication code: meloxicam	0.45	0.338	-0.06	0.806	0.96	0.338
Non-cancer illness code, self-reported: migraine	-0.07	0.470	0.03	0.503	-0.96	0.339
Which eye(s) affected by astigmatism: Right eye	-0.16	0.409	0.05	0.632	-0.96	0.339
Cancer code, self-reported: testicular cancer	-0.25	0.235	-0.02	0.835	-0.95	0.340

Non-cancer illness code, self-reported: deep venous thrombosis (dvt)	-0.05	0.699	0.09	0.251	-0.95	0.340
Non-cancer illness code, self-reported: bronchiectasis	0.53	0.123	0.17	0.273	0.95	0.340
Non-cancer illness code, self-reported: allergy or anaphylactic reaction to food	-0.10	0.714	0.21	0.220	-0.95	0.341
Treatment/medication code: enalapril maleate+hydrochlorothiazide 20mg/12.5mg tablet	-0.23	0.366	0.05	0.738	-0.95	0.341
Cancer code, self-reported: basal cell carcinoma	-0.07	0.557	-0.19	7.00E-04	0.95	0.341
Age when periods started (menarche)	0.01	0.927	-0.06	0.051	0.95	0.341
Diagnoses - main ICD10: M10 Gout	0.21	0.373	-0.06	0.713	0.95	0.342
Impedance of arm (left)	-0.02	0.726	-0.07	0.026	0.95	0.342
Impedance of arm (right)	-0.02	0.732	-0.07	0.031	0.95	0.342
Treatment/medication code: spironolactone	0.33	0.273	-0.02	0.912	0.95	0.342
Diagnoses - main ICD10: C62 Malignant neoplasm of testis	-0.32	0.461	0.14	0.523	-0.95	0.342
Why stopped smoking: Financial reasons	-0.08	0.663	0.12	0.237	-0.95	0.343
Reason for glasses/contact lenses: For a 'squint' or 'turn' in an eye since childhood (called 'strabismus')	-0.17	0.373	0.03	0.737	-0.95	0.343
Blood clot, DVT, bronchitis, emphysema, asthma, rhinitis, eczema, allergy diagnosed by doctor: Hayfever, allergic rhinitis or eczema	-0.04	0.596	0.04	0.318	-0.95	0.344
Leg pain when walking uphill or hurrying	0.07	0.734	0.30	0.011	-0.94	0.345
Underlying (primary) cause of death: ICD10: E85.4 Organ-limited amyloidosis	-0.35	0.380	0.06	0.732	-0.94	0.346
Cancer code, self-reported: chronic myeloid	-0.23	0.553	0.19	0.396	-0.94	0.346
Treatment/medication code: rizatriptan	0.16	0.556	-0.13	0.375	0.94	0.348
Non-cancer illness code, self-reported: osteoporosis	0.01	0.948	0.13	0.076	-0.94	0.348
Vascular/heart problems diagnosed by doctor: Stroke	0.00	0.997	0.21	0.050	-0.94	0.349
Diagnoses - main ICD10: T17 Foreign body in respiratory tract	0.54	0.190	0.13	0.405	0.94	0.350
Underlying (primary) cause of death: ICD10: J84.1 Other interstitial pulmonary diseases with fibrosis	0.29	0.346	-0.02	0.869	0.93	0.350
Manic/hyper symptoms: None of the above	0.23	0.283	0.01	0.946	0.93	0.350
Diagnoses - main ICD10: G47 Sleep disorders	-0.06	0.704	0.11	0.240	-0.93	0.350
Treatment/medication code: cipralex 5mg tablet	-0.05	0.853	0.24	0.117	-0.93	0.351
Fractured bone site(s): Spine	-0.31	0.286	-0.01	0.920	-0.93	0.351
Loneliness, isolation	0.11	0.128	0.19	1.82E-06	-0.93	0.352

Non-cancer illness code, self-reported: cholelithiasis/gall stones	0.03	0.819	0.18	0.009	-0.93	0.353
Diagnoses - main ICD10: T43 Poisoning by psychotropic drugs, not elsewhere classified	-0.13	0.679	0.20	0.218	-0.93	0.353
Diagnoses - main ICD10: R14 Flatulence and related conditions	-0.18	0.384	0.04	0.734	-0.93	0.354
Hand grip strength (left)	0.04	0.543	-0.02	0.356	0.92	0.355
Diagnoses - main ICD10: O75 Other complications of labour and delivery, not elsewhere classified	0.27	0.250	0.03	0.793	0.92	0.355
Treatment/medication code: co-amoxiclav	-0.07	0.778	0.21	0.187	-0.92	0.356
Qualifications: NVQ or HND or HNC or equivalent	-0.03	0.791	0.08	0.117	-0.92	0.357
Longest period of depression	-0.05	0.822	0.17	0.111	-0.92	0.357
Diagnoses - main ICD10: L73 Other follicular disorders	-0.37	0.540	0.39	0.489	-0.92	0.358
Average weekly beer plus cider intake	-0.04	0.617	0.05	0.345	-0.92	0.358
Medication for cholesterol, blood pressure or diabetes: None of the above	-0.06	0.378	-0.14	0.002	0.92	0.359
Diagnoses - main ICD10: O70 Perineal laceration during delivery	0.39	0.248	0.06	0.657	0.92	0.359
Diagnoses - main ICD10: M77 Other enthesopathies	0.34	0.239	0.05	0.698	0.92	0.360
Treatment/medication code: nitrofurantoin	-0.13	0.757	0.49	0.353	-0.91	0.361
Treatment/medication code: amiodarone	0.30	0.272	0.02	0.856	0.91	0.361
Diagnoses - main ICD10: R61 Hyperhidrosis	-0.70	0.299	-0.06	0.779	-0.91	0.361
Time from waking to first cigarette	0.04	0.781	-0.11	0.100	0.91	0.362
Diagnoses - main ICD10: I45 Other conduction disorders	0.44	0.405	-0.08	0.711	0.91	0.362
Non-cancer illness code, self-reported: glaucoma	-0.18	0.103	-0.07	0.256	-0.91	0.363
Adopted as a child	0.17	0.280	0.00	1.000	0.91	0.363
Diagnoses - main ICD10: M47 Spondylosis	-0.19	0.378	0.04	0.755	-0.91	0.363
Treatment/medication code: indometacin	-0.63	0.512	0.34	0.467	-0.91	0.364
Treatment/medication code: co-dydramol	0.54	0.113	0.21	0.134	0.91	0.364
Diagnoses - main ICD10: F20 Schizophrenia	0.15	0.566	-0.12	0.397	0.91	0.365
Ever smoked	-0.02	0.687	0.04	0.218	-0.90	0.367
Basal metabolic rate	0.06	0.259	0.11	2.00E-04	-0.90	0.368
Treatment/medication code: senna product	-0.23	0.531	0.13	0.444	-0.90	0.369
Diagnoses - main ICD10: M25 Other joint disorders, not elsewhere classified	-0.30	0.518	0.16	0.455	-0.90	0.370
Treatment/medication code: zydol 50mg capsule	0.38	0.396	-0.05	0.777	0.89	0.371

Wheeze or whistling in the chest in last year	0.11	0.085	0.17	3.00E-05	-0.89	0.372
Non-cancer illness code, self-reported: emphysema/chronic bronchitis	0.34	0.005	0.20	0.068	0.89	0.372
Leg pain when walking ever disappears while walking	0.16	0.512	-0.09	0.505	0.89	0.373
Headaches for 3+ months	0.13	0.255	0.02	0.741	0.89	0.374
Illnesses of mother: Heart disease	0.14	0.172	0.24	1.33E-05	-0.89	0.374
Diagnoses - main ICD10: N23 Unspecified renal colic	-0.28	0.501	0.12	0.484	-0.89	0.376
Treatment/medication code: rhinocort 50micrograms nasal spray	0.32	0.117	0.11	0.281	0.88	0.377
Fractured bone site(s): Ankle	0.33	0.051	0.17	0.010	0.88	0.377
Non-cancer illness code, self-reported: chronic obstructive airways disease/copd	0.18	0.263	0.02	0.820	0.88	0.379
Diagnoses - main ICD10: N92 Excessive, frequent and irregular menstruation	0.26	0.133	0.08	0.467	0.88	0.379
Treatment/medication code: simvastatin	0.07	0.392	0.15	6.00E-04	-0.88	0.380
Blood clot, DVT, bronchitis, emphysema, asthma, rhinitis, eczema, allergy diagnosed by doctor: Asthma	-0.01	0.938	0.07	0.101	-0.88	0.380
Treatment/medication code: sildenafil	-0.14	0.688	0.23	0.339	-0.88	0.381
Diagnoses - main ICD10: N76 Other inflammation of vagina and vulva	-0.09	0.792	0.31	0.317	-0.88	0.381
Diagnoses - main ICD10: I65 Occlusion and stenosis of precerebral arteries, not resulting in cerebral infarction	-0.03	0.926	0.37	0.201	-0.88	0.381
Underlying (primary) cause of death: ICD10: C10.9 Oropharynx, unspecified	0.12	0.781	-0.51	0.379	0.88	0.381
Medication for cholesterol, blood pressure, diabetes, or take exogenous hormones: Oral contraceptive pill or minipill	-0.16	0.415	0.04	0.725	-0.88	0.381
Treatment/medication code: cozaar 25mg tablet	-0.44	0.144	-0.14	0.400	-0.87	0.382
Treatment/medication code: carbimazole	0.61	0.116	0.20	0.430	0.87	0.382
Non-cancer illness code, self-reported: ulcerative colitis	0.23	0.155	0.07	0.370	0.87	0.382
Treatment/medication code: vitamin a	0.36	0.251	0.07	0.573	0.87	0.383
Diagnoses - main ICD10: O16 Unspecified maternal hypertension	0.84	0.545	-0.66	0.514	0.87	0.383
Diagnoses - main ICD10: O00 Ectopic pregnancy	0.46	0.497	-0.17	0.498	0.87	0.383
Diagnoses - main ICD10: Z12 Special screening examination for neoplasms	0.26	0.403	-0.04	0.790	0.87	0.383
Diagnoses - main ICD10: M65 Synovitis and tenosynovitis	-0.12	0.523	0.06	0.509	-0.87	0.384
Non-cancer illness code, self-reported: renal failure requiring dialysis	-0.03	0.909	0.25	0.142	-0.87	0.384
Non-cancer illness code, self-reported: venous thromboembolic disease	-0.13	0.706	0.22	0.294	-0.87	0.385
Average weekly spirits intake	-0.01	0.944	0.09	0.063	-0.87	0.385

Treatment/medication code: dovonex 50micrograms/g cream	-0.12	0.801	0.47	0.337	-0.87	0.385
Non-cancer illness code, self-reported: polycystic ovaries/polycystic ovarian syndrome	-0.02	0.927	0.22	0.125	-0.87	0.386
Types of physical activity in last 4 weeks: Strenuous sports	0.04	0.613	-0.04	0.360	0.87	0.386
Diagnoses - main ICD10: S92 Fracture of foot, except ankle	0.85	0.374	0.00	0.993	0.87	0.387
Treatment/medication code: pizotifen	0.50	0.219	0.12	0.495	0.86	0.387
Diagnoses - main ICD10: Z47 Other orthopaedic follow-up care	-0.15	0.423	0.03	0.745	-0.86	0.387
Diagnoses - main ICD10: N49 Inflammatory disorders of male genital organs, not elsewhere classified	-0.30	0.535	0.17	0.494	-0.86	0.389
Diagnoses - main ICD10: E10 Insulin-dependent diabetes mellitus	0.72	0.045	0.37	0.036	0.86	0.389
Knee pain for 3+ months	0.02	0.912	0.16	0.054	-0.86	0.389
Home area population density - urban or rural: Scotland - Other Urban Area	-0.01	0.963	0.21	0.089	-0.86	0.390
Worry too long after embarrassment	0.02	0.803	0.09	0.057	-0.86	0.390
Diagnoses - main ICD10: Z01 Other special examinations and investigations of persons without complaint or reported diagnosis	0.54	0.103	0.23	0.135	0.86	0.390
Treatment/medication code: codeine	0.14	0.392	-0.02	0.834	0.86	0.390
Treatment/medication code: elleste-solo 1mg tablet	0.13	0.658	-0.16	0.348	0.86	0.391
Treatment/medication code: priadel 200mg m/r tablet	-0.31	0.240	-0.06	0.663	-0.86	0.391
Treatment/medication code: vitamin k product	-0.15	0.620	0.15	0.399	-0.85	0.393
Frequency of stair climbing in last 4 weeks	0.09	0.257	0.02	0.666	0.85	0.393
Non-cancer illness code, self-reported: muscle or soft tissue injuries	-0.18	0.485	0.09	0.623	-0.85	0.393
Non-cancer illness code, self-reported: polycystic kidney	-0.36	0.574	0.33	0.504	-0.85	0.394
Arm fat-free mass (right)	0.05	0.323	0.10	6.00E-04	-0.85	0.394
Cancer code, self-reported: uterine/endometrial cancer	-0.42	0.321	-0.03	0.858	-0.85	0.395
Diagnoses - main ICD10: M24 Other specific joint derangements	-0.11	0.602	0.10	0.441	-0.85	0.395
Non-cancer illness code, self-reported: interstitial lung disease	0.30	0.179	0.09	0.396	0.85	0.396
Diagnoses - main ICD10: D27 Benign neoplasm of ovary	0.01	0.959	-0.19	0.061	0.85	0.396
Treatment/medication code: mometasone	-0.07	0.813	0.21	0.199	-0.85	0.397
Treatment/medication code: thyroxine sodium	0.55	0.169	1.14	0.046	-0.85	0.397
Non-cancer illness code, self-reported: appendicitis	-0.04	0.866	0.18	0.160	-0.85	0.398
Leg pain on walking	0.12	0.179	0.21	1.00E-04	-0.84	0.400
Treatment/medication code: thiamine preparation	-0.11	0.600	0.10	0.447	-0.84	0.401

Length of longest manic/irritable episode	0.24	0.473	-0.06	0.644	0.84	0.401
Treatment/medication code: amitriptyline	0.06	0.634	0.17	0.012	-0.84	0.402
Cancer code, self-reported: breast cancer	-0.12	0.344	0.00	0.948	-0.84	0.403
Type of tobacco currently smoked: Cigars or pipes	-0.28	0.570	0.18	0.468	-0.84	0.403
Diagnoses - main ICD10: S61 Open wound of wrist and hand	0.58	0.283	0.07	0.802	0.83	0.405
Non-cancer illness code, self-reported: pericardial problem	-0.39	0.447	0.07	0.737	-0.83	0.406
Treatment/medication code: sustanon 100 oily injection	0.35	0.422	-0.04	0.816	0.83	0.407
Diagnoses - main ICD10: C92 Myeloid leukaemia	-0.27	0.510	0.10	0.559	-0.83	0.407
Treatment/medication code: salmeterol product	0.42	0.049	0.22	0.061	0.83	0.409
Diagnoses - main ICD10: N50 Other disorders of male genital organs	0.26	0.288	0.03	0.828	0.82	0.410
Vitamin and mineral supplements: Vitamin E	0.00	0.994	0.14	0.076	-0.82	0.410
Non-cancer illness code, self-reported: iritis	0.19	0.497	-0.07	0.628	0.82	0.410
Illnesses of father: Prostate cancer	-0.06	0.658	-0.19	0.010	0.82	0.413
Reproduciblity of spirometry measurement using ERS/ATS criteria	-0.01	0.953	0.11	0.155	-0.81	0.415
Treatment/medication code: cymalon cranberry 1.5g/5ml liquid	0.33	0.301	0.05	0.744	0.81	0.416
Past tobacco smoking	0.02	0.769	-0.04	0.247	0.81	0.417
Medication for cholesterol, blood pressure, diabetes, or take exogenous hormones: Cholesterol lowering medication	0.11	0.198	0.19	2.05E-05	-0.81	0.418
Vitamin and mineral supplements: None of the above	-0.11	0.165	-0.04	0.383	-0.81	0.418
Diagnoses - main ICD10: J35 Chronic diseases of tonsils and adenoids	0.33	0.458	-0.09	0.738	0.81	0.418
Diagnoses - main ICD10: J18 Pneumonia, organism unspecified	-0.05	0.840	0.18	0.166	-0.81	0.419
Doctor diagnosed emphysema	0.27	0.259	0.06	0.551	0.81	0.419
Treatment/medication code: naproxen	0.05	0.842	0.28	0.048	-0.81	0.419
Doctor diagnosed sarcoidosis	0.70	0.023	0.41	0.032	0.81	0.420
Diagnoses - main ICD10: L03 Cellulitis	0.40	0.058	0.20	0.143	0.81	0.420
Treatment/medication code: food supplement/plant/herbal extract	0.42	0.130	0.16	0.316	0.81	0.420
Treatment/medication code: imigran 50mg tablet	0.26	0.198	0.08	0.459	0.80	0.421
Non-cancer illness code, self-reported: fracture vertebra / crush fracture / vertebral collapse	-0.40	0.562	0.20	0.483	-0.80	0.422
Medication for pain relief, constipation, heartburn: Omeprazole (e.g. Zanprol)	0.08	0.395	0.16	2.00E-04	-0.80	0.423
Reason for reducing amount of alcohol drunk: Doctor's advice	-0.17	0.497	0.05	0.662	-0.80	0.423

Arm predicted mass (right)	0.05	0.318	0.10	7.00E-04	-0.80	0.424
Treatment/medication code: dovobet ointment	-0.02	0.953	0.30	0.181	-0.80	0.424
Frequency of strenuous sports in last 4 weeks	0.12	0.489	-0.04	0.691	0.80	0.425
Years of cough on most days	-0.43	0.178	-0.14	0.386	-0.80	0.425
Treatment/medication code: diclomax sr 75mg m/r capsule	0.31	0.255	0.06	0.666	0.80	0.426
Non-cancer illness code, self-reported: iron deficiency anaemia	0.00	0.984	0.17	0.085	-0.79	0.427
Treatment/medication code: celiprolol	0.39	0.399	-0.01	0.976	0.79	0.428
Diagnoses - main ICD10: J46 Status asthmaticus	-0.12	0.697	0.16	0.353	-0.79	0.428
Diagnoses - main ICD10: C15 Malignant neoplasm of oesophagus	0.23	0.387	0.00	0.993	0.79	0.429
Treatment/medication code: timolol 0.25% eye drops	1.20	0.452	-0.08	0.784	0.79	0.430
Diagnoses - main ICD10: O69 Labour and delivery complicated by umbilical cord complications	0.54	0.371	0.04	0.835	0.79	0.430
Diagnoses - main ICD10: K51 Ulcerative colitis	0.26	0.139	0.11	0.185	0.79	0.432
Smoking status: Previous	-0.02	0.748	0.04	0.276	-0.78	0.433
Treatment/medication code: isosorbide dinitrate	-0.26	0.330	-0.03	0.834	-0.78	0.433
Age of primiparous women at birth of child	-0.05	0.725	-0.18	0.006	0.78	0.433
Treatment/medication code: diprosalic ointment	-0.19	0.455	0.03	0.808	-0.78	0.436
Diagnoses - main ICD10: C80 Malignant neoplasm without specification of site	-0.13	0.622	0.10	0.483	-0.78	0.436
Forced vital capacity (FVC), Best measure	-0.07	0.165	-0.03	0.282	-0.78	0.437
Illnesses of siblings: Parkinson's disease	-0.91	0.375	-0.08	0.790	-0.78	0.437
Non-cancer illness code, self-reported: measles / morbillivirus	0.07	0.809	-0.20	0.256	0.78	0.437
Diagnoses - main ICD10: N36 Other disorders of urethra	-0.30	0.492	0.07	0.715	-0.78	0.438
Non-cancer illness code, self-reported: asthma	0.01	0.906	0.07	0.077	-0.78	0.438
Treatment/medication code: amantadine	0.04	0.929	-0.41	0.319	0.78	0.438
Treatment/medication code: celecoxib	0.04	0.928	0.60	0.305	-0.78	0.438
Illnesses of father: Parkinson's disease	0.15	0.569	-0.08	0.554	0.77	0.439
Illnesses of mother: High blood pressure	0.16	0.077	0.08	0.099	0.77	0.441
Underlying (primary) cause of death: ICD10: I73.9 Peripheral vascular disease, unspecified	0.59	0.218	0.19	0.361	0.77	0.441
Illnesses of mother: Severe depression	0.04	0.787	0.16	0.019	-0.77	0.442
Treatment/medication code: xalacom 0.005%/0.5% eye drops	-0.16	0.406	0.01	0.956	-0.77	0.443

Diagnoses - main ICD10: J90 Pleural effusion, not elsewhere classified	-0.70	0.396	-0.05	0.798	-0.77	0.443
Diagnoses - main ICD10: S62 Fracture at wrist and hand level	-0.01	0.960	0.23	0.121	-0.77	0.444
Treatment/medication code: lisinopril	0.12	0.258	0.02	0.726	0.77	0.444
Non-cancer illness code, self-reported: hypopituitarism	0.14	0.710	0.49	0.080	-0.76	0.444
Diagnoses - main ICD10: J86 Pyothorax	0.36	0.370	0.02	0.934	0.76	0.445
Diagnoses - main ICD10: R19 Other symptoms and signs involving the digestive system and abdomen	-0.18	0.715	0.26	0.393	-0.76	0.445
Non-cancer illness code, self-reported: hepatitis b	-0.41	0.192	-0.14	0.378	-0.76	0.445
Treatment/medication code: glyceryl trinitrate	0.08	0.811	0.40	0.100	-0.76	0.446
Treatment/medication code: atenolol	0.23	0.030	0.13	0.069	0.76	0.446
Treatment/medication code: mirapexin 0.088mg tablet	0.63	0.183	0.19	0.590	0.76	0.449
Diagnoses - main ICD10: Z35 Supervision of high-risk pregnancy	0.28	0.269	0.06	0.696	0.76	0.449
Underlying (primary) cause of death: ICD10: C66 Malignant neoplasm of ureter	0.19	0.525	-0.06	0.660	0.76	0.449
Diabetes diagnosed by doctor	0.12	0.104	0.18	1.61E-05	-0.76	0.450
Worrier / anxious feelings	0.03	0.653	0.10	0.051	-0.75	0.451
Diagnoses - main ICD10: G24 Dystonia	0.44	0.508	-0.11	0.717	0.75	0.451
Diagnoses - main ICD10: L90 Atrophic disorders of skin	-0.52	0.506	0.10	0.698	-0.75	0.451
Mineral and other dietary supplements: Calcium	-0.01	0.917	0.07	0.149	-0.75	0.451
Non-cancer illness code, self-reported: ovarian cyst or cysts	-0.09	0.796	0.25	0.362	-0.75	0.452
Non-cancer illness code, self-reported: liver failure/cirrhosis	0.32	0.263	0.08	0.545	0.75	0.453
Treatment/medication code: beclomethasone	-0.26	0.503	0.10	0.723	-0.75	0.453
Non-cancer illness code, self-reported: high cholesterol	0.10	0.194	0.16	1.00E-04	-0.75	0.453
Vascular/heart problems diagnosed by doctor: Angina	0.11	0.225	0.18	4.45E-05	-0.75	0.454
Diagnoses - main ICD10: A87 Viral meningitis	0.25	0.535	-0.09	0.669	0.75	0.454
Illnesses of mother: Lung cancer	-0.12	0.530	0.04	0.677	-0.75	0.455
Non-cancer illness code, self-reported: fracture upper arm / humerus / elbow	-0.07	0.787	0.15	0.255	-0.75	0.455
Cancer code, self-reported: oesophageal cancer	0.21	0.576	-0.10	0.570	0.74	0.457
Treatment/medication code: estradot 25micrograms patch	-0.24	0.671	0.28	0.495	-0.74	0.457
Number of full brothers	-0.02	0.831	0.06	0.272	-0.74	0.458
Diagnoses - main ICD10: H81 Disorders of vestibular function	-0.30	0.673	0.32	0.466	-0.74	0.458

Medication for cholesterol, blood pressure, diabetes, or take exogenous hormones: Blood pressure medication	0.17	0.024	0.10	0.068	0.74	0.458
Diagnoses - main ICD10: S30 Superficial injury of abdomen, lower back and pelvis	-0.24	0.507	0.06	0.740	-0.74	0.458
Diagnoses - main ICD10: N83 Noninflammatory disorders of ovary, Fallopian tube and broad ligament	0.43	0.328	0.04	0.900	0.74	0.459
Cancer diagnosed by doctor	-0.01	0.933	-0.14	0.088	0.74	0.460
Non-cancer illness code, self-reported: bowel / intestinal obstruction	0.35	0.509	-0.08	0.738	0.74	0.460
Standing height	0.04	0.331	0.01	0.775	0.74	0.461
Diagnoses - main ICD10: I63 Cerebral infarction	-0.01	0.983	0.24	0.113	-0.74	0.461
Diagnoses - main ICD10: O68 Labour and delivery complicated by foetal stress [distress]	0.10	0.724	-0.13	0.365	0.74	0.462
Underlying (primary) cause of death: ICD10: C23 Malignant neoplasm of gallbladder	-0.20	0.375	-0.01	0.951	-0.74	0.462
Non-cancer illness code, self-reported: diabetes	0.12	0.116	0.18	2.35E-05	-0.74	0.462
Diagnoses - main ICD10: J44 Other chronic obstructive pulmonary disease	0.32	0.064	0.18	0.073	0.73	0.466
Treatment/medication code: ventolin 100micrograms inhaler	-0.07	0.500	0.01	0.781	-0.73	0.467
Current employment status: Unemployed	0.01	0.937	0.15	0.091	-0.73	0.467
Diagnoses - main ICD10: K86 Other diseases of pancreas	-0.07	0.791	0.13	0.246	-0.72	0.471
Treatment/medication code: viagra 50mg tablet	-0.31	0.658	0.35	0.555	-0.72	0.472
Treatment/medication code: metronidazole product	0.00	0.993	0.30	0.266	-0.72	0.472
Diagnoses - main ICD10: K40 Inguinal hernia	-0.04	0.705	0.05	0.468	-0.72	0.473
Likelihood of resuming smoking	0.17	0.418	0.00	0.995	0.72	0.473
Diagnoses - main ICD10: C43 Malignant melanoma of skin	0.12	0.722	-0.16	0.393	0.72	0.473
Treatment/medication code: zimovane ls 3.75mg tablet	-0.20	0.663	0.16	0.446	-0.72	0.473
Treatment/medication code: aminophylline	-0.20	0.370	-0.02	0.820	-0.72	0.473
Treatment/medication code: norgeston tablet	-0.23	0.612	0.14	0.559	-0.72	0.473
Treatment/medication code: leflunomide	-0.38	0.314	-0.08	0.677	-0.72	0.474
Treatment/medication code: serevent 25mcg inhaler	0.31	0.159	0.14	0.177	0.71	0.475
Treatment/medication code: paracetamol	0.12	0.108	0.18	1.21E-06	-0.71	0.477
Non-cancer illness code, self-reported: leg claudication/ intermittent claudication	-0.05	0.806	0.12	0.298	-0.71	0.478
Treatment/medication code: furosemide	0.06	0.785	0.25	0.135	-0.71	0.478
Treatment/medication code: vitamin b6 preparation	0.03	0.906	-0.17	0.172	0.71	0.478

Eye problems/disorders: Glaucoma	-0.16	0.166	-0.07	0.240	-0.71	0.479
Hearing difficulty/problems with background noise	0.06	0.398	0.11	5.00E-04	-0.71	0.480
Non-cancer illness code, self-reported: anal problem	0.81	0.399	0.11	0.592	0.71	0.480
Treatment/medication code: perindopril+indapamide	-0.14	0.794	0.33	0.401	-0.71	0.481
Treatment/medication code: sodium cromoglicate	0.26	0.295	0.07	0.564	0.71	0.481
Treatment/medication code: atrovent 20micrograms inhaler	0.34	0.214	0.13	0.285	0.70	0.481
Non-cancer illness code, self-reported: inguinal hernia	-0.14	0.616	0.09	0.603	-0.70	0.483
Non-cancer illness code, self-reported: liver/biliary/pancreas problem	0.07	0.785	0.27	0.078	-0.70	0.483
Treatment/medication code: climaval 1mg tablet	0.06	0.847	-0.20	0.277	0.70	0.483
Diagnoses - main ICD10: I50 Heart failure	-0.12	0.650	0.09	0.499	-0.70	0.483
Treatment/medication code: lactulose product	0.11	0.712	0.34	0.048	-0.70	0.483
Vitamin and mineral supplements: Vitamin A	0.41	0.064	0.22	0.131	0.70	0.484
Treatment/medication code: warfarin	0.36	0.060	0.21	0.021	0.70	0.484
Reason former drinker stopped drinking alcohol: Doctor's advice	-0.03	0.931	0.28	0.247	-0.70	0.485
Doctor diagnosed tuberculosis	0.45	0.208	0.17	0.321	0.70	0.486
Diagnoses - main ICD10: N20 Calculus of kidney and ureter	-0.11	0.423	0.00	0.980	-0.70	0.486
Treatment/medication code: lansoprazole	0.08	0.510	0.16	0.002	-0.70	0.486
Bilateral oophorectomy (both ovaries removed)	-0.09	0.434	0.00	0.988	-0.70	0.487
Treatment/medication code: nexium 20mg tablet	0.28	0.369	0.05	0.712	0.69	0.489
Treatment/medication code: nasonex 0.05% aqueous nasal spray	-0.09	0.728	0.10	0.402	-0.69	0.490
Diagnoses - main ICD10: N94 Pain and other conditions associated with female genital organs and menstrual cycle	0.57	0.559	-0.17	0.705	0.69	0.491
Illness, injury, bereavement, stress in last 2 years: Death of a spouse or partner	-0.03	0.904	0.17	0.174	-0.69	0.492
Diagnoses - main ICD10: M50 Cervical disk disorders	-0.23	0.454	0.01	0.950	-0.69	0.492
Non-cancer illness code, self-reported: testicular problems (not cancer)	0.47	0.169	0.20	0.252	0.69	0.493
Non-cancer illness code, self-reported: peritonitis	0.37	0.090	0.20	0.068	0.69	0.493
Underlying (primary) cause of death: ICD10: K63.1 Perforation of intestine (nontraumatic)	0.16	0.693	-0.15	0.454	0.68	0.494
Pain type(s) experienced in last month: Neck or shoulder pain	0.11	0.116	0.17	6.22E-07	-0.68	0.495
Treatment/medication code: qvar 50 inhaler	0.16	0.701	-0.18	0.514	0.68	0.497
Manic/hyper symptoms: All of the above	0.20	0.466	-0.01	0.935	0.68	0.497

Treatment/medication code: phenytoin	-0.14	0.491	0.01	0.898	-0.68	0.498
Treatment/medication code: depakote 250mg e/c tablet	0.99	0.409	0.15	0.611	0.68	0.498
Mother's age at death	-0.03	0.800	-0.12	0.116	0.68	0.499
Illnesses of father: Diabetes	0.16	0.130	0.08	0.242	0.68	0.499
Treatment/medication code: dipyridamole	0.23	0.355	0.04	0.749	0.68	0.499
Fluid intelligence score	-0.02	0.738	-0.06	0.046	0.68	0.499
Non-cancer illness code, self-reported: pernicious anaemia	0.54	0.025	0.36	0.007	0.68	0.500
Leg fat-free mass (left)	0.06	0.201	0.10	5.00E-04	-0.68	0.500
Home area population density - urban or rural: England/Wales - Town and Fringe - less sparse	0.56	0.611	-0.37	0.655	0.67	0.500
Treatment/medication code: spasmonal 60mg capsule	0.39	0.374	0.07	0.687	0.67	0.500
Non-cancer illness code, self-reported: cervical polyps	-0.42	0.491	0.01	0.964	-0.67	0.501
Distance between home and job workplace	0.11	0.480	-0.01	0.889	0.67	0.501
Treatment/medication code: ginkgo forte tablet	-0.13	0.606	0.06	0.637	-0.67	0.503
Diagnoses - main ICD10: I42 Cardiomyopathy	-0.05	0.835	0.12	0.270	-0.67	0.504
Treatment/medication code: iron sulphate	0.35	0.361	0.07	0.702	0.67	0.505
Diagnoses - main ICD10: J02 Acute pharyngitis	-0.20	0.718	0.37	0.570	-0.67	0.505
Non-cancer illness code, self-reported: stomach disorder	0.54	0.260	0.20	0.269	0.67	0.505
Medication for cholesterol, blood pressure, diabetes, or take exogenous hormones: Insulin	0.28	0.089	0.41	2.99E-05	-0.67	0.506
Non-cancer illness code, self-reported: pituitary adenoma/tumour	0.31	0.597	-0.13	0.664	0.67	0.506
Impedance of whole body	-0.04	0.437	-0.08	0.024	0.66	0.506
Non-cancer illness code, self-reported: labyrinthitis	0.22	0.709	-0.32	0.570	0.66	0.507
Why stopped smoking: Doctor's advice	0.24	0.212	0.10	0.247	0.66	0.507
Diagnoses - main ICD10: N47 Redundant prepuce, phimosis and paraphimosis	0.51	0.289	0.16	0.413	0.66	0.509
Underlying (primary) cause of death: ICD10: G31.8 Other specified degenerative diseases of nervous system	-0.13	0.775	-0.75	0.362	0.66	0.509
Diagnoses - main ICD10: O36 Maternal care for other known or suspected foetal problems	0.10	0.733	-0.12	0.427	0.66	0.511
Treatment/medication code: letrozole	0.38	0.102	0.21	0.080	0.66	0.512
Treatment/medication code: pariet 10mg e/c tablet	-0.10	0.781	0.17	0.372	-0.66	0.512

Treatment/medication code: adcal 600mg chewable tablet	0.07	0.818	-0.16	0.345	0.66	0.512
Treatment/medication code: synalar 1:10 cream	-0.25	0.665	0.19	0.576	-0.66	0.512
Manic/hyper symptoms: I was more active than usual	-0.21	0.409	-0.03	0.769	-0.65	0.513
Treatment/medication code: adizem-xl plus m/r capsule	0.42	0.357	0.10	0.639	0.65	0.513
Non-cancer illness code, self-reported: hypertrophic cardiomyopathy (hcm / hocm)	0.08	0.793	-0.14	0.329	0.65	0.514
Doctor diagnosed COPD (chronic obstructive pulmonary disease)	0.33	0.223	0.13	0.298	0.65	0.514
Diagnoses - main ICD10: Z80 Family history of malignant neoplasm	0.23	0.516	-0.03	0.878	0.65	0.514
Diagnoses - main ICD10: S76 Injury of muscle and tendon at hip and thigh level	-0.35	0.279	-0.12	0.400	-0.65	0.514
Treatment/medication code: cardura 1mg tablet	-0.21	0.409	-0.02	0.857	-0.65	0.515
Diagnoses - main ICD10: R54 Senility	-0.37	0.425	-0.02	0.924	-0.65	0.516
Diagnoses - main ICD10: J13 Pneumonia due to Streptococcus pneumoniae	-0.43	0.530	0.05	0.856	-0.65	0.516
Treatment/medication code: hyoscine butylbromide	0.05	0.797	0.21	0.064	-0.65	0.516
Non-cancer illness code, self-reported: abnormal smear (cervix)	0.45	0.559	-0.09	0.775	0.65	0.517
Diagnoses - main ICD10: K59 Other functional intestinal disorders	0.06	0.849	0.33	0.161	-0.65	0.517
Non-cancer illness code, self-reported: gestational hypertension/pre-eclampsia	0.30	0.213	0.13	0.264	0.65	0.518
Diagnoses - main ICD10: M53 Other dorsopathies, not elsewhere classified	0.20	0.386	0.03	0.744	0.65	0.518
Hearing aid user	0.03	0.783	0.12	0.038	-0.65	0.518
Current employment status: Doing unpaid or voluntary work	0.06	0.627	-0.03	0.645	0.65	0.518
Non-cancer illness code, self-reported: fracture lower leg / ankle	-0.57	0.627	0.25	0.604	-0.65	0.519
Treatment/medication code: brimonidine tartrate	-0.09	0.832	0.26	0.438	-0.64	0.519
Non-cancer illness code, self-reported: pyloric stenosis	0.17	0.413	0.02	0.879	0.64	0.519
Non-cancer illness code, self-reported: fibrosing alveolitis/unspecified alveolitis	0.42	0.345	0.11	0.552	0.64	0.520
Diagnoses - main ICD10: M51 Other intervertebral disk disorders	0.06	0.717	0.19	0.041	-0.64	0.521
Treatment/medication code: bisoprolol	0.04	0.786	0.15	0.042	-0.64	0.522
Diagnoses - main ICD10: M18 Arthrosis of first carpometacarpal joint	-0.03	0.894	0.15	0.243	-0.64	0.522
Tinnitus: Yes, but not now, but have in the past	0.26	0.088	0.15	0.099	0.64	0.522
Fractured bone site(s): Leg	0.33	0.159	0.17	0.116	0.64	0.522
Underlying (primary) cause of death: ICD10: I11.0 Hypertensive heart disease with (congestive) heart failure	-0.25	0.512	0.01	0.941	-0.64	0.522
Diagnoses - main ICD10: F33 Recurrent depressive disorder	0.58	0.500	0.00	0.985	0.64	0.522

Treatment/medication code: pilocarpine	-0.18	0.357	-0.04	0.654	-0.64	0.522
Diagnoses - main ICD10: R50 Fever of unknown origin	0.01	0.979	0.17	0.129	-0.64	0.522
Illnesses of mother: Stroke	0.22	0.206	0.09	0.351	0.64	0.523
Treatment/medication code: calcium+ergocalciferol tablet	0.12	0.774	-0.21	0.488	0.64	0.524
Diagnoses - main ICD10: C67 Malignant neoplasm of bladder	0.06	0.794	-0.11	0.410	0.64	0.525
Non-cancer illness code, self-reported: spontaneous pneumothorax/recurrent pneumothorax	-0.09	0.735	0.10	0.468	-0.63	0.526
Diagnoses - main ICD10: C61 Malignant neoplasm of prostate	0.18	0.283	0.06	0.436	0.63	0.526
Number of days/week of vigorous physical activity 10+ minutes	0.03	0.748	-0.03	0.436	0.63	0.527
Bipolar disorder status	-0.01	0.967	0.20	0.256	-0.63	0.527
Non-cancer illness code, self-reported: femoral hernia	0.47	0.303	0.16	0.398	0.63	0.527
Diagnoses - main ICD10: O32 Maternal care for known or suspected malpresentation of foetus	0.07	0.812	-0.16	0.403	0.63	0.528
Doctor diagnosed asthma	-0.07	0.473	0.00	0.987	-0.63	0.529
Diagnoses - main ICD10: G50 Disorders of trigeminal nerve	-0.62	0.526	0.01	0.953	-0.63	0.529
Leg predicted mass (left)	0.06	0.184	0.10	5.00E-04	-0.63	0.529
Treatment/medication code: climagest 1mg tablet	-0.99	0.562	0.10	0.743	-0.63	0.530
Cancer code, self-reported: eye and/or adnexal cancer	-0.09	0.729	0.10	0.467	-0.63	0.531
Non-cancer illness code, self-reported: atrial fibrillation	0.08	0.620	-0.03	0.684	0.62	0.533
Treatment/medication code: budesonide	0.20	0.485	0.00	0.981	0.62	0.533
Treatment/medication code: bimatoprost	-0.44	0.273	-0.17	0.307	-0.62	0.534
Treatment/medication code: co-tenidone	0.28	0.317	0.09	0.516	0.62	0.534
Cancer code, self-reported: skin cancer	0.31	0.573	-0.05	0.786	0.62	0.534
Treatment/medication code: chlorphenamine	-0.34	0.115	-0.19	0.047	-0.62	0.536
Underlying (primary) cause of death: ICD10: C78.6 Secondary malignant neoplasm of retroperitoneum and peritoneum	-0.24	0.359	-0.06	0.682	-0.62	0.536
Underlying (primary) cause of death: ICD10: N39.0 Urinary tract infection, site not specified	-0.32	0.448	-0.03	0.888	-0.62	0.536
Treatment/medication code: rabeprazole sodium	0.32	0.488	0.01	0.950	0.62	0.536
Diagnoses - main ICD10: N40 Hyperplasia of prostate	0.15	0.385	0.03	0.728	0.62	0.536
Diagnoses - main ICD10: Z44 Fitting and adjustment of external prosthetic device	1.09	0.478	0.12	0.666	0.62	0.537

Reason for glasses/contact lenses: For short-sightedness, i.e. only or mainly for distance viewing such as driving, cinema etc (called 'myopia')	0.02	0.800	-0.04	0.411	0.62	0.538
Maximum workload during fitness test	0.02	0.915	-0.09	0.252	0.62	0.538
Illnesses of mother: None of the above (group 1)	-0.24	0.020	-0.17	8.00E-04	-0.61	0.539
Non-cancer illness code, self-reported: hereditary/genetic haematological disorder	-0.02	0.948	0.21	0.242	-0.61	0.541
Diagnoses - main ICD10: H53 Visual disturbances	0.07	0.693	0.20	0.047	-0.61	0.542
Diagnoses - main ICD10: G56 Mononeuropathies of upper limb	0.09	0.388	0.16	7.00E-04	-0.61	0.542
Illnesses of siblings: Diabetes	0.07	0.475	0.15	0.015	-0.61	0.542
Treatment/medication code: protopic 0.03% ointment	-0.25	0.746	0.38	0.585	-0.61	0.543
Non-cancer illness code, self-reported: varicose veins	-0.08	0.646	0.04	0.647	-0.61	0.544
Treatment/medication code: omacor 1g capsule	0.37	0.601	-0.10	0.751	0.61	0.545
Diagnoses - main ICD10: K09 Cysts of oral region, not elsewhere classified	-0.10	0.857	0.34	0.472	-0.60	0.546
Gestational diabetes only	-0.37	0.436	-0.06	0.759	-0.60	0.546
Treatment/medication code: phenoxymethylpenicillin	-0.42	0.561	0.05	0.863	-0.60	0.546
Part of a multiple birth	0.05	0.854	-0.14	0.371	0.60	0.546
Cochlear implant	0.16	0.622	0.40	0.089	-0.60	0.547
Non-cancer illness code, self-reported: eczema/dermatitis	-0.02	0.906	0.08	0.325	-0.60	0.548
Reason for glasses/contact lenses: Other eye condition	0.11	0.759	-0.14	0.504	0.60	0.548
Diagnoses - main ICD10: G58 Other mononeuropathies	0.45	0.521	0.01	0.982	0.60	0.548
Treatment/medication code: buscopan 10mg tablet	0.01	0.977	0.28	0.298	-0.60	0.548
Current tobacco smoking	0.01	0.882	0.06	0.180	-0.60	0.549
Treatment/medication code: diazepam	0.02	0.913	0.17	0.150	-0.60	0.549
Diagnoses - main ICD10: N81 Female genital prolapse	0.17	0.222	0.08	0.275	0.60	0.549
Treatment/medication code: colchicine	-0.14	0.543	0.01	0.904	-0.60	0.550
Whole body fat-free mass	0.06	0.225	0.09	6.00E-04	-0.60	0.550
Former alcohol drinker	-0.11	0.412	-0.02	0.773	-0.60	0.550
Diagnoses - main ICD10: N30 Cystitis	0.42	0.393	0.10	0.607	0.60	0.550
Treatment/medication code: minims artificial tears single-use eye drops	0.42	0.332	0.14	0.451	0.60	0.551
Diagnoses - main ICD10: S00 Superficial injury of head	0.36	0.559	-0.03	0.894	0.60	0.552
Diagnoses - main ICD10: Z46 Fitting and adjustment of other devices	0.00	0.985	0.13	0.221	-0.60	0.552

Non-cancer illness code, self-reported: osteoarthritis	0.11	0.301	0.17	4.00E-04	-0.59	0.553
Treatment/medication code: amitriptyline hydrochloride+perphenazine 10mg/2mg tablet	-0.12	0.717	0.10	0.518	-0.59	0.554
Non-cancer illness code, self-reported: angina	0.12	0.170	0.18	7.37E-05	-0.59	0.554
Treatment/medication code: bendrofluazide	0.18	0.253	0.07	0.509	0.59	0.556
Treatment/medication code: prempak 0.625 tablet	0.53	0.343	0.18	0.396	0.59	0.556
Non-cancer illness code, self-reported: pericarditis	0.01	0.970	0.19	0.217	-0.59	0.557
Diagnoses - main ICD10: Z34 Supervision of normal pregnancy	0.20	0.568	-0.03	0.872	0.59	0.558
Hair/balding pattern: Pattern 4	0.08	0.124	0.05	0.089	0.58	0.559
Non-cancer illness code, self-reported: pneumonia	0.21	0.321	0.07	0.453	0.58	0.559
Treatment/medication code: fentanyl	-0.05	0.871	0.16	0.309	-0.58	0.560
Heel bone mineral density (BMD) T-score, automated	0.01	0.884	-0.03	0.456	0.58	0.560
Treatment/medication code: morphine	0.11	0.779	-0.14	0.474	0.58	0.560
Non-cancer illness code, self-reported: polio / poliomyelitis	0.10	0.654	0.26	0.072	-0.58	0.560
Treatment/medication code: oxytetracycline	0.25	0.621	-0.09	0.757	0.58	0.560
Non-cancer illness code, self-reported: bladder problem (not cancer)	-0.09	0.721	0.08	0.579	-0.58	0.561
Diagnoses - main ICD10: K13 Other diseases of lip and oral mucosa	0.38	0.439	0.07	0.778	0.58	0.561
Chest pain or discomfort	0.19	0.014	0.24	2.58E-11	-0.58	0.561
Diagnoses - main ICD10: J45 Asthma	0.20	0.516	0.43	0.092	-0.58	0.562
Treatment/medication code: procyclidine	-0.34	0.470	-0.05	0.788	-0.58	0.562
Treatment/medication code: piroxicam	0.29	0.448	0.05	0.788	0.58	0.562
Diagnoses - main ICD10: I73 Other peripheral vascular diseases	0.43	0.239	0.19	0.326	0.58	0.563
Whole body water mass	0.06	0.223	0.09	7.00E-04	-0.58	0.563
Leg predicted mass (right)	0.06	0.185	0.10	6.00E-04	-0.58	0.563
Underlying (primary) cause of death: ICD10: C02.9 Tongue, unspecified	-0.83	0.556	-0.01	0.980	-0.57	0.565
Diagnoses - main ICD10: T40 Poisoning by narcotics and psychodysleptics [hallucinogens]	-0.03	0.901	0.14	0.315	-0.57	0.565
Treatment/medication code: adalat 5mg capsule	0.22	0.536	0.48	0.105	-0.57	0.566
Current employment status: Retired	0.08	0.591	-0.02	0.831	0.57	0.566
Treatment/medication code: salmeterol+fluticasone propionate	-0.13	0.725	0.14	0.631	-0.57	0.567

Diagnoses - main ICD10: I51 Complications and ill-defined descriptions of heart disease	0.18	0.540	-0.01	0.965	0.57	0.568
Illnesses of mother: None of the above (group 2)	0.09	0.492	0.01	0.919	0.57	0.568
Chest pain felt outside physical activity	-0.12	0.808	0.33	0.594	-0.57	0.569
Treatment/medication code: mesalazine	0.10	0.742	0.32	0.150	-0.57	0.569
Chest pain or discomfort when walking uphill or hurrying	0.01	0.954	0.13	0.129	-0.57	0.570
Underlying (primary) cause of death: ICD10: C26.0 Intestinal tract, part unspecified	-0.28	0.420	-0.06	0.686	-0.57	0.570
Vitamin and mineral supplements: Vitamin C	0.10	0.368	0.03	0.688	0.57	0.571
Underlying (primary) cause of death: ICD10: C14.0 Pharynx, unspecified	0.16	0.719	-0.13	0.610	0.57	0.571
Treatment/medication code: Free-text entry, unable to be coded	0.15	0.478	0.30	0.041	-0.57	0.571
Illness, injury, bereavement, stress in last 2 years: Serious illness, injury or assault of a close relative	0.03	0.820	0.11	0.156	-0.56	0.572
Diagnoses - main ICD10: R13 Dysphagia	-0.33	0.704	0.22	0.622	-0.56	0.573
Non-cancer illness code, self-reported: parkinsons disease	0.14	0.680	-0.08	0.679	0.56	0.573
Diagnoses - main ICD10: E11 Non-insulin-dependent diabetes mellitus	0.37	0.113	0.21	0.155	0.56	0.574
Chest pain felt during physical activity	0.24	0.296	0.10	0.379	0.56	0.575
Treatment/medication code: risedronate sodium	0.59	0.219	0.29	0.249	0.56	0.575
Treatment/medication code: bezafibrate	0.05	0.886	0.29	0.224	-0.56	0.576
Diagnoses - main ICD10: N17 Acute renal failure	0.39	0.460	0.06	0.797	0.56	0.576
Non-cancer illness code, self-reported: brain haemorrhage	-0.16	0.766	0.19	0.572	-0.56	0.576
Treatment/medication code: latanoprost	0.09	0.656	-0.04	0.734	0.56	0.576
Diagnoses - main ICD10: C25 Malignant neoplasm of pancreas	-0.17	0.378	-0.05	0.599	-0.56	0.576
Difficulty not smoking for 1 day	0.02	0.882	0.11	0.100	-0.56	0.576
Leg fat-free mass (right)	0.07	0.171	0.10	6.00E-04	-0.56	0.578
Non-cancer illness code, self-reported: multiple sclerosis	-0.07	0.711	0.05	0.629	-0.56	0.578
Non-cancer illness code, self-reported: fracture finger	-0.14	0.704	0.11	0.667	-0.56	0.579
Non-cancer illness code, self-reported: arthritis (nos)	-0.02	0.946	0.15	0.326	-0.55	0.579
Diagnoses - main ICD10: N93 Other abnormal uterine and vaginal bleeding	0.50	0.602	-0.06	0.854	0.55	0.580
Treatment/medication code: zapain caplet	0.34	0.612	-0.08	0.824	0.55	0.581
Qualifications: O levels/GCSEs or equivalent	0.01	0.838	-0.03	0.466	0.55	0.581
Diagnoses - main ICD10: N41 Inflammatory diseases of prostate	0.02	0.965	-0.26	0.387	0.55	0.582

Diagnoses - main ICD10: S72 Fracture of femur	0.26	0.298	0.10	0.512	0.55	0.582
Diagnoses - main ICD10: N84 Polyp of female genital tract	0.00	0.989	0.14	0.281	-0.55	0.583
Potassium in urine	0.03	0.717	-0.02	0.635	0.55	0.583
Non-cancer illness code, self-reported: ankylosing spondylitis	0.28	0.405	0.07	0.671	0.55	0.584
Non-cancer illness code, self-reported: nephritis	0.18	0.567	-0.01	0.958	0.55	0.585
Treatment/medication code: prednisolone	0.29	0.176	0.43	5.00E-04	-0.55	0.585
Diagnoses - main ICD10: K05 Gingivitis and periodontal diseases	-0.10	0.752	0.09	0.550	-0.55	0.585
Length of working week for main job	0.03	0.818	-0.05	0.496	0.55	0.585
Diagnoses - main ICD10: M23 Internal derangement of knee	-0.08	0.575	0.01	0.919	-0.55	0.585
Diagnoses - main ICD10: M13 Other arthritis	0.07	0.800	0.25	0.166	-0.55	0.586
Non-cancer illness code, self-reported: vertigo	-0.28	0.608	0.04	0.857	-0.54	0.587
Non-cancer illness code, self-reported: trigemminal neuralgia	-0.59	0.700	0.39	0.682	-0.54	0.587
Reason for glasses/contact lenses: For just reading/near work as you are getting older (called 'presbyopia')	0.27	0.239	0.13	0.241	0.54	0.587
Treatment/medication code: crestor 10mg tablet	-0.09	0.742	0.07	0.587	-0.54	0.588
Treatment/medication code: opticrom allergy eye drops	-0.60	0.392	-0.19	0.510	-0.54	0.588
Diagnoses - main ICD10: H71 Cholesteatoma of middle ear	-0.20	0.445	-0.04	0.767	-0.54	0.588
Diagnoses - main ICD10: M62 Other disorders of muscle	0.61	0.461	0.14	0.574	0.54	0.589
Treatment/medication code: reductil 10mg capsule	-0.06	0.822	0.10	0.468	-0.54	0.589
Treatment/medication code: zinc product	-0.42	0.508	-0.04	0.892	-0.54	0.590
Diagnoses - main ICD10: T83 Complications of genito-urinary prosthetic devices, implants and grafts	0.12	0.771	-0.13	0.550	0.54	0.590
Diagnoses - main ICD10: Z00 General examination and investigation of persons without complaint or reported diagnosis	0.01	0.976	-0.22	0.357	0.54	0.591
Creatinine (enzymatic) in urine	0.01	0.892	0.05	0.163	-0.54	0.591
Treatment/medication code: tamsulosin	-0.02	0.957	-0.20	0.222	0.54	0.591
Diagnoses - main ICD10: Z36 Antenatal screening	0.15	0.464	0.03	0.807	0.54	0.592
Treatment/medication code: escitalopram	0.27	0.207	0.14	0.139	0.54	0.592
Treatment/medication code: diclofenac	0.04	0.780	0.12	0.192	-0.54	0.593
Treatment/medication code: fenofibrate	-0.79	0.697	0.52	0.704	-0.54	0.593
Home area population density - urban or rural: Scotland - Remote Rural	0.26	0.123	0.16	0.084	0.53	0.593

Treatment/medication code: duloxetine	0.32	0.652	-0.09	0.767	0.53	0.594
Diagnoses - main ICD10: M15 Polyarthrosis	-0.14	0.658	0.05	0.766	-0.53	0.594
Treatment/medication code: oilatum cream	0.42	0.247	0.20	0.274	0.53	0.595
Diagnoses - main ICD10: H43 Disorders of vitreous body	0.50	0.548	0.03	0.918	0.53	0.595
Non-cancer illness code, self-reported: breast cysts	0.49	0.302	0.21	0.345	0.53	0.595
Diagnoses - main ICD10: D16 Benign neoplasm of bone and articular cartilage	0.01	0.953	-0.11	0.321	0.53	0.596
Non-cancer illness code, self-reported: psoriatic arthropathy	0.51	0.262	0.24	0.247	0.53	0.596
Underlying (primary) cause of death: ICD10: J43.9 Emphysema, unspecified	0.41	0.747	-0.51	0.666	0.53	0.596
Diagnoses - main ICD10: S46 Injury of muscle and tendon at shoulder and upper arm level	-0.06	0.791	0.08	0.511	-0.53	0.598
Eye problems/disorders: Other serious eye condition	0.30	0.359	0.11	0.478	0.53	0.598
Treatment/medication code: diovan 40mg capsule	0.10	0.668	0.24	0.038	-0.53	0.598
Treatment/medication code: epilim 100mg crushable tablet	-0.04	0.882	0.11	0.414	-0.52	0.600
Non-cancer illness code, self-reported: bph / benign prostatic hypertrophy	0.08	0.894	-0.52	0.592	0.52	0.600
Had menopause	-0.05	0.714	0.03	0.654	-0.52	0.601
Medication for pain relief, constipation, heartburn: Ibuprofen (e.g. Nurofen)	0.10	0.273	0.04	0.396	0.52	0.601
Risk taking	-0.03	0.670	0.01	0.759	-0.52	0.601
Illnesses of siblings: High blood pressure	0.15	0.083	0.10	0.032	0.52	0.602
Treatment/medication code: captopril	0.24	0.614	-0.03	0.889	0.52	0.602
Illnesses of mother: Breast cancer	-0.10	0.436	-0.03	0.654	-0.52	0.602
Treatment/medication code: arthrotec 50 tablet	-0.09	0.743	0.08	0.632	-0.52	0.603
Underlying (primary) cause of death: ICD10: J44.9 Chronic obstructive pulmonary disease, unspecified	0.23	0.736	-0.18	0.651	0.52	0.604
Non-cancer illness code, self-reported: haemorrhoids / piles	0.05	0.823	0.19	0.142	-0.52	0.605
Underlying (primary) cause of death: ICD10: C22.1 Intrahepatic bile duct carcinoma	0.52	0.539	0.06	0.802	0.52	0.605
Underlying (primary) cause of death: ICD10: C32.9 Larynx, unspecified	0.04	0.893	0.21	0.190	-0.51	0.608
Non-cancer illness code, self-reported: stroke	0.06	0.754	0.18	0.096	-0.51	0.608
Diagnoses - main ICD10: G43 Migraine	-0.14	0.603	0.01	0.926	-0.51	0.609
Treatment/medication code: ortho-gynest 500micrograms pessary	-0.34	0.656	0.07	0.783	-0.51	0.610
Number of cigarettes previously smoked daily	0.08	0.340	0.03	0.463	0.51	0.610
Treatment/medication code: azopt 10mg/ml eye drops	0.64	0.540	0.09	0.726	0.51	0.610

Underlying (primary) cause of death: ICD10: I42.9 Cardiomyopathy, unspecified	0.22	0.506	0.03	0.827	0.51	0.611
Diagnoses - main ICD10: T84 Complications of internal orthopaedic prosthetic devices, implants and grafts	0.11	0.567	-0.01	0.967	0.51	0.612
Treatment/medication code: paracetamol + codeine	0.84	0.605	0.01	0.976	0.51	0.613
Treatment/medication code: humira 40mg injection solution 0.8ml prefilled syringe	0.12	0.627	0.27	0.036	-0.51	0.613
Frequency of other exercises in last 4 weeks	0.05	0.721	-0.03	0.680	0.50	0.614
Treatment/medication code: flixonase 50micrograms aqueous nasal spray	0.14	0.527	0.02	0.843	0.50	0.614
Cancer code, self-reported: cervical cancer	0.10	0.572	-0.01	0.950	0.50	0.615
Birth weight of first child	-0.01	0.928	-0.05	0.238	0.50	0.617
Treatment/medication code: implanon 68mg subdermal implant	0.50	0.559	0.05	0.841	0.50	0.618
Sitting height	0.04	0.330	0.02	0.398	0.50	0.618
Non-cancer illness code, self-reported: urinary frequency / incontinence	0.01	0.981	0.15	0.306	-0.50	0.618
Underlying (primary) cause of death: ICD10: X70.8 Other specified place	0.07	0.770	-0.07	0.584	0.50	0.618
Diagnoses - main ICD10: J38 Diseases of vocal cords and larynx, not elsewhere classified	0.84	0.215	0.46	0.224	0.50	0.618
Diagnoses - main ICD10: R25 Abnormal involuntary movements	0.21	0.549	0.02	0.932	0.50	0.619
Non-cancer illness code, self-reported: cellulitis	0.08	0.795	-0.09	0.558	0.50	0.619
Underlying (primary) cause of death: ICD10: I62.9 Intracranial haemorrhage (nontraumatic), unspecified	0.10	0.844	-0.19	0.523	0.50	0.620
Treatment/medication code: levocetirizine	-0.07	0.903	0.29	0.526	-0.50	0.620
Diagnoses - main ICD10: K02 Dental caries	0.04	0.888	0.20	0.281	-0.50	0.620
Which eye(s) affected by hypermetropia (long sight): Right eye	-0.02	0.939	0.11	0.378	-0.50	0.620
Alcohol drinker status: Never	0.02	0.886	0.08	0.180	-0.49	0.621
Underlying (primary) cause of death: ICD10: J18.1 Lobar pneumonia, unspecified	-0.52	0.224	-0.28	0.201	-0.49	0.622
Average weekly fortified wine intake	0.05	0.715	-0.03	0.730	0.49	0.622
Diagnoses - main ICD10: L72 Follicular cysts of skin and subcutaneous tissue	-0.17	0.431	-0.05	0.677	-0.49	0.623
Non-cancer illness code, self-reported: tendonitis / tendinitis / tenosynovitis	0.33	0.421	0.11	0.572	0.49	0.623
Treatment/medication code: omega-3/fish oil supplement	0.01	0.957	-0.09	0.312	0.49	0.623
Average weekly intake of other alcoholic drinks	0.16	0.600	0.00	0.983	0.49	0.624
Diagnoses - main ICD10: M70 Soft tissue disorders related to use, overuse and pressure	-0.07	0.707	0.03	0.738	-0.49	0.624
Non-cancer illness code, self-reported: fracture shaft of femur	0.20	0.447	0.06	0.626	0.49	0.626

Diagnoses - main ICD10: G37 Other demyelinating diseases of central nervous system	-0.09	0.697	0.04	0.754	-0.49	0.627
Underlying (primary) cause of death: ICD10: C91.1 Chronic lymphocytic leukaemia	-0.03	0.952	0.28	0.430	-0.49	0.627
Cough on most days	0.12	0.311	0.19	0.004	-0.49	0.627
Had other major operations	0.17	0.116	0.23	6.00E-04	-0.48	0.628
Treatment/medication code: ranitidine	0.18	0.270	0.27	0.004	-0.48	0.628
Diagnoses - main ICD10: R31 Unspecified haematuria	-0.11	0.612	0.01	0.931	-0.48	0.628
Treatment/medication code: fluticasone	0.11	0.707	0.27	0.096	-0.48	0.629
Treatment/medication code: clomipramine	0.01	0.989	-0.21	0.344	0.48	0.630
Treatment/medication code: conjugated oestrogens	-0.06	0.823	0.09	0.529	-0.48	0.630
Surgery on leg arteries (other than for varicose veins)	-0.16	0.648	0.03	0.871	-0.48	0.631
Diagnoses - main ICD10: L27 Dermatitis due to substances taken internally	-0.88	0.648	0.06	0.850	-0.48	0.631
Mouth/teeth dental problems: Loose teeth	0.09	0.412	0.03	0.683	0.48	0.631
Diagnoses - main ICD10: K81 Cholecystitis	0.01	0.968	0.13	0.312	-0.48	0.632
Treatment/medication code: nasobec aqueous 50micrograms nasal spray	0.31	0.675	-0.07	0.810	0.48	0.632
Diagnoses - main ICD10: R79 Other abnormal findings of blood chemistry	0.24	0.320	0.11	0.323	0.47	0.635
Underlying (primary) cause of death: ICD10: M06.9 Rheumatoid arthritis, unspecified	0.86	0.709	-0.31	0.731	0.47	0.636
Medication for cholesterol, blood pressure or diabetes: Blood pressure medication	0.05	0.507	0.09	0.057	-0.47	0.637
Illnesses of siblings: Bowel cancer	-0.16	0.382	-0.06	0.552	-0.47	0.637
Manic/hyper symptoms: I needed less sleep than usual	0.26	0.699	-0.10	0.781	0.47	0.637
Non-cancer illness code, self-reported: encephalitis	0.39	0.308	0.18	0.368	0.47	0.638
Non-cancer illness code, self-reported: mania/bipolar disorder/manic depression	0.17	0.343	0.08	0.402	0.47	0.638
Treatment/medication code: telfast 30 tablet	0.36	0.454	0.10	0.716	0.47	0.638
Treatment/medication code: perindopril	0.33	0.027	0.25	4.00E-04	0.47	0.638
Non-cancer illness code, self-reported: urticaria	-0.33	0.539	-0.06	0.772	-0.47	0.639
Diagnoses - main ICD10: C53 Malignant neoplasm of cervix uteri	-0.69	0.557	-0.13	0.657	-0.47	0.640
Underlying (primary) cause of death: ICD10: F03 Unspecified dementia	-0.27	0.514	-0.05	0.791	-0.47	0.640
Trunk fat-free mass	0.06	0.263	0.08	0.002	-0.47	0.641
Transport type for commuting to job workplace: Car/motor vehicle	0.06	0.595	0.11	0.038	-0.47	0.641
Non-cancer illness code, self-reported: trapped nerve/compressed nerve	-0.36	0.351	-0.16	0.390	-0.47	0.641
Diagnoses - main ICD10: I21 Acute myocardial infarction	0.03	0.793	0.10	0.174	-0.47	0.641

Illnesses of mother: Diabetes	0.06	0.531	0.11	0.020	-0.46	0.642
Mean time to correctly identify matches	-0.02	0.770	0.01	0.656	-0.46	0.642
Non-cancer illness code, self-reported: menorrhagia (unknown cause)	0.17	0.529	0.32	0.074	-0.46	0.642
Average monthly spirits intake	-0.01	0.976	0.09	0.271	-0.46	0.643
Non-cancer illness code, self-reported: ureteric obstruction/hydronephrosis	0.66	0.397	0.26	0.467	0.46	0.643
Trunk predicted mass	0.06	0.261	0.08	0.002	-0.46	0.643
Treatment/medication code: prozac 20mg capsule	-0.26	0.511	-0.03	0.909	-0.46	0.644
Treatment/medication code: co-careldopa	-0.06	0.876	0.14	0.461	-0.46	0.645
Duration of vigorous activity	0.02	0.826	0.07	0.253	-0.46	0.645
Treatment/medication code: mycophenolate	0.21	0.428	0.07	0.643	0.46	0.646
Non-cancer illness code, self-reported: fracture forearm / wrist	-0.32	0.700	0.09	0.795	-0.46	0.649
Non-cancer illness code, self-reported: alcohol dependency	-0.02	0.907	0.07	0.480	-0.45	0.649
Treatment/medication code: flixotide 25micrograms inhaler	0.40	0.324	0.19	0.311	0.45	0.649
Diagnoses - main ICD10: F31 Bipolar affective disorder	0.06	0.737	-0.03	0.738	0.45	0.650
Treatment/medication code: adalate 10mg capsule	-0.07	0.833	0.10	0.574	-0.45	0.650
Treatment/medication code: neoclarityn 5mg tablet	0.17	0.512	0.04	0.690	0.45	0.651
Illnesses of father: Heart disease	0.08	0.334	0.13	0.004	-0.45	0.651
Diagnoses - main ICD10: L60 Nail disorders	-0.18	0.349	-0.08	0.416	-0.45	0.652
Diagnoses - main ICD10: Z52 Donors of organs and tissues	0.09	0.760	0.26	0.212	-0.45	0.652
Non-cancer illness code, self-reported: clotting disorder/excessive bleeding	0.09	0.795	-0.09	0.661	0.45	0.653
Treatment/medication code: salamol 100micrograms inhaler	-0.02	0.944	0.11	0.383	-0.45	0.654
Treatment/medication code: glucosamine product	-0.12	0.352	-0.06	0.359	-0.45	0.654
Treatment/medication code: zirtek 10mg tablet	-0.95	0.680	0.10	0.816	-0.45	0.654
Non-cancer illness code, self-reported: fracture foot	-0.10	0.769	0.07	0.663	-0.45	0.654
Type of tobacco previously smoked: Cigars or pipes	0.02	0.926	0.12	0.286	-0.45	0.655
Mineral and other dietary supplements: Iron	0.21	0.176	0.13	0.116	0.45	0.655
Treatment/medication code: lisinopril+hydrochlorothiazide 10mg/12.5mg tablet	-0.12	0.640	0.01	0.931	-0.45	0.655
Diagnoses - main ICD10: Z53 Persons encountering health services for specifie procedures, not carried out	0.10	0.739	-0.05	0.754	0.44	0.657
Non-cancer illness code, self-reported: heart failure/pulmonary odema	0.24	0.414	0.08	0.720	0.44	0.657

Nap during day	0.05	0.366	0.08	0.002	-0.44	0.657
Underlying (primary) cause of death: ICD10: C22.0 Liver cell carcinoma	-0.37	0.407	-0.15	0.417	-0.44	0.657
Treatment/medication code: enalapril	0.22	0.187	0.13	0.237	0.44	0.660
Diagnoses - main ICD10: D86 Sarcoidosis	-0.57	0.731	0.20	0.718	-0.44	0.660
Treatment/medication code: dixarit 25mcg tablet	-0.49	0.503	-0.14	0.623	-0.44	0.661
Illnesses of siblings: None of the above (group 1)	-0.18	0.024	-0.14	0.002	-0.44	0.661
Non-cancer illness code, self-reported: chickenpox	0.58	0.567	0.11	0.741	0.44	0.661
Morning/evening person (chronotype)	0.03	0.582	0.06	0.085	-0.44	0.661
Treatment/medication code: multivitamins	-0.31	0.438	-0.11	0.565	-0.44	0.662
Diagnoses - main ICD10: S86 Injury of muscle and tendon at lower leg level	0.17	0.593	0.01	0.946	0.44	0.662
Which eye(s) affected by presbyopia: Right eye	-0.26	0.520	-0.07	0.694	-0.44	0.663
Non-cancer illness code, self-reported: other joint disorder	-0.06	0.896	0.17	0.553	-0.44	0.663
Diagnoses - main ICD10: R04 Haemorrhage from respiratory passages	-0.22	0.417	-0.09	0.532	-0.43	0.665
Diagnoses - main ICD10: F43 Reaction to severe stress, and adjustment disorders	0.63	0.363	0.30	0.357	0.43	0.666
Diagnoses - main ICD10: S22 Fracture of rib(s), sternum and thoracic spine	0.12	0.695	-0.03	0.857	0.43	0.666
Diagnoses - main ICD10: C82 Follicular [nodular] non-Hodgkin's lymphoma	0.23	0.689	-0.04	0.875	0.43	0.668
Illness, injury, bereavement, stress in last 2 years: Marital separation/divorce	-0.05	0.781	0.04	0.673	-0.43	0.668
Number of incorrect matches in round	0.04	0.544	0.01	0.757	0.43	0.670
Diagnoses - main ICD10: N82 Fistulae involving female genital tract	-0.20	0.651	0.00	0.985	-0.42	0.671
Treatment/medication code: berocca effervescent tablet	0.19	0.586	0.39	0.202	-0.42	0.672
Pulse wave Arterial Stiffness index	0.07	0.542	0.01	0.826	0.42	0.672
Treatment/medication code: vitamin c product	-0.10	0.749	0.05	0.761	-0.42	0.672
Ever tried to stop smoking	0.22	0.616	0.02	0.940	0.42	0.672
Treatment/medication code: vitamin b compound tablet	0.01	0.966	-0.09	0.450	0.42	0.673
Chest pain or discomfort walking normally	0.17	0.153	0.11	0.085	0.42	0.673
Diagnoses - main ICD10: R20 Disturbances of skin sensation	0.39	0.731	-0.12	0.780	0.42	0.673
Hip pain for 3+ months	0.38	0.569	0.07	0.839	0.42	0.674
Treatment/medication code: spiriva 18micrograms inhalation capsule	0.08	0.610	0.00	0.980	0.42	0.675
Diagnoses - main ICD10: G44 Other headache syndromes	0.04	0.835	-0.06	0.612	0.42	0.677
Treatment/medication code: cyclizine	0.06	0.886	0.30	0.417	-0.42	0.678

Non-cancer illness code, self-reported: retinal artery/vein occlusion	0.90	0.558	0.23	0.603	0.42	0.678
Non-cancer illness code, self-reported: transient ischaemic attack (tia)	0.26	0.316	0.14	0.326	0.41	0.679
Treatment/medication code: salbutamol	0.26	0.085	0.19	0.028	0.41	0.679
Illnesses of mother: Alzheimer's disease/dementia	0.12	0.535	0.03	0.705	0.41	0.679
Treatment/medication code: isotard 25xl m/r tablet	0.34	0.363	0.17	0.311	0.41	0.680
Treatment/medication code: cozaar-comp 50mg/12.5mg tablet	0.03	0.901	0.15	0.278	-0.41	0.681
Underlying (primary) cause of death: ICD10: C90.0 Multiple myeloma	-0.61	0.397	-0.29	0.317	-0.41	0.681
Pace-maker	0.32	0.353	0.16	0.308	0.41	0.682
Illnesses of siblings: Severe depression	0.11	0.310	0.17	0.004	-0.41	0.682
Neck/shoulder pain for 3+ months	0.03	0.881	0.11	0.326	-0.41	0.683
Treatment/medication code: adipine mr 10 m/r tablet	-0.67	0.657	-0.04	0.884	-0.41	0.683
Treatment/medication code: imdur 60mg durule	-0.02	0.963	-0.21	0.441	0.41	0.684
Hearing difficulty/problems: Yes	0.07	0.350	0.10	0.002	-0.41	0.685
Diagnoses - main ICD10: I70 Atherosclerosis	-0.25	0.563	-0.06	0.756	-0.41	0.685
Vitamin and mineral supplements: Multivitamins +/- minerals	0.06	0.493	0.02	0.651	0.41	0.685
Diagnoses - main ICD10: I25 Chronic ischaemic heart disease	0.13	0.156	0.17	4.00E-04	-0.41	0.685
Heel bone mineral density (BMD) T-score, automated (left)	0.01	0.861	-0.02	0.662	0.40	0.686
Diagnoses - main ICD10: M16 Coxarthrosis [arthrosis of hip]	-0.14	0.288	-0.08	0.358	-0.40	0.686
Diagnoses - main ICD10: F60 Specific personality disorders	-0.08	0.722	-0.18	0.141	0.40	0.686
Diagnoses - main ICD10: T79 Certain early complications of trauma, not elsewhere classified	0.49	0.405	0.23	0.451	0.40	0.687
Diagnoses - main ICD10: C20 Malignant neoplasm of rectum	0.24	0.762	-0.14	0.783	0.40	0.687
Diagnoses - main ICD10: H00 Hordeolum and chalazion	0.43	0.732	-0.09	0.803	0.40	0.689
Treatment/medication code: prochlorperazine	-0.48	0.708	0.06	0.888	-0.40	0.689
Diagnoses - main ICD10: L05 Pilonidal cyst	0.05	0.862	-0.08	0.594	0.40	0.690
Treatment/medication code: amlodipine	0.13	0.176	0.09	0.110	0.40	0.690
Non-cancer illness code, self-reported: eye/eyelid problem	0.34	0.378	0.17	0.420	0.40	0.690
Treatment/medication code: evening primrose oil	0.24	0.324	0.13	0.329	0.40	0.690
Underlying (primary) cause of death: ICD10: G90.3 Multisystem degeneration	0.29	0.308	0.16	0.295	0.40	0.691
Non-cancer illness code, self-reported: polycythaemia vera	0.08	0.781	-0.05	0.752	0.40	0.691

Diagnoses - main ICD10: E07 Other disorders of thyroid	0.50	0.330	0.27	0.342	0.40	0.692
Treatment/medication code: dicloflex 25mg e/c tablet	-0.31	0.737	0.13	0.833	-0.40	0.692
Treatment/medication code: calcichew 1.25g chewable tablet	-0.12	0.815	0.11	0.699	-0.40	0.692
Illnesses of siblings: Heart disease	0.27	0.008	0.22	8.44E-06	0.40	0.693
Treatment/medication code: pregabalin	0.27	0.388	0.13	0.420	0.39	0.693
Diagnoses - main ICD10: E03 Other hypothyroidism	0.31	0.542	0.64	0.318	-0.39	0.693
Treatment/medication code: arcoxia 90mg tablet	0.27	0.787	-0.30	0.776	0.39	0.694
Ever had breast cancer screening / mammogram	0.09	0.592	0.02	0.842	0.39	0.694
Types of transport used (excluding work): Public transport	0.08	0.394	0.04	0.524	0.39	0.695
Leg pain when standing still or sitting	0.19	0.516	0.06	0.679	0.39	0.695
Cancer code, self-reported: unclassifiable	0.00	0.998	0.14	0.440	-0.39	0.696
Treatment/medication code: hepacon b12 1mg/1ml injection	0.24	0.646	0.54	0.333	-0.39	0.696
Alcohol intake versus 10 years previously	0.06	0.423	0.09	0.011	-0.39	0.696
Time spent driving	0.04	0.650	0.00	0.992	0.39	0.696
Non-cancer illness code, self-reported: chronic fatigue syndrome	-0.03	0.900	0.07	0.471	-0.39	0.696
Reason former drinker stopped drinking alcohol: Financial reasons	-0.96	0.609	-0.20	0.689	-0.39	0.697
Diagnoses - main ICD10: K20 Oesophagitis	-0.05	0.855	0.06	0.608	-0.39	0.697
Non-cancer illness code, self-reported: allergy or anaphylactic reaction to drug	0.47	0.229	0.30	0.175	0.39	0.697
Underlying (primary) cause of death: ICD10: K70.1 Alcoholic hepatitis	-0.21	0.632	-0.02	0.903	-0.39	0.698
Diagnoses - main ICD10: H57 Other disorders of eye and adnexa	0.03	0.922	-0.11	0.500	0.39	0.698
Reason for glasses/contact lenses: For 'astigmatism'	-0.02	0.859	0.03	0.591	-0.39	0.698
Non-cancer illness code, self-reported: gastro-oesophageal reflux (gord) / gastric reflux	0.12	0.290	0.07	0.231	0.39	0.699
Treatment/medication code: vitamin b12 preparation	0.15	0.483	0.25	0.076	-0.39	0.699
Treatment/medication code: cialis 20mg tablet	-0.20	0.606	-0.03	0.886	-0.39	0.700
Manic/hyper symptoms: I was more creative or had more ideas than usual	0.17	0.668	0.37	0.223	-0.39	0.700
Duration of other exercises	0.03	0.826	-0.03	0.667	0.38	0.701
Non-cancer illness code, self-reported: heart valve problem/heart murmur	0.35	0.449	0.15	0.493	0.38	0.701
Treatment/medication code: vagifem 25mcg pessary	0.44	0.596	0.10	0.709	0.38	0.701
Diagnoses - main ICD10: M80 Osteoporosis with pathological fracture	0.03	0.948	0.27	0.528	-0.38	0.703
Diagnoses - main ICD10: H33 Retinal detachments and breaks	0.08	0.621	0.01	0.891	0.38	0.703

Treatment/medication code: volsaid retard 75mg m/r tablet	0.02	0.952	0.12	0.334	-0.38	0.703
Father's age at death	-0.06	0.536	-0.11	0.121	0.38	0.703
Diagnoses - main ICD10: D46 Myelodysplastic syndromes	0.13	0.810	-0.10	0.724	0.38	0.703
Treatment/medication code: livial 2.5mg tablet	0.18	0.557	0.05	0.803	0.38	0.705
Treatment/medication code: rosuvastatin	0.19	0.412	0.09	0.361	0.38	0.705
Treatment/medication code: adcal-d3 1.5g/10micrograms chewable tablet	0.25	0.125	0.18	0.055	0.38	0.705
Treatment/medication code: creon e/c granules in capsule	-0.08	0.905	0.27	0.682	-0.38	0.706
Treatment/medication code: hypromellose	-0.14	0.853	0.66	0.741	-0.38	0.707
Treatment/medication code: eumovate cream	0.32	0.271	0.20	0.237	0.37	0.708
Underlying (primary) cause of death: ICD10: C73 Malignant neoplasm of thyroid gland	-0.08	0.752	-0.18	0.114	0.37	0.708
Non-cancer illness code, self-reported: disc problem	-0.14	0.797	-0.45	0.441	0.37	0.708
Treatment/medication code: movelat cream	-0.15	0.693	0.01	0.954	-0.37	0.708
Diagnoses - main ICD10: R23 Other skin changes	-0.05	0.830	0.04	0.673	-0.37	0.708
Stomach/abdominal pain for 3+ months	-0.06	0.816	0.04	0.717	-0.37	0.710
Duration walking for pleasure	-0.09	0.389	-0.05	0.289	-0.37	0.711
Underlying (primary) cause of death: ICD10: C25.9 Pancreas, unspecified	-0.08	0.782	0.04	0.786	-0.37	0.712
Diagnoses - main ICD10: D37 Neoplasm of uncertain or unknown behaviour of oral cavity and digestive organs	-0.16	0.862	0.39	0.738	-0.37	0.712
Treatment/medication code: metoclopramide	0.12	0.579	0.03	0.834	0.37	0.713
Longest period of unenthusiasm / disinterest	0.12	0.682	0.00	0.995	0.37	0.713
Treatment/medication code: sodium thyroxine	0.06	0.927	0.83	0.679	-0.37	0.714
Treatment/medication code: magnesium citrate	0.11	0.727	-0.02	0.906	0.37	0.714
Non-cancer illness code, self-reported: thyroid goitre	-0.04	0.880	-0.15	0.347	0.37	0.715
Diagnoses - main ICD10: N35 Urethral stricture	0.08	0.738	0.19	0.172	-0.36	0.716
Non-cancer illness code, self-reported: gall bladder disease	-0.13	0.604	-0.03	0.805	-0.36	0.716
Cancer code, self-reported: liver/hepatocellular cancer	0.22	0.445	0.11	0.463	0.36	0.719
Diagnoses - main ICD10: T39 Poisoning by nonopioid analgesics, antipyretics and antirheumatics	0.22	0.497	0.09	0.527	0.36	0.719
Impedance of leg (right)	-0.05	0.303	-0.07	0.041	0.36	0.719
Treatment/medication code: ismo - isosorbide mononitrate	-0.02	0.961	-0.21	0.483	0.36	0.720
Treatment/medication code: solifenacin	0.11	0.618	0.02	0.892	0.36	0.720

Underlying (primary) cause of death: ICD10: I51.7 Cardiomegaly	0.26	0.390	0.14	0.366	0.36	0.722
Underlying (primary) cause of death: ICD10: X42.0 Home	-0.04	0.907	0.10	0.572	-0.36	0.722
Types of physical activity in last 4 weeks: Heavy DIY (eg: weeding, lawn mowing, carpentry, digging)	-0.01	0.869	-0.04	0.279	0.36	0.722
Forced expiratory volume in 1-second (FEV1), predicted	0.03	0.610	0.06	0.065	-0.36	0.723
Diagnoses - main ICD10: D47 Other neoplasms of uncertain or unknown behaviour of lymphoid, haematopoietic and related tissue	0.09	0.777	-0.03	0.808	0.35	0.723
Wants to stop smoking	-0.03	0.897	0.07	0.641	-0.35	0.723
Treatment/medication code: phenobarbitone	-0.11	0.860	0.14	0.685	-0.35	0.724
Treatment/medication code: dothiepin	-0.38	0.343	-0.23	0.225	-0.35	0.724
Diagnoses - main ICD10: G35 Multiple sclerosis	-0.08	0.898	0.21	0.698	-0.35	0.726
Non-cancer illness code, self-reported: anorexia/bulimia/other eating disorder	-0.31	0.583	-0.10	0.689	-0.35	0.726
Treatment/medication code: salbutamol 100micrograms spacehaler	0.28	0.088	0.21	0.097	0.35	0.727
Eye problems/disorders: Injury or trauma resulting in loss of vision	-0.40	0.600	-0.11	0.717	-0.35	0.727
Treatment/medication code: lofepramine	-0.06	0.921	-0.39	0.595	0.35	0.727
Treatment/medication code: temazepam	0.18	0.500	0.07	0.626	0.35	0.727
Non-cancer illness code, self-reported: burns	0.00	0.991	0.16	0.501	-0.35	0.728
Eye problems/disorders: None of the above	-0.06	0.609	-0.01	0.827	-0.35	0.728
Diagnoses - main ICD10: O20 Haemorrhage in early pregnancy	0.23	0.657	0.04	0.865	0.34	0.731
Treatment/medication code: zomig 2.5mg tablet	0.04	0.835	0.12	0.244	-0.34	0.731
Treatment/medication code: travoprost	-0.24	0.647	-0.04	0.862	-0.34	0.731
Diagnoses - main ICD10: H72 Perforation of tympanic membrane	0.09	0.885	0.48	0.615	-0.34	0.733
Treatment/medication code: prednisone	0.29	0.615	0.07	0.808	0.34	0.733
Treatment/medication code: carbomers	0.04	0.913	0.17	0.396	-0.34	0.734
Diagnoses - main ICD10: D17 Benign lipomatous neoplasm	-0.17	0.458	-0.09	0.431	-0.34	0.734
Diagnoses - main ICD10: O23 Infections of genito-urinary tract in pregnancy	0.05	0.904	-0.11	0.601	0.34	0.735
Treatment/medication code: oxybutynin	-0.10	0.661	-0.01	0.900	-0.34	0.735
Pulse wave peak to peak time	-0.05	0.657	-0.01	0.936	-0.34	0.735
Treatment/medication code: dihydrocodeine	0.23	0.263	0.15	0.213	0.34	0.735
Non-cancer illness code, self-reported: malaria	0.39	0.544	0.15	0.602	0.34	0.735
Mouth/teeth dental problems: Toothache	0.22	0.092	0.17	0.013	0.34	0.736

Diagnoses - main ICD10: J84 Other interstitial pulmonary diseases	0.43	0.356	0.25	0.373	0.34	0.737
Non-cancer illness code, self-reported: peptic ulcer	0.17	0.682	0.02	0.906	0.34	0.737
Treatment/medication code: evorel 25 patch	0.11	0.728	-0.01	0.956	0.34	0.737
Cancer code, self-reported: bladder cancer	0.09	0.898	-0.38	0.754	0.33	0.738
Diagnoses - main ICD10: L30 Other dermatitis	-0.05	0.898	0.10	0.641	-0.33	0.739
Underlying (primary) cause of death: ICD10: K74.6 Other and unspecified cirrhosis of liver	-0.25	0.772	0.05	0.866	-0.33	0.741
Tobacco smoking: Ex-smoker	0.07	0.504	0.03	0.638	0.33	0.741
Treatment/medication code: premarin 625micrograms tablet	-0.12	0.699	-0.25	0.169	0.33	0.742
Bring up phlegm/sputum/mucus on most days	0.05	0.735	0.11	0.123	-0.33	0.742
Treatment/medication code: st john's wort/hypericum [ctsu]	-0.06	0.908	0.71	0.756	-0.33	0.743
Tinnitus severity/nuisance	0.22	0.419	0.12	0.393	0.33	0.743
Treatment/medication code: tranexamic acid	0.39	0.763	-0.06	0.895	0.33	0.743
Treatment/medication code: arcoxia 60mg tablet	-0.12	0.687	-0.01	0.931	-0.33	0.743
Diagnoses - main ICD10: I22 Subsequent myocardial infarction	0.15	0.686	0.31	0.256	-0.33	0.743
Treatment/medication code: bendroflumethiazide	0.03	0.789	0.06	0.374	-0.33	0.744
Non-cancer illness code, self-reported: erectile dysfunction / impotence	-0.04	0.946	0.20	0.685	-0.33	0.744
Comparative height size at age 10	0.04	0.378	0.03	0.289	0.33	0.744
Non-cancer illness code, self-reported: type 2 diabetes	0.09	0.584	0.15	0.098	-0.33	0.745
Treatment/medication code: alfacalcidol	0.57	0.332	0.34	0.335	0.33	0.745
Getting up in morning	-0.08	0.235	-0.10	5.00E-04	0.33	0.745
Treatment/medication code: erythromycin	0.35	0.300	0.23	0.253	0.32	0.746
Non-cancer illness code, self-reported: dupuytren's contracture	-0.01	0.967	0.09	0.542	-0.32	0.747
Non-cancer illness code, self-reported: cervical erosion	0.74	0.710	0.09	0.769	0.32	0.748
Treatment/medication code: omeprazole	0.16	0.111	0.20	1.00E-04	-0.32	0.749
Treatment/medication code: premique 0.625mg/5mg tablet	-0.03	0.921	-0.13	0.358	0.32	0.749
Breastfed as a baby	-0.06	0.496	-0.03	0.573	-0.32	0.750
Tobacco smoking: Smokes on most or all days	0.11	0.664	0.02	0.875	0.32	0.750
Treatment/medication code: testogel 50mg gel 5g sachet	0.03	0.868	0.10	0.257	-0.32	0.750
Prospective memory result	0.00	0.975	0.04	0.399	-0.32	0.751

Diagnoses - main ICD10: F45 Somatoform disorders	0.24	0.497	0.12	0.473	0.32	0.752
Treatment/medication code: clarithromycin	0.22	0.693	0.01	0.970	0.32	0.752
Diagnoses - main ICD10: M79 Other soft tissue disorders, not elsewhere classified	0.14	0.496	0.07	0.579	0.31	0.753
Treatment/medication code: xalatan 0.005% eye drops	-0.08	0.539	-0.03	0.622	-0.31	0.753
Vitamin and mineral supplements: Vitamin D	0.08	0.592	0.13	0.106	-0.31	0.754
Treatment/medication code: slozem 120mg m/r capsule	0.26	0.497	0.41	0.146	-0.31	0.755
Illnesses of father: None of the above (group 2)	0.06	0.635	0.11	0.196	-0.31	0.755
Treatment/medication code: estraderm mx 25 patch	0.04	0.919	0.19	0.422	-0.31	0.755
Illnesses of siblings: Lung cancer	-0.16	0.537	-0.06	0.740	-0.31	0.756
Average monthly intake of other alcoholic drinks	0.14	0.592	0.05	0.711	0.31	0.756
Diagnoses - main ICD10: O02 Other abnormal products of conception	-0.13	0.676	-0.25	0.209	0.31	0.758
Diagnoses - main ICD10: K14 Diseases of tongue	0.10	0.727	0.19	0.216	-0.31	0.759
Diagnoses - main ICD10: Z08 Follow-up examination after treatment for malignant neoplasm	0.35	0.809	-0.19	0.851	0.31	0.760
Non-cancer illness code, self-reported: endometriosis	0.02	0.947	-0.06	0.547	0.31	0.760
Job involves shift work	0.05	0.588	0.08	0.048	-0.30	0.761
Treatment/medication code: fybogel mebeverine sachet	-0.01	0.973	-0.15	0.499	0.30	0.762
Non-cancer illness code, self-reported: hiatus hernia	0.10	0.409	0.15	0.032	-0.30	0.763
Average monthly beer plus cider intake	-0.01	0.953	0.06	0.586	-0.30	0.767
Type of tobacco previously smoked: Hand-rolled cigarettes	0.09	0.697	0.16	0.155	-0.30	0.768
Diagnoses - main ICD10: H34 Retinal vascular occlusions	0.28	0.567	0.12	0.582	0.30	0.768
Reason for glasses/contact lenses: For a 'lazy' eye or an eye with poor vision since childhood (called 'amblyopia')	0.07	0.693	0.13	0.143	-0.30	0.768
Treatment/medication code: azathioprine	0.21	0.688	0.48	0.515	-0.29	0.768
Illnesses of father: High blood pressure	0.07	0.439	0.11	0.053	-0.29	0.769
Non-cancer illness code, self-reported: pneumothorax	0.06	0.813	-0.02	0.854	0.29	0.770
Other eye problems	0.28	0.042	0.24	0.001	0.29	0.770
Treatment/medication code: carbamazepine	0.06	0.911	0.32	0.659	-0.29	0.771
Treatment/medication code: irbesartan	0.09	0.684	0.17	0.144	-0.29	0.771
Treatment/medication code: symbicort 100/6 turbohaler	0.03	0.874	0.10	0.380	-0.29	0.772
Diagnoses - main ICD10: I24 Other acute ischaemic heart diseases	0.09	0.754	0.18	0.211	-0.29	0.773

Treatment/medication code: epaderm ointment	0.01	0.985	0.10	0.453	-0.29	0.773
Non-cancer illness code, self-reported: vitiligo	0.30	0.317	0.41	0.079	-0.29	0.775
Illnesses of father: Lung cancer	0.05	0.648	0.01	0.822	0.29	0.775
Diagnoses - main ICD10: J01 Acute sinusitis	0.38	0.602	0.16	0.562	0.29	0.775
Eye problems/disorders: Diabetes related eye disease	0.20	0.190	0.25	0.003	-0.29	0.775
Number of older siblings	0.18	0.498	0.09	0.579	0.28	0.776
Treatment/medication code: trazodone	0.05	0.897	-0.08	0.739	0.28	0.777
Diagnoses - main ICD10: L08 Other local infections of skin and subcutaneous tissue	-0.09	0.687	-0.02	0.876	-0.28	0.777
Diagnoses - main ICD10: M84 Disorders of continuity of bone	0.15	0.512	0.22	0.054	-0.28	0.777
Current employment status: In paid employment or self-employed	-0.06	0.676	-0.10	0.084	0.28	0.777
Treatment/medication code: gliclazide	0.11	0.367	0.15	0.025	-0.28	0.779
Diagnoses - main ICD10: J22 Unspecified acute lower respiratory infection	0.11	0.605	0.04	0.774	0.28	0.779
Non-cancer illness code, self-reported: osteopenia	0.05	0.918	-0.10	0.717	0.28	0.780
Non-cancer illness code, self-reported: fracture neck of femur / hip	0.17	0.713	0.35	0.400	-0.28	0.780
Diagnoses - main ICD10: D45 Polycythaemia vera	-0.17	0.527	-0.08	0.628	-0.28	0.780
Diagnoses - main ICD10: E66 Obesity	0.19	0.366	0.13	0.171	0.28	0.780
Diagnoses - main ICD10: O90 Complications of the puerperium, not elsewhere classified	0.56	0.567	0.26	0.590	0.28	0.781
Medication for pain relief, constipation, heartburn: Aspirin	0.13	0.190	0.16	0.009	-0.28	0.781
Diagnoses - main ICD10: M60 Myositis	0.50	0.533	0.25	0.516	0.28	0.782
Comparative body size at age 10	0.06	0.237	0.04	0.211	0.28	0.782
Treatment/medication code: salbutamol+ipratropium 100micrograms/20micrograms inhaler	0.47	0.665	0.15	0.744	0.28	0.783
Work/job satisfaction	-0.04	0.704	-0.01	0.868	-0.27	0.784
Treatment/medication code: microgynon 30 tablet	0.07	0.843	0.19	0.331	-0.27	0.784
Treatment/medication code: angitil sr 90 m/r capsule	-0.01	0.968	0.07	0.583	-0.27	0.785
Diagnoses - main ICD10: O80 Single spontaneous delivery	-0.15	0.824	-0.54	0.670	0.27	0.786
Underlying (primary) cause of death: ICD10: K70.3 Alcoholic cirrhosis of liver	-0.08	0.801	0.01	0.922	-0.27	0.787
Treatment/medication code: protelos 2g sachets	-0.07	0.767	-0.14	0.253	0.27	0.787
Treatment/medication code: lumigan 0.3mg/ml eye drops	-0.20	0.491	-0.11	0.295	-0.27	0.787
Frequency of light DIY in last 4 weeks	0.05	0.654	0.02	0.766	0.27	0.788

Diagnoses - main ICD10: T86 Failure and rejection of transplanted organs and tissues	-0.11	0.625	-0.04	0.763	-0.27	0.788
Treatment/medication code: carvedilol	-0.10	0.742	-0.01	0.944	-0.27	0.788
Diagnoses - main ICD10: K43 Ventral hernia	0.16	0.321	0.11	0.133	0.27	0.789
Treatment/medication code: dermol 500 lotion	0.25	0.749	0.49	0.226	-0.27	0.789
Diagnoses - main ICD10: H91 Other hearing loss	0.00	0.996	-0.14	0.574	0.27	0.790
Diagnoses - main ICD10: I05 Rheumatic mitral valve diseases	0.29	0.690	0.08	0.793	0.27	0.790
Treatment/medication code: calcium carbonate+cholecalciferol 1.25g/200iu tablet	0.31	0.407	0.44	0.115	-0.27	0.791
Treatment/medication code: clopidogrel	0.11	0.540	0.17	0.087	-0.26	0.791
Treatment/medication code: securon 40mg tablet	0.23	0.548	0.11	0.620	0.26	0.791
Illnesses of father: None of the above (group 1)	-0.13	0.228	-0.17	0.023	0.26	0.792
Treatment/medication code: quetiapine	-0.06	0.841	-0.15	0.374	0.26	0.792
Diagnoses - main ICD10: N60 Benign mammary dysplasia	0.06	0.739	0.11	0.216	-0.26	0.793
Diagnoses - main ICD10: S42 Fracture of shoulder and upper arm	-0.19	0.677	-0.06	0.793	-0.26	0.793
Non-cancer illness code, self-reported: polymyalgia rheumatica	0.15	0.358	0.19	0.024	-0.26	0.793
Treatment/medication code: asacol 400mg e/c tablet	-0.02	0.937	0.05	0.647	-0.26	0.794
Diagnoses - main ICD10: I49 Other cardiac arrhythmias	-0.56	0.827	0.13	0.845	-0.26	0.794
Treatment/medication code: mercilon tablet	0.33	0.360	0.23	0.215	0.26	0.794
Treatment/medication code: losartan	0.12	0.349	0.08	0.507	0.26	0.795
Diagnoses - main ICD10: C34 Malignant neoplasm of bronchus and lung	0.20	0.550	0.11	0.506	0.26	0.795
Treatment/medication code: quinine	0.14	0.393	0.09	0.352	0.26	0.796
Underlying (primary) cause of death: ICD10: C92.0 Acute myeloid leukaemia	-0.21	0.607	-0.09	0.624	-0.25	0.799
Treatment/medication code: calcichew d3 tablet	0.21	0.199	0.25	0.008	-0.25	0.800
Non-cancer illness code, self-reported: cervical spondylosis	0.19	0.304	0.13	0.154	0.25	0.801
Diagnoses - main ICD10: J39 Other diseases of upper respiratory tract	0.12	0.674	0.20	0.251	-0.25	0.801
Number of self-reported cancers	-0.09	0.582	-0.13	0.105	0.25	0.802
Cancer code, self-reported: sarcoma/fibrosarcoma	-0.36	0.361	-0.24	0.308	-0.25	0.803
Underlying (primary) cause of death: ICD10: G20 Parkinson's disease	-0.60	0.635	-0.26	0.649	-0.25	0.803
Diagnoses - main ICD10: L98 Other disorders of skin and subcutaneous tissue, not elsewhere classified	-0.11	0.545	-0.06	0.522	-0.25	0.804
Underlying (primary) cause of death: ICD10: C16.0 Cardia	0.06	0.800	0.13	0.261	-0.25	0.805

Diagnoses - main ICD10: J36 Peritonsillar abscess	-0.75	0.631	-0.34	0.572	-0.24	0.806
Non-cancer illness code, self-reported: vaginal prolapse/uterine prolapse	-0.05	0.746	-0.01	0.944	-0.24	0.808
Leg pain on walking : effect of standing still	-0.19	0.474	-0.12	0.440	-0.24	0.810
Treatment/medication code: hydroxocobalamin	0.17	0.603	0.08	0.592	0.24	0.810
Pain type(s) experienced in last month: Headache	0.11	0.107	0.09	0.016	0.24	0.810
Smoking status: Current	0.03	0.686	0.05	0.287	-0.24	0.811
Diagnoses - main ICD10: I12 Hypertensive renal disease	0.01	0.980	0.08	0.536	-0.24	0.811
Underlying (primary) cause of death: ICD10: C45.0 Mesothelioma of pleura	-0.21	0.447	-0.13	0.391	-0.24	0.811
Non-cancer illness code, self-reported: headaches (not migraine)	0.11	0.713	0.03	0.825	0.24	0.812
Diagnoses - main ICD10: R42 Dizziness and giddiness	0.45	0.216	0.35	0.093	0.24	0.812
Medication for pain relief, constipation, heartburn: Paracetamol	0.14	0.050	0.16	7.60E-06	-0.24	0.812
Why reduced smoking: Health precaution	-0.08	0.726	-0.15	0.217	0.24	0.813
Diagnoses - main ICD10: C69 Malignant neoplasm of eye and adnexa	0.18	0.604	0.28	0.233	-0.24	0.813
Impedance of leg (left)	-0.05	0.308	-0.06	0.070	0.24	0.813
Why stopped smoking: None of the above	0.03	0.809	0.06	0.301	-0.24	0.814
Loud music exposure frequency	0.10	0.424	0.13	0.018	-0.23	0.814
Non-cancer illness code, self-reported: sleep apnoea	0.23	0.263	0.28	0.013	-0.23	0.815
Diagnoses - main ICD10: G93 Other disorders of brain	0.06	0.937	0.31	0.697	-0.23	0.816
Treatment/medication code: clonazepam	-0.12	0.838	0.03	0.910	-0.23	0.816
Treatment/medication code: co-enzyme q10/ubiquinone/bio-quinone/coenzyme q10	-0.05	0.937	-0.28	0.687	0.23	0.816
Diagnoses - main ICD10: L91 Hypertrophic disorders of skin	0.01	0.972	0.10	0.564	-0.23	0.816
Current employment status: Looking after home and/or family	0.07	0.665	0.03	0.697	0.23	0.817
Treatment/medication code: aqueous cream bp	-0.02	0.936	-0.09	0.537	0.23	0.819
Treatment/medication code: nitromin 400micrograms cfc-free spray	0.10	0.784	0.20	0.405	-0.23	0.820
Time spent using computer	0.02	0.796	0.00	0.972	0.23	0.820
Weight change compared with 1 year ago	0.14	0.273	0.17	0.005	-0.23	0.820
Non-cancer illness code, self-reported: cardiomyopathy	0.27	0.380	0.19	0.183	0.23	0.821
Diagnoses - main ICD10: Z51 Other medical care	-0.01	0.978	0.06	0.664	-0.22	0.822
Non-cancer illness code, self-reported: meniere's disease	0.19	0.447	0.25	0.115	-0.22	0.823
Treatment/medication code: macrogol ointment	0.15	0.741	0.28	0.397	-0.22	0.824

Treatment/medication code: metronidazole	0.16	0.734	0.31	0.503	-0.22	0.824
Non-cancer illness code, self-reported: bursitis	-0.26	0.632	-0.09	0.871	-0.22	0.825
Diagnoses - main ICD10: M45 Ankylosing spondylitis	0.05	0.844	-0.02	0.920	0.22	0.826
Underlying (primary) cause of death: ICD10: I50.9 Heart failure, unspecified	0.37	0.640	0.17	0.667	0.22	0.826
Treatment/medication code: digoxin	0.12	0.586	0.07	0.539	0.22	0.826
Treatment/medication code: brinzolamide	-0.18	0.676	-0.08	0.643	-0.22	0.827
Diagnoses - main ICD10: S20 Superficial injury of thorax	0.15	0.588	0.22	0.150	-0.22	0.828
Treatment/medication code: detrusitol 1mg tablet	0.10	0.692	0.04	0.762	0.22	0.828
Treatment/medication code: metformin	0.11	0.195	0.13	0.005	-0.22	0.829
Diagnoses - main ICD10: R86 Abnormal findings in specimens from male genital organs	0.48	0.769	0.12	0.787	0.21	0.831
Wears glasses or contact lenses	0.04	0.725	0.01	0.783	0.21	0.831
Diagnoses - main ICD10: N75 Diseases of Bartholin's gland	-0.08	0.721	-0.13	0.274	0.21	0.831
Non-cancer illness code, self-reported: peripheral vascular disease	-0.04	0.826	0.00	0.977	-0.21	0.833
Doctor diagnosed hayfever or allergic rhinitis	-0.01	0.881	0.01	0.870	-0.21	0.834
Illnesses of siblings: Chronic bronchitis/emphysema	0.19	0.229	0.23	0.009	-0.21	0.834
Heel bone mineral density (BMD) T-score, automated (right)	0.00	0.991	-0.02	0.739	0.21	0.834
Happiness	0.10	0.259	0.08	0.068	0.21	0.834
Underlying (primary) cause of death: ICD10: C53.9 Cervix uteri, unspecified	-0.55	0.880	0.27	0.852	-0.21	0.835
Treatment/medication code: nurofen 200mg tablet	0.22	0.824	0.00	0.997	0.21	0.835
Diagnoses - main ICD10: N86 Erosion and ectropion of cervix uteri	0.05	0.823	0.00	0.987	0.21	0.835
Average monthly red wine intake	-0.06	0.744	-0.02	0.834	-0.21	0.835
Cancer code, self-reported: small intestine/small bowel cancer	0.10	0.838	-0.01	0.959	0.20	0.838
Treatment/medication code: pioglitazone	0.21	0.254	0.17	0.052	0.20	0.838
Non-cancer illness code, self-reported: renal failure not requiring dialysis	0.78	0.759	0.25	0.749	0.20	0.840
Tinnitus: Yes, now some of the time	0.06	0.785	0.11	0.299	-0.20	0.841
Diagnoses - main ICD10: N18 Chronic renal failure	0.23	0.835	0.90	0.776	-0.20	0.841
Non-cancer illness code, self-reported: bronchitis	0.05	0.902	0.13	0.460	-0.20	0.843
Diagnoses - main ICD10: R51 Headache	0.01	0.982	0.06	0.674	-0.20	0.843
Treatment/medication code: progynova 1mg tablet	-0.20	0.412	-0.14	0.269	-0.20	0.843

Diagnoses - main ICD10: R47 Speech disturbances, not elsewhere classified	-0.19	0.661	-0.31	0.408	0.20	0.844
Fractured bone site(s): Wrist	0.11	0.441	0.15	0.071	-0.20	0.844
Diagnoses - main ICD10: J03 Acute tonsillitis	0.02	0.925	-0.03	0.816	0.20	0.845
Treatment/medication code: coversyl plus 4mg/1.25mg tablet	-0.12	0.724	-0.05	0.791	-0.19	0.847
Diagnoses - main ICD10: K61 Abscess of anal and rectal regions	0.25	0.417	0.19	0.207	0.19	0.847
Treatment/medication code: eprosartan	-0.20	0.729	-0.08	0.766	-0.19	0.848
Vascular/heart problems diagnosed by doctor: High blood pressure	0.10	0.090	0.09	0.031	0.19	0.848
Treatment/medication code: viazem xl 120mg m/r capsule	0.01	0.961	-0.04	0.722	0.19	0.849
Diagnoses - main ICD10: K63 Other diseases of intestine	-0.03	0.910	0.03	0.849	-0.19	0.851
Diagnoses - main ICD10: C44 Other malignant neoplasms of skin	-0.21	0.096	-0.24	2.00E-04	0.19	0.851
Diagnoses - main ICD10: S66 Injury of muscle and tendon at wrist and hand level	0.12	0.590	0.07	0.514	0.19	0.851
Pain type(s) experienced in last month: None of the above	-0.13	0.024	-0.14	5.19E-06	0.19	0.851
Treatment/medication code: hydroxocobalamin product	0.05	0.850	0.00	0.972	0.19	0.852
Diagnoses - main ICD10: K52 Other non-infective gastro-enteritis and colitis	0.24	0.162	0.20	0.024	0.19	0.853
Diagnoses - main ICD10: K01 Embedded and impacted teeth	0.10	0.708	0.04	0.782	0.19	0.853
Treatment/medication code: anadin tablet	0.03	0.925	-0.03	0.831	0.19	0.853
Diagnoses - main ICD10: I48 Atrial fibrillation and flutter	0.01	0.899	0.04	0.479	-0.19	0.853
Diagnoses - main ICD10: K91 Postprocedural disorders of digestive system, not elsewhere classified	-0.07	0.720	-0.03	0.736	-0.18	0.855
Treatment/medication code: zolmitriptan	-0.09	0.731	-0.04	0.787	-0.18	0.856
Diagnoses - main ICD10: H60 Otitis externa	0.19	0.600	0.27	0.227	-0.18	0.856
Treatment/medication code: aspirin	0.15	0.131	0.17	0.005	-0.18	0.856
Ever had hysterectomy (womb removed)	-0.11	0.399	-0.09	0.275	-0.18	0.858
Non-cancer illness code, self-reported: raynaud's phenomenon/disease	-0.10	0.680	-0.05	0.716	-0.17	0.861
Treatment/medication code: ramipril	0.13	0.250	0.11	0.103	0.17	0.862
Non-cancer illness code, self-reported: peripheral nerve disorder	-0.07	0.846	-0.15	0.452	0.17	0.863
Which eye(s) affected by astigmatism: Left eye	-0.59	0.493	-0.41	0.463	-0.17	0.863
Diagnoses - main ICD10: C19 Malignant neoplasm of rectosigmoid junction	0.04	0.953	-0.11	0.849	0.17	0.863
Diagnoses - main ICD10: D12 Benign neoplasm of colon, rectum, anus and anal canal	0.01	0.956	0.03	0.633	-0.17	0.864
Pack years of smoking PREVIEW ONLY	0.10	0.187	0.08	0.047	0.17	0.864

Diagnoses - main ICD10: H11 Other disorders of conjunctiva	-0.13	0.638	-0.19	0.216	0.17	0.865
Diagnoses - main ICD10: R55 Syncope and collapse	-0.03	0.868	0.00	0.966	-0.17	0.867
Treatment/medication code: nicorandil	0.22	0.289	0.18	0.096	0.17	0.867
Non-cancer illness code, self-reported: joint disorder	0.12	0.595	0.08	0.480	0.17	0.867
Illnesses of siblings: Breast cancer	-0.08	0.711	-0.12	0.248	0.17	0.867
Treatment/medication code: kliovance 1mg/0.5mg tablet	-0.40	0.898	0.14	0.872	-0.17	0.868
Diagnoses - main ICD10: R53 Malaise and fatigue	0.10	0.736	0.05	0.705	0.17	0.868
Diagnoses - main ICD10: K25 Gastric ulcer	0.33	0.701	0.56	0.606	-0.17	0.868
Treatment/medication code: pantoprazole	0.02	0.937	0.05	0.591	-0.17	0.868
Cancer code, self-reported: chronic lymphocytic	0.01	0.970	-0.04	0.795	0.17	0.869
Non-cancer illness code, self-reported: bone disorder	0.15	0.647	0.21	0.214	-0.17	0.869
Systolic blood pressure, automated reading	-0.01	0.915	0.01	0.894	-0.16	0.870
Treatment/medication code: voltarol emulgel	0.05	0.946	0.25	0.786	-0.16	0.870
Treatment/medication code: ibuprofen	0.09	0.413	0.07	0.232	0.16	0.870
Non-cancer illness code, self-reported: obsessive compulsive disorder (ocd)	0.06	0.889	0.13	0.536	-0.16	0.871
Smoked cigarette or pipe within last hour	-0.28	0.789	-0.10	0.818	-0.16	0.872
Treatment/medication code: esomeprazole	-0.05	0.861	-0.09	0.454	0.16	0.872
Treatment/medication code: lodine 200mg tablet	0.18	0.522	0.13	0.389	0.16	0.872
Treatment/medication code: chlorpheniramine	-0.03	0.966	0.49	0.877	-0.16	0.873
Treatment/medication code: aciclovir	-0.41	0.588	-0.59	0.480	0.16	0.873
Diagnoses - main ICD10: G57 Mononeuropathies of lower limb	0.08	0.907	0.35	0.821	-0.16	0.874
Treatment/medication code: fucibet cream	-0.05	0.878	-0.11	0.569	0.16	0.874
Treatment/medication code: rhumalgan 25mg e/c tablet	-0.34	0.497	-0.25	0.425	-0.16	0.875
Father still alive	-0.02	0.887	-0.05	0.552	0.16	0.875
Treatment/medication code: verapamil	0.05	0.863	0.00	0.997	0.16	0.875
Treatment/medication code: calcium salts	-0.06	0.848	-0.11	0.479	0.16	0.875
Treatment/medication code: anusol cream	0.17	0.483	0.13	0.288	0.16	0.876
Diagnoses - main ICD10: I95 Hypotension	0.45	0.722	0.22	0.747	0.16	0.876
Eye problems/disorders: Macular degeneration	-0.05	0.881	0.01	0.963	-0.16	0.876
Non-cancer illness code, self-reported: diabetic eye disease	0.50	0.288	0.60	0.129	-0.15	0.877

Treatment/medication code: garlic product	0.05	0.816	0.09	0.400	-0.15	0.877
Diagnoses - main ICD10: C18 Malignant neoplasm of colon	-0.09	0.632	-0.05	0.539	-0.15	0.878
Underlying (primary) cause of death: ICD10: I42.0 Dilated cardiomyopathy	0.00	0.999	0.06	0.713	-0.15	0.878
Transport type for commuting to job workplace: Walk	-0.10	0.426	-0.08	0.190	-0.15	0.879
Cancer code, self-reported: lung cancer	0.45	0.732	0.23	0.751	0.15	0.879
Treatment/medication code: calcium+vitamin d 500units tablet	-0.54	0.664	-0.32	0.688	-0.15	0.880
Treatment/medication code: losartan potassium+hydrochlorothiazide 50mg/12.5mg tablet	-0.18	0.587	-0.12	0.509	-0.15	0.880
Treatment/medication code: kalms tablet	0.14	0.581	0.10	0.437	0.15	0.880
Ever had stillbirth, spontaneous miscarriage or termination	0.03	0.819	0.01	0.940	0.15	0.882
Pain type(s) experienced in last month: Pain all over the body	0.20	0.121	0.18	0.004	0.15	0.883
Non-cancer illness code, self-reported: rheumatoid arthritis	0.24	0.147	0.27	0.009	-0.15	0.884
Non-cancer illness code, self-reported: uterine fibroids	0.17	0.248	0.15	0.046	0.15	0.884
Treatment/medication code: sulphasalazine	-0.17	0.745	-0.26	0.444	0.15	0.884
Non-cancer illness code, self-reported: dry eyes	0.36	0.419	0.28	0.202	0.14	0.885
Treatment/medication code: femoston 1/10 tablet	0.03	0.936	-0.03	0.863	0.14	0.885
Years since last cervical smear test	-0.03	0.872	0.00	0.998	-0.14	0.886
No-wear time bias adjusted acceleration standard deviation	-0.12	0.212	-0.10	0.019	-0.14	0.888
Underlying (primary) cause of death: ICD10: C80.9 Malignant neoplasm, unspecified	0.06	0.873	0.00	0.996	0.14	0.888
Diagnoses - main ICD10: Q38 Other congenital malformations of tongue, mouth and pharynx	0.14	0.628	0.10	0.520	0.14	0.889
Average monthly fortified wine intake	0.01	0.976	-0.04	0.802	0.14	0.889
Diagnoses - main ICD10: O42 Premature rupture of membranes	0.04	0.917	-0.02	0.919	0.14	0.890
Treatment/medication code: cod liver oil capsule	-0.06	0.740	-0.03	0.717	-0.14	0.892
Cancer code, self-reported: rodent ulcer	-0.05	0.825	-0.09	0.564	0.13	0.893
Diagnoses - main ICD10: J34 Other disorders of nose and nasal sinuses	0.06	0.790	0.09	0.443	-0.13	0.893
Treatment/medication code: simvador 10mg tablet	0.34	0.431	0.41	0.164	-0.13	0.893
Transport type for commuting to job workplace: Cycle	-0.09	0.395	-0.07	0.094	-0.13	0.894
Treatment/medication code: tildiem 60mg m/r tablet	-0.05	0.815	-0.02	0.899	-0.13	0.895
Underlying (primary) cause of death: ICD10: K55.9 Vascular disorder of intestine, unspecified	0.01	0.971	-0.04	0.806	0.13	0.895

Treatment/medication code: becotide 50 inhaler	0.01	0.971	-0.02	0.833	0.13	0.896
Hand grip strength (right)	-0.01	0.843	0.00	0.894	-0.13	0.896
Treatment/medication code: colestyramine	0.43	0.574	0.56	0.438	-0.13	0.897
Treatment/medication code: oestrogen product	-0.17	0.711	-0.10	0.678	-0.13	0.897
Hair/balding pattern: Pattern 3	-0.01	0.941	0.01	0.892	-0.13	0.898
Diagnoses - main ICD10: E87 Other disorders of fluid, electrolyte and acid-base balance	0.10	0.813	0.16	0.514	-0.13	0.898
Number of live births	0.08	0.296	0.07	0.043	0.13	0.899
Non-cancer illness code, self-reported: umbilical hernia	0.17	0.468	0.14	0.256	0.13	0.899
Non-cancer illness code, self-reported: muscle/soft tissue problem	0.12	0.603	0.15	0.234	-0.13	0.900
Number of pregnancy terminations	-0.05	0.701	-0.03	0.658	-0.12	0.902
Treatment/medication code: selenium product	-0.05	0.856	-0.01	0.926	-0.12	0.903
Fracture resulting from simple fall	0.01	0.940	-0.01	0.905	0.12	0.906
Treatment/medication code: sotalol	-0.04	0.886	0.00	0.982	-0.12	0.907
Treatment/medication code: loratadine	0.32	0.137	0.29	0.013	0.12	0.908
Treatment/medication code: singulair 10mg tablet	0.15	0.762	0.08	0.717	0.12	0.908
Why reduced smoking: Financial reasons	0.25	0.630	0.18	0.468	0.12	0.908
Treatment/medication code: polyvinyl alcohol 1% eye drops	-0.10	0.774	-0.14	0.406	0.11	0.909
Diagnoses - main ICD10: I83 Varicose veins of lower extremities	-0.07	0.485	-0.05	0.357	-0.11	0.909
Illnesses of siblings: None of the above (group 2)	-0.02	0.861	-0.04	0.582	0.11	0.910
Treatment/medication code: propranolol	0.09	0.606	0.11	0.223	-0.11	0.910
Current employment status: Full or part-time student	0.21	0.367	0.18	0.281	0.11	0.911
Non-cancer illness code, self-reported: crohns disease	-0.01	0.952	0.01	0.905	-0.11	0.911
Diagnoses - main ICD10: N80 Endometriosis	-0.02	0.913	-0.04	0.622	0.11	0.911
Treatment/medication code: senna	0.20	0.466	0.24	0.099	-0.11	0.912
Family relationship satisfaction	0.09	0.325	0.08	0.074	0.11	0.914
Cancer code, self-reported: kidney/renal cell cancer	0.24	0.404	0.28	0.124	-0.11	0.914
Frequency of heavy DIY in last 4 weeks	0.05	0.692	0.06	0.368	-0.11	0.915
Diagnoses - main ICD10: L40 Psoriasis	0.03	0.909	0.00	1.000	0.11	0.916
Treatment/medication code: cosopt 2%/0.5% eye drops	-0.03	0.878	-0.01	0.948	-0.11	0.916

Diagnoses - main ICD10: L57 Skin changes due to chronic exposure to nonionising radiation	-0.22	0.524	-0.18	0.319	-0.10	0.917
Diagnoses - main ICD10: R12 Heartburn	0.26	0.707	0.36	0.605	-0.10	0.917
Diagnoses - main ICD10: G61 Inflammatory polyneuropathy	0.10	0.694	0.07	0.635	0.10	0.918
Diagnoses - main ICD10: K65 Peritonitis	-0.52	0.852	-0.22	0.843	-0.10	0.919
Non-cancer illness code, self-reported: duodenal ulcer	0.67	0.828	0.32	0.847	0.10	0.920
Mineral and other dietary supplements: Glucosamine	-0.05	0.502	-0.06	0.130	0.10	0.921
Non-cancer illness code, self-reported: scarlet fever / scarlatina	-0.20	0.627	-0.15	0.452	-0.10	0.921
Treatment/medication code: uniphyllin continus 200mg m/r tablet	0.59	0.710	0.41	0.678	0.10	0.922
Treatment/medication code: mirtazapine	-0.04	0.940	0.03	0.950	-0.10	0.922
Number of days/week of moderate physical activity 10+ minutes	-0.03	0.712	-0.02	0.624	-0.10	0.923
Duration of strenuous sports	-0.07	0.750	-0.04	0.691	-0.10	0.924
Used an inhaler for chest within last hour	0.12	0.570	0.15	0.141	-0.10	0.924
Treatment/medication code: fludrocortisone	0.19	0.531	0.22	0.222	-0.10	0.924
Diagnoses - main ICD10: I86 Varicose veins of other sites	0.16	0.556	0.13	0.320	0.09	0.924
Diagnoses - main ICD10: D28 Benign neoplasm of other and unspecified female genital organs	-0.16	0.513	-0.14	0.335	-0.09	0.925
Medication for cholesterol, blood pressure, diabetes, or take exogenous hormones: None of the above	-0.14	0.080	-0.15	0.006	0.09	0.925
Diagnoses - main ICD10: E16 Other disorders of pancreatic internal secretion	0.20	0.366	0.18	0.130	0.09	0.926
Treatment/medication code: fosinopril	0.15	0.571	0.18	0.216	-0.09	0.926
Underlying (primary) cause of death: ICD10: C16.9 Stomach, unspecified	0.05	0.865	0.02	0.888	0.09	0.927
Medication for pain relief, constipation, heartburn: Ranitidine (e.g. Zantac)	0.20	0.222	0.22	0.019	-0.09	0.927
Treatment/medication code: nitrolingual 400micrograms spray	0.08	0.744	0.05	0.727	0.09	0.927
Non-cancer illness code, self-reported: benign neuroma	0.18	0.472	0.16	0.217	0.09	0.928
Diagnoses - main ICD10: K50 Crohn's disease [regional enteritis]	0.11	0.487	0.10	0.340	0.09	0.929
Diagnoses - main ICD10: Z45 Adjustment and management of implanted device	0.07	0.849	0.11	0.518	-0.09	0.929
Diagnoses - main ICD10: D21 Other benign neoplasms of connective and other soft tissue	-0.19	0.485	-0.17	0.202	-0.09	0.931
Treatment/medication code: sandrena 0.5mg gel	-0.10	0.698	-0.13	0.345	0.09	0.931
Diagnoses - main ICD10: T81 Complications of procedures, not elsewhere classified	0.33	0.108	0.35	0.003	-0.09	0.931

Tinnitus: Yes, now a lot of the time	0.09	0.662	0.11	0.391	-0.08	0.932
Most recent bowel cancer screening	0.02	0.916	0.04	0.684	-0.08	0.933
Diagnoses - main ICD10: R33 Retention of urine	0.65	0.721	0.47	0.722	0.08	0.933
Illnesses of siblings: Stroke	-0.01	0.974	0.01	0.910	-0.08	0.933
Treatment/medication code: orlistat	0.37	0.628	0.46	0.502	-0.08	0.934
Diagnoses - main ICD10: S12 Fracture of neck	-0.03	0.931	-0.07	0.742	0.08	0.935
Length of menstrual cycle	0.04	0.749	0.03	0.601	0.08	0.935
Diagnoses - main ICD10: M67 Other disorders of synovium and tendon	0.23	0.389	0.21	0.168	0.08	0.935
Non-cancer illness code, self-reported: fracture shoulder / scapula	-0.07	0.878	-0.03	0.901	-0.08	0.936
Non-cancer illness code, self-reported: hiv/aids	-0.35	0.349	-0.32	0.220	-0.08	0.937
Treatment/medication code: rino clenil 50micrograms nasal spray	0.17	0.463	0.15	0.178	0.08	0.937
Non-cancer illness code, self-reported: hypertension	0.10	0.107	0.09	0.026	0.08	0.937
Treatment/medication code: moxonidine	0.26	0.640	0.31	0.462	-0.08	0.937
Diagnoses - main ICD10: I84 Haemorrhoids	0.11	0.380	0.10	0.100	0.08	0.938
Diagnoses - main ICD10: D64 Other anaemias	0.31	0.918	1.10	0.910	-0.08	0.938
Cancer code, self-reported: colon cancer/sigmoid cancer	0.06	0.865	0.03	0.886	0.08	0.940
Hair/balding pattern: Pattern 2	-0.06	0.435	-0.07	0.093	0.07	0.941
Reason for reducing amount of alcohol drunk: Health precaution	-0.04	0.627	-0.04	0.481	-0.07	0.942
Diagnoses - main ICD10: I30 Acute pericarditis	-0.07	0.792	-0.05	0.723	-0.07	0.944
Duration of fitness test	-0.05	0.816	-0.03	0.769	-0.07	0.944
Treatment/medication code: flax oil tablet	0.13	0.431	0.14	0.087	-0.07	0.945
Handedness (chirality/laterality): Use both right and left hands equally	0.17	0.330	0.15	0.096	0.07	0.946
Non-cancer illness code, self-reported: infectious mononucleosis / glandular fever / epstein barr virus (ebv)	0.00	0.994	0.02	0.886	-0.07	0.946
Non-cancer illness code, self-reported: unclassifiable	0.08	0.739	0.06	0.635	0.07	0.946
Treatment/medication code: lipantil micro 67mg capsule	-0.22	0.752	-0.17	0.648	-0.07	0.947
Treatment/medication code: antihistamine 60mg tablet	0.19	0.638	0.16	0.427	0.06	0.948
Treatment/medication code: cerazette 75micrograms tablet	0.15	0.613	0.13	0.413	0.06	0.949
Treatment/medication code: piriton 4mg tablet	0.14	0.648	0.11	0.519	0.06	0.949
Diagnoses - main ICD10: E23 Hypofunction and other disorders of pituitary gland	-0.02	0.961	0.01	0.965	-0.06	0.949

Diagnoses - main ICD10: S82 Fracture of lower leg, including ankle	0.37	0.779	0.50	0.747	-0.06	0.949
Number of full sisters	0.09	0.402	0.10	0.122	-0.06	0.951
Treatment/medication code: gabapentin	0.02	0.917	0.01	0.928	0.06	0.951
Birth weight	-0.08	0.264	-0.08	0.034	0.06	0.952
Illnesses of mother: Bowel cancer	-0.14	0.389	-0.15	0.098	0.06	0.952
Diagnoses - main ICD10: F30 Manic episode	0.01	0.988	0.04	0.890	-0.06	0.954
Types of transport used (excluding work): Walk	-0.03	0.725	-0.02	0.650	-0.06	0.955
Underlying (primary) cause of death: ICD10: G35 Multiple sclerosis	0.12	0.908	0.05	0.923	0.06	0.955
Treatment/medication code: seretide 50 evohaler	0.10	0.411	0.09	0.109	0.06	0.955
Cancer code, self-reported: cin/pre-cancer cells cervix	0.02	0.925	0.04	0.776	-0.06	0.956
Treatment/medication code: peptac liquid	0.01	0.990	-0.03	0.944	0.05	0.956
Diagnoses - main ICD10: B34 Viral infection of unspecified site	0.21	0.755	0.25	0.572	-0.05	0.957
Vascular/heart problems diagnosed by doctor: None of the above	-0.10	0.121	-0.10	0.014	0.05	0.959
Diagnoses - main ICD10: J43 Emphysema	0.01	0.965	0.03	0.801	-0.05	0.959
Time employed in main current job	-0.09	0.511	-0.08	0.425	-0.05	0.959
Diagnoses - main ICD10: D25 Leiomyoma of uterus	0.10	0.508	0.10	0.166	-0.05	0.960
Non-cancer illness code, self-reported: oesophagitis/barretts oesophagus	-0.19	0.524	-0.17	0.319	-0.05	0.961
Number of cigarettes currently smoked daily (current cigarette smokers)	0.06	0.649	0.06	0.436	0.05	0.963
Underlying (primary) cause of death: ICD10: K70.4 Alcoholic hepatic failure	0.13	0.695	0.11	0.519	0.05	0.963
Treatment/medication code: b12 - cyanocobalamin prep	0.00	0.993	0.03	0.928	-0.05	0.964
Treatment/medication code: tolterodine l-tartrate	0.25	0.388	0.27	0.085	-0.05	0.964
Diagnoses - main ICD10: N63 Unspecified lump in breast	-0.10	0.732	-0.11	0.473	0.04	0.965
Treatment/medication code: lacidipine	-0.12	0.770	-0.14	0.501	0.04	0.965
Diagnoses - main ICD10: M95 Other acquired deformities of musculoskeletal system and connective tissue	-0.01	0.975	-0.02	0.867	0.04	0.965
Daytime dozing / sleeping (narcolepsy)	0.10	0.097	0.10	7.00E-04	-0.04	0.966
Diagnoses - main ICD10: S13 Dislocation, sprain and strain of joints and ligaments at neck level	0.20	0.578	0.18	0.283	0.04	0.967
Diagnoses - main ICD10: S09 Other and unspecified injuries of head	0.14	0.368	0.14	0.194	0.04	0.968
Non-cancer illness code, self-reported: peripheral nerve injury	-0.20	0.580	-0.22	0.300	0.04	0.968
Underlying (primary) cause of death: ICD10: C83.3 Large cell (diffuse)	-0.53	0.502	-0.49	0.503	-0.04	0.968

Cancer code, self-reported: squamous cell carcinoma	-0.13	0.637	-0.12	0.392	-0.04	0.970
Treatment/medication code: salmeterol	0.12	0.571	0.12	0.294	-0.04	0.970
Non-cancer illness code, self-reported: rosacea	0.21	0.563	0.19	0.373	0.04	0.971
Diagnoses - main ICD10: N13 Obstructive and reflux uropathy	0.11	0.693	0.10	0.532	0.04	0.971
Diastolic blood pressure, automated reading	0.03	0.579	0.04	0.391	-0.04	0.971
Treatment/medication code: almotriptan	-0.05	0.812	-0.05	0.699	-0.03	0.972
Pack years adult smoking as proportion of life span exposed to smoking PREVIEW ONLY	0.08	0.240	0.08	0.063	0.03	0.973
Illnesses of mother: Parkinson's disease	-0.30	0.553	-0.33	0.427	0.03	0.974
Diagnoses - main ICD10: H02 Other disorders of eyelid	0.40	0.585	0.43	0.499	-0.03	0.974
Treatment/medication code: glucophage 500mg tablet	0.05	0.903	0.06	0.754	-0.03	0.974
Diagnoses - main ICD10: N43 Hydrocele and spermatocele	0.00	0.994	0.00	0.955	-0.03	0.974
Non-cancer illness code, self-reported: systemic lupus erythematosis/sle	0.45	0.426	0.43	0.261	0.03	0.975
Diagnoses - main ICD10: I62 Other nontraumatic intracranial haemorrhage	0.21	0.582	0.20	0.375	0.03	0.975
Relative age of first facial hair	-0.04	0.493	-0.05	0.189	0.03	0.975
Treatment/medication code: lipitor 10mg tablet	0.27	0.437	0.25	0.278	0.03	0.975
Diagnoses - main ICD10: K92 Other diseases of digestive system	0.16	0.510	0.15	0.215	0.03	0.978
Non-cancer illness code, self-reported: pleurisy	0.25	0.410	0.24	0.102	0.03	0.978
Treatment/medication code: co-proxamol	0.03	0.914	0.02	0.895	0.03	0.979
Chest pain due to walking ceases when standing still	0.12	0.754	0.13	0.466	-0.03	0.979
Diagnoses - main ICD10: T50 Poisoning by diuretics and other and unspecified drugs, medicaments and biological substances	0.04	0.863	0.04	0.700	-0.03	0.979
Illnesses of father: Bowel cancer	-0.08	0.763	-0.07	0.618	-0.03	0.979
Diagnoses - main ICD10: K62 Other diseases of anus and rectum	0.20	0.179	0.21	0.013	-0.03	0.980
Non-cancer illness code, self-reported: malabsorption/coeliac disease	0.23	0.165	0.22	0.033	0.03	0.980
Treatment/medication code: cinnarizine	0.15	0.553	0.15	0.245	0.02	0.980
Underlying (primary) cause of death: ICD10: K76.0 Fatty (change of) liver, not elsewhere classified	-0.19	0.550	-0.20	0.278	0.02	0.980
Home area population density - urban or rural: Scotland - Accessible Rural	-0.16	0.688	-0.17	0.479	0.02	0.981
Doctor diagnosed idiopathic pulmonary fibrosis	0.50	0.467	0.48	0.387	0.02	0.981
Non-cancer illness code, self-reported: rheumatic fever	0.11	0.761	0.10	0.586	0.02	0.982

Treatment/medication code: candesartan cilexetil	0.15	0.290	0.16	0.021	-0.02	0.983
Underlying (primary) cause of death: ICD10: I61.9 Intracerebral haemorrhage, unspecified	-0.50	0.908	-0.39	0.903	-0.02	0.983
Treatment/medication code: solpadol caplet	-0.19	0.674	-0.18	0.520	-0.02	0.984
Non-cancer illness code, self-reported: inflammatory bowel disease	0.14	0.696	0.14	0.470	-0.02	0.984
Years since last breast cancer screening / mammogram	-0.10	0.655	-0.11	0.408	0.02	0.984
Non-cancer illness code, self-reported: sarcoidosis	0.19	0.263	0.19	0.050	0.02	0.985
Diagnoses - main ICD10: N21 Calculus of lower urinary tract	-0.16	0.800	-0.17	0.727	0.02	0.987
Underlying (primary) cause of death: ICD10: C71.0 Cerebrum, except lobes and ventricles	0.19	0.656	0.20	0.479	-0.01	0.989
Diagnoses - main ICD10: R59 Enlarged lymph nodes	0.01	0.985	0.01	0.938	-0.01	0.991
Diagnoses - main ICD10: F32 Depressive episode	0.15	0.610	0.15	0.271	-0.01	0.991
Average monthly champagne plus white wine intake	0.06	0.811	0.06	0.608	0.01	0.992
Diagnoses - main ICD10: C09 Malignant neoplasm of tonsil	0.31	0.849	0.33	0.805	-0.01	0.993
Mouth/teeth dental problems: Bleeding gums	0.07	0.443	0.07	0.243	-0.01	0.993
Tinnitus: Yes, now most or all of the time	0.22	0.090	0.22	9.00E-04	-0.01	0.993
Non-cancer illness code, self-reported: other abdominal problem	0.08	0.780	0.09	0.620	-0.01	0.994
Diagnoses - main ICD10: K44 Diaphragmatic hernia	0.14	0.399	0.14	0.130	-0.01	0.994
Treatment/medication code: tacrolimus	0.27	0.527	0.27	0.288	-0.01	0.995
Medication for cholesterol, blood pressure, diabetes, or take exogenous hormones: Hormone replacement therapy	-0.01	0.936	-0.01	0.895	-0.01	0.995
Treatment/medication code: solpadeine capsule	0.37	0.963	0.31	0.961	0.01	0.995
Treatment/medication code: kelp+garlic product	0.21	0.399	0.21	0.145	0.00	0.996
Diagnoses - main ICD10: O64 Obstructed labour due to malposition and malpresentation of foetus	0.08	0.788	0.08	0.566	0.00	0.996
Non-cancer illness code, self-reported: otosclerosis	0.44	0.855	0.45	0.552	0.00	0.997
Pain type(s) experienced in last month: Back pain	0.13	0.062	0.13	8.00E-04	0.00	0.998
Diagnoses - main ICD10: D69 Purpura and other haemorrhagic conditions	0.40	0.458	0.40	0.210	0.00	0.999
Type of tobacco currently smoked: Hand-rolled cigarettes	-0.06	0.827	-0.06	0.596	0.00	0.999
Diagnoses - main ICD10: C21 Malignant neoplasm of anus and anal canal	-0.04	0.959	-0.04	0.928	0.00	0.999

Table S9: Results from enrichment analysis conducted with respect to hyperthyroidism and hypothyroidism.

**Enrichment**		**Hyperthyroidism**		**Hypothyroidism**		**Difference**	
	**Genes, N**	***β***	***p***	***β***	***p***	***z***	***p***
GO~mhc_class_ii_protein_complex	14	3.33	**4.58E-30**	3.18	**2.08E-18**	0.320	7.49E-01
KEGG~autoimmune_thyroid_disease	49	1.5	**4.41E-21**	1.9	**6.00E-22**	-1.567	1.17E-01
GO~mhc_protein_complex	24	1.66	**3.42E-19**	1.32	**7.16E-09**	1.138	2.55E-01
GO~mhc_class_ii_receptor_activity	10	2.34	**3.60E-17**	2.06	**1.91E-09**	0.626	5.31E-01
KEGG~allograft_rejection	34	1.36	**9.42E-16**	1.6	**2.06E-14**	-0.883	3.77E-01
KEGG~type_i_diabetes_mellitus	41	1.09	**2.29E-14**	1.08	**9.20E-10**	0.044	9.65E-01
KEGG~asthma	27	1.27	**2.51E-14**	1.32	**1.32E-10**	-0.186	8.52E-01
KEGG~graft_versus_host_disease	37	1.03	**8.75E-12**	1.21	**1.44E-10**	-0.734	4.63E-01
KEGG~intestinal_immune_network_for_iga_production	43	0.989	**3.31E-11**	1.16	**3.34E-10**	-0.709	4.78E-01
GO~mhc_class_ii_protein_complex_binding	16	1.39	**8.41E-10**	0.689	8.11E-03	1.906	5.67E-02
KEGG~viral_myocarditis	66	0.626	**3.48E-08**	0.558	5.60E-05	0.368	7.13E-01
GO~regulation_of_immune_response	780	0.171	**7.93E-08**	0.316	**3.53E-15**	-2.789	5.29E-03
GO~antigen_binding	79	0.546	**1.69E-07**	0.434	5.59E-04	0.656	5.12E-01
KEGG~systemic_lupus_erythematosus	124	0.49	**5.01E-07**	0.488	**4.58E-05**	0.012	9.90E-01
Gaurnier~psmd4_targets	66	0.528	**6.21E-07**	0.553	**2.27E-05**	-0.144	8.85E-01
GO~mhc_protein_complex_binding	19	0.978	**6.62E-07**	0.454	3.57E-02	1.622	1.05E-01
GO~positive_regulation_of_cell_activation	275	0.262	**8.44E-07**	0.539	**1.32E-15**	-3.169	1.53E-03
KEGG~leishmania_infection	66	0.559	**9.05E-07**	0.656	**3.34E-06**	-0.518	6.04E-01
GO~positive_regulation_of_immune_response	505	0.187	**1.02E-06**	0.342	**1.44E-12**	-2.465	1.37E-02
KEGG~antigen_processing_and_presentation	82	0.512	**1.76E-06**	0.675	**4.44E-07**	-0.928	3.54E-01
GO~interferon_gamma_mediated_signaling_pathway	66	0.53	**1.96E-06**	0.863	**7.38E-10**	-1.815	6.96E-02
GO~peptide_antigen_binding	26	0.861	**2.01E-06**	1.16	**2.89E-07**	-1.003	3.16E-01
GO~positive_regulation_of_cell_cell_adhesion	229	0.283	**2.02E-06**	0.475	**2.71E-10**	-1.956	5.05E-02
GO~lumenal_side_of_membrane	28	0.8	**4.23E-06**	1.29	**3.47E-09**	-1.710	8.73E-02
GO~positive_regulation_of_cytokine_production	356	0.209	**4.55E-06**	0.228	5.08E-05	-0.253	8.00E-01
GO~positive_regulation_of_immune_system_process	797	0.141	4.76E-06	0.301	**1.38E-14**	-3.150	1.63E-03

GO~antigen_receptor_mediated_signaling_pathway	167	0.299	9.03E-06	0.547	**1.40E-10**	-2.232	2.56E-02
GO~immune_response	1003	0.127	1.36E-05	0.272	**2.42E-13**	-3.007	2.64E-03
GO~positive_regulation_of_t_cell_proliferation	90	0.391	3.18E-05	0.671	**1.77E-08**	-1.791	7.33E-02
GO~innate_immune_response	551	0.162	3.61E-05	0.29	**5.46E-09**	-1.969	4.90E-02
GO~regulation_of_cell_activation	448	0.17	5.08E-05	0.405	**4.27E-14**	-3.372	7.47E-04
GO~regulation_of_b_cell_activation	97	0.356	8.83E-05	0.656	**1.34E-08**	-1.982	4.75E-02
GO~cellular_response_to_cytokine_stimulus	585	0.147	9.28E-05	0.243	**3.45E-07**	-1.530	1.26E-01
GO~activation_of_immune_response	374	0.171	9.76E-05	0.359	**1.83E-10**	-2.561	1.04E-02
Reactome~phosphorylation_of_cd3_and_tcr_zeta_chains	15	0.951	9.80E-05	2.03	**8.33E-11**	-2.652	8.00E-03
GO~regulation_of_t_cell_proliferation	139	0.29	1.36E-04	0.521	**7.42E-08**	-1.818	6.90E-02
GO~regulation_of_homotypic_cell_cell_adhesion	293	0.196	1.52E-04	0.425	**1.54E-10**	-2.648	8.10E-03
GO~positive_regulation_of_leukocyte_proliferation	131	0.287	1.55E-04	0.578	**2.75E-09**	-2.289	2.21E-02
GO~positive_regulation_of_b_cell_activation	64	0.396	1.62E-04	0.642	**1.42E-06**	-1.400	1.61E-01
GO~macrophage_activation_involved_in_immune_response	11	1.08	1.68E-04	1.98	**7.02E-08**	-1.866	6.20E-02
GO~regulation_of_immune_system_process	1312	0.0913	1.88E-04	0.232	**1.96E-13**	-3.435	5.93E-04
GO~cellular_response_to_interferon_gamma	117	0.309	2.47E-04	0.677	**4.14E-10**	-2.605	9.18E-03
GO~regulation_of_lymphocyte_differentiation	126	0.279	2.48E-04	0.5	**2.63E-07**	-1.728	8.40E-02
GO~t_cell_receptor_signaling_pathway	139	0.264	2.74E-04	0.5	**6.97E-08**	-1.938	5.26E-02
GO~regulation_of_leukocyte_proliferation	198	0.226	3.01E-04	0.537	**2.83E-11**	-2.958	3.09E-03
GO~regulation_of_cell_cell_adhesion	366	0.165	3.61E-04	0.355	**2.51E-09**	-2.442	1.46E-02
GO~cytokine_mediated_signaling_pathway	431	0.154	4.04E-04	0.299	**8.05E-08**	-1.981	4.76E-02
GO~positive_regulation_of_interferon_gamma_production	63	0.397	4.20E-04	0.721	**5.60E-07**	-1.706	8.80E-02
Biocarta~ctla4_pathway	19	0.782	5.34E-04	1.85	**2.37E-10**	-2.802	5.09E-03
GO~adaptive_immune_response	239	0.198	5.40E-04	0.399	**6.02E-08**	-2.081	3.74E-02
GO~regulation_of_innate_immune_response	337	0.162	5.53E-04	0.316	**1.62E-07**	-1.945	5.17E-02
Reactome~interferon_gamma_signaling	58	0.413	6.04E-04	1.11	**1.59E-12**	-3.438	5.85E-04
Reactome~pd1_signaling	16	0.711	8.02E-04	2.03	**2.02E-13**	-3.672	2.41E-04
GO~response_to_cytokine	686	0.107	1.30E-03	0.226	**1.72E-07**	-2.089	3.67E-02
Reactome~translocation_of_zap_70_to_immunological_synapse	13	0.795	1.35E-03	2.26	**3.76E-12**	-3.468	5.25E-04
GO~response_to_interferon_gamma	138	0.237	1.58E-03	0.61	**5.26E-10**	-2.910	3.61E-03

GO~immune_system_process	1854	0.0648	1.64E-03	0.211	**7.32E-15**	-4.161	3.17E-05
GO~lymphocyte_costimulation	67	0.338	1.70E-03	0.813	**7.25E-09**	-2.588	9.64E-03
GO~immune_response_regulating_cell_surface_receptor_signaling_pathway	277	0.155	1.83E-03	0.394	**1.26E-09**	-2.817	4.85E-03
GO~regulation_of_leukocyte_differentiation	223	0.172	2.66E-03	0.369	**8.13E-07**	-1.998	4.57E-02
GO~regulation_of_t_cell_differentiation	104	0.243	3.03E-03	0.557	**2.01E-07**	-2.225	2.61E-02
Basso~cd40_signaling_up	99	0.237	3.46E-03	0.511	**1.37E-06**	-1.959	5.01E-02
Biocarta~th1th2_pathway	18	0.625	3.70E-03	1.4	**7.77E-07**	-2.083	3.72E-02
GO~t_cell_differentiation	117	0.219	3.79E-03	0.476	**1.53E-06**	-1.965	4.95E-02
GO~positive_regulation_of_response_to_stimulus	1820	0.0577	4.59E-03	0.139	**2.32E-07**	-2.299	2.15E-02
GO~immune_effector_process	440	0.114	4.84E-03	0.257	**1.54E-06**	-2.027	4.27E-02
GO~positive_regulation_of_cell_adhesion	357	0.121	6.17E-03	0.32	**5.31E-08**	-2.581	9.85E-03
GO~leukocyte_activation	395	0.111	7.19E-03	0.366	**4.43E-11**	-3.522	4.28E-04
Reactome~tcr_signaling	50	0.326	7.65E-03	0.856	**1.58E-07**	-2.468	1.36E-02
GO~defense_response	1139	0.0667	9.30E-03	0.173	**4.81E-07**	-2.354	1.86E-02
Reactome~generation_of_second_messenger_molecules	25	0.43	1.05E-02	1.24	**3.81E-08**	-2.731	6.31E-03
GO~immune_system_development	560	0.0854	1.16E-02	0.268	**5.09E-09**	-3.042	2.35E-03
Zheng~foxp3_targets_up	26	0.408	1.19E-02	1.03	**2.36E-06**	-2.154	3.12E-02
Reactome~immune_system	857	0.0679	1.50E-02	0.22	**7.90E-09**	-3.046	2.32E-03
Reactome~adaptive_immune_system	496	0.0853	1.54E-02	0.231	**1.25E-06**	-2.312	2.08E-02
Reactome~downstream_tcr_signaling	34	0.355	1.55E-02	1.08	**7.17E-08**	-2.762	5.75E-03
PID~nfat_tfpathway	44	0.297	1.79E-02	1.15	**3.45E-11**	-3.772	1.62E-04
GO~positive_regulation_of_lymphocyte_differentiation	75	0.218	1.81E-02	0.576	**4.24E-06**	-2.161	3.07E-02
GO~lymphocyte_activation	324	0.0963	2.59E-02	0.35	**6.35E-09**	-3.210	1.33E-03
Reactome~costimulation_by_the_cd28_family	60	0.235	2.64E-02	0.921	**5.42E-10**	-3.545	3.92E-04
GO~regulation_of_cell_adhesion	604	0.0716	2.76E-02	0.247	**5.39E-08**	-2.939	3.29E-03
KEGG~t_cell_receptor_signaling_pathway	104	0.155	4.51E-02	0.574	**2.31E-07**	-2.865	4.17E-03
PID~il12_2pathway	62	0.192	4.71E-02	0.676	**1.10E-06**	-2.638	8.35E-03
GO~regulation_of_response_to_stress	1414	0.0405	4.82E-02	0.139	**2.38E-06**	-2.532	1.13E-02
GO~leukocyte_differentiation	279	0.0891	4.95E-02	0.393	**2.43E-09**	-3.528	4.18E-04
GO~lymphocyte_differentiation	200	0.104	5.00E-02	0.424	**3.67E-08**	-3.164	1.56E-03

PID~il12_stat4_pathway	33	0.27	6.32E-02	1.15	**9.37E-08**	-3.117	1.83E-03
GO~cell_activation	542	0.0569	7.24E-02	0.28	**4.05E-09**	-3.585	3.37E-04
GO~regulation_of_b_cell_proliferation	54	0.163	1.03E-01	0.744	**1.73E-06**	-2.827	4.70E-03
Reactome~interferon_alpha_beta_signaling	61	0.137	1.51E-01	0.766	**1.82E-06**	-2.968	3.00E-03
GO~positive_regulation_of_cytokine_biosynthetic_process	57	0.0729	2.75E-01	0.789	**1.06E-07**	-3.674	2.39E-04
Zheng~bound_by_foxp3	475	0.0234	2.91E-01	0.277	**8.29E-08**	-3.742	1.83E-04
GO~regulation_of_cytokine_biosynthetic_process	91	0.0394	3.47E-01	0.628	**2.32E-07**	-3.695	2.20E-04
Osman~bladder_cancer_dn	361	0.0137	3.82E-01	0.266	**1.29E-06**	-3.467	5.25E-04
Lu~il4_signaling	92	0.0216	4.10E-01	0.639	**2.94E-08**	-4.079	4.52E-05
PID~il27_pathway	26	0.0235	4.51E-01	1.27	**4.66E-08**	-4.095	4.22E-05
Reactome~regulation_of_ifng_signaling	12	-0.03	5.45E-01	1.67	**1.89E-07**	-4.030	5.58E-05
KEGG~jak_stat_signaling_pathway	147	-0.0137	5.67E-01	0.582	**4.41E-09**	-4.592	**4.39E-06**
GO~rac_guanyl_nucleotide_exchange_factor_activity	13	-0.36	9.00E-01	1.83	**8.01E-08**	-4.888	**1.02E-06**
